# Ensemble-AMPPred: Robust AMP Prediction and Recognition Using the Ensemble Learning Method with a New Hybrid Feature for Differentiating AMPs

**DOI:** 10.3390/genes12020137

**Published:** 2021-01-21

**Authors:** Supatcha Lertampaiporn, Tayvich Vorapreeda, Apiradee Hongsthong, Chinae Thammarongtham

**Affiliations:** National Center for Genetic Engineering and Biotechnology, Biochemical Engineering and Systems Biology Research Group, National Science and Technology Development Agency, King Mongkut’s University of Technology Thonburi, Khun Thian Bangkok 10150, Thailand; supatcha.ler@biotec.or.th (S.L.); tayvich.vor@biotec.or.th (T.V.); apiradee@biotec.or.th (A.H.)

**Keywords:** antimicrobial peptides, AMP prediction, heterogeneous ensemble machine learning, MaxProbVote, logistic regression

## Abstract

Antimicrobial peptides (AMPs) are natural peptides possessing antimicrobial activities. These peptides are important components of the innate immune system. They are found in various organisms. AMP screening and identification by experimental techniques are laborious and time-consuming tasks. Alternatively, computational methods based on machine learning have been developed to screen potential AMP candidates prior to experimental verification. Although various AMP prediction programs are available, there is still a need for improvement to reduce false positives (FPs) and to increase the predictive accuracy. In this work, several well-known single and ensemble machine learning approaches have been explored and evaluated based on balanced training datasets and two large testing datasets. We have demonstrated that the developed program with various predictive models has high performance in differentiating between AMPs and non-AMPs. Thus, we describe the development of a program for the prediction and recognition of AMPs using MaxProbVote, which is an ensemble model. Moreover, to increase prediction efficiency, the ensemble model was integrated with a new hybrid feature based on logistic regression. The ensemble model integrated with the hybrid feature can effectively increase the prediction sensitivity of the developed program called Ensemble-AMPPred, resulting in overall improvements in terms of both sensitivity and specificity compared to those of currently available programs.

## 1. Introduction

Antimicrobial peptides (AMPs), a group of natural peptides, have a significant role in the immune system. There are various types of peptides with antimicrobial activities, such as antibacterial, antifungal, antiviral, and anticancer peptides. These peptides have been found to be effective against disease-causing pathogens. Due to the increase in antibiotic resistance becoming a major global health problem, novel anti-infective therapies are needed [1,2]. Basically, AMPs have abilities to kill microbes and other pathogens but do not cause drug resistance in bacteria and have received great attention as a promising/potential alternative to conventional antibiotics. [2,3,4] Action mechanisms of AMPs include various mechanisms, such as “barrel-stave”, “carpet”, or “toroidal-pore” mechanisms, to disrupt the cell membrane or intracellular functions of microbes. AMP characteristics, including amino acid composition, amphipathic structure, cationic charge, and size contribute in facilitating AMP interaction and the insertion into membranes of pathogens resulting in pore forming and membrane disruption [2,5,6]. AMPs can also stimulate the immune system to work together efficiently [2]. Therefore, research studies on AMPs have received much attention and have been widely investigated for use as another option, as a potential alternative or in conjunction with current antibiotic therapeutics [7].

Finding new AMPs from various organisms is currently receiving significant attention. However, large-scale identification through wet lab experiments is costly, time consuming, and resource intensive [1,8]. Therefore, developing a computation program for screening AMPs with high accuracy and high effectiveness can help such complicated tasks. An efficient computational machine learning predictive tool is required to screen antimicrobial candidate sequences prior to in vitro experimentation [1,7,9]. Several antimicrobial prediction tools have been designed and developed, as summarized in Table 1. These diverse prediction tools have been developed using different data features and different machine learning methods. Therefore, their performances differ depending on the nature of the training technique and data features. Most existing methods use single classifier models such as support vector machine (SVM), discriminant analysis, fuzzy K-nearest neighbors, and deep learning. Some methods use homologous ensemble, random forests, which is a committee of decision tree models. However, several other types of machine learning techniques and heterogeneous ensemble techniques have not been applied in this AMPs prediction problem. Using different machine learning techniques may provide a prediction result of AMP candidates that remain to be discovered. Therefore, other types of machine learning and heterogeneous ensembles techniques should be explored.

Comparison of the performances of the available AMP prediction tools is difficult because different testing datasets are used for benchmarking these predictors [17]. Based on our preliminary study, we collected a benchmark AMP dataset S [13] composed of 920 AMPs and 920 non-AMPs and used it in testing current existing AMP prediction programs. From this preliminary review, we found that the false predictive answers of each program are different. This suggests that there is a different distribution of unpredictable answers due to the use of different models and features. Therefore, each program has gaps that should be considered for improvement, especially reducing false positives (FPs) and increasing predictive accuracy, in terms of both specificity and sensitivity. According to the issues mentioned above, we aim to (1) integrate different learning models using ensemble learning techniques to reduce FPs and to increase predictive accuracy and (2) use diverse informative features that contain sufficient discrimination information and are strongly related to AMP sequences.

Ensemble learning techniques are able to increase the accuracy and reduce the FPs of the prediction. The combination of predictions by different algorithms using different methods can reduce errors in bias or variance or otherwise reduce both bias and variance values found in a single algorithm through the voting of diverse algorithms. Moreover, for problems with complex decision boundaries, an appropriate combination of decision boundaries of various single models can learn the complex boundary of the problem [18]. The popular ensemble methods for incorporating individual classification models are bagging and boosting. There are different training procedures as follows. The bagging or bootstrap aggregating method [19] builds different classifiers from random bootstrapping of different training subsets. Therefore, individual models are different from each other, then reducing variance errors. Boosting [20] builds classifier models in incrementally sequential/linear combinations by adjusting the weight to improve the prediction values of the previous model and therefore can reduce the model bias [21].

Ensemble learning combines multiple points of view from different classifiers on the same problem domain to obtain a more accurate and robust (stable) prediction. In addition, this makes the ensemble model more generalizable with new data [22]. It also helps in reducing the overfitting problem found in single classification models [23], which makes it impossible to correctly predict new data. Moreover, the voting of heterogeneous methods can alleviate conflicting predictions found in single models.

The factors of a prediction method are a composite of good unbiased training data, a discriminative feature subset, and a suitable learning algorithm. To make the algorithm capable of learning patterns and distinguishing AMPs from other sequences, feature extraction, feature engineering, and feature selection became an important part of finding good representative features or informative features that can capture AMP patterns and increase the efficiency of predictions.

In this work, AMP prediction models based on ensemble methods, such as random forest (RF), max probability voting (MaxProbVote), majority voting, adaptive boosting (AdaBoost), and extreme gradient boosting (XGBoost), were built. In addition, we also compared various single models (support vector machine (SVM), naïve Bayes, logistic regression (LR), decision tree, multilayer perceptron (MLP), and K-nearest neighbor (KNN)). We collected and extracted various informative features related to AMP characteristics. The ability to train models relied substantially on a good representation of features that can detect the pattern of AMPs. Therefore, the extraction of data characteristics into data vectors was performed using a variety of 517 peptide features. Later, in addition to 517 features, we included a feature engineering process to explore the characteristics of AMPs and constructed a hybrid feature that combines four single preselected features based on the logistic regression equation. We observed that with the hybrid features integrated into the ensemble models, the sensitivity was between 93.39 and 97.51% for testing dataset 1, and the area under the receiver operating characteristic (ROC) curve (AUC) improved to between 0.917 and 0.946.

## 2. Materials and Methods

### 2.1. Workflow of Ensemble-AMPPred

**Figure 1:**
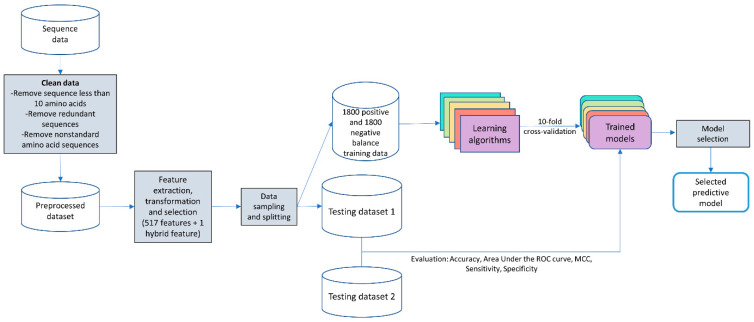
Flowchart of building a predictive model.

The proposed predictive program was designed and built, as shown in Figure 1.

### 2.2. Dataset Preparation

**Figure 2:**
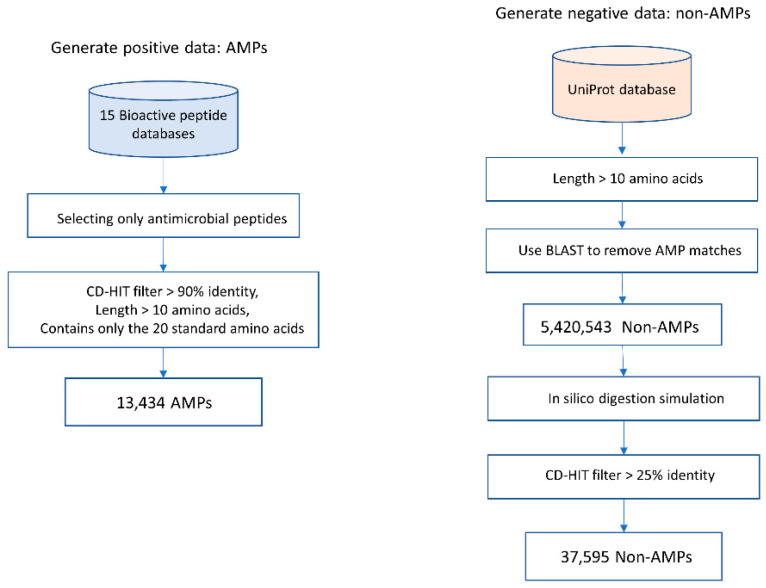
Steps for data collection and preparation.

Data collection used in training and testing of models is shown in Figure 2.

**Figure 3:**
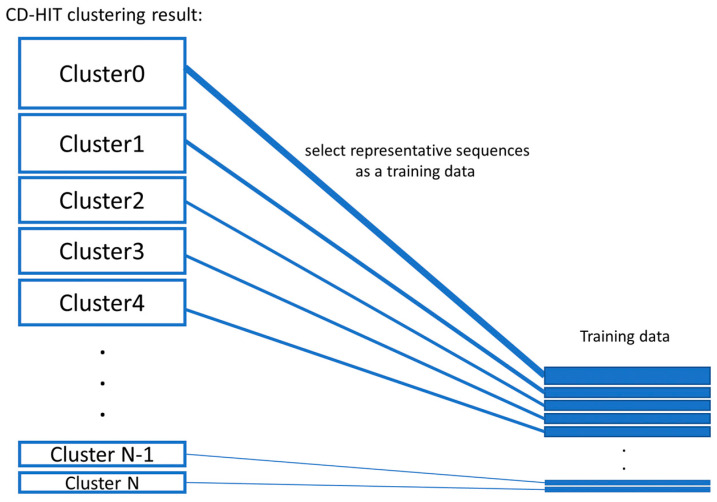
Proportionate stratified random sampling.

1. AMP data were collected from 15 public bioactive peptide databases, as listed in Table 2. Only peptides that have description-matched antimicrobial activities were selected. Peptide sequences with lengths <10 amino acids were discarded. To reduce data redundancy, we applied the Cluster Database at High Identity with Tolerance (CD-HIT) program [24] with threshold of 0.9 (90% sequence similarity). A total of 13,434 peptides were used as positive sequence data. Notably, lower sequence similarity thresholds (less than 50%) might reduce the sequence homology bias and could improve the model reliability [25]. Since AMPs are highly heterogeneous substances and there are likely various novel subtypes of AMPs that have not been discovered, using a threshold of 0.9 is applicable to identifying a novel AMP sequence.
2. Currently, there is no database of experimentally verified non-AMP available. Therefore, we build negative data using the approach described in [13,16]. Negative data or non-AMP data were collected from the UniProt [26] database (February 2020) by choosing only proteins that do not contain functional information related to antimicrobial activity and do not have a secretory signal peptide position. The basic local alignment search tool (BLAST) was used to filter out AMP matches. Peptide sequences with lengths <10 amino acids were discarded. Then, the in silico enzymatic digestion simulation [27] was performed to digest polypeptides into digested peptide sequences. Then, the CD-HIT program [24] was used to remove peptide sequences with >25% identity. Therefore, a total of 37,595 peptides were designated as negative sequence data.
3. Balanced training data were created by proportionate stratified random sampling to select peptide sequences to represent the positive and negative data. The stratified sampling was conducted by similarity clustering of sequence data into homogenous strata based on the CD-HIT clustering tool. The proportional stratified random sampling was performed with the following steps. (i) The sequences were clustered by using CD-HIT with a similarity threshold of 0.3. (ii) Representative sequences were selected from each cluster to use as training data, while the other remaining sequences that were not representative of the cluster will be used as testing data (results are shown in Supplementary File S1 the representative sequences are denoted with the * symbol at the end of the line; the non-representative sequences are displayed with the percentage of sequence similarity to the representative sequence of that cluster). We balanced the number of representative sequences based on the cluster size, as shown in Figure 3. Note that there is no testing sequence presented in the cluster with one sequence member. For some clusters that contain only 1 sequence, the sequence in that cluster will be used as a training set, and the testing sequence is not presented in that cluster. A summary of the sequence similarities between training and testing sequences in all clusters is presented in Supplementary File S2. The sequence similarity between training and testing sequences falls between 30% and 89.47%, with an average of 47.29%. Finally, the training data consists of 1800 peptide sequences of the AMP dataset and 1800 sequences of the non-AMP dataset to make an evenly balanced training dataset in order to reduce the likelihood of generating a predictive model biased toward the majority class.
4. Two testing datasets (testing dataset 1 and testing dataset 2) were created. The first set of testing data was the remaining sequences of both positive data and negative data after training data preparation. Therefore, the first testing dataset consists of 11,634 positive sequence data or AMPs and 35,795 negative sequence data or non-AMPs. The second set of testing data was the benchmark dataset S from published works [13,16]. This dataset can be downloaded from the websites ([28,29]).
5. Sequences having less than 10 amino acids were removed from further analysis. The benchmark dataset S1 contains 1461 AMPs (classified into five functional types: antibacterial peptides, anticancer/tumor peptides, antifungal peptides, anti-HIV peptides, and antiviral peptides) and 2404 non-AMPs, and the dataset S2 contains 917 AMP sequences and 828 non-AMP sequences.

### 2.3. Feature Extraction and Feature Engineering

**Figure 4:**
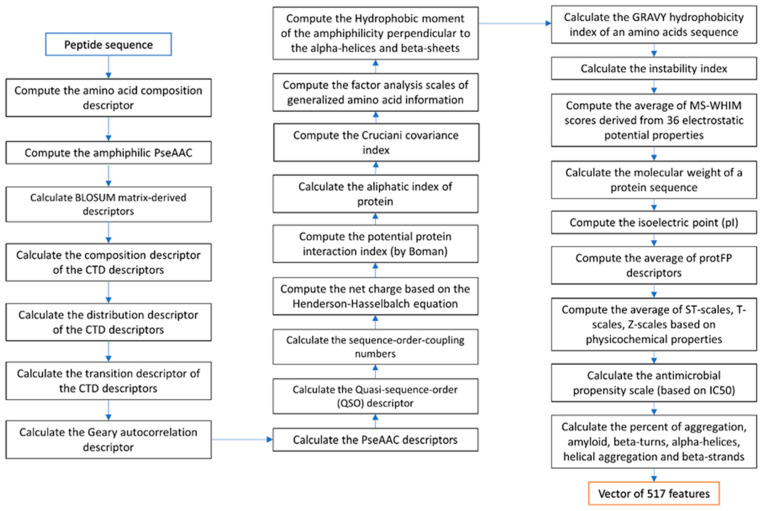
Flowchart of feature extraction.

Various numerical representation schemes of proteins and peptides from amino acid sequences of both AMP and non-AMP datasets were generated. Feature extraction of peptide characteristics that would be useful in predicting, namely, the amino acid composition, pseudo amino acid composition (PseAAC) in parallel and in series correlation, and the details of the secondary structure conformation, composition–transition–distribution (CTD), various physical–chemical properties, antimicrobial propensity scale (antimicrobial IC50 index derived from high-throughput values of different amino acids), and the percentage of different conformations in the peptide sequence were calculated using the protr R package [44,45], peptide R package [45], AMPA program [3,46], Tango program [47], and Pse-in-one program [48,49]. Various modes of Chou’s PseAAC descriptors were generated. Chou’s PseAAC has been widely used to convert complicated protein sequences with various lengths to fixed-length digital feature vectors while keeping considerable sequence-order information. [50]. Chou’s PseAAC can represent a protein sequence in a discrete model without completely losing its sequence-order information and hence has been widely applied for improving the prediction quality for various protein problems [51,52]. The description of features is described in Supplementary Table S3 (in Supplementary File S3). These feature characteristics are stored in the form of a vector of peptide data characteristics that consists of 517 numerical features. The flowchart of feature extraction of peptide data characteristics follows the steps shown in Figure 4.

**Figure 5:**
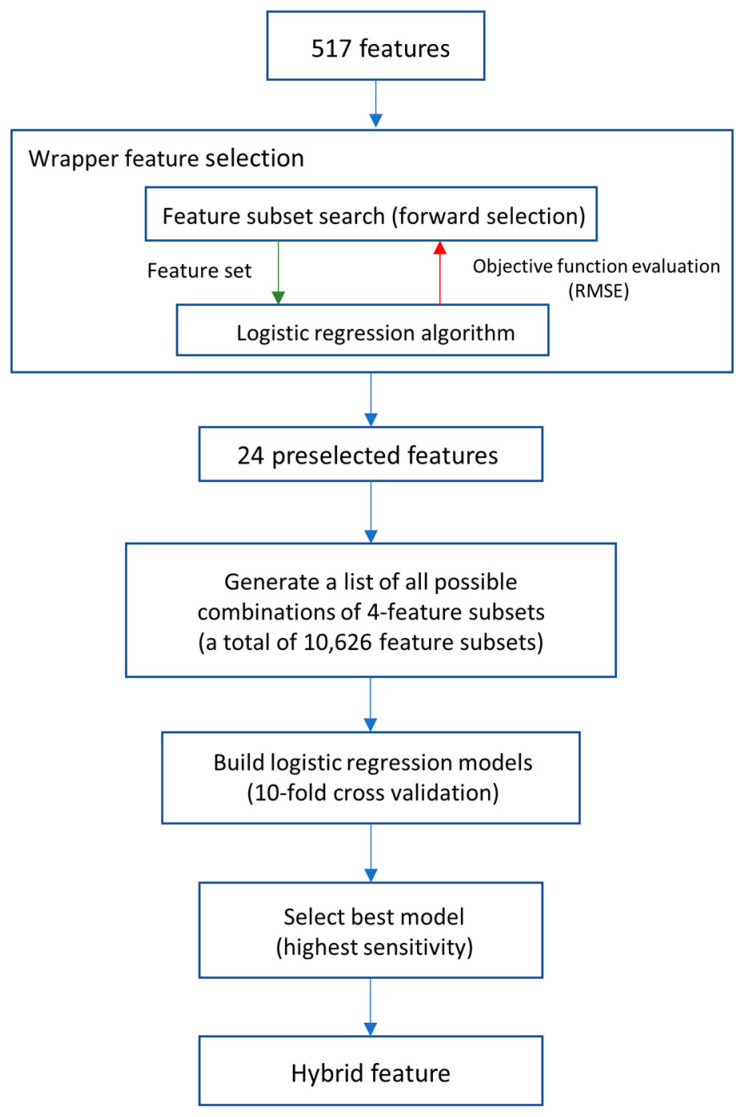
Flowchart of building a hybrid feature.

To improve the prediction with informative features, we proposed a hybrid feature generation by the fusion of various selected features using a logistic regression model. Logistic regression models were built by following the steps shown in Figure 5. Beginning with the preselection of features by using the wrapper feature selection method, feature subsets that specifically suit the logistic regression model were selected. The root-mean-square error (RMSE) and forward search method were performed in the wrapper method, providing 24 features that are most informative for the logistic regression model. To reduce the complexity of hybrid features and avoid the overfitting issues of the models, we used 4 features for creating a composite feature. A combination of 4 randomly selected features out of 24 preselected features generated a total of 10,626 sets of composite features. Next, logistic regression models were built using 10,626 sets of features for a total of 10,626 equations. Then, the performances of the logistic regression models were compared. The logistic regression model with the highest sensitivity would be selected for further study as a hybrid feature. The hybrid feature is defined as the following equation:

Hybrid feature = β_0_ + β_1_ APAAC1_5 + β_2_ CTDD66 + β_3_ AMPA + β_4_ Tango4 (1)

where β_0_ is the intercept, β_1,_ β_2,_ β_3,_ and β_4_ represent the regression coefficients, APAAC1_5 is Amphiphilic Pseudo Amino Acid Composition of Cys (sequence-order-coupling mode along a protein sequence through a hydrophobicity correlation function), CTDD66 is the distribution descriptor of the first residue of the neutral charged amino acid found at the N terminus (Property 5 Group2 Residue 0), AMPA is the antimicrobial IC_50_ propensity index, and Tango4 is the percentage of aggregation conformation.

### 2.4. Feature Selection

To select a discriminative feature subset, the feature selection (FS) method was used to select relevant and informative features that efficiently discriminate AMPs from non-AMPs. Various learning algorithms were used to compare the effectiveness of various feature selections, such as Infogain, ReliefF, and correlation-based feature selection (CFS). We used the area under the ROC curve (AUC) and sensitivity (Sn) values that were averaged over 10-fold cross-validation as measurements of the performance.

### 2.5. Prediction Models

We used a 10-fold cross-validation to examine the machine learning models. The performance of 10 results is averaged and reported as the performance of the classifier. Machine learning techniques, using both single predictive models and ensemble learning methods, were built and compared. The predictive models include neural networks using MLP, SVM, decision tree (DT), KNN, deep learning (DL), naïve Bayes (NB), linear discriminant analysis (LDA), radial basis function network (RBF), RF, max probability voting (MaxProbVote), majority voting, XGBoost, and AdaBoost. These models were built by using LibSVM [53], R programming [45], Waikato Environment for Knowledge Analysis (Weka) [54], and Python. Then, the models were fine-tuned and evaluated based on their performance and CPU processing requirements. The details of the models, hyperparameters, and parameter grid searches are described in the Supplementary File S3

In the algorithm selection for predictive program development, the model was selected based on the total efficiency of the program by evaluating the following metrics:

(2) $$\mathrm{ACC}=\frac{\mathrm{TP}+\mathrm{TN}}{\left(\mathrm{TP}+\mathrm{TN}+\mathrm{FP}+\mathrm{FN}\right)}$$

(3) $$\mathrm{Sn}=\frac{\mathrm{TP}}{\left(\mathrm{TP}+\mathrm{FN}\right)}$$

(4) $$\mathrm{Sp}=\frac{\mathrm{TN}}{\left(\mathrm{TN}+\mathrm{FP}\right)}$$

(5) $$\mathrm{MCC}=\frac{\mathrm{TP}\times \mathrm{TN}-\mathrm{FP}\times \mathrm{FN}}{\sqrt{\left(\mathrm{TP}+\mathrm{FP}\right)\times \left(\mathrm{TP}+\mathrm{FN}\right)\times \left(\mathrm{TN}+\mathrm{FP}\right)\times \left(\mathrm{TN}+\mathrm{FN}\right)}}$$

where ACC, Sn, Sp, and MCC are the accuracy, sensitivity, specificity, and Matthews coefficient correlation, respectively. These measurements were calculated based on the numbers of true positives (TPs), true negatives (TNs), FPs, and false negatives (FNs). The AUC was calculated to assess the tradeoff between the sensitivity and specificity performance of the different methods. The ROC is a plot of the TP rate vs. the FP rate at different thresholds. For a perfect predictor, the AUC is equal to 1.

## 3. Results and Discussion

### 3.1. Informative Features Extracted from Peptides Affect the Performance the Most

To detect hidden patterns, the feature extraction step is an important step to represent biological sequences with a fixed-length numerical form that can be further analyzed using a machine learning model to generalize to new unseen peptide data [51]. The proposed program consists of a module for extracting various features to represent peptides with 517 numerical features. This extraction module collects and extracts as many known peptide features as possible to have sufficient discrimination features in order to detect hidden patterns and to explain the characteristics of the peptide sequences that could be an active AMP.

The correlation between all 517 features was obtained by calculating the Pearson correlation coefficient. Then, Pearson correlation coefficients were plotted as shown in Figure S1. As shown in the correlation plot, there are some redundant and highly correlated features in the 517-dimensional feature set. Therefore, the feature selection step is needed to filter and select only informative and effective features. Feature selection is an important data analysis process to select a more effective feature subset, which can reduce the computation time and complexity, remove redundant features, and improve the understandability and simplicity of the model [55]. Therefore, different feature selection methods were compared. To select the discriminative feature subset, the empirical performance comparison of individual predictive models using different feature sets from various state-of-the-art feature selection methods, such as Infogain, ReliefF, CFS with a best-first search, and CFS with a genetics search, was performed. The CFS with a genetic search method consistently outperforms other feature selection methods in most classifiers while retaining fewer selected features on average. Compared to other feature selection methods, the CFS method can drastically reduce the dimensionality of datasets while maintaining or improving the performance of most learning algorithms (data not shown). The CFS method can remove redundant and irrelevant features based on the heuristic that “Good feature subsets contain features highly correlated with the target class, but uncorrelated with each other” [55]. Finally, based on comparison and the advantages of the CFS method, the 92 informative features (listed in Supplementary Table S2) that were selected by using the CFS method and genetics search were applied for further analysis in this work.

### 3.2. Performance Comparison of Various Predictive Models

Machine learning algorithms tend to be biased toward the majority class when the class distribution is imbalanced in order to yield high overall prediction accuracy. We also investigated the effect of balanced and imbalanced training sets in building models, as reported in Supplementary File S4. We compared the performance of two imbalanced datasets with the ratio of AMPs to non-AMPS equal to 1:3 (natural ratio of this dataset) and 1:2. From the empirical data, most classifiers do not learn the minority class well, often becoming more misclassified compared to the majority class. When the different ratio between classes is high, as the imbalance is more highly skewed, the effect of imbalanced training data will be more severe, as most learners will show more bias toward the majority class. However, in real life, the majority class is usually our class of interest, which is more focused. Therefore, developing well-balanced training data is an important step. There are two categories of methods that handle this class imbalance problem: Data-Level methods (e.g., data sampling), and Algorithm-Level methods (e.g., cost-sensitive and hybrid/ensemble) [56,57,58].

In this work, we have applied both the Data-Level and the Algorithm-Level methods to avoid the imbalance problem. Firstly, since AMP data are heterogeneous (various functional families, from various organisms), in order to ensure that the training sample can reflect all characteristics of the AMP sequences and remain generalized to all types of AMP data, proportionate stratified random sampling (Data-Level method) was applied. Therefore, the training data consisted of peptide sequences selected from both the AMP and non-AMP datasets to produce an equally balanced training dataset to reduce the likelihood of generating a predictive model biased toward the majority class. Then, balanced training data were used to train the predictive models. Secondly, our method is an ensemble or hybrid method (Algorithm-Level method), which is to say that it can also handle the imbalance problem (results in Section 3.3).

In this section, various single models were hyperparameter-optimized using a grid search, and the optimal selected parameters based on model selection are described in the Supplementary File S3: Parameter Optimization. There are many machine learning algorithms, and their performance is dependent on the characteristics of the data of interest. To detect the weaknesses and strengths of various algorithms on AMPs data, tenfold cross-validation was used to compare the performances of various single models, as shown in Table 3, consisting of eight single models, namely, MLP, SVM, KNN, RBF, LDA, NB, DT, and DL, and two existing state-of-the-art programs, namely, AMPScanner [16] (using the latest updated model in February 2020) and CAMP [11] (the results shown in Table 3 report only the highest performance of the four predictive models, RF, neural network (NN), SVM, and LDA).

To evaluate the model, two testing datasets were used. Testing dataset 1 is the dataset, containing the latest and updated AMP and non-AMP sequences, compiled from public databases. It is available to download at [59]. Testing dataset 2 is a benchmark dataset S that has been used in testing several AMP predictive programs taken from [13]. As shown in Table 3. The SVM had the highest performance with an accuracy of 85.89% in the training data. The SVM uses few parameters that have been carefully selected using a grid search. However, the AUC of the SVM model is lower than that of the KNN, MLP, LDA and RBF models. The AUC depicts the tradeoff between the sensitivity and specificity values, which implies achieving a balance in predicting both true positive and true negative instances. Interestingly, the KNN model shows good performance in both accuracy and AUC (85.37% and 0.906, respectively). The advantage of the KNN model is that it is well suited for multimodal classes [60,61]. In fact, AMP-positive data can be considered multimodal class data composed of several types of AMPs, such as antibacterial, anticancer, antiviral, and antifungal AMPs. This may explain the KNN performance on the testing.

In addition, the two existing state-of-the-art programs, namely, AMPScanner (using the latest updated model in February 2020) and CAMP (reporting only the highest performance of the four predictive models, RF, NN, SVM, and LDA), were tested with an independent testing dataset, and their performance is reported in Table 3.

### 3.3. Ensemble Models have Better Performance than Single Models

Since the AMPs have diverse functions with heterogeneous data from various organisms, we hypothesize that the ensemble may be suitable for this type of data. Therefore, we investigated various types of ensemble models. The ensemble model has the ability to increase accuracy by combining the output of multiple diverse classifiers to reduce bias and variance. Moreover, an ensemble can improve efficiency by decomposing complex problems into multiple subproblems. A proper combination of diverse predictors through the ensemble method can efficiently exploit the strength of the single predictive models by considering multiple points of view to obtain a more accurate and robust (stable) prediction. Therefore, in this work, the ensemble models based on bagging, boosting, and voting techniques were also built and compared, as shown in Table 4. The five ensemble models include the RF, MaxProbVote (where the answer from model with a higher probability between the RF and KNN models is chosen), majority vote, XGBoost, and AdaBoost. Comparing the five ensemble models based on accuracy, the MaxProb voting of the RF and KNN has the highest overall accuracy of 87.41% with a high AUC of 0.925 using the training datasets. When comparing the performance based on testing dataset 1, the MaxProbVote model has the highest performance compared to other models in predicting the AMPs of testing dataset 1, but it has a lower performance in identifying AMP-positive sequences than the RF model in testing dataset 2. Table 4 also includes the performances of the five ensemble models integrated with the new hybrid feature (the new hybrid feature will be discussed in Section 3.4). When comparing the 10 ensemble models (with and without the hybrid feature), based on overall accuracy, we found that the majority vote model with the hybrid feature achieved the highest accuracy of 88.63% with an AUC of 0.927. However, MaxProbVote models provide better predictive tradeoffs between sensitivity and specificity than the majority vote model, with a higher AUC value of 0.946. This suggests that a tradeoff between the specificity and sensitivity of the MaxProbVote method is relatively more appropriate.

We found that the performances of ensembles based on bagging and voting methods are comparable (RF, MaxProbVote, and majority voting with and without the hybrid feature). However, for all of the predictive models, both single and ensemble models are included in the Ensemble-AMPPred program (therefore, users can choose and compare the results between these models, especially to use them as a decision support system for a situation in which conflicting predictions occur).

### 3.4. Hybrid Feature to Improve the Sensitivity of the Predictive Performance

To add some additional features for capturing the major and subtle patterns to differentiate actual positives from negatives, we include feature engineering and transformation experiments in this work. We propose a new feature by building a hybrid feature based on feature fusion using a logistic regression equation. The logistic regression model that had the highest sensitivity (Sn = 77.07%) among the others was selected for further use as a hybrid feature. For this new feature, APAAC1_5, CTDD66, AMPA, and Tango4 features were included in this logistic regression and applied as a hybrid feature. The hybrid feature was integrated into the ensemble to test whether this new proposed feature can further improve the sensitivity of the models. We also performed feature ranking based on various filtering feature selection techniques (including information gain, gain ratio, chi-square, consistency, and Pearson’s correlation feature ranking) and found that the newly added hybrid feature can be ranked in the top ranks (in the top 1–5 ranking of most feature selection methods). Moreover, the hybrid feature was the top ranked variable in the plots of variable importance during the process of building both the RF model and the XGBoost model (as shown in Supplementary Figures S2–S3), which indicates the highly significant contribution of the hybrid feature to the prediction performance.

To demonstrate the enhancement when adding the proposed new hybrid feature to the model, Table 4 shows that the overall ACC and AUC values obtained from the ensemble with the hybrid feature are 0.54–2.99% and 0.30–3.92% higher, respectively, than those of the model without the hybrid feature based on 10-fold cross-validation (CV) of the training datasets. Interestingly, we found that the hybrid feature can improve the sensitivity and overall accuracy of the ensemble model based on the bagging and voting methods (RF, MaxProbVote, and majority vote models). We highlight that our hybrid feature is more advantageous for use with bagging or voting ensemble strategies. In particular, including the hybrid feature showed significantly improved performance in the tradeoff between true positive and false positive prediction, as indicated by a better improvement in AUC values.

Actually, the MaxProbVote model with hybrid features (with an ACC of 88.13% and an AUC of 0.946) and the majority vote model with hybrid features (with an ACC of 88.63% and an AUC of 0.927) showed comparable performances. However, the MaxProbVote model with the hybrid feature was chosen as the default model in the Ensemble-AMPPred program because the AUC is higher (AUC is a measure of the value that shows that the model has a better tradeoff between both positive and negative predictions).

### 3.5. Comparison with Existing Prediction Methods

As shown in Table 3, we also reported the testing dataset 1 and 2 prediction results of the other two existing methods: CAMP and AMPScanner. (We initially planned to test more predictive programs; however, since our testing dataset is quite large, other webserver programs become unavailable or nonresponsive.) Based on the testing results using the two testing datasets, our ensemble models (in Table 4) can recognize both AMPs and non-AMPs based on accuracy in predicting positive and negative data compared with the currently available programs, AMPScanner (a DL model using a deep neural network (DNN) and long short-term memory (LSTM) deep learning) and CAMP (LDA, SVM, RF, and NN models), as shown in Table 3. Testing dataset 2, which contains benchmarked data, was not generated by our group. In contrast, testing dataset 1 is quite large, and the latest updated dataset (updated February 2020) was generated by our group. In addition to these two testing datasets, we added another smaller benchmark dataset that included all AMPs from the ADP3 database [62] (obtained in October 2020) and the smaller UniProt dataset as negative data to be compared with another two programs, i.e., iAMP-2 L and iAMPpred. The comparison result is in the Supplementary File S5. The results provide more details beyond the predictive performance summary. The information shows more details about the distribution of incorrect predictions of both false positive and false negative results of all five programs, including our Ensemble_AMPPred (MaxProbVote model), CAMP, AMPscanner, iAMP-2 L, and iAMPpred. This result also gives a better understanding of the different behaviors of different predictive models and identifies influential instances in the testing data, which is the weakness of individual predictive models that makes them perform poorly.

The contributions of this research are as follows: (1) To the best of our knowledge, we collected the largest AMP dataset from 15 public databases, in contrast to previous works that used one to two databases, such as the CAMP and/or APD database. As AMPs are highly heterogeneous, different subtypes of AMPs exist; therefore, we performed proportionate stratified random sampling by partitioning into homogeneous strata based on clustering. Representative sequences were selected from each cluster for using as training data. (2) We collected and employed as many features as possible. Sequence features are important for the antimicrobial activity and in vivo stability of AMPs. Such sequence features also contribute to AMP prediction. Different AMP predictors use different sets of features. For a state-of-the-art deep learning approach, the algorithm automatically extracts features (in the form of a matrix of the weight numbers from deep neural nets). Thus, the deep learning method does not possess a feature engineering step. In our method, feature extraction and selection were conducted. The features are relatively more explainable in terms of biological meanings. We attempted to capture some explainable relationship in the features, which may provide an advantage in AMP sequence design in the future. (3) We include the process of feature engineering, i.e., modeling, to describe or capture relationships between interpretable features to create the hybrid feature. Moreover, we expect that this type of information will be helpful in exploring or designing AMPs sequence in the future. Moreover, we plan to explore this type of feature in greater detail. (4) We investigated various types of machine learning technique for building predictors, including both single and ensemble techniques.

## 4. Conclusions

Ensemble-AMPPred is an AMP prediction and recognition program that contains various predictive models, including individual single models and ensemble models. The overall accuracy obtained by Ensemble-AMPPred is significantly higher than that obtained by the existing methods on the same benchmark dataset. We found that the ensemble model based on the voting technique, especially the MaxProbVote model, has a better tradeoff performance between sensitivity and specificity. Moreover, including the new hybrid feature into the ensemble-based models can improve the accuracy of these predictive models. All the predictive models based on single or ensemble machine learning algorithms are included in Ensemble-AMPPred and are available to download at [59]. Therefore, users can choose between models and can compare and distinguish the results between these models. Moreover, users can use these models as a decision support system for screening new AMPs prior to in vitro laboratory experiments or use them in a situation in which conflicting predictions occur.

## Figures and Tables

**Table 1:** Summary of existing antimicrobial predictions using various machine learning techniques and different features.

Program Name	Techniques	Features	References
AMPer	Random Forests	Profile hidden Markov model (HMM) score	[10]
CAMP-SVM	Support Vector Machine	Sequence composition, physicochemical properties, and structural characteristics of amino acids	[11]
CAMP-RF	Random Forests	Sequence composition, physicochemical properties, and structural characteristics of amino acids	[11]
CAMP-DA	Discriminant Analysis	Sequence composition, physicochemical properties, and structural characteristics of amino acids	[11]
AntiBP	Support Vector Machine	N-terminal and C-terminal residues	[8]
AntiBP2	Support Vector Machine	N-terminal and C-terminal residues	[12]
AMPA	Antimicrobial propensity scale threshold	Antimicrobial index based on IC50 value	[3]
iAMP-2 L	fuzzy K-nearest neighbor	Pseudo amino acid composition (PseAAC) incorporating five physicochemical properties	[13]
DBAASP	Cutoff discriminator	Physicochemical characteristics of peptides: hydrophobic moment, charge density and depth-dependent potential	[14]
MLAMP	ML-SMOTE	PseAAC with the gray model (GM)	[15]
iAMPpred	Support Vector Machine	PseAAC, normalized amino acid compositions, structural features (α-helix, β-sheet and turn structure propensity), isoelectric point, hydrophobicity, and net charge	[9]
AMPscanner	Deep Learning	Numerical matrix from deep neural network (DNN)	[16]

**Table 2:** Public bioactive databases.

Database Name	Reference	Biological Function	Last Updated
The Antimicrobial Peptide Database (APD)	[30]	Antimicrobial	2020
Database Dedicated to Bacteriocin (BACTIBASE)	[31]	Antibacterial	May 2019
Prediction of Bacteriocins In Prokaryotes (BAGEL3)	[32]	Antibacterial	Jan 2019
Collection of Antimicrobial Peptides (CAMP)	[33]	Antimicrobial	Apr 2019
Data repository of antimicrobial peptides (DRAMP)	[34]	Antimicrobial	Sep 2020
Defensins Knowledgebase	[35]	Defensin, antimicrobial	Jun 2019
Endogenous Regulatory OligoPeptide knowledgebase	[36]	Neuropeptide, Antimicrobial	Dec 2019
The Shrimp Antimicrobial Peptide Penaeidin Database (PenBase)	[37]	Antimicrobial	Jul 2008 (Not available now)
A Database Linking Antimicrobial Peptides (LAMP)	[38]	Antimicrobial	Dec 2016
A Database Dedicated to Antimicrobial Plant Peptides (PhytAMP)	[39]	Antimicrobial	Jan 2012
Recombinantly produced Antimicrobial Peptides Database (RAPD)	[40]	Antimicrobial	Mar 2010 (Not available now)
Database of Antimicrobial Activity and Structure of Peptides (DBAASP)	[14]	Antimicrobial	Nov 2017 (Not available now)
BIOPEP-UWM database (BIOPEP)	[41]	Antimicrobial	N/A
A database of anticancer peptides and proteins (CancerPPD)	[42]	Anticancer	N/A
A database of Antiparasitic peptides (ParaPep)	[43]	Antiparasitic	N/A

**Table 3:** Performance comparison of single machine learning models.

Single Model	Training Accuracy	Training AUC	Training MCC	Testing Dataset 1		Testing Dataset 2			
				AMP (11,634)	Non_AMP (35,795)	AMP_S1 (1461)	AMP_S2 (917)	Non_AMP_S1 (2404)	Non_AMP_S2 (828)
**MLP**	81.92%	0.879	0.629	10,224	33,116	1411	907	1613	689
				87.88%	92.51%	96.58%	98.91%	67.07%	83.11%
**SVM**	85.89%	0.867	0.598	10,684	34,283	1437	915	1870	799
				91.83%	95.78%	98.36%	99.78%	77.75%	96.38%
**KNN**	85.37%	0.906	0.701	10,886	33,448	1400	908	1951	815
				93.57%	93.44%	95.82%	99.01%	81.12%	98.31%
**RBF**	83.04%	0.902	0.642	10,161	33,665	1420	912	1668	732
				87.34%	94.05%	97.19%	99.45%	69.36%	88.30%
**LDA**	83.57%	0.901	0.675	9984	33,767	1377	900	1773	762
				85.82%	94.33%	94.25%	98.15%	73.72%	91.92%
**NB**	74.00%	0.786	0.516	7426	34,629	1350	880	1786	782
				63.83%	96.74%	92.40%	95.97%	74.26%	94.33%
**DT**	80.61%	0.797	0.603	10,483	29,020	1374	880	1824	777
				90.11%	81.07%	94.05%	95.97%	75.84%	93.73%
**DL**	85.36%	0.900	0.661	11,063	34,180	1405	900	1967	819
				95.09%	95.49%	96.17%	98.15%	81.79%	98.79%
**CAMP** [11]	-	-	-	8657	20,453	1375	893	1692	615
				74.41%	57.14%	94.11%	97.38%	70.38%	74.28%
**AMPScanner** [16]	-	-	-	10,557	22,511	1420	909	1491	516
				90.74%	62.89%	97.19%	99.13%	62.02%	62.32%

MLP: neural nets using the multilayer perceptron, SVM: support vector machine, KNN: K-nearest neighbors, RBF: radial basis function network, LDA: linear discriminant analysis, NB: naïve Bayes, DT: decision tree, DL: deep learning, ACC: accuracy, AUC: area under the ROC curve, MCC: Matthews correlation coefficient. The numbers in parentheses are the number of instances in the datasets. For the testing dataset, the column presents the number of correctly predicted and the percentage of correctly predicted.

**Table 4:** Performance comparison of ensemble machine learning models.

**Ensemble Model**	**Training Accuracy**	**Training AUC**	**Training MCC**	**Testing Dataset 1**		**Testing Dataset 2**			
				**AMP** **(11,634)**	**Non_AMP** **(35,795)**	**AMP_S1** **(1461)**	**AMP_S2** **(917)**	**Non_AMP_S1** **(2404)**	**Non_AMP_S2** **(828)**
**RF**	86.45%	0.936	0.730	11,115	34,314	1447	915	1895	818
				95.54%	95.86%	99.04%	99.78%	78.79%	98.67%
**MaxProbVote (RF, KNN)**	87.41%	0.925	0.749	11,254	33,992	1441	913	1965	824
				96.73%	94.96%	98.63%	99.56%	81.70%	99.40%
**Majority voting** **(RF, KNN, SVM)**	86.05%	0.892	0.767	11,094	34,598	1441	916	2006	822
				95.36%	96.65%	98.63%	99.89%	83.41%	99.16%
**XGBoost**	85.68%	0.924	0.734	11,156	33,421	1439	910	1920	809
				95.89%	93.37%	98.50%	99.24%	79.88%	97.71%
**AdaBoost**	82.52%	0.910	0.668	9899	33,560	1404	905	1664	711
				85.09%	93.76%	96.09%	98.69%	69.19%	85.77%
**Ensemble model with hybrid feature**	**Training Accuracy**	**Training AUC**	**Training MCC**	**Testing Dataset 1**		**Testing Dataset 2**			
				**AMP** **(11,634)**	**Non_AMP** **(35,795)**	**AMP_S1** **(1461)**	**AMP_S2** **(917)**	**Non_AMP_S1** **(2404)**	**Non_AMP_S2** **(828)**
**RF**	86.92%	0.939	0.744	11,127	34,447	1451	915	1904	822
				95.64%	96.23%	99.32%	99.78%	79.17%	99.15%
**MaxProbVote (RF, KNN)**	88.13%	0.946	0.764	11,344	33,866	1448	915	2042	825
				97.51%	94.61%	99.11%	99.78%	84.94%	99.63%
**Majority voting** **(RF, KNN, SVM)**	88.63%	0.927	0.773	11,295	34,516	1448	915	1993	827
				97.09%	96.43%	99.11%	99.78%	82.87%	99.76%
**XGBoost**	87.44%	0.941	0.749	11,118	33,594	1440	911	1951	818
				95.57%	93.85%	98.56%	99.35%	81.16%	98.79%
**AdaBoost**	83.19%	0.917	0.693	10,866	33,917	1436	909	1681	729
				93.39%	94.75%	98.29%	99.12%	69.89%	87.94%

RF: random forest, XGBoost: extreme gradient boosting, AdaBoost: adaptive boosting, ACC: accuracy, AUC: area under the ROC curve, MCC: Matthews correlation coefficient. The numbers in parentheses are the numbers of instances in the datasets. For the testing dataset, the column presents the number of correctly predicted and the percentage of correctly predicted.

## Data Availability

The data presented in this study are available in the supplementary files.

## Supplementary Materials

The following are available online at https://www.mdpi.com/2073-4425/12/2/137/s1, Supplementary File S1: CD-HIT clustering result of AMP sequences (positive data), Supplementary File S2: Sequence similarities between training and testing sequences, Supplementary File S3: Ensemble AMP Prediction (Figure S1: The correlation between all features obtained by calculating the Pearson correlation coefficient, Figure S2: Variable importance plot. The importance of each of the features for predicting AMPs with a random forest. The most important feature that significantly contributed to the prediction performance was the hybrid feature, Figure S3: Variable importance plot. The importance of each of the features for predicting AMPs with XGBoost. The most important feature that significantly contributed to the prediction performance was the hybrid feature. Table S1: 517 feature descriptors, Table S2: A list of 92 features selected by CFS + Genetic Search), Supplementary File S4: Performance comparison of machine learning models when trained with imbalanced datasets, Supplementary File S5: Preliminary performance comparison of available AMP prediction tools.
