# Supplementary material for: Ensemble-AMPPred: Robust AMP Prediction and Recognition Using the Ensemble Learning Method with a New Hybrid Feature for Differentiating AMPs
Source: Genes (Basel). 2021 Jan 21;12(2):137. doi: 10.3390/genes12020137 (PMC7911732; doi:10.3390/genes12020137)
Supplement: Supplementary file 1 [file genes-12-00137-s001.zip › Ensemble-AMPPred_supplement/Suppelementary File S3_Ensemble_AMPpred.docx]

**Table S1.** 517 feature descriptors.

**Table S2.** A list of 92 features selected by CFS + genetics Search.

| **AAC2** | **CTDD74** | **SOCN7** |
| --- | --- | --- |
| **AAC5** | **CTDD75** | **SOCN8** |
| **AAC13** | **CTDD77** | **charge** |
| **APAAC1_6** | **CTDD78** | **crosscov2** |
| **APAAC1_13** | **CTDD79** | **crucian3** |
| **APAAC1_24** | **CTDD91** | **fasgai2** |
| **APAAC1_27** | **CTDD103** | **fasgai5** |
| **APAAC2_20** | **geary2** | **protFP3** |
| **APAAC2_25** | **geary12** | **protFP5** |
| **APAAC2_29** | **geary18** | **protFP6** |
| **APAAC2_30** | **geary25** | **stscales4** |
| **Blosum3** | **geary27** | **tscales3** |
| **CTDD4** | **geary33** | **vhsescales4** |
| **CTDD7** | **geary37** | **vhsescales8** |
| **CTDD11** | **geary38** | **zscales5** |
| **CTDD12** | **PAAC6** | **ACC4** |
| **CTDD16** | **QSO3** | **ACC10** |
| **CTDD22** | **QSO4** | **ACC11** |
| **CTDD23** | **QSO5** | **ACC14** |
| **CTDD24** | **QSO8** | **ACC15** |
| **CTDD26** | **QSO22** | **Pse_PC2** |
| **CTDD27** | **QSO25** | **Pse_PC3** |
| **CTDD28** | **QSO35** | **Pse_PC17** |
| **CTDD31** | **QSO44** | **Pse_SC2** |
| **CTDD32** | **QSO48** | **Pse_SC3** |
| **CTDD37** | **QSO51** | **Pse_SC6** |
| **CTDD44** | **QSO52** | **Pse_SC26** |
| **CTDD46** | **QSO53** | **AMPA** |
| **CTDD66** | **QSO56** | **tango4** |
| **CTDD68** | **QSO57** | **tango5** |
| **CTDD70** | **QSO58** |  |


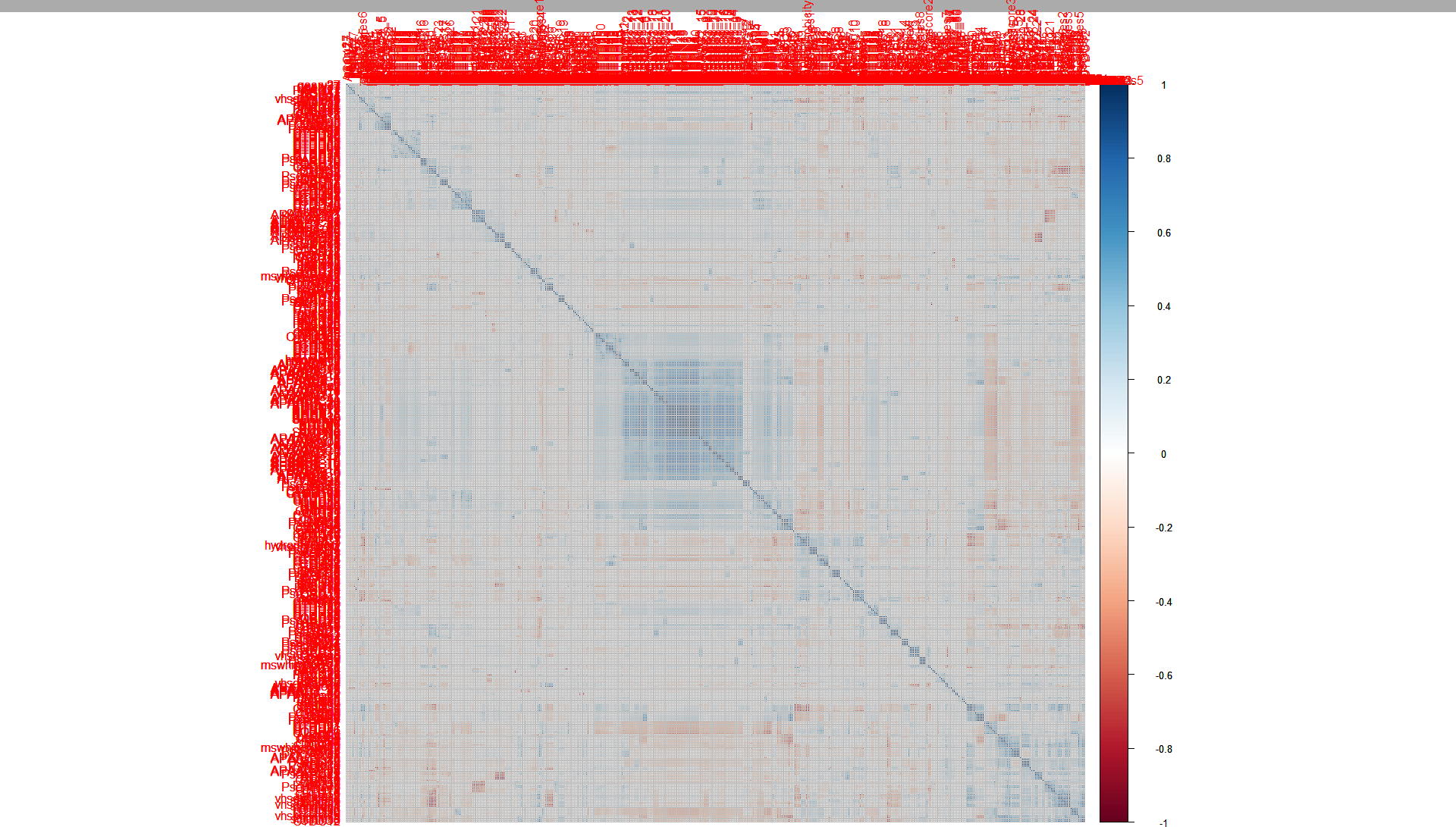


**Figure S1.** The correlation between all features obtained by calculating the Pearson correlation coefficient.


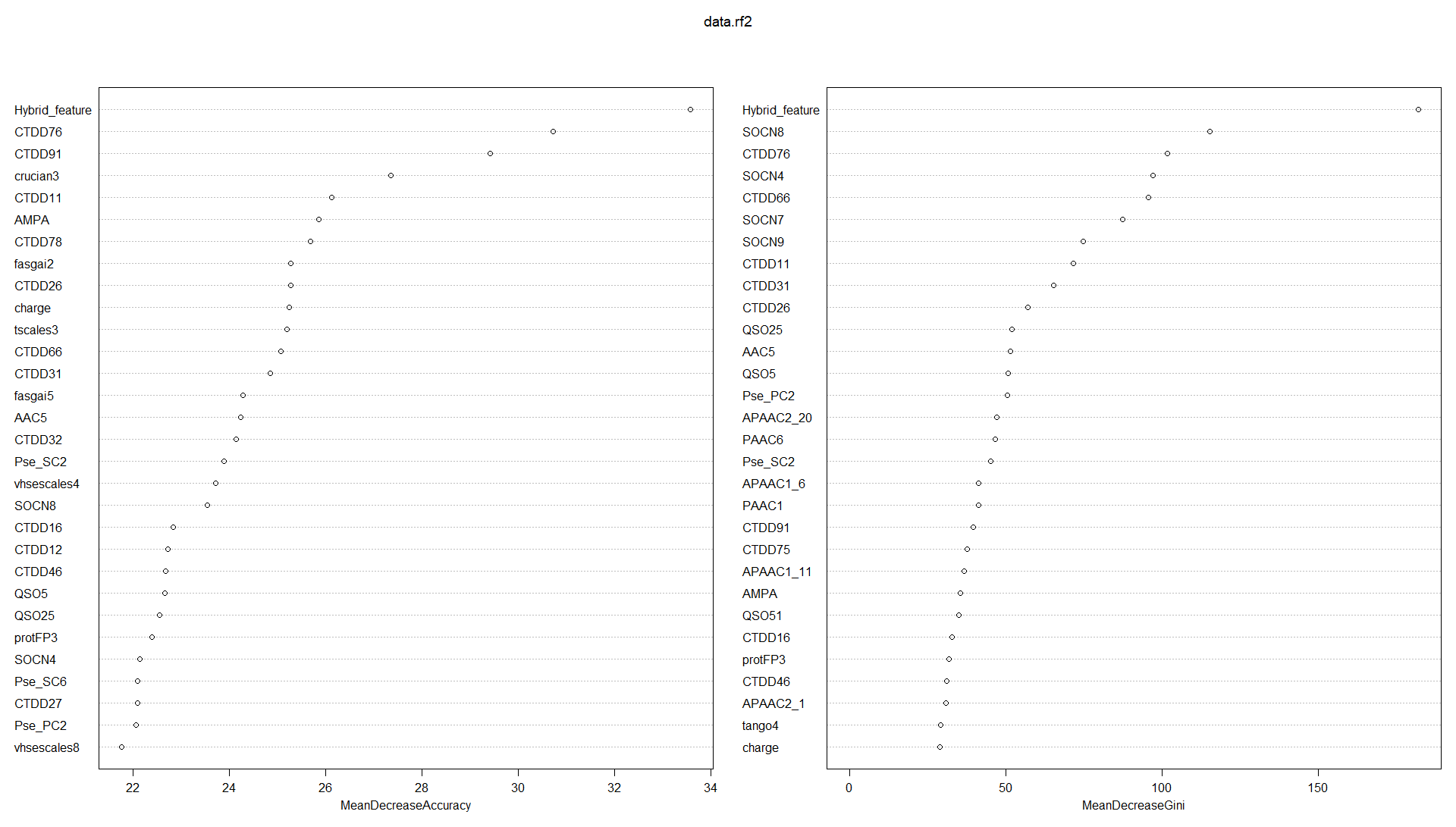


**Figure S2.** Variable Importance plot. The importance of each of the features for predicting AMPs with a random forest. The most important feature as it significantly contributes to the prediction performance was hybrid feature.


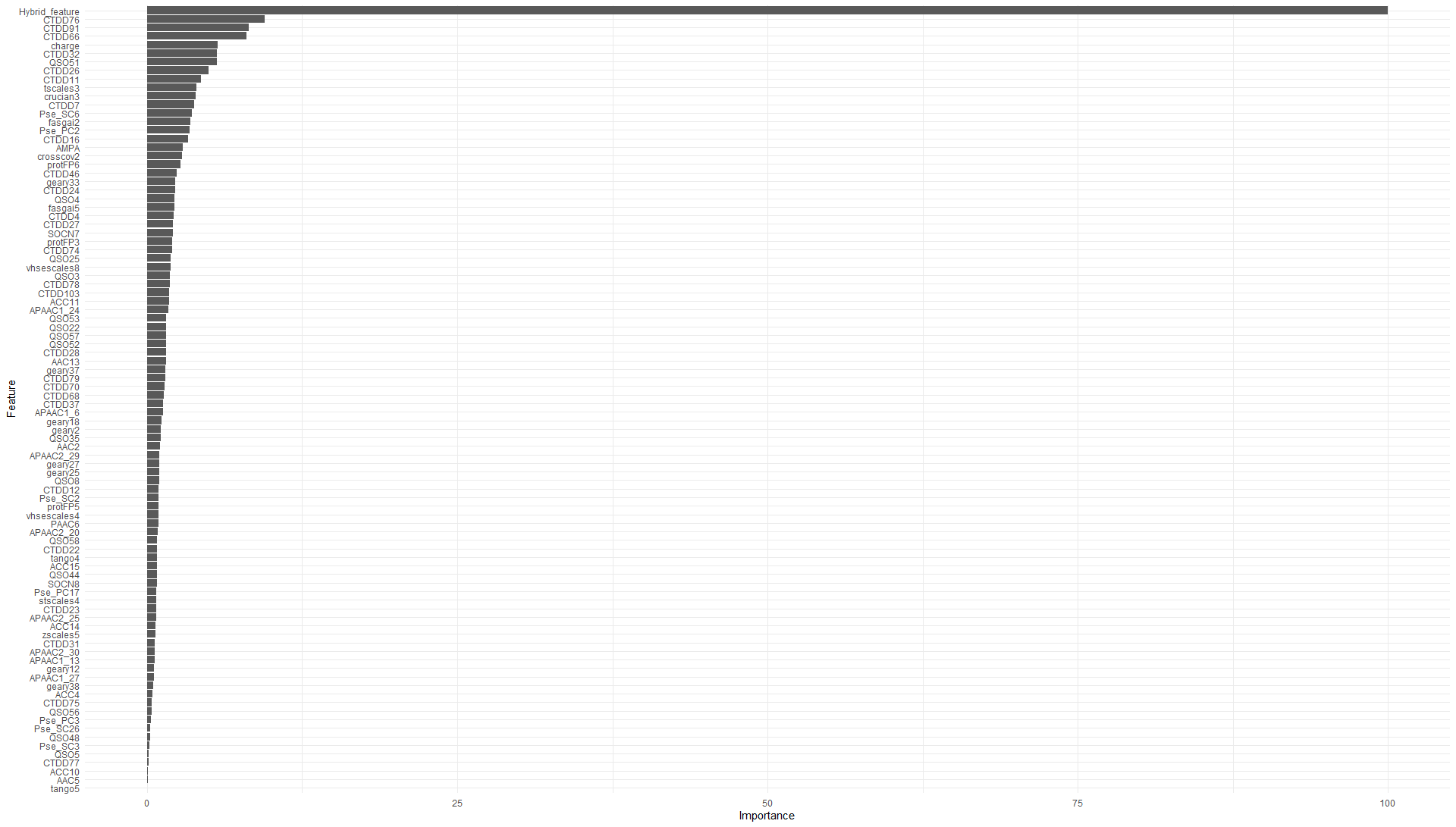


**Figure S3.** Variable Importance plot. The importance of each of the features for predicting AMPs with a XGBoost. The most important feature as it significantly contributes to the prediction performance was hybrid feature.

**Parameter Optimization**

The selected parameters were selected by running different combination of hyperparameters. Parameter setting of each model was optimized and selected by grid search based on 10-fold cross validation.

1. Naïve Bayes (NB): no parameter required.
2. Neural nets using multi-layer perceptron (MLP) with number of epochs: 500; learning rate: 0.1; momentum of updating weight: 0.2.
3. Support Vector Machine (SVM): with a radial basis functions (RBFs) kernel in LIBSVM; grid search was used to find optimal c and gamma values (c= 8; gamma= 0.3125).
4. K-nearest neighbor (KNN) with k=6 and inverse weight yielded the best result. Thus, the KNN with K=6 and inverse weighting was used further.
5. Decision Tree (DT) confidence factor used for pruning was 24% and minimum number of instances per leaf was 2.
6. Gaussian radial basis function network (RBFnets) with number of clusters for K-Means = 2.
7. Deep learning (DL) with 2 dense layers (number of outputs=1,000), 1 output layer
8. Linear Discriminant Analysis (LDA)
9. Random Forest (RF) with the number of variables at the split of the tree = 1 and the number of trees = 1250
10. GB with learning rate = 0.1, number of estimators= 50
11. XGBoost with number of estimators= 50, learning rate =0.1
