# Supplementary material for: Ensemble-AMPPred: Robust AMP Prediction and Recognition Using the Ensemble Learning Method with a New Hybrid Feature for Differentiating AMPs
Source: Genes (Basel). 2021 Jan 21;12(2):137. doi: 10.3390/genes12020137 (PMC7911732; doi:10.3390/genes12020137)
Supplement: Supplementary file 1 [file genes-12-00137-s001.zip › Ensemble-AMPPred_supplement/Supplementary File S1_clustering.pdf]

```
>Cluster 0
0     21aa, >antimicrobial... *
1     11aa, >antimicrobial... at 63.64%
2     17aa, >anti-Gram-;antibact... at 41.18%
3     13aa, >antimicrobial... at 61.54%
4     11aa, >antibacterial;antim... at 54.55%
5     22aa, >antimicrobial... at 40.91%
6     13aa, >antimicrobial... at 46.15%
7     16aa, >antibacterial;antim... at 43.75%
8     19aa, >antibacterial;antim... at 42.11%
9     11aa, >anti-HIV;Antifungal... at 45.45%
10    21aa, >antibacterial... at 42.86%
11    19aa, >antimicrobial... at 42.11%
12    16aa, >Antimicrobial... at 43.75%
13    13aa, >antimicrobial... at 46.15%
14    13aa, >anti-Gram+;antibact... at 46.15%
15    11aa, >anti-Gram+;antibact... at 45.45%
16    13aa, >anti-Gram+;antibact... at 46.15%
17    13aa, >anti-Gram+;antibact... at 46.15%
18    19aa, >antimicrobial... at 42.11%
19    13aa, >anti-Gram+;antimicr... at 46.15%
20    17aa, >antibacterial;Antif... at 41.18%
21    13aa, >antimicrobial... at 46.15%
22    11aa, >antimicrobial... at 33.64%
23    13aa, >antibacterial;antim... at 36.15%
24    13aa, >Antibacterial... at 36.15%
25    13aa, >anti-Gram+;Antimicr... at 36.15%
26    13aa, >Antimicrobial... at 36.15%
27    15aa, >antibacterial;antim... at 46.67%
28    15aa, >antimicrobial... at 35.00%
29    13aa, >anti-Gram+;Antibact... at 36.15%
30    13aa, >anti-Gram+;Antibact... at 36.15%
31    15aa, >antibacterial;antif... at 35.00%
32    13aa, >antimicrobial... at 61.54%
33    11aa, >anti-Gram+;antibact... at 54.55%
34    13aa, >antibacterial;antif... at 46.15%
35    13aa, >anti-Gram+;antibact... at 53.85%
36    13aa, >antibacterial;antif... at 53.85%
37    14aa, >anti-Gram+;Antibact... at 50.00%
38    15aa, >antimicrobial... at 35.00%
39    15aa, >antimicrobial... at 35.00%
40    13aa, >Antibacterial;Antif... at 36.15%
41    13aa, >antibacterial;antim... at 36.15%
42    15aa, >Antibacterial;antim... at 35.00%
43    13aa, >Antibacterial;Gram-... at 36.15%
44    15aa, >antimicrobial... at 46.67%
45    19aa, >antibacterial;antim... at 42.11%
46    13aa, >anti-Gram+;antibact... at 36.15%
47    15aa, >Antimicrobial... at 35.00%
48    13aa, >antibacterial;antif... at 36.15%
49    24aa, >anti-Gram+;antibact... at 41.67%
50    15aa, >antimicrobial... at 35.00%
51    13aa, >Antibacterial;Antif... at 36.15%
52    15aa, >antimicrobial... at 35.00%
```

53 19aa, >Antimicrobial... at 42.11%
54 13aa, >antibacterial;Antif... at 36.15%
55 13aa, >Antimicrobial... at 36.15%
56 11aa, >Antibacterial... at 54.55%
57 12aa, >Antibacterial... at 41.67%
58 20aa, >antimicrobial... at 35.00%
59 11aa, >Antifungal... at 45.45%
60 19aa, >anti-Gram+;Antibact... at 42.11%
61 15aa, >Antimicrobial... at 35.00%
62 26aa, >antimicrobial.... *
63 17aa, >anti-Gram+;Antibact... at 41.18%
64 17aa, >anti-Gram+;Antibact... at 42.94%
65 12aa, >antimicrobial... at 41.67%
66 11aa, >Antiviral... *
67 29aa, >Antimicrobial... at 41.38%
68 12aa, >antimicrobial... at 50.00%
69 13aa, >anti-Gram-... at 53.85%
70 12aa, >Antifungal;Antimicr... at 41.67%
71 12aa, >Antifungal;Antimicr... at 41.67%
72 15aa, >Anti-Gram-... at 35.00%
73 12aa, >antimicrobial... at 50.00%
74 12aa, >Antimicrobial... at 50.00%
75 13aa, >antibacterial... at 53.85%
76 13aa, >antibacterial... at 46.15%
77 12aa, >antimicrobial... at 41.67%
78 11aa, >antimicrobial... at 45.45%
79 12aa, >antibacterial... at 41.67%
80 22aa, >antimicrobial... at 45.45%
81 12aa, >Antifungal;Antimicr... at 50.00%
82 12aa, >Antifungal;Antimicr... at 50.00%
83 12aa, >antimicrobial... at 50.00%
84 24aa, >Antimicrobial... at 41.67%
85 19aa, >anti-Gram+;antibact... at 42.11%
86 12aa, >Anti-Gram-... at 50.00%
87 14aa, >antimicrobial... at 42.86%
88 17aa, >Antimicrobial... at 47.06%
89 17aa, >Antimicrobial... at 41.18%
90 22aa, >Anti-Gram-;Antibact... at 40.91%
91 22aa, >antibacterial;antim... at 40.91%
92 14aa, >Antibacterial;Gram-... at 42.86%
93 12aa, >antimicrobial... at 41.67%
94 14aa, >antimicrobial.... at 42.86%
95 12aa, >antibacterial;Antif... at 50.00%
96 15aa, >antimicrobial... at 35.00%
97 14aa, >Antibacterial... at 57.14%
98 14aa, >anti-Gram+;antibact... at 57.14%
99 14aa, >anti-Gram+;antibact... at 57.14%
100 14aa, >Antibacterial;Antif... at 57.14%
101 14aa, >anti-Gram+;antibact... at 42.86%
102 14aa, >antibacterial;Antif... at 50.00%
103 15aa, >Antibacterial... at 35.00%
104 27aa, >anti-Gram+;antibact... *
105 28aa, >anti-Gram-;antibact... at 42.86%
106 12aa, >antimicrobial... at 50.00%

107   13aa, >Gram-... at 46.15%
108   22aa, >antibacterial;antim... at 40.91%
109   11aa, >antithrombotic... at 45.45%
110   15aa, >Antimicrobial... at 35.00%
111   11aa, >antibacterial;antim... at 45.45%
112   13aa, >antimicrobial... at 46.15%
113   14aa, >antimicrobial... at 50.00%
114   13aa, >antimicrobial... at 46.15%
115   13aa, >antimicrobial... at 46.15%
116   13aa, >Antibacterial;Antif... at 46.15%
117   11aa, >anti-Gram+;Antibact... at 45.45%
118   13aa, >antibacterial... at 46.15%
119   15aa, >antibacterial;Antif... at 35.00%
120   15aa, >antibacterial;Antif... at 35.00%
121   18aa, >Antimicrobial... at 44.44%
122   17aa, >Antimicrobial... at 52.94%
123   17aa, >antibacterial;antim... at 41.18%
124   11aa, >Antimicrobial... at 45.45%
125   11aa, >anti-Gram+;antimicr... at 54.55%
126   16aa, >antimicrobial... at 43.75%
127   836aa, >Antimicrobial... *
128   11aa, >antibacterial;antim... at 54.55%
129   11aa, >antibacterial;antim... at 45.45%
130   20aa, >antimicrobial... at 35.00%
131   12aa, >antithrombotic... at 41.67%
132   12aa, >antibacterial;antim... at 41.67%
133   18aa, >Antifungal... at 44.44%
134   13aa, >anti-Gram+;antibact... at 61.54%
135   16aa, >antimicrobial... at 50.00%
136   14aa, >anti-Gram+;antibact... at 42.86%
137   13aa, >anti-Gram+;antibact... at 46.15%
138   11aa, >antimicrobial... at 45.45%
139   17aa, >Antifungal... at 41.18%
140   13aa, >anti-Gram-... at 46.15%
141   20aa, >Antifungal... at 35.00%
142   15aa, >Antifungal;Antimicr... at 35.00%
143   23aa, >antifungal;antimicr... at 47.83%
144   15aa, >Antifungal;Antimicr... at 35.00%
145   13aa, >antibacterial06509... at 61.54%
146   15aa, >antibacterial06574... at 35.00%
147   20aa, >antibacterial06933... at 35.00%
148   12aa, >antibacterial06934... *
149   12aa, >antibacterial06952... at 41.67%
150   13aa, >antibacterial07014... at 46.15%
151   12aa, >antibacterial07018... at 41.67%
152   12aa, >antibacterial07021... at 50.00%
153   20aa, >antibacterial07037... at 35.00%
154   14aa, >antibacterial07050... at 42.86%
155   13aa, >antibacterial07376... at 46.15%
156   12aa, >antibacterial07385... at 41.67%
157   12aa, >antibacterial07423... at 41.67%
158   11aa, >antibacterial07473... *
159   11aa, >antibacterial07474... at 45.45%
160   20aa, >antibacterial07524... at 35.00%

161 25aa, >antibacterial07531... at 35.00%
162 14aa, >antibacterial07532... at 42.86%
163 20aa, >antibacterial07559... at 35.00%
164 14aa, >antibacterial07659... at 50.00%
165 20aa, >antibacterial07675... at 35.00%
166 14aa, >antibacterial07686... at 42.86%
167 14aa, >antibacterial07689... at 42.86%
168 15aa, >antibacterial07690... at 35.00%
169 11aa, >antibacterial07710... at 45.45%
170 11aa, >antibacterial07738... at 45.45%
171 15aa, >antibacterial07740... at 35.00%
172 11aa, >antibacterial07829... at 54.55%
173 11aa, >antibacterial07830... at 54.55%
174 17aa, >antibacterial07885... at 47.06%
175 13aa, >antibacterial07903... at 46.15%
176 12aa, >antibacterial07915... at 41.67%
177 20aa, >antibacterial07949... at 35.00%
178 12aa, >antibacterial07964... at 41.67%
179 11aa, >antibacterial07978... at 45.45%
180 14aa, >antibacterial07979... at 42.86%
181 14aa, >antibacterial07980... at 42.86%
182 12aa, >antibacterial08034... at 41.67%
183 13aa, >antibacterial08078... at 46.15%
184 13aa, >antibacterial08083... at 46.15%
185 13aa, >antibacterial08086... at 46.15%
186 16aa, >antibacterial08170... at 43.75%
187 13aa, >antibacterial08172... at 46.15%
188 15aa, >antibacterial08196... at 35.00%
189 15aa, >antibacterial08198... at 35.00%
190 11aa, >antibacterial08206... at 54.55%
191 11aa, >antibacterial08211... at 45.45%
192 21aa, >antibacterial08308... at 42.86%
193 13aa, >antibacterial08385... at 46.15%
194 16aa, >antibacterial08415... at 50.00%
195 27aa, >antibacterial08418... at 40.74%
196 18aa, >antibacterial08452... at 50.00%
197 20aa, >antibacterial08479... at 35.00%
198 11aa, >antibacterial08516... at 45.45%
199 12aa, >antibacterial08529... at 41.67%
200 28aa, >antibacterial08572... at 42.86%
201 14aa, >antibacterial08605... at 50.00%
202 14aa, >antibacterial08606... at 50.00%
203 14aa, >antibacterial08607... at 50.00%
204 14aa, >antibacterial08636... at 42.86%
205 15aa, >antibacterial08698... at 35.00%
206 17aa, >antibacterial08711... at 52.94%
207 18aa, >antibacterial08733... at 50.00%
208 14aa, >antibacterial08735... at 57.14%
209 14aa, >antibacterial08737... at 42.86%
210 14aa, >antibacterial08739... at 42.86%
211 14aa, >antibacterial08740... at 50.00%
212 15aa, >antibacterial08741... at 35.00%
213 11aa, >antibacterial08755... at 54.55%
214 12aa, >antibacterial08779... at 50.00%

215 18aa, >antibacterial08780... at 50.00%
216 15aa, >antibacterial08784... at 35.00%
217 12aa, >antibacterial08791... at 41.67%
218 11aa, >antibacterial08822... at 45.45%
219 15aa, >antibacterial08825... at 35.00%
220 12aa, >antibacterial08841... at 41.67%
221 12aa, >antibacterial08842... at 41.67%
222 17aa, >antibacterial08847... at 47.06%
223 13aa, >antibacterial08850... at 46.15%
224 17aa, >antibacterial08857... at 47.06%
225 12aa, >antibacterial08936... at 41.67%
226 12aa, >antibacterial08937... at 41.67%
227 12aa, >antibacterial08938... at 41.67%
228 12aa, >antibacterial08939... at 41.67%
229 12aa, >antibacterial08972... at 41.67%
230 13aa, >antibacterial08975... at 46.15%
231 18aa, >antibacterial09009... at 50.00%
232 12aa, >antibacterial09023... at 41.67%
233 12aa, >antibacterial09024... at 41.67%
234 14aa, >antibacterial09080... at 42.86%
235 14aa, >antibacterial09081... at 42.86%
236 13aa, >antibacterial09130... at 46.15%
237 13aa, >antibacterial09131... at 53.85%
238 13aa, >antibacterial09135... at 46.15%
239 13aa, >antibacterial09136... at 53.85%
240 13aa, >antibacterial09138... at 46.15%
241 13aa, >antibacterial09140... at 46.15%
242 15aa, >antibacterial09279... at 35.00%
243 11aa, >antibacterial09321... at 45.45%
244 15aa, >antibacterial09337... at 35.00%
245 20aa, >antibacterial09382... at 35.00%
246 20aa, >antibacterial09394... at 50.00%
247 20aa, >antibacterial09400... at 45.00%
248 11aa, >antibacterial09402... at 45.45%
249 20aa, >antibacterial09405... at 35.00%
250 20aa, >antibacterial09412... at 35.00%
251 14aa, >antibacterial09413... at 50.00%
252 20aa, >antibacterial09421... at 45.00%
253 20aa, >antibacterial09428... at 35.00%
254 20aa, >antibacterial09434... at 45.00%
255 20aa, >antibacterial09443... at 35.00%
256 20aa, >antibacterial09450... at 35.00%
257 20aa, >antibacterial09451... at 35.00%
258 20aa, >antibacterial09456... at 45.00%
259 22aa, >antibacterial09474... at 40.91%
260 13aa, >antibacterial09539... at 46.15%
261 13aa, >antibacterial09541... *
262 13aa, >antibacterial09569... at 53.85%
263 11aa, >antibacterial09573... at 54.55%
264 14aa, >antibacterial09632... at 42.86%
265 18aa, >antibacterial09673... at 44.44%
266 15aa, >antibacterial09680... at 35.00%
267 15aa, >antibacterial09682... at 35.00%
268 13aa, >antibacterial09725... at 46.15%

```
269   13aa, >antibacterial09729... at 46.15%
270   13aa, >antibacterial09742... at 46.15%
271   13aa, >antibacterial09743... at 53.85%
272   13aa, >antibacterial09813... at 61.54%
273   14aa, >antibacterial09814... at 57.14%
274   11aa, >antibacterial09826... at 54.55%
275   11aa, >antibacterial09828... at 54.55%
276   11aa, >antibacterial09829... at 54.55%
277   11aa, >antibacterial09832... at 54.55%
278   14aa, >antibacterial09910... at 42.86%
279   12aa, >antibacterial09950... at 41.67%
280   12aa, >antibacterial09951... at 41.67%
281   11aa, >antibacterial09981... at 45.45%
282   12aa, >antibacterial09992... at 41.67%
283   15aa, >antibacterial10002... at 35.00%
284   12aa, >antibacterial10030... at 41.67%
285   15aa, >antibacterial10075... at 35.00%
286   14aa, >antibacterial10078... at 42.86%
287   13aa, >antibacterial10104... at 46.15%
288   12aa, >antibacterial10147... at 41.67%
289   17aa, >antibacterial10213... at 41.18%
290   17aa, >antibacterial10214... at 41.18%
291   12aa, >antibacterial10242... at 41.67%
292   12aa, >antibacterial10246... at 41.67%
293   20aa, >antibacterial10259... at 50.00%
294   20aa, >antibacterial10260... at 45.00%
295   11aa, >antibacterial10284... at 45.45%
296   19aa, >antibacterial10311... at 47.37%
297   19aa, >antibacterial10313... at 47.37%
298   19aa, >antibacterial10314... at 42.11%
299   21aa, >antibacterial10328... at 52.38%
300   14aa, >antibacterial10384... at 42.86%
301   14aa, >antibacterial10416... at 50.00%
302   15aa, >antibacterial10438... at 35.00%
303   16aa, >antibacterial10451... at 50.00%
304   14aa, >antibacterial10477... at 42.86%
305   14aa, >antibacterial10479... at 42.86%
306   16aa, >antibacterial10490... at 43.75%
307   15aa, >antibacterial10517... at 35.00%
308   11aa, >antibacterial10561... at 45.45%
309   11aa, >antibacterial10596... at 45.45%
310   15aa, >antibacterial10645... at 35.00%
311   13aa, >antibacterial10690... at 46.15%
312   13aa, >antibacterial10716... at 46.15%
313   18aa, >antibacterial10728... at 44.44%
314   15aa, >antibacterial10729... at 35.00%
315   11aa, >antibacterial10766... at 45.45%
316   14aa, >antibacterial10841... at 42.86%
317   27aa, >antibacterial10854... at 48.15%
318   21aa, >antibacterial10872... at 42.86%
319   20aa, >antibacterial10924... at 35.00%
320   20aa, >antibacterial10956... at 45.00%
321   20aa, >antibacterial10957... at 45.00%
322   20aa, >antibacterial10958... at 35.00%
```

```
323   14aa, >antibacterial10962... at 42.86%
324   14aa, >antibacterial11139... at 42.86%
325   14aa, >antibacterial11140... *
326   14aa, >antibacterial11141... at 50.00%
327   14aa, >antibacterial11142... at 50.00%
328   14aa, >antibacterial11143... at 50.00%
329   12aa, >antibacterial11144... at 41.67%
330   19aa, >antibacterial11156... at 42.11%
331   13aa, >antibacterial11158... at 46.15%
332   14aa, >antibacterial11167... at 42.86%
333   14aa, >antibacterial11168... at 42.86%
334   14aa, >antibacterial11170... at 42.86%
335   18aa, >antibacterial11195... at 44.44%
336   12aa, >antibacterial11198... at 50.00%
337   14aa, >antibacterial11223... at 42.86%
338   20aa, >antibacterial11248... at 35.00%
339   20aa, >antibacterial11263... at 45.00%
340   15aa, >antibacterial11301... at 35.00%
341   20aa, >antibacterial11303... at 35.00%
342   13aa, >antibacterial11313... at 46.15%
343   20aa, >antibacterial11327... at 35.00%
344   35aa, >antibacterial11341... *
345   12aa, >antibacterial11345... at 41.67%
346   15aa, >antibacterial11347... at 35.00%
347   21aa, >antibacterial11349... at 42.86%
348   15aa, >antibacterial11357... at 35.00%
349   12aa, >antibacterial11400... at 50.00%
350   15aa, >antibacterial11422... at 35.00%
351   30aa, >antibacterial11437... at 35.00%
352   15aa, >antibacterial11493... at 46.67%
353   17aa, >antibacterial11525... at 41.18%
354   15aa, >antibacterial11529... at 35.00%
355   15aa, >antibacterial11557... at 35.00%
356   12aa, >antibacterial11568... at 41.67%
357   15aa, >antibacterial11579... at 35.00%
358   11aa, >antibacterial11600... at 54.55%
359   15aa, >antibacterial11604... at 35.00%
360   13aa, >antibacterial11650... at 46.15%
361   20aa, >antibacterial11660... at 50.00%
362   15aa, >antibacterial11665... at 35.00%
363   16aa, >antibacterial11682... at 43.75%
364   19aa, >antibacterial11688... at 42.11%
365   13aa, >antibacterial11690... *
366   14aa, >antibacterial11693... at 42.86%
367   13aa, >antibacterial11695... at 46.15%
368   13aa, >antibacterial11698... at 53.85%
369   13aa, >antibacterial11701... at 46.15%
370   13aa, >antibacterial11702... at 46.15%
371   13aa, >antibacterial11705... at 46.15%
372   15aa, >antibacterial11715... *
373   17aa, >antibacterial11782... at 41.18%
374   27aa, >antibacterial11798... at 44.44%
375   17aa, >antibacterial11833... at 47.06%
376   15aa, >antibacterial11841... at 53.33%
```

377 15aa, >antibacterial11852... at 35.00%
378 12aa, >antibacterial11855... at 50.00%
379 12aa, >antibacterial11857... at 50.00%
380 13aa, >antibacterial11877... at 46.15%
381 15aa, >antibacterial11905... at 35.00%
382 15aa, >antibacterial11909... at 35.00%
383 16aa, >antibacterial11910... at 43.75%
384 20aa, >antibacterial11919... at 50.00%
385 13aa, >antibacterial11923... at 46.15%
386 15aa, >antibacterial11962... at 35.00%
387 14aa, >antibacterial11966... at 50.00%
388 14aa, >antibacterial11968... at 42.86%
389 14aa, >antibacterial11969... at 42.86%
390 12aa, >antibacterial11987... at 41.67%
391 12aa, >antibacterial11988... at 41.67%
392 20aa, >antibacterial12020... at 35.00%
393 14aa, >antibacterial12027... at 57.14%
394 14aa, >antibacterial12072... *
395 15aa, >antibacterial12073... at 35.00%
396 13aa, >antibacterial12074... at 46.15%
397 15aa, >antibacterial12105... at 46.67%
398 17aa, >antibacterial12138... at 41.18%
399 12aa, >antibacterial12141... at 66.67%
400 18aa, >antibacterial12172... at 55.56%
401 13aa, >antibacterial12180... at 46.15%
402 13aa, >antibacterial12235... at 46.15%
403 13aa, >antibacterial12279... at 46.15%
404 18aa, >antibacterial12336... at 44.44%
405 20aa, >antibacterial12378... at 35.00%
406 12aa, >antibacterial12389... at 41.67%
407 17aa, >antibacterial12404... at 41.18%
408 20aa, >antibacterial12452... at 45.00%
409 24aa, >antibacterial12457... at 45.83%
410 12aa, >antibacterial12460... at 41.67%
411 15aa, >antibacterial12478... at 35.00%
412 20aa, >antibacterial12510... at 35.00%
413 15aa, >antibacterial12517... at 35.00%
414 14aa, >antibacterial12527... at 42.86%
415 12aa, >antibacterial12531... at 41.67%
416 12aa, >antibacterial12562... at 41.67%
417 15aa, >antibacterial12597... at 35.00%
418 12aa, >antibacterial12602... at 41.67%
419 15aa, >antibacterial12640... at 35.00%
420 20aa, >antibacterial12670... at 35.00%
421 15aa, >antibacterial12715... at 46.67%
422 20aa, >antibacterial12717... at 55.00%
423 18aa, >antibacterial12723... at 44.44%
424 15aa, >antibacterial12737... at 46.67%
425 15aa, >antibacterial12796... at 35.00%
426 20aa, >antibacterial12797... at 35.00%
427 13aa, >antibacterial12817... at 46.15%
428 20aa, >antibacterial12873... at 35.00%
429 15aa, >antibacterial12874... at 53.33%
430 15aa, >antibacterial12885... at 35.00%

431 15aa, >antibacterial12902... at 35.00%
432 15aa, >antibacterial12963... at 35.00%
433 11aa, >antibacterial12968... at 45.45%
434 11aa, >antibacterial12969... at 45.45%
435 12aa, >antibacterial12970... at 41.67%
436 17aa, >antibacterial12985... at 47.06%
437 13aa, >antibacterial12988... at 46.15%
438 18aa, >antibacterial13006... at 44.44%
439 15aa, >antibacterial13009... at 35.00%
440 12aa, >antibacterial13041... at 41.67%
441 12aa, >antibacterial13055... at 50.00%
442 20aa, >antibacterial13060... at 35.00%
443 12aa, >antibacterial13086... at 41.67%
444 18aa, >antibacterial13089... at 44.44%
445 18aa, >antibacterial13102... at 44.44%
446 12aa, >antibacterial13105... at 41.67%
447 12aa, >antibacterial13110... at 50.00%
448 12aa, >antibacterial13112... at 41.67%
449 12aa, >antibacterial13115... at 50.00%
450 12aa, >antibacterial13117... at 41.67%
451 12aa, >antibacterial13118... at 50.00%
452 20aa, >antibacterial13145... at 35.00%
453 20aa, >antibacterial13158... at 35.00%
454 12aa, >antibacterial13171... at 41.67%
455 17aa, >antibacterial13188... at 52.94%
456 12aa, >antibacterial13212... at 41.67%
457 15aa, >antibacterial13225... at 35.00%
458 18aa, >antibacterial13241... at 44.44%
459 14aa, >antibacterial13250... *
460 14aa, >antibacterial13252... at 42.86%
461 14aa, >antibacterial13254... at 42.86%
462 12aa, >antibacterial13256... at 41.67%
463 12aa, >antibacterial13277... at 50.00%
464 12aa, >antibacterial13285... at 41.67%
465 12aa, >antibacterial13286... at 41.67%
466 20aa, >antibacterial13308... at 35.00%
467 12aa, >antibacterial13354... at 50.00%
468 20aa, >antibacterial13388... at 35.00%
469 14aa, >antibacterial13395... at 42.86%
470 12aa, >antibacterial13405... at 41.67%
471 12aa, >antibacterial13406... at 41.67%
472 15aa, >antibacterial13422... at 46.67%
>Cluster 1
0 26aa, >anti-Gram+;antifung... *
1 17aa, >Antimicrobial... at 41.18%
2 13aa, >anti-Gram-;antibact... at 33.85%
3 15aa, >Antifungal... at 46.67%
4 11aa, >Antibacterial;Antif... at 45.45%
5 11aa, >Antibacterial;Antif... at 45.45%
6 17aa, >antimicrobial... *
7 12aa, >antimicrobial... at 48.33%
8 20aa, >Antifungal... at 35.00%
9 12aa, >antifungal... at 50.00%
10 13aa, >antimicrobial... at 46.15%

11 20aa, >Antibacterial... at 45.00%
12 13aa, >antimicrobial... at 46.15%
13 13aa, >antimicrobial... at 46.15%
14 12aa, >antibacterial;Antif... at 41.67%
15 24aa, >antifungal;antimicr... at 41.67%
16 19aa, >Antimicrobial... at 47.37%
17 22aa, >Antimicrobial... at 45.45%
18 14aa, >antibacterial... at 42.86%
19 12aa, >antimicrobial... at 41.67%
20 17aa, >anti-Gram+;Antibact... at 41.18%
21 17aa, >anti-Gram+;antimicr... at 47.06%
22 27aa, >antimicrobial... at 44.44%
23 11aa, >Antiviral... at 54.55%
24 25aa, >anti-Gram+;antibact... at 35.00%
25 11aa, >Antiviral... at 45.45%
26 14aa, >anti-Gram+;antibact... at 42.86%
27 29aa, >Antimicrobial... at 41.38%
28 28aa, >antifungal;antimicr... at 46.43%
29 14aa, >antibacterial;Antif... at 42.86%
30 21aa, >antimicrobial... at 47.62%
31 20aa, >anti-Gram+;Antibact... at 45.00%
32 12aa, >Antifungal;Antimicr... at 41.67%
33 12aa, >Antifungal;Antimicr... at 41.67%
34 20aa, >anti-Gram+;Antibact... at 50.00%
35 20aa, >antimicrobial... at 35.00%
36 11aa, >antimicrobial... at 54.55%
37 20aa, >Antibacterial... at 35.00%
38 12aa, >antimicrobial... at 41.67%
39 21aa, >antibacterial;antim... at 42.86%
40 22aa, >antimicrobial... at 40.91%
41 12aa, >Antifungal;Antimicr... at 41.67%
42 12aa, >Antifungal;Antimicr... at 41.67%
43 12aa, >Antifungal;antibact... at 41.67%
44 15aa, >Antimicrobial... at 35.00%
45 17aa, >Antifungal;Antibact... at 41.18%
46 14aa, >Antiviral... at 50.00%
47 15aa, >Antimicrobial... at 46.67%
48 12aa, >antimicrobial... at 50.00%
49 14aa, >antibacterial;Antif... at 50.00%
50 14aa, >antimicrobial... at 50.00%
51 11aa, >Antibacterial... at 54.55%
52 14aa, >Antibacterial... at 50.00%
53 16aa, >anti-Gram+;antibact... at 43.75%
54 12aa, >Antibacterial;Gram-... at 50.00%
55 12aa, >anti-Gram+;antibact... at 41.67%
56 13aa, >anti-Gram+;antifung... at 46.15%
57 17aa, >anti-Gram+;Antimicr... at 41.18%
58 14aa, >Antibacterial... at 50.00%
59 17aa, >anti-Gram+;antibact... at 47.06%
60 14aa, >anti-Gram+;antibact... *
61 16aa, >antimicrobial... at 43.75%
62 11aa, >Antibacterial;Gram-... at 54.55%
63 20aa, >Antibacterial... at 35.00%
64 12aa, >antimicrobial... at 41.67%

65 12aa, >antimicrobial... at 50.00%
66 14aa, >Antimicrobial... at 42.86%
67 15aa, >Antibacterial;Antim... at 46.67%
68 15aa, >Antibacterial... at 46.67%
69 13aa, >antimicrobial... at 53.85%
70 16aa, >Antimicrobial... at 43.75%
71 22aa, >antibacterial;antim... at 40.91%
72 14aa, >antibacterial... at 42.86%
73 14aa, >antibacterial... at 42.86%
74 14aa, >antibacterial... at 50.00%
75 20aa, >Antimicrobial... at 35.00%
76 11aa, >antibacterial;antim... at 45.45%
77 12aa, >antibacterial;Antif... at 41.67%
78 19aa, >antibacterial;Antif... at 47.37%
79 626aa, >Antimicrobial... *
80 626aa, >Antimicrobial... at 71.31%
81 49aa, >Antimicrobial... at 40.82%
82 11aa, >antibacterial;antim... at 45.45%
83 13aa, >anti-Gram+;antibact... at 46.15%
84 15aa, >anti-Gram-;antibact... at 35.00%
85 23aa, >antibacterial;Antif... at 43.48%
86 19aa, >anti-Gram+;antibact... at 42.11%
87 18aa, >antimicrobial... at 44.44%
88 15aa, >antifungal... at 46.67%
89 14aa, >antimicrobial... at 42.86%
90 11aa, >Antimicrobial... at 45.45%
91 14aa, >antibacterial... at 42.86%
92 15aa, >Antifungal;Antimicr... at 46.67%
93 11aa, >anti-Gram+... at 45.45%
94 11aa, >anti-Gram+... at 45.45%
95 12aa, >antimicrobial... at 41.67%
96 12aa, >antifungal... at 50.00%
97 12aa, >antifungal... at 41.67%
98 12aa, >antifungal... at 41.67%
99 12aa, >antifungal... at 41.67%
100 12aa, >Antibacterial... at 41.67%
101 12aa, >antibacterial06444... at 41.67%
102 20aa, >antibacterial06524... at 45.00%
103 14aa, >antibacterial06689... at 42.86%
104 16aa, >antibacterial07032... at 43.75%
105 14aa, >antibacterial07049... at 57.14%
106 16aa, >antibacterial07057... at 50.00%
107 20aa, >antibacterial07519... at 35.00%
108 21aa, >antibacterial07534... at 42.86%
109 20aa, >antibacterial07657... at 35.00%
110 12aa, >antibacterial07726... at 41.67%
111 12aa, >antibacterial07828... at 41.67%
112 16aa, >antibacterial07886... at 43.75%
113 15aa, >antibacterial07896... at 35.00%
114 26aa, >antibacterial07935... at 42.31%
115 20aa, >antibacterial07947... at 35.00%
116 15aa, >antibacterial07967... at 35.00%
117 15aa, >antibacterial07974... at 35.00%
118 20aa, >antibacterial08040... at 35.00%

119 17aa, >antibacterial08064... at 41.18%
120 25aa, >antibacterial08069... at 44.00%
121 11aa, >antibacterial08100... *
122 20aa, >antibacterial08252... at 35.00%
123 20aa, >antibacterial08256... at 35.00%
124 11aa, >antibacterial08303... at 45.45%
125 15aa, >antibacterial08439... at 35.00%
126 15aa, >antibacterial08445... at 35.00%
127 15aa, >antibacterial08446... at 35.00%
128 14aa, >antibacterial08466... at 42.86%
129 14aa, >antibacterial08474... at 42.86%
130 12aa, >antibacterial08506... at 41.67%
131 11aa, >antibacterial08515... at 54.55%
132 12aa, >antibacterial08527... at 41.67%
133 12aa, >antibacterial08536... at 41.67%
134 12aa, >antibacterial08587... at 41.67%
135 26aa, >antibacterial08599... at 42.31%
136 17aa, >antibacterial08629... at 41.18%
137 14aa, >antibacterial08643... at 42.86%
138 19aa, >antibacterial08656... at 57.89%
139 14aa, >antibacterial08678... at 42.86%
140 14aa, >antibacterial08707... at 50.00%
141 20aa, >antibacterial08714... at 45.00%
142 13aa, >antibacterial08722... at 53.85%
143 22aa, >antibacterial08726... at 40.91%
144 15aa, >antibacterial08732... at 35.00%
145 15aa, >antibacterial08749... at 60.00%
146 11aa, >antibacterial08763... at 45.45%
147 11aa, >antibacterial08764... at 45.45%
148 11aa, >antibacterial08775... at 45.45%
149 15aa, >antibacterial08776... *
150 11aa, >antibacterial08777... at 45.45%
151 11aa, >antibacterial08778... at 45.45%
152 12aa, >antibacterial08790... at 41.67%
153 15aa, >antibacterial08793... at 35.00%
154 11aa, >antibacterial08797... at 45.45%
155 11aa, >antibacterial08799... at 45.45%
156 11aa, >antibacterial08800... at 45.45%
157 11aa, >antibacterial08801... at 45.45%
158 11aa, >antibacterial08803... at 45.45%
159 11aa, >antibacterial08805... at 45.45%
160 15aa, >antibacterial08809... at 35.00%
161 15aa, >antibacterial08811... at 35.00%
162 15aa, >antibacterial08812... at 46.67%
163 13aa, >antibacterial08906... *
164 20aa, >antibacterial08946... at 35.00%
165 12aa, >antibacterial08963... at 41.67%
166 12aa, >antibacterial08986... at 41.67%
167 12aa, >antibacterial09027... at 41.67%
168 12aa, >antibacterial09029... at 41.67%
169 22aa, >antibacterial09047... at 40.91%
170 15aa, >antibacterial09152... at 46.67%
171 12aa, >antibacterial09153... at 50.00%
172 12aa, >antibacterial09155... at 41.67%

173 14aa, >antibacterial09165... at 50.00%
174 12aa, >antibacterial09242... at 41.67%
175 12aa, >antibacterial09251... at 41.67%
176 12aa, >antibacterial09263... at 58.33%
177 15aa, >antibacterial09276... at 46.67%
178 15aa, >antibacterial09284... at 46.67%
179 26aa, >antibacterial09292... at 46.15%
180 20aa, >antibacterial09351... at 45.00%
181 20aa, >antibacterial09352... at 35.00%
182 20aa, >antibacterial09353... at 45.00%
183 20aa, >antibacterial09354... at 45.00%
184 20aa, >antibacterial09357... at 35.00%
185 20aa, >antibacterial09358... at 35.00%
186 20aa, >antibacterial09359... at 35.00%
187 20aa, >antibacterial09361... at 35.00%
188 20aa, >antibacterial09362... at 35.00%
189 20aa, >antibacterial09365... at 35.00%
190 20aa, >antibacterial09366... at 35.00%
191 20aa, >antibacterial09368... at 35.00%
192 20aa, >antibacterial09370... at 50.00%
193 20aa, >antibacterial09372... at 50.00%
194 20aa, >antibacterial09373... at 35.00%
195 20aa, >antibacterial09374... at 45.00%
196 20aa, >antibacterial09375... at 45.00%
197 20aa, >antibacterial09389... at 35.00%
198 20aa, >antibacterial09441... at 35.00%
199 20aa, >antibacterial09445... at 50.00%
200 13aa, >antibacterial09561... at 53.85%
201 15aa, >antibacterial09604... at 46.67%
202 15aa, >antibacterial09606... at 46.67%
203 15aa, >antibacterial09607... at 46.67%
204 15aa, >antibacterial09608... at 35.00%
205 15aa, >antibacterial09610... at 46.67%
206 15aa, >antibacterial09611... at 35.00%
207 11aa, >antibacterial09624... at 54.55%
208 11aa, >antibacterial09640... at 54.55%
209 11aa, >antibacterial09641... at 45.45%
210 11aa, >antibacterial09642... at 45.45%
211 15aa, >antibacterial09676... at 46.67%
212 25aa, >antibacterial09684... at 35.00%
213 25aa, >antibacterial09687... at 35.00%
214 18aa, >antibacterial09696... at 44.44%
215 18aa, >antibacterial09708... at 44.44%
216 18aa, >antibacterial09720... at 44.44%
217 13aa, >antibacterial09728... at 46.15%
218 11aa, >antibacterial09775... at 45.45%
219 11aa, >antibacterial09776... at 45.45%
220 12aa, >antibacterial09783... at 50.00%
221 11aa, >antibacterial09802... at 45.45%
222 16aa, >antibacterial09804... at 50.00%
223 11aa, >antibacterial09917... at 45.45%
224 12aa, >antibacterial09970... at 41.67%
225 12aa, >antibacterial09972... at 41.67%
226 12aa, >antibacterial09973... at 50.00%

227 15aa, >antibacterial09988... at 35.00%
228 12aa, >antibacterial09998... at 41.67%
229 15aa, >antibacterial10001... at 46.67%
230 12aa, >antibacterial10004... at 41.67%
231 12aa, >antibacterial10024... at 41.67%
232 12aa, >antibacterial10025... at 58.33%
233 12aa, >antibacterial10026... at 50.00%
234 12aa, >antibacterial10027... at 50.00%
235 12aa, >antibacterial10028... at 41.67%
236 12aa, >antibacterial10061... at 41.67%
237 12aa, >antibacterial10062... at 50.00%
238 15aa, >antibacterial10074... at 35.00%
239 13aa, >antibacterial10087... at 46.15%
240 15aa, >antibacterial10148... at 35.00%
241 11aa, >antibacterial10183... at 45.45%
242 13aa, >antibacterial10211... at 46.15%
243 12aa, >antibacterial10231... at 50.00%
244 20aa, >antibacterial10235... at 50.00%
245 22aa, >antibacterial10247... at 40.91%
246 12aa, >antibacterial10318... at 41.67%
247 13aa, >antibacterial10338... at 53.85%
248 15aa, >antibacterial10361... at 46.67%
249 14aa, >antibacterial10376... at 42.86%
250 14aa, >antibacterial10415... at 50.00%
251 14aa, >antibacterial10437... at 42.86%
252 15aa, >antibacterial10467... at 46.67%
253 12aa, >antibacterial10511... at 50.00%
254 19aa, >antibacterial10514... at 42.11%
255 12aa, >antibacterial10536... at 41.67%
256 14aa, >antibacterial10557... at 42.86%
257 14aa, >antibacterial10558... at 42.86%
258 11aa, >antibacterial10600... at 45.45%
259 22aa, >antibacterial10671... at 45.45%
260 13aa, >antibacterial10685... at 46.15%
261 13aa, >antibacterial10688... at 46.15%
262 20aa, >antibacterial10722... at 35.00%
263 20aa, >antibacterial10768... at 45.00%
264 20aa, >antibacterial10769... at 45.00%
265 17aa, >antibacterial10937... at 41.18%
266 20aa, >antibacterial10959... at 45.00%
267 14aa, >antibacterial11109... at 64.29%
268 13aa, >antibacterial11127... at 53.85%
269 14aa, >antibacterial11145... at 50.00%
270 14aa, >antibacterial11146... at 42.86%
271 16aa, >antibacterial11151... at 43.75%
272 15aa, >antibacterial11164... at 46.67%
273 14aa, >antibacterial11169... at 50.00%
274 12aa, >antibacterial11193... at 41.67%
275 11aa, >antibacterial11197... at 54.55%
276 11aa, >antibacterial11204... at 54.55%
277 12aa, >antibacterial11218... at 41.67%
278 12aa, >antibacterial11224... at 41.67%
279 14aa, >antibacterial11229... at 42.86%
280 14aa, >antibacterial11232... at 50.00%

281 14aa, >antibacterial11239... *
282 14aa, >antibacterial11240... at 50.00%
283 13aa, >antibacterial11260... at 46.15%
284 20aa, >antibacterial11286... at 35.00%
285 20aa, >antibacterial11287... at 35.00%
286 15aa, >antibacterial11288... at 35.00%
287 21aa, >antibacterial11348... at 47.62%
288 21aa, >antibacterial11350... at 42.86%
289 15aa, >antibacterial11502... at 46.67%
290 16aa, >antibacterial11516... at 50.00%
291 15aa, >antibacterial11517... at 46.67%
292 17aa, >antibacterial11533... at 41.18%
293 15aa, >antibacterial11534... at 35.00%
294 15aa, >antibacterial11581... at 46.67%
295 18aa, >antibacterial11611... at 44.44%
296 14aa, >antibacterial11624... at 42.86%
297 12aa, >antibacterial11658... at 41.67%
298 13aa, >antibacterial11672... at 46.15%
299 13aa, >antibacterial11709... at 53.85%
300 15aa, >antibacterial11710... at 35.00%
301 15aa, >antibacterial11783... at 46.67%
302 11aa, >antibacterial11847... at 45.45%
303 15aa, >antibacterial11868... at 35.00%
304 16aa, >antibacterial11879... at 43.75%
305 18aa, >antibacterial11903... at 44.44%
306 15aa, >antibacterial11915... at 35.00%
307 15aa, >antibacterial11932... at 35.00%
308 13aa, >antibacterial11942... at 46.15%
309 14aa, >antibacterial11970... at 50.00%
310 12aa, >antibacterial11983... at 41.67%
311 15aa, >antibacterial12039... at 35.00%
312 15aa, >antibacterial12045... at 35.00%
313 14aa, >antibacterial12075... at 42.86%
314 14aa, >antibacterial12078... at 57.14%
315 14aa, >antibacterial12084... at 57.14%
316 14aa, >antibacterial12089... at 50.00%
317 17aa, >antibacterial12123... at 41.18%
318 19aa, >antibacterial12133... at 47.37%
319 20aa, >antibacterial12142... at 35.00%
320 13aa, >antibacterial12148... at 46.15%
321 21aa, >antibacterial12167... at 42.86%
322 25aa, >antibacterial12182... at 35.00%
323 17aa, >antibacterial12186... at 47.06%
324 13aa, >antibacterial12239... at 46.15%
325 13aa, >antibacterial12241... at 46.15%
326 13aa, >antibacterial12242... at 46.15%
327 13aa, >antibacterial12245... at 46.15%
328 12aa, >antibacterial12366... at 41.67%
329 11aa, >antibacterial12400... at 45.45%
330 15aa, >antibacterial12420... at 35.00%
331 15aa, >antibacterial12421... at 35.00%
332 15aa, >antibacterial12428... at 46.67%
333 15aa, >antibacterial12429... at 46.67%
334 15aa, >antibacterial12456... at 35.00%

```
335   12aa, >antibacterial12459... at 41.67%
336   20aa, >antibacterial12493... at 50.00%
337   15aa, >antibacterial12511... at 35.00%
338   16aa, >antibacterial12543... at 43.75%
339   12aa, >antibacterial12565... at 41.67%
340   12aa, >antibacterial12568... at 50.00%
341   12aa, >antibacterial12571... at 41.67%
342   15aa, >antibacterial12580... at 35.00%
343   15aa, >antibacterial12582... at 46.67%
344   15aa, >antibacterial12601... at 35.00%
345   11aa, >antibacterial12648... at 45.45%
346   12aa, >antibacterial12649... at 50.00%
347   12aa, >antibacterial12693... at 50.00%
348   15aa, >antibacterial12709... at 35.00%
349   15aa, >antibacterial12722... at 35.00%
350   15aa, >antibacterial12790... at 35.00%
351   15aa, >antibacterial12794... at 46.67%
352   15aa, >antibacterial12803... at 53.33%
353   18aa, >antibacterial12822... at 44.44%
354   20aa, >antibacterial12940... at 50.00%
355   20aa, >antibacterial12956... at 55.00%
356   20aa, >antibacterial12961... at 50.00%
357   12aa, >antibacterial12966... at 41.67%
358   15aa, >antibacterial13077... at 35.00%
359   29aa, >antibacterial13106... at 41.38%
360   12aa, >antibacterial13113... at 41.67%
361   12aa, >antibacterial13120... at 41.67%
362   12aa, >antibacterial13154... at 50.00%
363   13aa, >antibacterial13203... at 53.85%
364   12aa, >antibacterial13206... at 41.67%
365   15aa, >antibacterial13215... at 35.00%
366   14aa, >antibacterial13255... at 42.86%
367   15aa, >antibacterial13261... at 35.00%
368   11aa, >antibacterial13272... at 45.45%
369   11aa, >antibacterial13316... at 54.55%
370   13aa, >antibacterial13343... at 46.15%
371   11aa, >antibacterial13346... *
372   11aa, >antibacterial13353... at 54.55%
373   12aa, >antibacterial13358... at 41.67%
374   22aa, >antibacterial13416... at 50.00%
>Cluster 2
0     20aa, >anti-Gram+;antibact... at 35.00%
1     12aa, >Antibacterial... at 41.67%
2     12aa, >antibacterial... at 50.00%
3     18aa, >Antibacterial;Antif... at 50.00%
4     17aa, >Antifungal... at 47.06%
5     11aa, >anti-Gram+;antibact... at 54.55%
6     29aa, >antibacterial;antim... at 41.38%
7     19aa, >Antibacterial... at 42.11%
8     20aa, >antibacterial;antif... at 35.00%
9     11aa, >anti-Gram+;Antimicr... at 45.45%
10    11aa, >anti-Gram+;Antimicr... at 45.45%
11    15aa, >Antifungal... at 35.00%
12    11aa, >Antibacterial... at 45.45%
```

13 15aa, >anti-Gram+;Antibact... *
14 14aa, >Antiviral... at 42.86%
15 15aa, >antimicrobial... at 35.00%
16 20aa, >antimicrobial... at 35.00%
17 15aa, >antimicrobial... at 35.00%
18 14aa, >antimicrobial... at 42.86%
19 20aa, >antimicrobial... at 45.00%
20 24aa, >anti-Gram+;antibact... at 41.67%
21 13aa, >antibacterial;Antif... at 46.15%
22 24aa, >anti-Gram+;antibact... at 41.67%
23 16aa, >anti-Gram+;antibact... at 43.75%
24 13aa, >anti-Gram+;antibact... *
25 13aa, >antibacterial;Antif... at 53.85%
26 23aa, >antibacterial;antim... at 43.48%
27 14aa, >Antimicrobial... at 42.86%
28 12aa, >anti-Gram+;antibact... at 41.67%
29 17aa, >antimicrobial... at 47.06%
30 24aa, >antibacterial;Antif... at 41.67%
31 20aa, >antimicrobial... at 35.00%
32 33aa, >anti-Gram+;antibact... at 42.42%
33 24aa, >anti-Gram+;antibact... at 41.67%
34 11aa, >anti-Gram-;antimicr... at 45.45%
35 19aa, >Anti-Gram-... at 47.37%
36 25aa, >Antimicrobial... at 35.00%
37 14aa, >anti-Gram+;antibact... at 42.86%
38 32aa, >antibacterial;Antif... at 40.62%
39 12aa, >Antifungal;Antimicr... at 41.67%
40 17aa, >Antibacterial;antim... at 52.94%
41 17aa, >anti-Gram+;Antibact... at 41.18%
42 24aa, >antibacterial;antim... at 45.83%
43 19aa, >anti-Gram-;Antibact... at 42.11%
44 27aa, >antimicrobial... at 40.74%
45 16aa, >antibacterial... at 50.00%
46 11aa, >Antibacterial;antim... at 54.55%
47 12aa, >Antibacterial;Antif... at 41.67%
48 28aa, >antibacterial;antim... at 42.86%
49 22aa, >antimicrobial... at 40.91%
50 22aa, >Antibacterial;antim... at 54.55%
51 22aa, >anti-Gram-;Antibact... at 50.00%
52 11aa, >membrane... at 45.45%
53 19aa, >anti-Gram+;Antibact... at 42.11%
54 15aa, >Antimicrobial... at 35.00%
55 15aa, >anti-Gram+;antibact... at 35.00%
56 15aa, >anti-Gram+;antibact... at 46.67%
57 12aa, >antimicrobial... at 41.67%
58 13aa, >antibacterial;antim... at 46.15%
59 20aa, >anti-Gram+;Antibact... at 60.00%
60 13aa, >anti-Gram+;antibact... at 53.85%
61 14aa, >anti-Gram+;antibact... at 57.14%
62 15aa, >anti-Gram+;Antibact... at 35.00%
63 21aa, >Antimicrobial;Gram-... at 47.62%
64 11aa, >antibacterial;antim... at 45.45%
65 13aa, >antibacterial;antim... at 46.15%
66 12aa, >Anti-Gram-... at 58.33%

67 16aa, >antimicrobial... at 50.00%
68 11aa, >Antimicrobial... at 45.45%
69 12aa, >antifungal... at 41.67%
70 12aa, >antifungal... at 41.67%
71 12aa, >antifungal... at 41.67%
72 11aa, >anti-Gram+;Antimicr... at 45.45%
73 17aa, >Antibacterial... at 47.06%
74 11aa, >antibacterial... at 45.45%
75 12aa, >antibacterial... at 41.67%
76 15aa, >Antibacterial... at 46.67%
77 15aa, >Antifungal;Antimicr... at 35.00%
78 15aa, >Antifungal;Antimicr... at 46.67%
79 15aa, >Antibacterial... *
80 20aa, >Gram-... at 35.00%
81 12aa, >antifungal... at 41.67%
82 19aa, >antibacterial;Antif... at 42.11%
83 17aa, >anti-Gram+;antibact... at 41.18%
84 13aa, >antimicrobial... at 53.85%
85 11aa, >antimicrobial... at 72.73%
86 14aa, >antibacterial... at 57.14%
87 618aa, >Antimicrobial... *
88 13aa, >antibacterial;antim... at 46.15%
89 14aa, >antimicrobial... at 42.86%
90 11aa, >antibacterial... at 45.45%
91 18aa, >Antimicrobial... at 50.00%
92 15aa, >Antifungal... at 46.67%
93 25aa, >Antifungal... at 35.00%
94 19aa, >anti-Gram-... at 42.11%
95 205aa, >Antimicrobial... at 40.49%
96 30aa, >antibacterial;antim... at 35.00%
97 16aa, >antimicrobial... at 43.75%
98 15aa, >Antifungal... at 46.67%
99 15aa, >anti-Gram-;antibact... at 35.00%
100 14aa, >antimicrobial... at 42.86%
101 14aa, >anti-Gram-;antifung... at 42.86%
102 11aa, >antimicrobial... at 63.64%
103 14aa, >antimicrobial... at 50.00%
104 15aa, >Antifungal;Antimicr... at 35.00%
105 15aa, >anti-Gram+;Gram-... at 35.00%
106 12aa, >antibacterial... at 41.67%
107 13aa, >antibacterial06462... at 46.15%
108 23aa, >antibacterial06552... at 43.48%
109 13aa, >antibacterial06561... at 46.15%
110 20aa, >antibacterial06562... at 35.00%
111 28aa, >antibacterial06626... at 42.86%
112 25aa, >antibacterial06641... at 52.00%
113 14aa, >antibacterial07042... at 42.86%
114 13aa, >antibacterial07388... at 53.85%
115 17aa, >antibacterial07444... at 41.18%
116 11aa, >antibacterial07504... at 45.45%
117 25aa, >antibacterial07560... at 35.00%
118 25aa, >antibacterial07561... at 35.00%
119 12aa, >antibacterial07613... at 41.67%
120 20aa, >antibacterial07626... at 35.00%

```
121   20aa, >antibacterial07627... at 35.00%
122   20aa, >antibacterial07628... at 35.00%
123   20aa, >antibacterial07629... at 35.00%
124   20aa, >antibacterial07630... at 35.00%
125   20aa, >antibacterial07636... at 35.00%
126   20aa, >antibacterial07637... at 35.00%
127   20aa, >antibacterial07638... at 35.00%
128   20aa, >antibacterial07674... at 35.00%
129   14aa, >antibacterial07678... at 50.00%
130   17aa, >antibacterial07699... at 47.06%
131   24aa, >antibacterial07704... at 41.67%
132   11aa, >antibacterial07706... at 45.45%
133   14aa, >antibacterial07707... at 42.86%
134   12aa, >antibacterial07708... at 41.67%
135   11aa, >antibacterial07729... at 45.45%
136   11aa, >antibacterial07734... at 45.45%
137   14aa, >antibacterial07766... at 50.00%
138   14aa, >antibacterial07767... at 50.00%
139   26aa, >antibacterial07781... at 42.31%
140   26aa, >antibacterial07782... at 42.31%
141   26aa, >antibacterial07786... at 42.31%
142   14aa, >antibacterial07821... at 42.86%
143   14aa, >antibacterial07842... at 42.86%
144   14aa, >antibacterial07843... at 50.00%
145   13aa, >antibacterial07926... at 46.15%
146   16aa, >antibacterial07936... at 43.75%
147   18aa, >antibacterial07963... at 50.00%
148   18aa, >antibacterial07968... at 50.00%
149   12aa, >antibacterial07998... at 41.67%
150   12aa, >antibacterial07999... at 50.00%
151   12aa, >antibacterial08000... at 50.00%
152   20aa, >antibacterial08042... at 35.00%
153   23aa, >antibacterial08096... at 43.48%
154   12aa, >antibacterial08115... at 41.67%
155   13aa, >antibacterial08151... at 46.15%
156   13aa, >antibacterial08164... at 53.85%
157   13aa, >antibacterial08167... at 46.15%
158   13aa, >antibacterial08169... at 46.15%
159   11aa, >antibacterial08230... at 45.45%
160   13aa, >antibacterial08348... at 46.15%
161   17aa, >antibacterial08378... at 41.18%
162   13aa, >antibacterial08417... at 46.15%
163   14aa, >antibacterial08450... at 42.86%
164   14aa, >antibacterial08469... at 42.86%
165   14aa, >antibacterial08471... *
166   20aa, >antibacterial08484... at 35.00%
167   12aa, >antibacterial08486... at 41.67%
168   19aa, >antibacterial08541... at 47.37%
169   14aa, >antibacterial08550... at 42.86%
170   12aa, >antibacterial08589... at 41.67%
171   15aa, >antibacterial08601... at 35.00%
172   14aa, >antibacterial08603... at 50.00%
173   14aa, >antibacterial08612... at 50.00%
174   15aa, >antibacterial08647... at 35.00%
```

175 15aa, >antibacterial08649... at 46.67%
176 15aa, >antibacterial08650... at 53.33%
177 19aa, >antibacterial08653... at 42.11%
178 19aa, >antibacterial08664... at 42.11%
179 19aa, >antibacterial08667... at 47.37%
180 15aa, >antibacterial08691... at 46.67%
181 15aa, >antibacterial08693... at 35.00%
182 15aa, >antibacterial08694... at 53.33%
183 15aa, >antibacterial08695... at 35.00%
184 15aa, >antibacterial08700... at 35.00%
185 15aa, >antibacterial08701... at 35.00%
186 13aa, >antibacterial08704... at 69.23%
187 20aa, >antibacterial08715... at 45.00%
188 14aa, >antibacterial08734... at 42.86%
189 15aa, >antibacterial08744... at 35.00%
190 11aa, >antibacterial08750... at 45.45%
191 15aa, >antibacterial08813... at 35.00%
192 15aa, >antibacterial08814... at 35.00%
193 15aa, >antibacterial08815... at 35.00%
194 15aa, >antibacterial08820... at 35.00%
195 13aa, >antibacterial08834... at 46.15%
196 19aa, >antibacterial08872... at 42.11%
197 19aa, >antibacterial08875... *
198 13aa, >antibacterial08902... at 46.15%
199 21aa, >antibacterial08911... at 52.38%
200 18aa, >antibacterial08913... at 44.44%
201 18aa, >antibacterial08941... at 44.44%
202 18aa, >antibacterial08944... at 44.44%
203 12aa, >antibacterial08950... at 41.67%
204 12aa, >antibacterial08953... at 41.67%
205 12aa, >antibacterial08959... at 41.67%
206 12aa, >antibacterial08960... at 50.00%
207 12aa, >antibacterial08968... at 41.67%
208 23aa, >antibacterial08974... at 43.48%
209 19aa, >antibacterial08977... at 42.11%
210 20aa, >antibacterial08979... at 45.00%
211 14aa, >antibacterial08996... at 42.86%
212 12aa, >antibacterial09031... at 41.67%
213 12aa, >antibacterial09051... at 50.00%
214 15aa, >antibacterial09063... at 35.00%
215 22aa, >antibacterial09064... at 40.91%
216 14aa, >antibacterial09074... at 42.86%
217 14aa, >antibacterial09077... at 42.86%
218 18aa, >antibacterial09160... at 44.44%
219 18aa, >antibacterial09161... at 44.44%
220 15aa, >antibacterial09277... at 35.00%
221 19aa, >antibacterial09307... at 52.63%
222 11aa, >antibacterial09313... at 63.64%
223 18aa, >antibacterial09340... at 50.00%
224 12aa, >antibacterial09341... at 41.67%
225 11aa, >antibacterial09363... at 45.45%
226 20aa, >antibacterial09380... at 45.00%
227 11aa, >antibacterial09381... at 54.55%
228 14aa, >antibacterial09424... at 42.86%

```
229   14aa, >antibacterial09446... at 42.86%
230   20aa, >antibacterial09463... at 35.00%
231   20aa, >antibacterial09489... at 35.00%
232   17aa, >antibacterial09522... at 41.18%
233   25aa, >antibacterial09562... at 48.00%
234   25aa, >antibacterial09563... at 48.00%
235   25aa, >antibacterial09564... at 48.00%
236   25aa, >antibacterial09565... at 48.00%
237   25aa, >antibacterial09566... at 48.00%
238   25aa, >antibacterial09567... at 48.00%
239   25aa, >antibacterial09568... at 48.00%
240   25aa, >antibacterial09570... at 52.00%
241   25aa, >antibacterial09571... at 48.00%
242   25aa, >antibacterial09572... at 48.00%
243   25aa, >antibacterial09578... at 48.00%
244   13aa, >antibacterial09591... at 46.15%
245   11aa, >antibacterial09627... at 45.45%
246   13aa, >antibacterial09727... at 69.23%
247   13aa, >antibacterial09731... at 61.54%
248   15aa, >antibacterial09735... at 46.67%
249   15aa, >antibacterial09767... at 35.00%
250   12aa, >antibacterial09782... at 41.67%
251   12aa, >antibacterial09791... at 41.67%
252   13aa, >antibacterial09857... at 46.15%
253   19aa, >antibacterial09906... at 47.37%
254   11aa, >antibacterial09911... at 54.55%
255   20aa, >antibacterial09930... at 45.00%
256   12aa, >antibacterial09971... at 41.67%
257   12aa, >antibacterial09993... at 50.00%
258   12aa, >antibacterial10015... at 41.67%
259   12aa, >antibacterial10020... at 50.00%
260   12aa, >antibacterial10021... at 50.00%
261   12aa, >antibacterial10022... at 50.00%
262   20aa, >antibacterial10039... at 45.00%
263   13aa, >antibacterial10053... at 46.15%
264   12aa, >antibacterial10059... at 50.00%
265   21aa, >antibacterial10083... at 57.14%
266   21aa, >antibacterial10084... at 47.62%
267   12aa, >antibacterial10141... at 50.00%
268   12aa, >antibacterial10180... at 58.33%
269   12aa, >antibacterial10181... at 41.67%
270   12aa, >antibacterial10182... at 41.67%
271   14aa, >antibacterial10193... at 42.86%
272   16aa, >antibacterial10219... *
273   17aa, >antibacterial10224... at 47.06%
274   12aa, >antibacterial10233... at 41.67%
275   18aa, >antibacterial10268... at 44.44%
276   21aa, >antibacterial10326... at 42.86%
277   24aa, >antibacterial10370... at 41.67%
278   17aa, >antibacterial10381... at 41.18%
279   17aa, >antibacterial10407... at 52.94%
280   16aa, >antibacterial10410... at 50.00%
281   13aa, >antibacterial10413... at 46.15%
282   15aa, >antibacterial10419... at 35.00%
```

283 15aa, >antibacterial10420... at 35.00%
284 15aa, >antibacterial10422... at 35.00%
285 13aa, >antibacterial10485... at 46.15%
286 17aa, >antibacterial10507... at 41.18%
287 15aa, >antibacterial10519... at 35.00%
288 28aa, >antibacterial10524... at 42.86%
289 12aa, >antibacterial10526... at 50.00%
290 12aa, >antibacterial10532... at 41.67%
291 12aa, >antibacterial10533... at 50.00%
292 11aa, >antibacterial10552... at 45.45%
293 11aa, >antibacterial10562... at 54.55%
294 11aa, >antibacterial10597... at 45.45%
295 13aa, >antibacterial10647... at 46.15%
296 14aa, >antibacterial10648... at 42.86%
297 12aa, >antibacterial10656... at 41.67%
298 12aa, >antibacterial10697... at 41.67%
299 11aa, >antibacterial10778... at 45.45%
300 11aa, >antibacterial10779... at 45.45%
301 11aa, >antibacterial10780... at 45.45%
302 11aa, >antibacterial10781... at 45.45%
303 14aa, >antibacterial10868... at 42.86%
304 34aa, >antibacterial10896... *
305 15aa, >antibacterial10907... at 46.67%
306 13aa, >antibacterial11108... at 46.15%
307 15aa, >antibacterial11133... at 35.00%
308 15aa, >antibacterial11157... at 46.67%
309 12aa, >antibacterial11162... at 41.67%
310 15aa, >antibacterial11180... at 53.33%
311 11aa, >antibacterial11185... at 45.45%
312 15aa, >antibacterial11187... at 35.00%
313 12aa, >antibacterial11219... at 50.00%
314 12aa, >antibacterial11245... at 41.67%
315 14aa, >antibacterial11251... at 57.14%
316 15aa, >antibacterial11268... at 35.00%
317 14aa, >antibacterial11278... *
318 15aa, >antibacterial11298... at 35.00%
319 18aa, >antibacterial11331... at 44.44%
320 11aa, >antibacterial11332... at 45.45%
321 11aa, >antibacterial11335... at 45.45%
322 11aa, >antibacterial11336... at 45.45%
323 11aa, >antibacterial11337... at 45.45%
324 13aa, >antibacterial11338... at 53.85%
325 12aa, >antibacterial11340... at 50.00%
326 18aa, >antibacterial11344... at 44.44%
327 15aa, >antibacterial11360... at 35.00%
328 23aa, >antibacterial11371... at 43.48%
329 35aa, >antibacterial11379... at 42.86%
330 15aa, >antibacterial11472... at 35.00%
331 15aa, >antibacterial11499... at 46.67%
332 35aa, >antibacterial11501... at 35.00%
333 15aa, >antibacterial11506... at 35.00%
334 12aa, >antibacterial11512... at 41.67%
335 15aa, >antibacterial11526... at 53.33%
336 15aa, >antibacterial11536... at 66.67%

337 20aa, >antibacterial11552... at 35.00%
338 20aa, >antibacterial11555... at 35.00%
339 20aa, >antibacterial11564... at 50.00%
340 13aa, >antibacterial11572... at 46.15%
341 18aa, >antibacterial11584... at 44.44%
342 16aa, >antibacterial11585... at 50.00%
343 20aa, >antibacterial11595... at 45.00%
344 15aa, >antibacterial11602... at 35.00%
345 19aa, >antibacterial11610... at 42.11%
346 14aa, >antibacterial11623... at 50.00%
347 12aa, >antibacterial11631... at 41.67%
348 15aa, >antibacterial11642... at 35.00%
349 18aa, >antibacterial11671... at 44.44%
350 15aa, >antibacterial11674... at 35.00%
351 14aa, >antibacterial11675... at 42.86%
352 14aa, >antibacterial11676... at 42.86%
353 15aa, >antibacterial11684... at 35.00%
354 13aa, >antibacterial11700... at 61.54%
355 18aa, >antibacterial11774... at 44.44%
356 21aa, >antibacterial11787... at 42.86%
357 15aa, >antibacterial11827... at 35.00%
358 15aa, >antibacterial11828... at 46.67%
359 15aa, >antibacterial11867... at 35.00%
360 15aa, >antibacterial11869... at 35.00%
361 15aa, >antibacterial11870... at 35.00%
362 15aa, >antibacterial11871... at 35.00%
363 15aa, >antibacterial11872... at 35.00%
364 15aa, >antibacterial11882... at 35.00%
365 12aa, >antibacterial11893... at 41.67%
366 11aa, >antibacterial11904... at 45.45%
367 15aa, >antibacterial11918... at 35.00%
368 20aa, >antibacterial11977... at 35.00%
369 35aa, >antibacterial11980... at 42.86%
370 12aa, >antibacterial11982... at 41.67%
371 12aa, >antibacterial11985... at 41.67%
372 12aa, >antibacterial11986... at 41.67%
373 22aa, >antibacterial11992... at 40.91%
374 12aa, >antibacterial11994... at 58.33%
375 14aa, >antibacterial12071... at 50.00%
376 14aa, >antibacterial12080... at 50.00%
377 14aa, >antibacterial12081... at 42.86%
378 14aa, >antibacterial12082... at 42.86%
379 15aa, >antibacterial12094... at 35.00%
380 12aa, >antibacterial12106... at 50.00%
381 12aa, >antibacterial12118... at 41.67%
382 21aa, >antibacterial12119... at 47.62%
383 14aa, >antibacterial12136... at 50.00%
384 13aa, >antibacterial12143... at 46.15%
385 25aa, >antibacterial12165... at 35.00%
386 25aa, >antibacterial12166... at 48.00%
387 17aa, >antibacterial12181... at 52.94%
388 13aa, >antibacterial12203... at 53.85%
389 13aa, >antibacterial12204... at 53.85%
390 13aa, >antibacterial12205... at 53.85%

391	13aa,	>antibacterial12206...	at 53.85%
392	13aa,	>antibacterial12207...	at 53.85%
393	13aa,	>antibacterial12208...	at 53.85%
394	13aa,	>antibacterial12209...	at 53.85%
395	13aa,	>antibacterial12210...	*
396	13aa,	>antibacterial12219...	at 46.15%
397	13aa,	>antibacterial12234...	at 46.15%
398	14aa,	>antibacterial12284...	at 42.86%
399	14aa,	>antibacterial12287...	at 42.86%
400	12aa,	>antibacterial12342...	at 50.00%
401	12aa,	>antibacterial12343...	at 41.67%
402	15aa,	>antibacterial12344...	at 53.33%
403	11aa,	>antibacterial12351...	at 45.45%
404	18aa,	>antibacterial12352...	at 44.44%
405	11aa,	>antibacterial12355...	at 45.45%
406	12aa,	>antibacterial12356...	at 50.00%
407	12aa,	>antibacterial12357...	at 50.00%
408	12aa,	>antibacterial12358...	at 41.67%
409	12aa,	>antibacterial12359...	at 41.67%
410	12aa,	>antibacterial12367...	at 41.67%
411	23aa,	>antibacterial12370...	at 43.48%
412	13aa,	>antibacterial12424...	at 46.15%
413	15aa,	>antibacterial12427...	at 35.00%
414	26aa,	>antibacterial12434...	at 42.31%
415	12aa,	>antibacterial12446...	at 41.67%
416	11aa,	>antibacterial12520...	at 45.45%
417	14aa,	>antibacterial12523...	at 42.86%
418	14aa,	>antibacterial12524...	at 50.00%
419	11aa,	>antibacterial12530...	at 54.55%
420	15aa,	>antibacterial12541...	at 46.67%
421	11aa,	>antibacterial12542...	at 45.45%
422	15aa,	>antibacterial12550...	at 35.00%
423	15aa,	>antibacterial12551...	at 35.00%
424	15aa,	>antibacterial12596...	at 35.00%
425	20aa,	>antibacterial12616...	at 50.00%
426	15aa,	>antibacterial12642...	at 35.00%
427	11aa,	>antibacterial12681...	at 54.55%
428	13aa,	>antibacterial12706...	at 46.15%
429	15aa,	>antibacterial12714...	at 46.67%
430	11aa,	>antibacterial12733...	at 45.45%
431	13aa,	>antibacterial12735...	at 46.15%
432	29aa,	>antibacterial12752...	at 44.83%
433	15aa,	>antibacterial12772...	at 35.00%
434	29aa,	>antibacterial12787...	at 41.38%
435	15aa,	>antibacterial12804...	at 35.00%
436	20aa,	>antibacterial12850...	at 35.00%
437	15aa,	>antibacterial12868...	at 35.00%
438	13aa,	>antibacterial12887...	at 46.15%
439	12aa,	>antibacterial12909...	at 50.00%
440	12aa,	>antibacterial12914...	*
441	11aa,	>antibacterial12939...	at 45.45%
442	20aa,	>antibacterial12955...	at 45.00%
443	17aa,	>antibacterial12974...	at 41.18%
444	18aa,	>antibacterial13001...	at 44.44%

445   15aa, >antibacterial13007... at 35.00%
446   24aa, >antibacterial13034... at 45.83%
447   18aa, >antibacterial13035... at 44.44%
448   23aa, >antibacterial13079... at 47.83%
449   15aa, >antibacterial13153... at 35.00%
450   12aa, >antibacterial13176... at 41.67%
451   11aa, >antibacterial13218... at 54.55%
452   11aa, >antibacterial13270... at 45.45%
453   11aa, >antibacterial13271... at 45.45%
454   18aa, >antibacterial13293... at 44.44%
455   20aa, >antibacterial13306... at 35.00%
456   18aa, >antibacterial13375... at 44.44%
457   18aa, >antibacterial13376... at 50.00%
458   20aa, >antibacterial13384... at 35.00%
>Cluster 3
0   19aa, >Antibacterial;Antif... at 42.11%
1   24aa, >Antibacterial... at 41.67%
2   28aa, >Antimicrobial... at 42.86%
3   28aa, >Antimicrobial... at 42.86%
4   28aa, >antibacterial;Antif... at 42.86%
5   19aa, >anti-Gram-... at 42.11%
6   23aa, >Antibacterial... at 43.48%
7   24aa, >Antifungal;Antimicr... at 41.67%
8   13aa, >antimicrobial... at 53.85%
9   13aa, >Antibacterial;antim... at 46.15%
10   13aa, >antimicrobial... at 46.15%
11   13aa, >antibacterial;antim... at 46.15%
12   13aa, >Antimicrobial... at 46.15%
13   13aa, >antibacterial;antim... at 53.85%
14   19aa, >anti-Gram+;antifung... at 42.11%
15   15aa, >antibacterial;antim... at 35.00%
16   12aa, >Antimicrobial... at 41.67%
17   19aa, >anti-Gram+;Antibact... at 47.37%
18   27aa, >Antimicrobial... at 40.74%
19   27aa, >antimicrobial... at 40.74%
20   27aa, >antimicrobial... at 40.74%
21   13aa, >Antimicrobial... at 46.15%
22   12aa, >Antibacterial... at 41.67%
23   29aa, >antibacterial;Antif... at 41.38%
24   16aa, >anti-Gram+;antibact... at 43.75%
25   17aa, >antimicrobial... at 41.18%
26   16aa, >antibacterial... at 43.75%
27   22aa, >anti-Gram-;antibact... at 40.91%
28   13aa, >antimicrobial... at 53.85%
29   24aa, >antimicrobial... at 41.67%
30   29aa, >antimicrobial... at 44.83%
31   32aa, >Anti-Gram-;antibact... at 40.62%
32   21aa, >anti-Gram+;antibact... at 42.86%
33   29aa, >antibacterial;antim... at 41.38%
34   24aa, >antibacterial;Antif... at 41.67%
35   12aa, >antithrombotic... at 41.67%
36   14aa, >Antibacterial... at 50.00%
37   28aa, >anti-Gram+;antibact... at 42.86%
38   13aa, >anti-Gram+;antibact... at 46.15%

39 25aa, >anti-Gram+;antifung... at 48.00%
40 18aa, >anti-Gram+;Gram-... at 50.00%
41 15aa, >Antifungal;Antimicr... at 35.00%
42 17aa, >antimicrobial... at 47.06%
43 15aa, >Antibacterial... at 46.67%
44 14aa, >Antimicrobial... at 50.00%
45 14aa, >antibacterial... at 42.86%
46 19aa, >antibacterial;Antif... at 42.11%
47 14aa, >anti-Gram+;antibact... at 42.86%
48 11aa, >Antimicrobial... at 54.55%
49 490aa, >Antimicrobial... *
50 255aa, >Antimicrobial... at 43.14%
51 255aa, >Antimicrobial... at 52.16%
52 255aa, >Antimicrobial... *
53 21aa, >Antibacterial... at 42.86%
54 18aa, >Antimicrobial... at 44.44%
55 13aa, >anti-Gram+;antibact... at 46.15%
56 17aa, >antimicrobial... at 41.18%
57 21aa, >Antibacterial... at 47.62%
58 12aa, >Antibacterial... at 41.67%
59 20aa, >antibacterial06572... at 45.00%
60 16aa, >antibacterial06589... at 43.75%
61 20aa, >antibacterial06707... at 55.00%
62 20aa, >antibacterial06767... at 45.00%
63 14aa, >antibacterial07041... at 50.00%
64 26aa, >antibacterial07062... at 42.31%
65 30aa, >antibacterial07352... at 46.67%
66 12aa, >antibacterial07370... at 41.67%
67 12aa, >antibacterial07551... at 41.67%
68 14aa, >antibacterial07589... at 42.86%
69 11aa, >antibacterial07662... at 54.55%
70 20aa, >antibacterial07663... at 35.00%
71 11aa, >antibacterial07714... at 45.45%
72 26aa, >antibacterial07788... at 42.31%
73 21aa, >antibacterial07930... at 42.86%
74 14aa, >antibacterial07987... at 42.86%
75 20aa, >antibacterial08038... at 35.00%
76 18aa, >antibacterial08077... at 44.44%
77 13aa, >antibacterial08082... at 46.15%
78 11aa, >antibacterial08107... at 45.45%
79 11aa, >antibacterial08108... at 45.45%
80 13aa, >antibacterial08146... at 46.15%
81 13aa, >antibacterial08188... at 46.15%
82 18aa, >antibacterial08195... at 50.00%
83 18aa, >antibacterial08234... at 55.56%
84 12aa, >antibacterial08304... at 41.67%
85 12aa, >antibacterial08343... at 58.33%
86 22aa, >antibacterial08434... at 45.45%
87 14aa, >antibacterial08444... at 42.86%
88 20aa, >antibacterial08480... at 35.00%
89 20aa, >antibacterial08483... at 55.00%
90 20aa, >antibacterial08485... at 35.00%
91 12aa, >antibacterial08504... at 41.67%
92 15aa, >antibacterial08609... at 46.67%

93 14aa, >antibacterial08634... at 42.86%
94 14aa, >antibacterial08635... at 42.86%
95 15aa, >antibacterial08687... at 35.00%
96 15aa, >antibacterial08697... at 35.00%
97 11aa, >antibacterial08753... at 45.45%
98 15aa, >antibacterial08862... at 35.00%
99 15aa, >antibacterial08863... at 35.00%
100 22aa, >antibacterial08880... at 40.91%
101 13aa, >antibacterial08898... at 46.15%
102 14aa, >antibacterial08999... at 57.14%
103 12aa, >antibacterial09018... at 41.67%
104 22aa, >antibacterial09048... at 40.91%
105 20aa, >antibacterial09091... at 35.00%
106 13aa, >antibacterial09139... at 46.15%
107 21aa, >antibacterial09234... at 42.86%
108 12aa, >antibacterial09252... at 41.67%
109 12aa, >antibacterial09254... at 41.67%
110 15aa, >antibacterial09266... at 35.00%
111 12aa, >antibacterial09306... at 41.67%
112 11aa, >antibacterial09322... at 45.45%
113 11aa, >antibacterial09323... at 45.45%
114 11aa, >antibacterial09324... at 54.55%
115 20aa, >antibacterial09403... at 45.00%
116 20aa, >antibacterial09408... at 35.00%
117 20aa, >antibacterial09449... at 35.00%
118 12aa, >antibacterial09502... at 41.67%
119 18aa, >antibacterial09542... at 44.44%
120 18aa, >antibacterial09717... at 44.44%
121 14aa, >antibacterial09744... at 50.00%
122 17aa, >antibacterial09745... at 41.18%
123 14aa, >antibacterial09856... at 42.86%
124 13aa, >antibacterial09865... at 61.54%
125 20aa, >antibacterial09994... at 35.00%
126 12aa, >antibacterial09999... at 41.67%
127 12aa, >antibacterial10016... at 41.67%
128 12aa, >antibacterial10017... at 41.67%
129 14aa, >antibacterial10192... at 42.86%
130 13aa, >antibacterial10212... at 53.85%
131 17aa, >antibacterial10215... at 52.94%
132 14aa, >antibacterial10269... at 42.86%
133 19aa, >antibacterial10312... at 42.11%
134 13aa, >antibacterial10344... at 46.15%
135 13aa, >antibacterial10345... at 46.15%
136 13aa, >antibacterial10375... at 46.15%
137 26aa, >antibacterial10450... at 42.31%
138 12aa, >antibacterial10541... at 50.00%
139 13aa, >antibacterial10544... at 53.85%
140 31aa, >antibacterial10568... at 41.94%
141 14aa, >antibacterial10646... at 50.00%
142 20aa, >antibacterial10676... at 35.00%
143 13aa, >antibacterial10691... at 53.85%
144 17aa, >antibacterial10707... at 41.18%
145 17aa, >antibacterial10708... at 41.18%
146 14aa, >antibacterial10860... at 42.86%

```
147   20aa, >antibacterial10917... at 50.00%
148   20aa, >antibacterial10919... at 55.00%
149   20aa, >antibacterial10920... at 45.00%
150   20aa, >antibacterial10921... at 50.00%
151   20aa, >antibacterial10925... at 60.00%
152   20aa, >antibacterial10926... at 60.00%
153   20aa, >antibacterial10927... at 45.00%
154   20aa, >antibacterial10928... at 45.00%
155   20aa, >antibacterial10929... at 35.00%
156   20aa, >antibacterial10930... at 45.00%
157   20aa, >antibacterial10931... at 50.00%
158   13aa, >antibacterial11132... at 53.85%
159   12aa, >antibacterial11149... at 41.67%
160   12aa, >antibacterial11171... at 50.00%
161   12aa, >antibacterial11173... at 50.00%
162   19aa, >antibacterial11194... at 47.37%
163   15aa, >antibacterial11255... at 53.33%
164   16aa, >antibacterial11280... at 43.75%
165   20aa, >antibacterial11285... at 50.00%
166   15aa, >antibacterial11346... at 35.00%
167   15aa, >antibacterial11355... at 35.00%
168   23aa, >antibacterial11406... at 47.83%
169   12aa, >antibacterial11476... at 50.00%
170   14aa, >antibacterial11482... at 50.00%
171   16aa, >antibacterial11556... at 43.75%
172   18aa, >antibacterial11558... at 50.00%
173   18aa, >antibacterial11559... at 44.44%
174   18aa, >antibacterial11591... at 44.44%
175   11aa, >antibacterial11607... at 45.45%
176   11aa, >antibacterial11608... at 54.55%
177   21aa, >antibacterial11686... at 42.86%
178   20aa, >antibacterial11748... at 35.00%
179   14aa, >antibacterial11770... at 42.86%
180   15aa, >antibacterial11785... at 46.67%
181   26aa, >antibacterial11796... at 50.00%
182   15aa, >antibacterial11854... at 35.00%
183   25aa, >antibacterial11944... at 44.00%
184   14aa, >antibacterial12003... at 50.00%
185   13aa, >antibacterial12012... at 46.15%
186   18aa, >antibacterial12028... at 44.44%
187   20aa, >antibacterial12029... at 35.00%
188   13aa, >antibacterial12147... at 46.15%
189   17aa, >antibacterial12175... at 52.94%
190   17aa, >antibacterial12183... at 41.18%
191   17aa, >antibacterial12185... at 41.18%
192   17aa, >antibacterial12187... at 41.18%
193   17aa, >antibacterial12192... at 41.18%
194   27aa, >antibacterial12375... at 40.74%
195   12aa, >antibacterial12410... at 41.67%
196   15aa, >antibacterial12431... at 35.00%
197   12aa, >antibacterial12454... at 41.67%
198   12aa, >antibacterial12458... at 41.67%
199   15aa, >antibacterial12518... at 35.00%
200   11aa, >antibacterial12522... at 45.45%
```

201   18aa, >antibacterial12537... at 50.00%
202   11aa, >antibacterial12547... at 45.45%
203   31aa, >antibacterial12559... at 41.94%
204   12aa, >antibacterial12560... at 41.67%
205   12aa, >antibacterial12566... at 41.67%
206   15aa, >antibacterial12586... at 35.00%
207   20aa, >antibacterial12705... at 35.00%
208   20aa, >antibacterial12719... at 35.00%
209   12aa, >antibacterial12736... at 41.67%
210   20aa, >antibacterial12761... at 35.00%
211   12aa, >antibacterial12926... at 41.67%
212   12aa, >antibacterial12949... at 41.67%
213   20aa, >antibacterial12957... at 35.00%
214   14aa, >antibacterial12964... at 42.86%
215   17aa, >antibacterial12973... at 41.18%
216   18aa, >antibacterial13100... at 44.44%
217   18aa, >antibacterial13101... at 44.44%
218   15aa, >antibacterial13249... at 46.67%
219   12aa, >antibacterial13276... at 41.67%
220   15aa, >antibacterial13301... at 35.00%
221   12aa, >antibacterial13369... at 41.67%
222   18aa, >antibacterial13377... at 50.00%
223   12aa, >antibacterial13401... at 41.67%
224   12aa, >antibacterial13402... at 41.67%
>Cluster 4
0   11aa, >antithrombotic... at 45.45%
1   28aa, >Antibacterial;antim... at 50.00%
2   13aa, >antimicrobial... *
3   11aa, >antibacterial;antim... at 45.45%
4   11aa, >Antibacterial... at 45.45%
5   13aa, >antimicrobial... at 46.15%
6   17aa, >antimicrobial... at 41.18%
7   15aa, >Antibacterial... at 35.00%
8   14aa, >Antibacterial;antim... at 42.86%
9   13aa, >Antimicrobial... at 46.15%
10   11aa, >antimicrobial... at 45.45%
11   14aa, >anti-Gram+;Antibact... at 42.86%
12   13aa, >anti-Gram+;antibact... at 46.15%
13   13aa, >anti-Gram+;antibact... at 46.15%
14   13aa, >antimicrobial... at 46.15%
15   25aa, >Antibacterial;Antif... at 35.00%
16   33aa, >antimicrobial... at 42.42%
17   21aa, >antimicrobial... at 47.62%
18   21aa, >anti-Gram+;Antibact... at 42.86%
19   21aa, >antimicrobial... at 47.62%
20   16aa, >antibacterial... at 43.75%
21   20aa, >anti-Gram+;antibact... at 35.00%
22   21aa, >antibacterial... at 42.86%
23   14aa, >Antibacterial;Antim... at 42.86%
24   13aa, >antimicrobial... at 46.15%
25   11aa, >antithrombotic... at 45.45%
26   15aa, >antifungal;antibact... at 35.00%
27   20aa, >Antibacterial... at 35.00%
28   19aa, >Antibacterial... at 42.11%

29 15aa, >antibacterial... at 46.67%
30 14aa, >antimicrobial... at 42.86%
31 12aa, >Antimicrobial... at 41.67%
32 359aa, >Antimicrobial... *
33 12aa, >antimicrobial... at 41.67%
34 16aa, >antibacterial;antim... at 50.00%
35 14aa, >antibacterial;antim... at 42.86%
36 15aa, >anti-Gram-;antibact... at 35.00%
37 12aa, >antimicrobial... at 41.67%
38 14aa, >antimicrobial... at 50.00%
39 14aa, >Antifungal... at 64.29%
40 13aa, >antibacterial06526... at 46.15%
41 13aa, >antibacterial06595... at 53.85%
42 13aa, >antibacterial06597... at 53.85%
43 14aa, >antibacterial07523... at 42.86%
44 12aa, >antibacterial07580... at 41.67%
45 19aa, >antibacterial07646... at 42.11%
46 15aa, >antibacterial07681... at 35.00%
47 15aa, >antibacterial07683... at 35.00%
48 15aa, >antibacterial07684... at 35.00%
49 15aa, >antibacterial07685... at 35.00%
50 11aa, >antibacterial07709... at 45.45%
51 11aa, >antibacterial07728... at 54.55%
52 11aa, >antibacterial07731... at 45.45%
53 12aa, >antibacterial07758... at 50.00%
54 12aa, >antibacterial07798... at 41.67%
55 12aa, >antibacterial07800... at 50.00%
56 12aa, >antibacterial07820... at 41.67%
57 20aa, >antibacterial07923... at 35.00%
58 26aa, >antibacterial07932... at 42.31%
59 14aa, >antibacterial07986... at 42.86%
60 14aa, >antibacterial07988... *
61 15aa, >antibacterial08075... at 53.33%
62 24aa, >antibacterial08259... at 41.67%
63 11aa, >antibacterial08432... at 45.45%
64 18aa, >antibacterial08443... at 50.00%
65 14aa, >antibacterial08472... at 42.86%
66 20aa, >antibacterial08478... at 35.00%
67 17aa, >antibacterial08539... at 47.06%
68 15aa, >antibacterial08586... at 35.00%
69 15aa, >antibacterial08736... at 35.00%
70 11aa, >antibacterial08752... at 45.45%
71 11aa, >antibacterial08754... at 45.45%
72 17aa, >antibacterial08824... at 47.06%
73 15aa, >antibacterial08843... at 46.67%
74 14aa, >antibacterial08855... at 50.00%
75 18aa, >antibacterial08912... at 50.00%
76 12aa, >antibacterial09033... at 41.67%
77 14aa, >antibacterial09082... at 42.86%
78 15aa, >antibacterial09103... at 35.00%
79 14aa, >antibacterial09104... at 42.86%
80 13aa, >antibacterial09132... at 53.85%
81 18aa, >antibacterial09290... at 44.44%
82 12aa, >antibacterial09342... at 41.67%

83	12aa,	>antibacterial09344...	at 41.67%
84	20aa,	>antibacterial09429...	at 35.00%
85	25aa,	>antibacterial09537...	at 35.00%
86	25aa,	>antibacterial09538...	at 35.00%
87	11aa,	>antibacterial09574...	at 45.45%
88	13aa,	>antibacterial09593...	at 46.15%
89	14aa,	>antibacterial09634...	at 42.86%
90	19aa,	>antibacterial09712...	*
91	11aa,	>antibacterial09746...	at 45.45%
92	12aa,	>antibacterial09825...	at 38.33%
93	11aa,	>antibacterial09887...	at 45.45%
94	11aa,	>antibacterial09908...	at 45.45%
95	12aa,	>antibacterial10060...	at 41.67%
96	12aa,	>antibacterial10065...	at 50.00%
97	16aa,	>antibacterial10079...	at 50.00%
98	16aa,	>antibacterial10080...	at 50.00%
99	16aa,	>antibacterial10081...	at 50.00%
100	16aa,	>antibacterial10082...	at 43.75%
101	13aa,	>antibacterial10115...	at 46.15%
102	15aa,	>antibacterial10223...	at 46.67%
103	16aa,	>antibacterial10276...	at 50.00%
104	16aa,	>antibacterial10277...	at 50.00%
105	16aa,	>antibacterial10278...	at 50.00%
106	16aa,	>antibacterial10279...	at 50.00%
107	19aa,	>antibacterial10315...	at 42.11%
108	14aa,	>antibacterial10333...	at 42.86%
109	13aa,	>antibacterial10353...	at 53.85%
110	16aa,	>antibacterial10409...	at 43.75%
111	24aa,	>antibacterial10452...	at 41.67%
112	15aa,	>antibacterial10464...	at 35.00%
113	15aa,	>antibacterial10518...	at 35.00%
114	15aa,	>antibacterial10520...	at 35.00%
115	12aa,	>antibacterial10531...	at 50.00%
116	13aa,	>antibacterial10545...	at 53.85%
117	13aa,	>antibacterial10546...	at 53.85%
118	13aa,	>antibacterial10547...	at 53.85%
119	13aa,	>antibacterial10548...	at 53.85%
120	14aa,	>antibacterial10576...	at 42.86%
121	31aa,	>antibacterial10612...	at 41.94%
122	30aa,	>antibacterial10615...	at 35.00%
123	12aa,	>antibacterial10666...	at 41.67%
124	14aa,	>antibacterial10758...	at 42.86%
125	18aa,	>antibacterial10881...	at 50.00%
126	14aa,	>antibacterial11112...	at 50.00%
127	13aa,	>antibacterial11191...	at 61.54%
128	15aa,	>antibacterial11192...	at 53.33%
129	11aa,	>antibacterial11206...	at 45.45%
130	11aa,	>antibacterial11207...	at 54.55%
131	11aa,	>antibacterial11208...	at 45.45%
132	11aa,	>antibacterial11209...	at 45.45%
133	11aa,	>antibacterial11211...	*
134	11aa,	>antibacterial11212...	at 45.45%
135	11aa,	>antibacterial11213...	at 45.45%
136	15aa,	>antibacterial11215...	at 46.67%

137 15aa, >antibacterial11216... at 46.67%
138 12aa, >antibacterial11217... at 50.00%
139 15aa, >antibacterial11220... at 53.33%
140 15aa, >antibacterial11221... at 46.67%
141 15aa, >antibacterial11222... at 53.33%
142 15aa, >antibacterial11225... at 53.33%
143 12aa, >antibacterial11226... at 41.67%
144 15aa, >antibacterial11228... at 46.67%
145 14aa, >antibacterial11233... at 50.00%
146 14aa, >antibacterial11247... at 50.00%
147 21aa, >antibacterial11291... at 42.86%
148 25aa, >antibacterial11326... at 35.00%
149 20aa, >antibacterial11505... at 35.00%
150 12aa, >antibacterial11635... at 41.67%
151 13aa, >antibacterial11704... at 46.15%
152 20aa, >antibacterial11716... at 35.00%
153 12aa, >antibacterial11726... at 41.67%
154 12aa, >antibacterial11727... at 41.67%
155 12aa, >antibacterial11728... at 41.67%
156 23aa, >antibacterial11826... at 43.48%
157 12aa, >antibacterial11887... at 50.00%
158 20aa, >antibacterial11890... at 35.00%
159 15aa, >antibacterial11920... at 35.00%
160 13aa, >antibacterial11943... at 43.85%
161 15aa, >antibacterial12070... at 35.00%
162 13aa, >antibacterial12227... at 36.15%
163 13aa, >antibacterial12243... at 36.15%
164 13aa, >antibacterial12325... at 36.15%
165 11aa, >antibacterial12341... at 45.45%
166 20aa, >antibacterial12402... at 35.00%
167 15aa, >antibacterial12419... at 35.00%
168 12aa, >antibacterial12561... at 41.67%
169 14aa, >antibacterial12690... at 42.86%
170 11aa, >antibacterial12844... at 45.45%
171 11aa, >antibacterial12853... at 45.45%
172 11aa, >antibacterial12857... at 54.55%
173 15aa, >antibacterial12878... at 35.00%
174 15aa, >antibacterial12905... at 46.67%
175 20aa, >antibacterial12951... at 45.00%
176 20aa, >antibacterial12952... at 45.00%
177 20aa, >antibacterial12958... at 45.00%
178 19aa, >antibacterial12959... at 42.11%
179 11aa, >antibacterial12962... at 45.45%
180 18aa, >antibacterial12965... at 44.44%
181 15aa, >antibacterial12971... at 35.00%
182 15aa, >antibacterial12994... at 46.67%
183 17aa, >antibacterial13004... at 41.18%
184 20aa, >antibacterial13038... at 35.00%
185 20aa, >antibacterial13061... at 45.00%
186 17aa, >antibacterial13072... at 41.18%
187 15aa, >antibacterial13108... at 53.33%
188 15aa, >antibacterial13183... at 35.00%
189 15aa, >antibacterial13191... *
190 15aa, >antibacterial13222... at 46.67%

```
191   13aa, >antibacterial13291... at 46.15%
192   15aa, >antibacterial13370... *
>Cluster 5
0     15aa, >antimicrobial... at 35.00%
1     12aa, >antimicrobial... at 41.67%
2     13aa, >antimicrobial... at 46.15%
3     21aa, >antimicrobial... at 42.86%
4     12aa, >antimicrobial... at 41.67%
5     11aa, >anti-Gram+;Antimicr... *
6     11aa, >anti-Gram+;Antimicr... at 45.45%
7     13aa, >Antibacterial;antim... at 46.15%
8     13aa, >anti-Gram+;antibact... at 46.15%
9     22aa, >Gram-... at 40.91%
10    13aa, >antimicrobial... *
11    14aa, >anti-Gram+;antibact... at 42.86%
12    26aa, >antibacterial;antif... at 42.31%
13    19aa, >antibacterial;Antif... at 42.11%
14    26aa, >anti-Gram+;antibact... at 42.31%
15    12aa, >Antibacterial... at 50.00%
16    20aa, >antimicrobial... at 35.00%
17    21aa, >anti-Gram+;Antibact... at 42.86%
18    12aa, >Antibacterial;Antif... at 41.67%
19    21aa, >antimicrobial... at 42.86%
20    25aa, >antimicrobial... at 35.00%
21    14aa, >antimicrobial... at 50.00%
22    16aa, >anti-Gram-;antibact... at 43.75%
23    12aa, >Antibacterial... at 41.67%
24    14aa, >anti-Gram+;Antibact... at 42.86%
25    11aa, >antimicrobial... at 45.45%
26    18aa, >anti-Gram+;antibact... at 44.44%
27    333aa, >Antimicrobial... *
28    18aa, >anti-Gram+;antifung... at 44.44%
29    11aa, >anti-Gram+;antibact... at 45.45%
30    14aa, >Antifungal... at 42.86%
31    15aa, >Antifungal... at 35.00%
32    20aa, >antimicrobial... at 35.00%
33    15aa, >antibacterial... at 46.67%
34    21aa, >antimicrobial... at 42.86%
35    23aa, >antimicrobial... at 43.48%
36    21aa, >antimicrobial... at 42.86%
37    13aa, >antimicrobial... at 46.15%
38    18aa, >antibacterial06640... at 50.00%
39    14aa, >antibacterial07265... at 42.86%
40    25aa, >antibacterial07275... at 44.00%
41    18aa, >antibacterial07351... at 44.44%
42    15aa, >antibacterial07464... at 35.00%
43    15aa, >antibacterial07539... at 46.67%
44    15aa, >antibacterial07540... at 35.00%
45    20aa, >antibacterial07632... at 45.00%
46    20aa, >antibacterial07635... at 35.00%
47    15aa, >antibacterial07687... at 35.00%
48    12aa, >antibacterial07838... at 41.67%
49    11aa, >antibacterial07856... at 45.45%
50    20aa, >antibacterial07929... at 35.00%
```

51	20aa,	>antibacterial07942...	at 35.00%
52	20aa,	>antibacterial08041...	at 45.00%
53	19aa,	>antibacterial08044...	at 42.11%
54	11aa,	>antibacterial08216...	at 45.45%
55	17aa,	>antibacterial08269...	at 41.18%
56	19aa,	>antibacterial08424...	*
57	19aa,	>antibacterial08654...	at 42.11%
58	15aa,	>antibacterial08677...	at 35.00%
59	13aa,	>antibacterial08747...	at 46.15%
60	22aa,	>antibacterial08761...	at 45.45%
61	22aa,	>antibacterial08762...	at 40.91%
62	11aa,	>antibacterial08806...	at 45.45%
63	12aa,	>antibacterial09021...	at 50.00%
64	12aa,	>antibacterial09041...	at 41.67%
65	12aa,	>antibacterial09042...	at 41.67%
66	12aa,	>antibacterial09050...	at 41.67%
67	14aa,	>antibacterial09076...	at 42.86%
68	14aa,	>antibacterial09083...	at 42.86%
69	14aa,	>antibacterial09084...	at 42.86%
70	14aa,	>antibacterial09085...	at 42.86%
71	12aa,	>antibacterial09241...	at 58.33%
72	12aa,	>antibacterial09243...	at 58.33%
73	15aa,	>antibacterial09267...	at 35.00%
74	15aa,	>antibacterial09271...	at 46.67%
75	15aa,	>antibacterial09272...	at 46.67%
76	20aa,	>antibacterial09433...	at 35.00%
77	14aa,	>antibacterial09635...	at 42.86%
78	14aa,	>antibacterial09636...	at 50.00%
79	14aa,	>antibacterial09637...	at 42.86%
80	15aa,	>antibacterial09646...	at 46.67%
81	12aa,	>antibacterial09793...	at 50.00%
82	11aa,	>antibacterial09821...	at 45.45%
83	12aa,	>antibacterial10008...	at 50.00%
84	12aa,	>antibacterial10009...	*
85	12aa,	>antibacterial10010...	at 41.67%
86	12aa,	>antibacterial10019...	at 41.67%
87	12aa,	>antibacterial10135...	at 41.67%
88	17aa,	>antibacterial10178...	at 41.18%
89	12aa,	>antibacterial10238...	at 41.67%
90	12aa,	>antibacterial10241...	at 41.67%
91	15aa,	>antibacterial10360...	at 46.67%
92	11aa,	>antibacterial10500...	at 45.45%
93	11aa,	>antibacterial10504...	at 54.55%
94	12aa,	>antibacterial10527...	at 41.67%
95	12aa,	>antibacterial10530...	at 41.67%
96	12aa,	>antibacterial10539...	at 50.00%
97	29aa,	>antibacterial10614...	at 41.38%
98	14aa,	>antibacterial10795...	at 42.86%
99	24aa,	>antibacterial11106...	at 41.67%
100	14aa,	>antibacterial11252...	at 50.00%
101	15aa,	>antibacterial11296...	at 35.00%
102	15aa,	>antibacterial11388...	at 35.00%
103	15aa,	>antibacterial11404...	at 46.67%
104	15aa,	>antibacterial11538...	at 35.00%

```
105   15aa, >antibacterial11582... at 35.00%
106   15aa, >antibacterial11632... at 35.00%
107   18aa, >antibacterial11640... at 44.44%
108   13aa, >antibacterial11648... at 46.15%
109   15aa, >antibacterial11667... at 35.00%
110   12aa, >antibacterial11731... at 41.67%
111   13aa, >antibacterial11743... *
112   12aa, >antibacterial11981... at 41.67%
113   15aa, >antibacterial12013... at 46.67%
114   15aa, >antibacterial12036... at 35.00%
115   18aa, >antibacterial12076... at 44.44%
116   12aa, >antibacterial12100... at 41.67%
117   13aa, >antibacterial12237... at 46.15%
118   14aa, >antibacterial12302... at 42.86%
119   14aa, >antibacterial12308... at 42.86%
120   15aa, >antibacterial12407... at 46.67%
121   11aa, >antibacterial12416... at 45.45%
122   11aa, >antibacterial12846... at 45.45%
123   11aa, >antibacterial12848... at 45.45%
124   18aa, >antibacterial12888... at 50.00%
125   17aa, >antibacterial12896... at 41.18%
126   15aa, >antibacterial12897... at 35.00%
127   13aa, >antibacterial12989... at 46.15%
128   15aa, >antibacterial13065... at 35.00%
129   15aa, >antibacterial13265... at 35.00%
130   12aa, >antibacterial13278... at 41.67%
131   12aa, >antibacterial13294... at 50.00%
132   15aa, >antibacterial13297... at 35.00%
133   11aa, >antibacterial13352... at 45.45%
>Cluster 6
0     316aa, >Antimicrobial... *
1     22aa, >Antimicrobial... at 40.91%
2     15aa, >Antibacterial... at 35.00%
3     19aa, >anti-Gram-;antibact... at 42.11%
4     20aa, >Antibacterial;antim... at 35.00%
5     13aa, >anti-Gram+;antibact... at 53.85%
6     13aa, >anti-Gram+;antibact... at 36.15%
7     13aa, >antimicrobial... at 36.15%
8     13aa, >anti-Gram+;antibact... *
9     27aa, >antimicrobial.... at 40.74%
10    27aa, >antimicrobial... at 44.44%
11    14aa, >anti-Gram+;antibact... at 50.00%
12    21aa, >antimicrobial... at 42.86%
13    14aa, >Antifungal;antimicr... at 42.86%
14    13aa, >antithrombotic... at 53.85%
15    13aa, >Antibacterial... at 36.15%
16    13aa, >Antibacterial... at 36.15%
17    13aa, >antimicrobial... at 36.15%
18    14aa, >Antimicrobial... at 42.86%
19    23aa, >anti-Gram+;antibact... at 43.48%
20    255aa, >Antimicrobial... *
21    30aa, >anti-Gram+;Antibact... at 35.00%
22    15aa, >antibacterial... at 46.67%
23    20aa, >anti-Gram+;Gram-... at 35.00%
```

```
24    15aa, >Antibacterial... at 35.00%
25    14aa, >Antibacterial... at 57.14%
26    11aa, >Antimicrobial... at 45.45%
27    20aa, >antibacterial06584... at 35.00%
28    25aa, >antibacterial07274... at 35.00%
29    20aa, >antibacterial07353... at 45.00%
30    29aa, >antibacterial07456... *
31    20aa, >antibacterial07762... at 35.00%
32    13aa, >antibacterial07772... at 46.15%
33    13aa, >antibacterial07774... at 46.15%
34    13aa, >antibacterial07775... at 46.15%
35    13aa, >antibacterial07776... at 53.85%
36    13aa, >antibacterial08175... at 46.15%
37    13aa, >antibacterial08182... at 46.15%
38    13aa, >antibacterial08183... at 46.15%
39    20aa, >antibacterial08481... at 35.00%
40    15aa, >antibacterial08595... at 35.00%
41    15aa, >antibacterial08708... at 46.67%
42    15aa, >antibacterial08810... at 46.67%
43    20aa, >antibacterial09447... at 35.00%
44    11aa, >antibacterial09469... at 45.45%
45    11aa, >antibacterial09470... at 54.55%
46    22aa, >antibacterial09617... at 40.91%
47    36aa, >antibacterial09716... *
48    15aa, >antibacterial10154... at 35.00%
49    20aa, >antibacterial10802... at 50.00%
50    15aa, >antibacterial11269... at 35.00%
51    15aa, >antibacterial11339... at 35.00%
52    15aa, >antibacterial11511... at 35.00%
53    15aa, >antibacterial11573... at 35.00%
54    20aa, >antibacterial11577... at 35.00%
55    14aa, >antibacterial11626... at 50.00%
56    11aa, >antibacterial11685... at 54.55%
57    12aa, >antibacterial11721... at 41.67%
58    14aa, >antibacterial12126... at 42.86%
59    13aa, >antibacterial12201... at 46.15%
60    15aa, >antibacterial12364... at 46.67%
61    15aa, >antibacterial12615... at 35.00%
62    11aa, >antibacterial12805... at 54.55%
63    11aa, >antibacterial12807... at 45.45%
64    15aa, >antibacterial12818... at 35.00%
65    11aa, >antibacterial12840... *
66    11aa, >antibacterial12854... at 45.45%
67    11aa, >antibacterial12866... at 45.45%
68    11aa, >antibacterial13075... at 45.45%
69    18aa, >antibacterial13097... at 44.44%
70    20aa, >antibacterial13169... at 35.00%
>Cluster 7
0     16aa, >antibacterial;antim... at 50.00%
1     15aa, >anti-Gram+;Gram-... at 46.67%
2     15aa, >Antimicrobial... at 46.67%
3     15aa, >Antimicrobial... at 35.00%
4     11aa, >antimicrobial... at 45.45%
5     16aa, >anti-Gram+;antibact... at 43.75%
```

6	13aa, >antimicrobial... at 46.15%
7	13aa, >antimicrobial... at 46.15%
8	24aa, >antimicrobial... at 41.67%
9	25aa, >antibacterial;Antif... at 35.00%
10	27aa, >antimicrobial... at 40.74%
11	27aa, >Antibacterial;antim... at 40.74%
12	23aa, >anti-Gram-;Antibact... at 43.48%
13	16aa, >Antibacterial;Antif... at 50.00%
14	18aa, >antimicrobial... at 50.00%
15	17aa, >antibacterial;antib... at 41.18%
16	18aa, >Antibacterial;antim... at 50.00%
17	20aa, >anti-Gram+;Antibact... at 35.00%
18	21aa, >antimicrobial... at 42.86%
19	29aa, >antimicrobial... at 41.38%
20	23aa, >anti-Gram+;Antibact... at 43.48%
21	19aa, >antimicrobial... at 47.37%
22	20aa, >Antifungal... at 55.00%
23	18aa, >antibiotic... at 44.44%
24	13aa, >anti-Gram+;anti-HIV... at 46.15%
25	20aa, >antimicrobial... at 35.00%
26	14aa, >antibacterial;antif... at 42.86%
27	17aa, >anti-Gram+;antibact... at 47.06%
28	19aa, >antimicrobial... at 42.11%
29	11aa, >Antifungal;Antivira... at 45.45%
30	38aa, >antibacterial;antim... *
31	311aa, >Antimicrobial... *
32	17aa, >antimicrobial... at 41.18%
33	12aa, >antimicrobial... at 41.67%
34	11aa, >Antimicrobial... at 45.45%
35	15aa, >Antibacterial;Antif... at 35.00%
36	37aa, >Antimicrobial... at 40.54%
37	14aa, >antithrombotic... at 42.86%
38	12aa, >antifungal... at 41.67%
39	13aa, >antibacterial06464... at 46.15%
40	35aa, >antibacterial07039... at 35.00%
41	29aa, >antibacterial07501... at 41.38%
42	12aa, >antibacterial07836... at 41.67%
43	11aa, >antibacterial07858... *
44	11aa, >antibacterial07860... at 45.45%
45	17aa, >antibacterial07910... at 41.18%
46	20aa, >antibacterial07992... at 35.00%
47	11aa, >antibacterial08222... at 45.45%
48	11aa, >antibacterial08298... at 45.45%
49	11aa, >antibacterial08567... at 54.55%
50	15aa, >antibacterial08610... at 35.00%
51	18aa, >antibacterial08655... at 44.44%
52	15aa, >antibacterial08682... at 35.00%
53	11aa, >antibacterial08757... at 45.45%
54	12aa, >antibacterial08933... at 41.67%
55	16aa, >antibacterial09038... at 43.75%
56	39aa, >antibacterial09298... at 43.59%
57	11aa, >antibacterial09347... at 45.45%
58	20aa, >antibacterial09396... at 35.00%
59	20aa, >antibacterial09438... at 50.00%

60 20aa, >antibacterial09460... at 35.00%
61 20aa, >antibacterial09462... at 35.00%
62 14aa, >antibacterial09479... at 42.86%
63 25aa, >antibacterial09685... at 35.00%
64 12aa, >antibacterial09954... at 41.67%
65 12aa, >antibacterial10013... at 41.67%
66 28aa, >antibacterial10264... at 42.86%
67 25aa, >antibacterial10266... at 48.00%
68 11aa, >antibacterial10502... at 54.55%
69 12aa, >antibacterial10528... at 41.67%
70 12aa, >antibacterial10529... at 41.67%
71 12aa, >antibacterial10542... at 41.67%
72 11aa, >antibacterial10564... at 45.45%
73 11aa, >antibacterial10566... at 54.55%
74 14aa, >antibacterial10581... at 42.86%
75 13aa, >antibacterial10689... at 46.15%
76 19aa, >antibacterial10790... at 47.37%
77 13aa, >antibacterial10894... at 46.15%
78 19aa, >antibacterial11246... at 47.37%
79 15aa, >antibacterial11274... at 35.00%
80 11aa, >antibacterial11276... at 54.55%
81 14aa, >antibacterial11289... at 50.00%
82 14aa, >antibacterial11290... at 42.86%
83 14aa, >antibacterial11292... at 42.86%
84 35aa, >antibacterial11494... at 35.00%
85 15aa, >antibacterial11532... at 35.00%
86 15aa, >antibacterial11551... at 46.67%
87 13aa, >antibacterial11699... *
88 19aa, >antibacterial11713... at 32.11%
89 20aa, >antibacterial11714... at 35.00%
90 19aa, >antibacterial11846... at 32.11%
91 18aa, >antibacterial11912... at 44.44%
92 20aa, >antibacterial11937... at 45.00%
93 14aa, >antibacterial12079... at 42.86%
94 15aa, >antibacterial12128... at 46.67%
95 13aa, >antibacterial12240... at 46.15%
96 13aa, >antibacterial12277... *
97 14aa, >antibacterial12285... at 50.00%
98 13aa, >antibacterial12322... at 46.15%
99 15aa, >antibacterial12514... at 46.67%
100 15aa, >antibacterial12701... at 35.00%
101 14aa, >antibacterial12738... at 42.86%
102 20aa, >antibacterial12743... at 35.00%
103 15aa, >antibacterial12875... at 35.00%
104 15aa, >antibacterial12944... at 35.00%
105 15aa, >antibacterial13067... at 35.00%
106 19aa, >antibacterial13138... at 32.11%
107 19aa, >antibacterial13143... at 32.11%
108 19aa, >antibacterial13144... at 32.11%
109 38aa, >antibacterial13201... *
110 15aa, >antibacterial13204... at 46.67%
111 13aa, >antibacterial13247... at 46.15%
112 15aa, >antibacterial13298... at 53.33%
113 15aa, >antibacterial13315... at 35.00%

```
114   11aa, >antibacterial13350... at 45.45%
115   12aa, >antibacterial13398... at 41.67%
116   12aa, >antibacterial13404... at 41.67%
117   12aa, >antibacterial13408... at 41.67%
>Cluster 8
0     17aa, >Antimicrobial... at 41.18%
1     20aa, >antimicrobial... at 35.00%
2     22aa, >anti-Gram+;antibact... at 40.91%
3     15aa, >antimicrobial... at 35.00%
4     14aa, >antibacterial;Antif... at 42.86%
5     22aa, >antibacterial;antim... at 40.91%
6     19aa, >antimicrobial... *
7     16aa, >anti-Gram+;antibact... at 43.75%
8     17aa, >antifungal;antimicr... at 41.18%
9     13aa, >anti-Gram-;anti-HIV... at 53.85%
10    13aa, >antibacterial;Antif... *
11    13aa, >anti-Gram+;antibact... at 53.85%
12    22aa, >antibacterial;Antif... at 40.91%
13    13aa, >antimicrobial... at 61.54%
14    13aa, >Antibacterial... at 53.85%
15    11aa, >Antimicrobial;Antim... at 45.45%
16    17aa, >anti-Gram+;antibact... at 47.06%
17    13aa, >antibacterial;Antif... at 46.15%
18    15aa, >antimicrobial... at 35.00%
19    15aa, >antibacterial;antim... at 46.67%
20    13aa, >anti-Gram+;antibact... at 46.15%
21    13aa, >anti-Gram+;antibact... at 46.15%
22    24aa, >antibacterial;antim... at 41.67%
23    30aa, >Antimicrobial... *
24    20aa, >antimicrobial... at 35.00%
25    34aa, >anti-Gram+;antibact... at 44.12%
26    23aa, >antimicrobial... at 43.48%
27    19aa, >antimicrobial... at 47.37%
28    25aa, >antibacterial;Antif... at 35.00%
29    21aa, >antimicrobial... at 42.86%
30    12aa, >Antibacterial... at 41.67%
31    12aa, >Antibacterial... at 41.67%
32    14aa, >antibacterial;antim... at 42.86%
33    21aa, >antimicrobial... at 42.86%
34    21aa, >antimicrobial... at 42.86%
35    21aa, >antimicrobial... at 42.86%
36    21aa, >antimicrobial... *
37    32aa, >antibacterial;antim... at 40.62%
38    16aa, >Antimicrobial... at 43.75%
39    16aa, >antibacterial... at 43.75%
40    17aa, >antibacterial;antib... at 52.94%
41    14aa, >antibiotic... at 64.29%
42    14aa, >antibacterial;antib... at 64.29%
43    18aa, >antibiotic... at 44.44%
44    23aa, >Antibacterial;Antif... at 43.48%
45    22aa, >antibacterial... at 45.45%
46    16aa, >Antimicrobial... at 50.00%
47    24aa, >antimicrobial... at 54.17%
48    30aa, >antimicrobial... at 35.00%
```

49 25aa, >anti-Gram+;antibact... at 44.00%
50 21aa, >anti-Gram+;Antimicr... at 52.38%
51 27aa, >antibacterial... at 51.85%
52 25aa, >Antibacterial;antim... at 35.00%
53 23aa, >Antimicrobial;Gram-... at 43.48%
54 21aa, >antimicrobial... at 47.62%
55 22aa, >antibacterial;Antif... at 40.91%
56 21aa, >antimicrobial... at 42.86%
57 30aa, >antimicrobial;Antim... at 35.00%
58 25aa, >anti-Gram-;Antibact... at 44.00%
59 25aa, >antimicrobial... at 35.00%
60 11aa, >antibacterial;antim... at 45.45%
61 17aa, >Antifungal;antimicr... at 41.18%
62 20aa, >anti-Gram+;Antimicr... at 50.00%
63 20aa, >anti-Gram+;antibact... at 35.00%
64 14aa, >Antiviral... at 42.86%
65 14aa, >Antibacterial;Gram-... at 42.86%
66 16aa, >Antibacterial... at 50.00%
67 17aa, >antimicrobial... at 41.18%
68 19aa, >anti-Gram+;antibact... at 42.11%
69 11aa, >Antifungal;Antivira... at 45.45%
70 11aa, >anti-Gram-... at 54.55%
71 20aa, >antibacterial;antif... at 35.00%
72 20aa, >Antibacterial... at 45.00%
73 16aa, >antimicrobial... at 43.75%
74 13aa, >Antifungal;Antimicr... at 46.15%
75 22aa, >Antimicrobial... at 54.55%
76 309aa, >Antimicrobial... *
77 12aa, >antibacterial;antif... at 50.00%
78 12aa, >antibacterial;antif... at 41.67%
79 12aa, >antifungal... at 50.00%
80 11aa, >Antibacterial;Antim... at 54.55%
81 12aa, >antimicrobial... at 41.67%
82 14aa, >antibacterial06535... at 42.86%
83 18aa, >antibacterial06976... *
84 20aa, >antibacterial07031... at 35.00%
85 16aa, >antibacterial07033... at 43.75%
86 26aa, >antibacterial07293... at 46.15%
87 12aa, >antibacterial07422... at 41.67%
88 14aa, >antibacterial07648... at 42.86%
89 23aa, >antibacterial07654... at 47.83%
90 15aa, >antibacterial07656... at 66.67%
91 12aa, >antibacterial07658... at 41.67%
92 22aa, >antibacterial07826... at 50.00%
93 11aa, >antibacterial07868... at 45.45%
94 20aa, >antibacterial07973... at 55.00%
95 14aa, >antibacterial07985... at 42.86%
96 12aa, >antibacterial07995... at 50.00%
97 12aa, >antibacterial07997... *
98 11aa, >antibacterial08221... at 54.55%
99 20aa, >antibacterial08251... at 45.00%
100 18aa, >antibacterial08295... at 50.00%
101 11aa, >antibacterial08305... at 54.55%
102 11aa, >antibacterial08306... at 54.55%

103 20aa, >antibacterial08475... at 35.00%
104 21aa, >antibacterial08490... at 42.86%
105 12aa, >antibacterial08505... at 41.67%
106 14aa, >antibacterial08578... at 57.14%
107 14aa, >antibacterial08637... at 42.86%
108 14aa, >antibacterial08638... at 50.00%
109 14aa, >antibacterial08639... at 50.00%
110 15aa, >antibacterial08646... at 35.00%
111 17aa, >antibacterial08672... at 47.06%
112 15aa, >antibacterial08727... at 35.00%
113 15aa, >antibacterial08818... at 35.00%
114 15aa, >antibacterial08826... at 46.67%
115 13aa, >antibacterial08833... *
116 13aa, >antibacterial08896... at 61.54%
117 13aa, >antibacterial08909... at 61.54%
118 12aa, >antibacterial08940... at 41.67%
119 13aa, >antibacterial08976... at 61.54%
120 16aa, >antibacterial09039... at 56.25%
121 20aa, >antibacterial09088... at 55.00%
122 20aa, >antibacterial09090... at 35.00%
123 18aa, >antibacterial09228... at 55.56%
124 18aa, >antibacterial09229... at 44.44%
125 20aa, >antibacterial09388... at 35.00%
126 20aa, >antibacterial09407... at 35.00%
127 14aa, >antibacterial09435... at 50.00%
128 20aa, >antibacterial09440... at 45.00%
129 16aa, >antibacterial09493... at 56.25%
130 16aa, >antibacterial09494... at 43.75%
131 12aa, >antibacterial09513... at 41.67%
132 12aa, >antibacterial09515... at 41.67%
133 12aa, >antibacterial09516... at 41.67%
134 12aa, >antibacterial09517... at 41.67%
135 24aa, >antibacterial09531... at 45.83%
136 11aa, >antibacterial09613... at 54.55%
137 11aa, >antibacterial09614... at 54.55%
138 11aa, >antibacterial09639... at 54.55%
139 11aa, >antibacterial09644... *
140 18aa, >antibacterial09660... at 44.44%
141 18aa, >antibacterial09661... at 50.00%
142 14aa, >antibacterial09670... at 42.86%
143 25aa, >antibacterial09683... at 35.00%
144 12aa, >antibacterial09785... at 41.67%
145 12aa, >antibacterial09789... at 41.67%
146 11aa, >antibacterial09827... at 45.45%
147 12aa, >antibacterial09949... at 41.67%
148 12aa, >antibacterial09977... at 41.67%
149 12aa, >antibacterial10018... at 50.00%
150 12aa, >antibacterial10029... at 41.67%
151 20aa, >antibacterial10035... at 45.00%
152 16aa, >antibacterial10132... at 43.75%
153 12aa, >antibacterial10139... at 41.67%
154 11aa, >antibacterial10272... at 45.45%
155 26aa, >antibacterial10304... at 46.15%
156 28aa, >antibacterial10457... *

157 11aa, >antibacterial10503... at 63.64%
158 12aa, >antibacterial10567... at 41.67%
159 17aa, >antibacterial10634... at 52.94%
160 14aa, >antibacterial10638... at 42.86%
161 13aa, >antibacterial10686... at 46.15%
162 14aa, >antibacterial10774... at 57.14%
163 22aa, >antibacterial10777... at 40.91%
164 18aa, >antibacterial10846... at 44.44%
165 14aa, >antibacterial10944... at 50.00%
166 18aa, >antibacterial11152... at 44.44%
167 14aa, >antibacterial11241... at 42.86%
168 14aa, >antibacterial11242... at 42.86%
169 18aa, >antibacterial11306... at 44.44%
170 14aa, >antibacterial11477... at 42.86%
171 14aa, >antibacterial11481... at 42.86%
172 15aa, >antibacterial11652... at 46.67%
173 13aa, >antibacterial11703... at 46.15%
174 27aa, >antibacterial11730... at 40.74%
175 15aa, >antibacterial11735... at 35.00%
176 17aa, >antibacterial11771... at 41.18%
177 15aa, >antibacterial11772... at 53.33%
178 23aa, >antibacterial11831... at 43.48%
179 13aa, >antibacterial11838... *
180 24aa, >antibacterial11842... at 41.67%
181 18aa, >antibacterial11856... at 44.44%
182 14aa, >antibacterial11965... at 42.86%
183 13aa, >antibacterial12177... at 53.85%
184 17aa, >antibacterial12184... at 41.18%
185 13aa, >antibacterial12195... at 53.85%
186 13aa, >antibacterial12221... at 56.92%
187 13aa, >antibacterial12244... at 51.54%
188 13aa, >antibacterial12257... at 51.54%
189 13aa, >antibacterial12265... at 53.85%
190 13aa, >antibacterial12268... at 53.85%
191 13aa, >antibacterial12276... at 53.85%
192 14aa, >antibacterial12280... at 42.86%
193 13aa, >antibacterial12281... at 53.85%
194 14aa, >antibacterial12286... at 50.00%
195 13aa, >antibacterial12292... at 53.85%
196 13aa, >antibacterial12293... at 53.85%
197 14aa, >antibacterial12294... at 50.00%
198 14aa, >antibacterial12300... at 42.86%
199 13aa, >antibacterial12304... at 46.15%
200 13aa, >antibacterial12305... at 46.15%
201 13aa, >antibacterial12312... at 46.15%
202 11aa, >antibacterial12338... at 45.45%
203 12aa, >antibacterial12461... at 41.67%
204 20aa, >antibacterial12495... at 35.00%
205 17aa, >antibacterial12502... at 41.18%
206 11aa, >antibacterial12519... at 45.45%
207 12aa, >antibacterial12564... at 58.33%
208 12aa, >antibacterial12567... at 58.33%
209 20aa, >antibacterial12573... at 35.00%
210 18aa, >antibacterial12645... at 44.44%

211   17aa, >antibacterial12655... at 41.18%
212   35aa, >antibacterial12685... *
213   16aa, >antibacterial12686... at 43.75%
214   15aa, >antibacterial12698... at 35.00%
215   14aa, >antibacterial12792... at 42.86%
216   20aa, >antibacterial12801... at 35.00%
217   18aa, >antibacterial12828... at 44.44%
218   15aa, >antibacterial12865... at 46.67%
219   11aa, >antibacterial12867... at 45.45%
220   19aa, >antibacterial13309... at 47.37%
>Cluster 9
0   11aa, >anti-Gram+;antibact... at 45.45%
1   16aa, >anti-Gram+;Antibact... at 43.75%
2   15aa, >Antibacterial... at 35.00%
3   19aa, >antib;antibacterial... at 52.63%
4   14aa, >Antibacterial;Antif... at 42.86%
5   23aa, >antimicrobial... at 43.48%
6   12aa, >Antifungal;Antimicr... at 41.67%
7   16aa, >anti-Gram-;antibact... at 43.75%
8   17aa, >anti-Gram+;Antibact... at 41.18%
9   12aa, >Antifungal... at 41.67%
10   13aa, >antibacterial;Antif... at 46.15%
11   13aa, >anti-Gram+;Antibact... at 46.15%
12   28aa, >antimicrobial... at 46.43%
13   33aa, >Antibacterial;Antif... at 45.45%
14   288aa, >Antimicrobial... *
15   12aa, >antibacterial;antim... at 50.00%
16   12aa, >Antibacterial... at 58.33%
17   17aa, >anti-Gram+;anti-HIV... at 41.18%
18   17aa, >antibacterial;antim... at 41.18%
19   20aa, >Antimicrobial... at 35.00%
20   15aa, >anti-Gram+;Antimicr... at 35.00%
21   11aa, >Antibacterial... at 45.45%
22   11aa, >Antimicrobial... at 45.45%
23   11aa, >Antimicrobial... *
24   20aa, >antibacterial06664... at 50.00%
25   15aa, >antibacterial07586... at 35.00%
26   14aa, >antibacterial07588... at 42.86%
27   19aa, >antibacterial07759... at 42.11%
28   12aa, >antibacterial07876... at 41.67%
29   14aa, >antibacterial07983... at 42.86%
30   19aa, >antibacterial08369... at 42.11%
31   14aa, >antibacterial08640... at 42.86%
32   15aa, >antibacterial08688... at 46.67%
33   12aa, >antibacterial08839... at 41.67%
34   12aa, >antibacterial08840... at 41.67%
35   15aa, >antibacterial08864... at 35.00%
36   12aa, >antibacterial08935... at 41.67%
37   14aa, >antibacterial08997... at 42.86%
38   12aa, >antibacterial09249... at 41.67%
39   20aa, >antibacterial09391... *
40   12aa, >antibacterial09962... at 41.67%
41   12aa, >antibacterial09974... at 41.67%
42   12aa, >antibacterial09975... at 50.00%

```
43    12aa, >antibacterial10007... at 41.67%
44    12aa, >antibacterial10058... at 50.00%
45    12aa, >antibacterial10137... at 41.67%
46    15aa, >antibacterial10150... at 35.00%
47    15aa, >antibacterial10164... at 46.67%
48    15aa, >antibacterial10166... at 35.00%
49    15aa, >antibacterial10167... at 35.00%
50    11aa, >antibacterial10216... at 45.45%
51    12aa, >antibacterial10237... at 50.00%
52    12aa, >antibacterial10244... at 50.00%
53    12aa, >antibacterial10316... at 50.00%
54    11aa, >antibacterial11125... at 45.45%
55    13aa, >antibacterial11128... at 46.15%
56    11aa, >antibacterial11147... at 45.45%
57    14aa, >antibacterial11266... at 42.86%
58    11aa, >antibacterial11329... at 45.45%
59    17aa, >antibacterial11419... at 41.18%
60    20aa, >antibacterial11449... at 35.00%
61    19aa, >antibacterial11633... at 47.37%
62    13aa, >antibacterial11824... *
63    12aa, >antibacterial11979... at 41.67%
64    14aa, >antibacterial12087... at 42.86%
65    12aa, >antibacterial12093... at 41.67%
66    18aa, >antibacterial12134... at 44.44%
67    20aa, >antibacterial12450... at 35.00%
68    15aa, >antibacterial12509... at 46.67%
69    12aa, >antibacterial12659... at 41.67%
70    20aa, >antibacterial12694... at 35.00%
71    11aa, >antibacterial12806... at 54.55%
72    12aa, >antibacterial12836... at 41.67%
73    11aa, >antibacterial12845... at 45.45%
74    11aa, >antibacterial12852... *
75    12aa, >antibacterial13066... at 50.00%
>Cluster 10
0     25aa, >Antifungal... at 44.00%
1     26aa, >antimicrobial... at 42.31%
2     20aa, >antimicrobial... at 35.00%
3     13aa, >anti-Gram+;antibact... at 46.15%
4     17aa, >antibacterial;Antif... at 41.18%
5     12aa, >antifungal... at 41.67%
6     17aa, >antibiotic... at 41.18%
7     13aa, >antibacterial;Antif... at 46.15%
8     17aa, >anti-Gram+;Antimicr... at 41.18%
9     20aa, >antibacterial;antim... at 35.00%
10    285aa, >Antimicrobial... *
11    11aa, >Antiviral... at 45.45%
12    15aa, >Antifungal;Antimicr... at 35.00%
13    15aa, >Antifungal... at 35.00%
14    21aa, >Antimicrobial... at 42.86%
15    21aa, >antimicrobial... at 42.86%
16    16aa, >anti-Gram-... at 43.75%
17    12aa, >antibacterial06480... at 50.00%
18    18aa, >antibacterial06975... at 44.44%
19    12aa, >antibacterial07363... at 41.67%
```

```
20    22aa, >antibacterial07649... at 45.45%
21    11aa, >antibacterial07735... at 45.45%
22    20aa, >antibacterial07754... at 35.00%
23    20aa, >antibacterial07953... at 35.00%
24    19aa, >antibacterial08365... at 42.11%
25    15aa, >antibacterial08577... at 35.00%
26    15aa, >antibacterial08642... at 35.00%
27    15aa, >antibacterial08669... at 35.00%
28    12aa, >antibacterial08965... at 41.67%
29    20aa, >antibacterial09094... *
30    17aa, >antibacterial09547... at 47.06%
31    13aa, >antibacterial09741... at 53.85%
32    12aa, >antibacterial10138... at 41.67%
33    13aa, >antibacterial10461... at 46.15%
34    18aa, >antibacterial10474... at 44.44%
35    17aa, >antibacterial11362... at 41.18%
36    15aa, >antibacterial11465... at 35.00%
37    17aa, >antibacterial11479... at 41.18%
38    13aa, >antibacterial11681... at 46.15%
39    12aa, >antibacterial11900... at 41.67%
40    15aa, >antibacterial11931... at 60.00%
41    15aa, >antibacterial11991... at 35.00%
42    13aa, >antibacterial12153... at 53.85%
43    13aa, >antibacterial12194... at 46.15%
44    13aa, >antibacterial12199... at 46.15%
45    13aa, >antibacterial12226... at 46.15%
46    13aa, >antibacterial12248... at 46.15%
47    13aa, >antibacterial12289... at 53.85%
48    13aa, >antibacterial12290... at 46.15%
49    13aa, >antibacterial12295... at 53.85%
50    20aa, >antibacterial12598... at 35.00%
51    15aa, >antibacterial12634... *
52    15aa, >antibacterial12653... at 35.00%
53    18aa, >antibacterial12726... at 44.44%
54    15aa, >antibacterial12774... at 35.00%
55    12aa, >antibacterial13013... at 50.00%
56    15aa, >antibacterial13030... at 35.00%
57    15aa, >antibacterial13070... at 46.67%
58    12aa, >antibacterial13083... at 41.67%
59    20aa, >antibacterial13155... at 45.00%
60    20aa, >antibacterial13159... at 35.00%
61    13aa, >antibacterial13160... *
62    12aa, >antibacterial13207... at 41.67%
63    15aa, >antibacterial13242... at 35.00%
64    15aa, >antibacterial13300... at 35.00%
65    32aa, >antibacterial13312... at 40.62%
66    12aa, >antibacterial13394... at 41.67%
>Cluster 11
0     255aa, >Antimicrobial... *
1     105aa, >Antimicrobial... at 46.67%
2     106aa, >Antimicrobial... at 47.17%
3     101aa, >Antimicrobial... at 46.53%
4     102aa, >Antimicrobial... at 50.00%
5     90aa, >Antimicrobial... at 51.11%
```

6	102aa, >Antimicrobial... at 43.14%
7	86aa, >Antimicrobial... at 44.19%
8	105aa, >Antimicrobial... at 43.81%
9	104aa, >Antimicrobial... at 43.27%
10	102aa, >Antimicrobial... at 45.10%
11	105aa, >Antimicrobial... at 35.00%
12	105aa, >Antimicrobial... at 45.71%
13	18aa, >anti-Gram+;Antibact... at 44.44%
14	30aa, >antimicrobial... *
15	31aa, >antimicrobial;antiv... at 51.61%
16	29aa, >antimicrobial;antiv... at 41.38%
17	33aa, >Antimicrobial... at 48.48%
18	31aa, >Antimicrobial... at 35.16%
19	31aa, >Antimicrobial... at 35.16%
20	27aa, >antimicrobial... at 51.85%
21	28aa, >Antimicrobial... at 42.86%
22	31aa, >Antimicrobial;Antim... at 35.16%
23	94aa, >Antimicrobial... at 38.94%
24	11aa, >anti-Gram+;antifung... *
25	13aa, >anti-Gram+;Antibact... at 43.85%
26	14aa, >antimicrobial... at 42.86%
27	32aa, >antimicrobial... at 43.75%
28	31aa, >antimicrobial... at 41.94%
29	30aa, >antibacterial;antim... at 50.00%
30	26aa, >anti-Gram+... at 50.00%
31	30aa, >antimicrobial... at 63.33%
32	30aa, >antimicrobial... at 43.33%
33	18aa, >Antibacterial;Antif... at 44.44%
34	30aa, >anti-HIV;antimicrob... at 46.67%
35	29aa, >antimicrobial... at 51.72%
36	30aa, >antimicrobial... at 50.00%
37	33aa, >antimicrobial;antiv... *
38	20aa, >antimicrobial... at 35.00%
39	21aa, >antimicrobial... at 42.86%
40	21aa, >antimicrobial... at 42.86%
41	21aa, >antimicrobial... at 42.86%
42	21aa, >anti-Gram+;Antibact... at 42.86%
43	30aa, >Antimicrobial... at 46.67%
44	30aa, >antimicrobial... at 53.33%
45	31aa, >antimicrobial... at 45.16%
46	30aa, >antimicrobial... at 53.33%
47	30aa, >Antimicrobial;Antim... at 43.33%
48	31aa, >antimicrobial... at 41.94%
49	30aa, >antimicrobial... at 43.33%
50	30aa, >Antimicrobial;Antim... at 46.67%
51	34aa, >antimicrobial... at 44.12%
52	21aa, >antimicrobial... at 42.86%
53	31aa, >antimicrobial... at 41.94%
54	21aa, >Antibacterial;Antif... at 42.86%
55	30aa, >Antimicrobial... at 50.00%
56	30aa, >antimicrobial... at 50.00%
57	29aa, >antimicrobial... at 51.72%
58	29aa, >antimicrobial... at 58.62%
59	29aa, >Antibacterial;antim... at 48.28%

60 29aa, >Antimicrobial... at 51.72%
61 29aa, >Antimicrobial;Antim... at 51.72%
62 29aa, >Antimicrobial... at 55.17%
63 30aa, >anti-Gram-;antibact... at 53.33%
64 30aa, >antimicrobial... at 43.33%
65 31aa, >Antibacterial;Antim... at 41.94%
66 31aa, >Antibacterial;Antim... at 48.39%
67 32aa, >antimicrobial... *
68 31aa, >antimicrobial... at 51.61%
69 31aa, >Antimicrobial;Antim... at 48.39%
70 31aa, >anti-HIV;antimicrob... at 41.94%
71 31aa, >antimicrobial... at 45.16%
72 32aa, >antimicrobial... at 40.62%
73 36aa, >antibacterial;antim... at 41.67%
74 16aa, >anti-Gram+;antibact... at 43.75%
75 29aa, >antibacterial;Antif... at 44.83%
76 30aa, >antimicrobial... at 43.33%
77 25aa, >Antibacterial... at 52.00%
78 29aa, >anti-HIV;antiviral... at 41.38%
79 93aa, >Antimicrobial... at 46.24%
80 93aa, >Antimicrobial... at 48.39%
81 15aa, >anti-Gram+;Antibact... at 46.67%
82 12aa, >antibacterial;antim... at 41.67%
83 11aa, >Antifungal... at 45.45%
84 120aa, >Antimicrobial... at 35.00%
85 198aa, >Antimicrobial... at 42.93%
86 207aa, >Antimicrobial... at 43.48%
87 207aa, >Antimicrobial... at 42.51%
88 207aa, >Antimicrobial... at 41.55%
89 29aa, >antimicrobial... at 41.38%
90 27aa, >Antimicrobial... at 44.44%
91 19aa, >anti-Gram+;antibact... at 47.37%
92 31aa, >anti-HIV;antiviral... at 45.16%
93 35aa, >Antimicrobial... at 35.00%
94 32aa, >antimicrobial... at 40.62%
95 15aa, >Antimicrobial... at 46.67%
96 33aa, >antibacterial;antim... at 45.45%
97 20aa, >antibacterial06443... at 35.00%
98 31aa, >antibacterial06484... at 41.94%
99 30aa, >antibacterial06487... at 50.00%
100 13aa, >antibacterial06534... at 46.15%
101 18aa, >antibacterial06546... at 44.44%
102 31aa, >L02A001811... at 48.39%
103 29aa, >antibacterial06945... at 48.28%
104 30aa, >antibacterial06946... at 50.00%
105 30aa, >antibacterial06948... at 53.33%
106 30aa, >antibacterial07448... at 35.00%
107 31aa, >antibacterial07460... at 45.16%
108 29aa, >antibacterial07794... at 51.72%
109 29aa, >antibacterial07795... at 51.72%
110 29aa, >antibacterial07796... at 51.72%
111 29aa, >antibacterial07797... at 51.72%
112 11aa, >antibacterial08210... at 54.55%
113 30aa, >antibacterial08395... at 35.00%

```
114   20aa, >antibacterial08542... at 35.00%
115   14aa, >antibacterial08832... at 42.86%
116   12aa, >antibacterial09020... at 41.67%
117   19aa, >antibacterial09197... at 42.11%
118   20aa, >antibacterial09404... at 45.00%
119   29aa, >antibacterial09511... at 41.38%
120   29aa, >antibacterial09512... at 41.38%
121   27aa, >antibacterial09526... at 51.85%
122   27aa, >antibacterial09528... at 55.56%
123   26aa, >antibacterial09529... *
124   15aa, >antibacterial10155... at 35.00%
125   15aa, >antibacterial10161... at 35.00%
126   11aa, >antibacterial10270... at 45.45%
127   14aa, >antibacterial11238... at 42.86%
128   18aa, >antibacterial11308... at 44.44%
129   14aa, >antibacterial11387... at 42.86%
130   30aa, >antibacterial11411... at 56.67%
131   30aa, >antibacterial11412... at 50.00%
132   15aa, >antibacterial11605... at 35.00%
133   18aa, >antibacterial11615... at 44.44%
134   24aa, >antibacterial11689... at 45.83%
135   21aa, >antibacterial11794... at 42.86%
136   29aa, >antibacterial11876... at 48.28%
137   12aa, >antibacterial11928... *
138   15aa, >antibacterial11993... at 35.00%
139   15aa, >antibacterial12137... at 46.67%
140   20aa, >antibacterial12140... at 35.00%
141   15aa, >antibacterial12157... at 35.00%
142   15aa, >antibacterial12507... at 53.33%
143   12aa, >antibacterial12563... at 50.00%
144   20aa, >antibacterial12577... at 35.00%
145   15aa, >antibacterial12832... at 35.00%
146   20aa, >antibacterial13052... at 35.00%
147   15aa, >antibacterial13229... at 35.00%
148   18aa, >antibacterial13237... at 44.44%
149   20aa, >antibacterial13387... at 35.00%
>Cluster 12
0     255aa, >Antifungal... *
1     16aa, >antimicrobial... at 43.75%
2     11aa, >antibacterial;antim... at 35.45%
3     14aa, >antimicrobial;Gram-... at 42.86%
4     14aa, >anti-Gram+;Antibact... at 42.86%
5     12aa, >Anti-Gram-... at 50.00%
6     17aa, >anti-Gram+;Antibact... *
7     30aa, >antimicrobial... at 43.33%
8     21aa, >antimicrobial... at 47.62%
9     21aa, >antibacterial;Antif... at 42.86%
10    19aa, >anti-Gram+;Antibact... at 42.11%
11    15aa, >Antimicrobial;Antiv... at 46.67%
12    15aa, >Antifungal;Gram-... at 46.67%
13    21aa, >anti-Gram+;antifung... at 47.62%
14    21aa, >antibacterial;antim... *
15    15aa, >anti-Gram-;antifung... at 35.00%
16    11aa, >anti-Gram+;antibact... at 45.45%
```

17 16aa, >antibacterial;antim... at 43.75%
18 17aa, >antibacterial;antim... at 41.18%
19 19aa, >antimicrobial... at 42.11%
20 12aa, >anti-Gram+;Antibact... at 41.67%
21 25aa, >Antimicrobial... at 35.00%
22 12aa, >antimicrobial... at 41.67%
23 12aa, >antibacterial;antim... at 58.33%
24 17aa, >anti-Gram+;antibact... at 41.18%
25 11aa, >Antifungal;Gram-... at 54.55%
26 34aa, >anti-Gram+;Antibact... at 41.18%
27 11aa, >antithrombotic... at 45.45%
28 14aa, >antithrombotic... at 42.86%
29 14aa, >antimicrobial... at 50.00%
30 16aa, >antimicrobial... at 50.00%
31 19aa, >antibacterial07647... at 42.11%
32 11aa, >antibacterial07715... at 54.55%
33 13aa, >antibacterial07746... at 46.15%
34 12aa, >antibacterial08118... at 41.67%
35 11aa, >antibacterial08226... at 45.45%
36 15aa, >antibacterial08618... at 35.00%
37 17aa, >antibacterial08680... at 41.18%
38 19aa, >antibacterial08712... at 42.11%
39 12aa, >antibacterial09025... at 41.67%
40 13aa, >antibacterial09230... at 46.15%
41 12aa, >antibacterial09250... at 41.67%
42 15aa, >antibacterial09275... at 35.00%
43 20aa, >antibacterial09386... at 35.00%
44 20aa, >antibacterial09411... at 35.00%
45 20aa, >antibacterial09414... at 45.00%
46 20aa, >antibacterial09453... at 45.00%
47 20aa, >antibacterial09464... at 35.00%
48 17aa, >antibacterial09694... at 47.06%
49 13aa, >antibacterial09739... *
50 18aa, >antibacterial09780... at 50.00%
51 11aa, >antibacterial09907... at 45.45%
52 11aa, >antibacterial09912... at 54.55%
53 11aa, >antibacterial09920... at 45.45%
54 12aa, >antibacterial10023... at 41.67%
55 14aa, >antibacterial10196... at 42.86%
56 12aa, >antibacterial10232... at 58.33%
57 13aa, >antibacterial10349... at 46.15%
58 15aa, >antibacterial10359... at 35.00%
59 14aa, >antibacterial10436... at 42.86%
60 12aa, >antibacterial10538... at 41.67%
61 14aa, >antibacterial10637... at 50.00%
62 14aa, >antibacterial10641... at 50.00%
63 12aa, >antibacterial10696... at 41.67%
64 11aa, >antibacterial10699... at 54.55%
65 14aa, >antibacterial10718... at 42.86%
66 15aa, >antibacterial11134... at 35.00%
67 16aa, >antibacterial11183... at 43.75%
68 15aa, >antibacterial11227... at 35.00%
69 15aa, >antibacterial11300... at 35.00%
70 18aa, >antibacterial11305... at 44.44%

71 18aa, >antibacterial11317... at 55.56%
72 25aa, >antibacterial11342... at 35.00%
73 14aa, >antibacterial11483... at 42.86%
74 20aa, >antibacterial11500... at 35.00%
75 25aa, >antibacterial11711... at 35.00%
76 21aa, >antibacterial11808... at 42.86%
77 13aa, >antibacterial12301... at 46.15%
78 13aa, >antibacterial12303... at 46.15%
79 14aa, >antibacterial12374... at 42.86%
80 11aa, >antibacterial12383... at 45.45%
81 20aa, >antibacterial12408... at 50.00%
82 14aa, >antibacterial12499... at 42.86%
83 25aa, >antibacterial12534... at 44.00%
84 11aa, >antibacterial12548... at 45.45%
85 11aa, >antibacterial12549... at 45.45%
86 13aa, >antibacterial12554... at 53.85%
87 15aa, >antibacterial12631... at 35.00%
88 15aa, >antibacterial12636... at 46.67%
89 13aa, >antibacterial12675... at 46.15%
90 12aa, >antibacterial12734... at 41.67%
91 15aa, >antibacterial12767... at 35.00%
92 14aa, >antibacterial13010... at 42.86%
93 12aa, >antibacterial13056... at 41.67%
94 18aa, >antibacterial13165... at 44.44%
95 15aa, >antibacterial13190... *
96 17aa, >antibacterial13216... at 41.18%
97 14aa, >antibacterial13415... at 50.00%
>Cluster 13
0 255aa, >Antibacterial... *
1 27aa, >Antibacterial... at 85.19%
2 49aa, >Antibacterial... *
3 75aa, >Antibacterial... *
4 255aa, >Antibacterial... at 82.55%
5 61aa, >Antibacterial... *
6 255aa, >Antibacterial... at 75.29%
7 255aa, >Antibacterial... at 80.00%
8 255aa, >Antibacterial... at 73.53%
9 255aa, >Antibacterial... *
10 28aa, >antimicrobial... at 40.86%
11 19aa, >antibacterial;antim... at 42.63%
12 20aa, >Antimicrobial... at 35.00%
13 11aa, >antifungal... at 35.45%
14 22aa, >Antifungal;Gram-... at 30.91%
15 12aa, >antimicrobial... at 35.00%
16 15aa, >antimicrobial... at 30.00%
17 13aa, >antibacterial;Antif... at 43.85%
18 16aa, >anti-Gram+;antibact... *
19 16aa, >Antimicrobial... at 35.00%
20 13aa, >anti-Gram+;antibact... at 41.54%
21 15aa, >antimicrobial... at 30.00%
22 13aa, >anti-Gram+;Antibact... at 41.54%
23 13aa, >anti-Gram+;antimicr... at 36.15%
24 25aa, >Anti-Gram-... at 35.00%
25 25aa, >anti-Gram-;antibact... at 35.00%

26 12aa, >Antibacterial... at 41.67%
27 33aa, >antimicrobial... at 45.45%
28 17aa, >anti-Gram+;antibact... at 31.18%
29 23aa, >anti-Gram-;antibact... at 37.83%
30 23aa, >Anti-Gram-;antibact... at 33.48%
31 60aa, >Antibacterial... *
32 255aa, >Antibacterial... at 46.47%
33 19aa, >Antibacterial... at 32.11%
34 13aa, >Antiviral... at 36.15%
35 25aa, >Antimicrobial... at 30.00%
36 20aa, >antimicrobial... at 30.00%
37 16aa, >Antifungal;Antivira... at 33.75%
38 20aa, >antibacterial;Antif... at 30.00%
39 12aa, >antimicrobial... at 31.67%
40 15aa, >Antifungal... at 30.00%
41 26aa, >antibacterial;antim... at 36.15%
42 12aa, >antimicrobial... at 31.67%
43 25aa, >antibacterial;Antif... at 30.00%
44 33aa, >antimicrobial... at 32.42%
45 255aa, >Antibacterial... at 31.57%
46 15aa, >Antifungal;Antimicr... at 36.67%
47 38aa, >antibacterial06493... at 48.42%
48 12aa, >antibacterial07727... at 31.67%
49 11aa, >antibacterial07799... at 35.45%
50 20aa, >antibacterial07956... at 30.00%
51 18aa, >antibacterial07966... at 44.44%
52 14aa, >antibacterial07982... at 32.86%
53 30aa, >antibacterial07991... at 35.00%
54 12aa, >antibacterial08119... at 41.67%
55 12aa, >antibacterial08121... at 41.67%
56 13aa, >antibacterial08168... at 53.85%
57 13aa, >antibacterial08177... at 46.15%
58 13aa, >antibacterial08185... at 53.85%
59 13aa, >antibacterial08189... at 53.85%
60 15aa, >antibacterial08192... at 35.00%
61 15aa, >antibacterial08367... at 35.00%
62 27aa, >antibacterial08422... *
63 15aa, >antibacterial08632... at 35.00%
64 15aa, >antibacterial08742... at 35.00%
65 26aa, >antibacterial08823... at 42.31%
66 19aa, >antibacterial08873... at 42.11%
67 12aa, >antibacterial08970... at 41.67%
68 26aa, >antibacterial09002... at 46.15%
69 12aa, >antibacterial09026... at 41.67%
70 12aa, >antibacterial09028... at 41.67%
71 13aa, >antibacterial09034... at 46.15%
72 12aa, >antibacterial09244... at 41.67%
73 12aa, >antibacterial09246... at 41.67%
74 11aa, >antibacterial09314... at 45.45%
75 15aa, >antibacterial09480... at 35.00%
76 16aa, >antibacterial09546... at 50.00%
77 13aa, >antibacterial09592... at 46.15%
78 14aa, >antibacterial09692... at 42.86%
79 14aa, >antibacterial09695... at 42.86%

80 18aa, >antibacterial09855... *
81 12aa, >antibacterial09955... at 41.67%
82 12aa, >antibacterial09956... at 41.67%
83 12aa, >antibacterial09963... at 41.67%
84 12aa, >antibacterial09964... at 41.67%
85 15aa, >antibacterial09965... at 35.00%
86 13aa, >antibacterial09980... at 46.15%
87 15aa, >antibacterial10157... at 46.67%
88 14aa, >antibacterial10191... at 42.86%
89 20aa, >antibacterial10225... at 45.00%
90 12aa, >antibacterial10243... at 41.67%
91 15aa, >antibacterial10355... at 35.00%
92 15aa, >antibacterial10356... at 35.00%
93 13aa, >antibacterial10448... at 53.85%
94 18aa, >antibacterial10633... at 44.44%
95 16aa, >antibacterial10830... at 43.75%
96 20aa, >antibacterial11375... at 35.00%
97 20aa, >antibacterial11440... at 35.00%
98 16aa, >antibacterial11542... at 43.75%
99 12aa, >antibacterial11723... at 41.67%
100 18aa, >antibacterial11757... at 55.56%
101 15aa, >antibacterial11788... at 35.00%
102 21aa, >antibacterial11859... at 42.86%
103 13aa, >antibacterial11884... at 46.15%
104 21aa, >antibacterial11935... at 42.86%
105 12aa, >antibacterial12011... at 41.67%
106 13aa, >antibacterial12258... at 46.15%
107 13aa, >antibacterial12262... at 46.15%
108 13aa, >antibacterial12263... at 46.15%
109 13aa, >antibacterial12264... at 53.85%
110 13aa, >antibacterial12266... at 46.15%
111 13aa, >antibacterial12267... at 46.15%
112 13aa, >antibacterial12298... *
113 13aa, >antibacterial12299... at 46.15%
114 14aa, >antibacterial12306... at 42.86%
115 14aa, >antibacterial12307... at 42.86%
116 13aa, >antibacterial12324... at 46.15%
117 13aa, >antibacterial12326... at 46.15%
118 12aa, >antibacterial12386... at 41.67%
119 12aa, >antibacterial12387... at 41.67%
120 12aa, >antibacterial12396... at 41.67%
121 12aa, >antibacterial12569... at 41.67%
122 19aa, >antibacterial12585... at 42.11%
123 20aa, >antibacterial12799... at 45.00%
124 18aa, >antibacterial12808... at 50.00%
125 12aa, >antibacterial12911... at 41.67%
126 12aa, >antibacterial12915... at 41.67%
127 12aa, >antibacterial12916... at 41.67%
128 12aa, >antibacterial13114... at 50.00%
129 27aa, >antibacterial13239... at 40.74%
130 13aa, >antibacterial13275... at 53.85%
131 12aa, >antibacterial13282... at 41.67%
132 12aa, >antibacterial13283... at 41.67%
133 12aa, >antibacterial13284... at 38.33%

134   12aa, >antibacterial13396... at 40.00%
>Cluster 14
0   255aa, >Antimicrobial... *
1   13aa, >anti-Gram-;antibact... at 46.15%
2   13aa, >Antibacterial;Antim... *
3   11aa, >Antibacterial... at 45.45%
4   112aa, >Antimicrobial... at 45.54%
5   22aa, >antibacterial;antim... at 40.91%
6   13aa, >antimicrobial... at 46.15%
7   17aa, >Antimicrobial... at 41.18%
8   17aa, >antibacterial;antim... at 41.18%
9   12aa, >Antifungal;Antimicr... at 41.67%
10   12aa, >Antifungal;Antimicr... at 41.67%
11   16aa, >antibacterial... at 50.00%
12   19aa, >anti-Gram-;Antibact... at 42.11%
13   39aa, >antibacterial;antif... at 41.03%
14   11aa, >antimicrobial... at 45.45%
15   32aa, >anti-Gram+;antimicr... *
16   12aa, >antimicrobial... at 41.67%
17   255aa, >Antimicrobial... at 59.61%
18   52aa, >Antimicrobial... *
19   255aa, >Antimicrobial... at 43.14%
20   16aa, >antimicrobial... at 43.75%
21   16aa, >antimicrobial... at 43.75%
22   15aa, >antimicrobial... at 35.00%
23   15aa, >antimicrobial... at 35.00%
24   11aa, >antibacterial... at 45.45%
25   13aa, >antibacterial06463... at 53.85%
26   20aa, >antibacterial06510... at 35.00%
27   35aa, >antibacterial06541... at 35.00%
28   13aa, >antibacterial06939... at 46.15%
29   13aa, >antibacterial07773... at 46.15%
30   12aa, >antibacterial07958... at 41.67%
31   11aa, >antibacterial08285... at 45.45%
32   11aa, >antibacterial08290... at 45.45%
33   15aa, >antibacterial08316... at 35.00%
34   12aa, >antibacterial08326... at 41.67%
35   20aa, >antibacterial08423... at 55.00%
36   15aa, >antibacterial08684... at 35.00%
37   15aa, >antibacterial08690... at 53.33%
38   11aa, >antibacterial08751... *
39   11aa, >antibacterial08756... at 45.45%
40   12aa, >antibacterial08821... at 66.67%
41   25aa, >antibacterial09099... at 48.00%
42   14aa, >antibacterial09878... at 42.86%
43   11aa, >antibacterial09943... at 45.45%
44   15aa, >antibacterial10070... at 35.00%
45   15aa, >antibacterial10071... at 35.00%
46   15aa, >antibacterial10073... at 35.00%
47   15aa, >antibacterial10439... at 46.67%
48   12aa, >antibacterial10505... at 41.67%
49   12aa, >antibacterial10537... at 50.00%
50   27aa, >antibacterial10712... at 40.74%
51   22aa, >antibacterial11267... *

52 12aa, >antibacterial11366... at 31.67%
53 18aa, >antibacterial11586... at 44.44%
54 20aa, >antibacterial11593... at 35.00%
55 14aa, >antibacterial11620... at 50.00%
56 13aa, >antibacterial11861... at 46.15%
57 23aa, >antibacterial11908... at 47.83%
58 12aa, >antibacterial11913... at 41.67%
59 14aa, >antibacterial12085... at 50.00%
60 26aa, >antibacterial12437... at 42.31%
61 13aa, >antibacterial12521... at 33.85%
62 18aa, >antibacterial12614... at 35.00%
63 19aa, >antibacterial12667... at 47.37%
64 20aa, >antibacterial12724... at 35.00%
65 15aa, >antibacterial12791... at 35.00%
66 20aa, >antibacterial12802... at 42.00%
67 20aa, >antibacterial12809... at 35.00%
68 11aa, >antibacterial12856... at 35.45%
69 15aa, >antibacterial12886... at 35.00%
70 25aa, >antibacterial12922... at 44.00%
71 15aa, >antibacterial12935... at 53.33%
72 20aa, >antibacterial13020... at 35.00%
73 12aa, >antibacterial13037... *
74 15aa, >antibacterial13305... at 35.00%
75 11aa, >antibacterial13330... at 45.45%
76 11aa, >antibacterial13347... at 54.55%
77 11aa, >antibacterial13348... at 63.64%
78 11aa, >antibacterial13349... at 45.45%
79 11aa, >antibacterial13351... at 54.55%
80 17aa, >antibacterial13412... at 41.18%
>Cluster 15
0 15aa, >Antibacterial;Antif... at 53.33%
1 255aa, >Antimicrobial... *
2 43aa, >Antimicrobial... at 41.86%
3 43aa, >Antimicrobial... at 41.86%
4 255aa, >Antimicrobial... at 52.16%
5 25aa, >anti-Gram+;Antifung... at 35.00%
6 13aa, >antimicrobial... at 46.15%
7 255aa, >Antimicrobial... at 52.55%
8 20aa, >antibacterial;antim... at 45.00%
9 14aa, >anti-Gram-;antibact... at 42.86%
10 15aa, >antibacterial;antim... at 46.67%
11 13aa, >antibacterial;Antif... *
12 12aa, >Antibacterial... at 41.67%
13 12aa, >Antibacterial... at 41.67%
14 12aa, >anti-Gram+;antibact... at 50.00%
15 18aa, >anti-Gram+;antibact... at 44.44%
16 16aa, >Antimicrobial... at 43.75%
17 21aa, >anti-Gram+;anti-HIV... at 42.86%
18 18aa, >Antibacterial;Antif... at 55.56%
19 26aa, >antibacterial;antim... at 42.31%
20 31aa, >Antimicrobial... at 45.16%
21 14aa, >antibacterial;antim... at 42.86%
22 255aa, >Antimicrobial... at 70.59%
23 54aa, >Antimicrobial... at 40.74%

24 255aa, >Antibacterial... at 55.29%
25 14aa, >Antibacterial;Antim... at 50.00%
26 11aa, >Antifungal;antimicr... at 45.45%
27 230aa, >Antimicrobial... at 47.83%
28 255aa, >Antimicrobial... at 50.59%
29 255aa, >Antimicrobial... at 51.37%
30 255aa, >Antimicrobial... at 35.00%
31 255aa, >Antimicrobial... at 55.69%
32 255aa, >Antimicrobial... at 60.78%
33 255aa, >Antimicrobial... at 60.39%
34 256aa, >Antimicrobial... at 48.24%
35 256aa, >Antimicrobial... at 78.04%
36 256aa, >Antimicrobial... at 49.80%
37 257aa, >Antimicrobial... at 48.63%
38 257aa, >Antimicrobial... at 49.02%
39 257aa, >Antimicrobial... at 54.51%
40 257aa, >Antimicrobial... at 49.02%
41 265aa, >Antimicrobial... at 48.24%
42 258aa, >Antimicrobial... *
43 258aa, >Antimicrobial... at 48.24%
44 182aa, >Antimicrobial... at 42.86%
45 255aa, >Antimicrobial... at 48.24%
46 255aa, >Antimicrobial... at 46.67%
47 255aa, >Antimicrobial... at 47.84%
48 255aa, >Antimicrobial... at 49.41%
49 255aa, >Antimicrobial... at 44.12%
50 255aa, >Antimicrobial... at 46.27%
51 222aa, >Antimicrobial... at 54.05%
52 255aa, >Antimicrobial... at 48.63%
53 255aa, >Antimicrobial... at 50.59%
54 255aa, >Antimicrobial... at 53.33%
55 255aa, >Antimicrobial... at 51.76%
56 255aa, >Antimicrobial... at 42.75%
57 255aa, >Antimicrobial... at 45.49%
58 255aa, >Antimicrobial... at 47.06%
59 255aa, >Antibacterial... at 46.67%
60 255aa, >Antimicrobial... at 50.98%
61 255aa, >Antimicrobial... at 52.16%
62 255aa, >Antimicrobial... at 49.02%
63 255aa, >Antimicrobial... at 46.67%
64 255aa, >Antimicrobial... at 51.37%
65 255aa, >Antimicrobial... at 40.78%
66 255aa, >Antimicrobial... at 50.98%
67 13aa, >antimicrobial... *
68 20aa, >anti-Gram+;antibact... at 35.00%
69 25aa, >antibacterial07320... at 44.00%
70 12aa, >antibacterial07372... at 41.67%
71 29aa, >antibacterial07502... at 41.38%
72 20aa, >antibacterial07631... at 35.00%
73 18aa, >antibacterial08045... at 50.00%
74 13aa, >antibacterial08150... at 46.15%
75 13aa, >antibacterial08165... at 46.15%
76 13aa, >antibacterial08174... at 46.15%
77 17aa, >antibacterial08245... at 41.18%

78 15aa, >antibacterial08318... at 46.67%
79 16aa, >antibacterial08354... at 43.75%
80 16aa, >antibacterial08356... at 43.75%
81 12aa, >antibacterial08503... at 41.67%
82 12aa, >antibacterial08588... at 41.67%
83 14aa, >antibacterial08604... at 50.00%
84 18aa, >antibacterial08668... at 50.00%
85 12aa, >antibacterial08786... at 50.00%
86 12aa, >antibacterial08836... at 41.67%
87 12aa, >antibacterial08987... at 41.67%
88 12aa, >antibacterial08989... at 50.00%
89 21aa, >antibacterial09235... at 47.62%
90 21aa, >antibacterial09236... at 42.86%
91 12aa, >antibacterial09952... at 50.00%
92 12aa, >antibacterial09953... at 41.67%
93 15aa, >antibacterial10151... at 46.67%
94 20aa, >antibacterial10226... at 35.00%
95 19aa, >antibacterial10379... at 47.37%
96 34aa, >antibacterial10604... at 41.18%
97 14aa, >antibacterial10620... at 42.86%
98 29aa, >antibacterial10755... at 44.83%
99 29aa, >antibacterial10756... at 44.83%
100 23aa, >antibacterial11135... at 47.83%
101 14aa, >antibacterial11234... at 50.00%
102 14aa, >antibacterial11257... at 42.86%
103 19aa, >antibacterial11528... at 42.11%
104 27aa, >antibacterial11583... at 44.44%
105 13aa, >antibacterial11609... at 46.15%
106 11aa, >antibacterial11641... at 45.45%
107 24aa, >antibacterial11680... at 41.67%
108 13aa, >antibacterial11696... at 46.15%
109 15aa, >antibacterial11759... at 35.00%
110 15aa, >antibacterial11797... at 35.00%
111 15aa, >antibacterial11997... at 46.67%
112 15aa, >antibacterial12131... *
113 21aa, >antibacterial12349... at 42.86%
114 14aa, >antibacterial12449... at 42.86%
115 17aa, >antibacterial12595... at 47.06%
116 20aa, >antibacterial12742... at 45.00%
117 11aa, >antibacterial13081... at 45.45%
118 12aa, >antibacterial13397... at 41.67%
>Cluster 16
0 14aa, >anti-Gram+;Gram-... at 42.86%
1 255aa, >Antibacterial... *
2 14aa, >antimicrobial... at 42.86%
3 27aa, >anti-Gram+;antibact... at 40.74%
4 27aa, >antimicrobial... at 40.74%
5 21aa, >antimicrobial... *
6 12aa, >Antibacterial... at 41.67%
7 21aa, >antimicrobial... at 47.62%
8 21aa, >Antibacterial... at 42.86%
9 15aa, >Antifungal... at 35.00%
10 19aa, >Antibacterial... *
11 13aa, >antimicrobial... at 46.15%

```
12    11aa, >Antibacterial... at 54.55%
13    13aa, >antibacterial06991... at 46.15%
14    17aa, >antibacterial07790... at 41.18%
15    11aa, >antibacterial07861... at 45.45%
16    11aa, >antibacterial07865... *
17    11aa, >antibacterial07866... at 54.55%
18    30aa, >antibacterial07993... at 35.00%
19    18aa, >antibacterial08032... at 44.44%
20    13aa, >antibacterial08162... at 53.85%
21    17aa, >antibacterial08391... at 41.18%
22    13aa, >antibacterial08705... at 53.85%
23    15aa, >antibacterial08765... at 35.00%
24    11aa, >antibacterial09312... at 54.55%
25    11aa, >antibacterial09316... at 43.64%
26    20aa, >antibacterial09461... at 35.00%
27    26aa, >antibacterial09892... at 42.31%
28    27aa, >antibacterial09893... at 44.44%
29    16aa, >antibacterial09918... at 43.75%
30    12aa, >antibacterial10136... at 41.67%
31    20aa, >antibacterial10340... at 45.00%
32    15aa, >antibacterial11188... at 35.00%
33    14aa, >antibacterial11293... at 50.00%
34    18aa, >antibacterial11307... at 44.44%
35    11aa, >antibacterial11358... at 45.45%
36    25aa, >antibacterial11380... at 35.00%
37    15aa, >antibacterial11549... at 46.67%
38    15aa, >antibacterial11829... at 35.00%
39    20aa, >antibacterial11891... at 45.00%
40    20aa, >antibacterial11914... at 45.00%
41    25aa, >antibacterial11945... at 48.00%
42    13aa, >antibacterial12149... at 46.15%
43    13aa, >antibacterial12176... at 53.85%
44    13aa, >antibacterial12178... at 46.15%
45    13aa, >antibacterial12193... at 46.15%
46    13aa, >antibacterial12222... at 46.15%
47    13aa, >antibacterial12223... at 46.15%
48    13aa, >antibacterial12236... at 46.15%
49    13aa, >antibacterial12238... at 46.15%
50    13aa, >antibacterial12255... at 46.15%
51    13aa, >antibacterial12256... at 46.15%
52    13aa, >antibacterial12278... at 53.85%
53    13aa, >antibacterial12283... at 46.15%
54    20aa, >antibacterial12489... at 35.00%
55    12aa, >antibacterial12558... at 41.67%
56    15aa, >antibacterial12771... at 35.00%
57    20aa, >antibacterial12872... at 35.00%
58    21aa, >antibacterial13032... at 42.86%
59    12aa, >antibacterial13047... at 41.67%
60    20aa, >antibacterial13128... at 35.00%
>Cluster 17
0     255aa, >Antibacterial... *
1     27aa, >antibacterial;Antif... at 40.74%
2     18aa, >anti-Gram+;antimicr... at 44.44%
3     24aa, >Antimicrobial... at 41.67%
```

```
4     13aa, >Gram-... *
5     20aa, >antimicrobial... at 35.00%
6     20aa, >antimicrobial... at 35.00%
7     25aa, >antimicrobial... at 35.00%
8     25aa, >antimicrobial... at 35.00%
9     13aa, >antibacterial;Antif... at 46.15%
10    17aa, >Antimicrobial... at 41.18%
11    15aa, >Antimicrobial... at 46.67%
12    14aa, >antimicrobial... at 42.86%
13    13aa, >anti-Gram-;Antibact... at 46.15%
14    11aa, >Antimicrobial... at 45.45%
15    13aa, >Antibacterial... at 46.15%
16    16aa, >anti-Gram+;Gram-... at 43.75%
17    17aa, >antibacterial06646... at 41.18%
18    15aa, >antibacterial07667... *
19    11aa, >antibacterial07854... at 45.45%
20    13aa, >antibacterial08184... at 46.15%
21    11aa, >antibacterial08209... at 45.45%
22    11aa, >antibacterial08363... at 45.45%
23    18aa, >antibacterial08731... at 50.00%
24    13aa, >antibacterial08894... at 46.15%
25    20aa, >antibacterial09126... at 35.00%
26    17aa, >antibacterial09548... at 41.18%
27    20aa, >antibacterial10033... at 35.00%
28    16aa, >antibacterial10588... at 40.00%
29    16aa, >antibacterial10589... at 43.75%
30    14aa, >antibacterial10792... at 42.86%
31    13aa, >antibacterial10871... *
32    20aa, >antibacterial11495... at 45.00%
33    20aa, >antibacterial11940... at 35.00%
34    15aa, >antibacterial12097... at 35.00%
35    20aa, >antibacterial12397... at 45.00%
36    15aa, >antibacterial12652... at 35.00%
37    20aa, >antibacterial13243... at 35.00%
38    18aa, >antibacterial13274... at 44.44%
39    12aa, >antibacterial13393... at 40.00%
>Cluster 18
0     21aa, >Antifungal... at 42.86%
1     255aa, >Antimicrobial... *
2     15aa, >Antimicrobial... at 35.00%
3     15aa, >antimicrobial... at 35.00%
4     29aa, >antimicrobial... at 44.83%
5     13aa, >anti-Gram+;Antimicr... at 46.15%
6     13aa, >anti-Gram+;Antibact... *
7     13aa, >antimicrobial... at 46.15%
8     19aa, >Antimicrobial... at 42.11%
9     13aa, >Antifungal;Antimicr... at 53.85%
10    13aa, >Antifungal;Antimicr... at 46.15%
11    17aa, >anti-Gram+;antibact... at 47.06%
12    14aa, >anti-Gram+;antibact... at 42.86%
13    12aa, >Antifungal;Antimicr... *
14    12aa, >Antifungal;Antimicr... at 50.00%
15    12aa, >Antifungal;antibact... at 50.00%
16    12aa, >Antifungal;Antimicr... at 41.67%
```

17 12aa, >Antifungal;Antimicr... at 50.00%
18 12aa, >Antifungal;Antimicr... at 41.67%
19 12aa, >Antifungal;Antimicr... at 41.67%
20 21aa, >anti-Gram+;Antimicr... at 42.86%
21 29aa, >anti-Gram+;antibact... at 41.38%
22 17aa, >Antimicrobial... *
23 23aa, >antimicrobial... at 43.48%
24 14aa, >anti-Gram+;antibact... at 42.86%
25 20aa, >anti-Gram+;antibact... at 35.00%
26 13aa, >antimicrobial... at 46.15%
27 11aa, >anti-Gram-;antifung... at 45.45%
28 20aa, >antimicrobial... at 45.00%
29 18aa, >Antibacterial... at 44.44%
30 43aa, >Antimicrobial... at 44.19%
31 17aa, >anti-Gram+;Gram-... at 41.18%
32 14aa, >anti-Gram+;antibact... at 42.86%
33 17aa, >antimicrobial... at 41.18%
34 13aa, >antimicrobial... at 46.15%
35 11aa, >antimicrobial... at 45.45%
36 13aa, >Antibacterial... *
37 15aa, >antimicrobial... at 35.00%
38 15aa, >antimicrobial... at 35.00%
39 26aa, >antibacterial06655... at 42.31%
40 14aa, >antibacterial06660... at 50.00%
41 14aa, >antibacterial06935... *
42 13aa, >antibacterial06965... at 46.15%
43 25aa, >antibacterial07268... at 35.00%
44 26aa, >antimicrobial... at 42.31%
45 17aa, >antibacterial07700... at 47.06%
46 15aa, >antibacterial07825... at 35.00%
47 26aa, >antibacterial07883... at 42.31%
48 20aa, >antibacterial07931... at 35.00%
49 19aa, >antibacterial07937... at 42.11%
50 16aa, >antibacterial08052... at 43.75%
51 13aa, >antibacterial08154... at 46.15%
52 13aa, >antibacterial08181... at 46.15%
53 18aa, >antibacterial08406... at 44.44%
54 19aa, >antibacterial08533... at 42.11%
55 14aa, >antibacterial08602... at 42.86%
56 19aa, >antibacterial08721... at 47.37%
57 11aa, >antibacterial08748... at 45.45%
58 15aa, >antibacterial08802... at 35.00%
59 17aa, >antibacterial08848... at 41.18%
60 16aa, >antibacterial09065... at 43.75%
61 15aa, >antibacterial09163... at 35.00%
62 11aa, >antibacterial09167... at 45.45%
63 11aa, >antibacterial09184... at 45.45%
64 12aa, >antibacterial09247... at 50.00%
65 12aa, >antibacterial09248... at 50.00%
66 12aa, >antibacterial09253... at 41.67%
67 11aa, >antibacterial09833... at 45.45%
68 27aa, >antibacterial09873... at 40.74%
69 11aa, >antibacterial09914... at 45.45%
70 13aa, >antibacterial09990... at 46.15%

```
71    13aa, >antibacterial10121... at 46.15%
72    13aa, >antibacterial10131... at 46.15%
73    12aa, >antibacterial10172... at 41.67%
74    12aa, >antibacterial10218... at 41.67%
75    21aa, >antibacterial10443... at 42.86%
76    20aa, >antibacterial10444... at 35.00%
77    13aa, >antibacterial10458... at 46.15%
78    16aa, >antibacterial10590... at 43.75%
79    16aa, >antibacterial10591... at 46.25%
80    13aa, >antibacterial10631... at 46.15%
81    14aa, >antibacterial11123... *
82    25aa, >antibacterial11138... at 35.00%
83    13aa, >antibacterial11488... at 46.15%
84    36aa, >antibacterial11546... at 41.67%
85    15aa, >antibacterial11561... at 35.00%
86    17aa, >antibacterial11939... at 41.18%
87    14aa, >antibacterial12004... at 42.86%
88    18aa, >antibacterial12122... at 44.44%
89    13aa, >antibacterial12216... at 46.15%
90    18aa, >antibacterial12339... at 50.00%
91    18aa, >antibacterial12340... at 50.00%
92    12aa, >antibacterial12348... at 41.67%
93    12aa, >antibacterial12368... at 41.67%
94    25aa, >antibacterial12422... at 35.00%
95    14aa, >antibacterial12423... *
96    15aa, >antibacterial12432... at 35.00%
97    26aa, >antibacterial12438... at 42.31%
98    11aa, >antibacterial12445... *
99    20aa, >antibacterial12479... at 35.00%
100   20aa, >antibacterial12481... at 35.00%
101   20aa, >antibacterial12483... at 35.00%
102   20aa, >antibacterial12491... at 45.00%
103   15aa, >antibacterial12544... at 46.67%
104   13aa, >antibacterial12545... at 46.15%
105   11aa, >antibacterial12546... at 54.55%
106   15aa, >antibacterial12605... at 46.67%
107   15aa, >antibacterial12718... at 35.00%
108   25aa, >antibacterial12766... at 35.00%
109   11aa, >antibacterial12938... at 45.45%
110   14aa, >antibacterial12999... at 42.86%
111   35aa, >antibacterial13211... at 35.00%
112   15aa, >antibacterial13363... at 35.00%
>Cluster 19
0     222aa, >Antimicrobial... at 50.90%
1     221aa, >Antifungal... at 52.94%
2     226aa, >Antimicrobial... at 45.13%
3     202aa, >Antimicrobial... at 43.07%
4     32aa, >antimicrobial;Antim... at 40.62%
5     13aa, >antimicrobial... at 46.15%
6     255aa, >Antimicrobial... *
7     42aa, >Antimicrobial... at 40.48%
8     255aa, >Antimicrobial... at 51.96%
9     15aa, >antibacterial;antim... at 35.00%
10    13aa, >anti-Gram+;antibact... at 46.15%
```

11	231aa, >Antimicrobial... at 41.13%
12	27aa, >antibacterial;antim... at 40.74%
13	29aa, >antimicrobial... at 44.83%
14	21aa, >antimicrobial... at 47.62%
15	19aa, >anti-Gram+;antibact... *
16	23aa, >antimicrobial... at 43.48%
17	187aa, >Antimicrobial... at 40.11%
18	22aa, >Anti-Gram-;Antibact... at 40.91%
19	25aa, >anti-Gram+;antibact... at 35.00%
20	25aa, >anti-Gram+;antibact... at 35.00%
21	30aa, >anti-Gram+;Antibact... at 35.00%
22	34aa, >anti-Gram-... at 41.18%
23	118aa, >Antimicrobial... at 44.07%
24	13aa, >antimicrobial... at 46.15%
25	13aa, >antimicrobial;antiv... at 46.15%
26	25aa, >anti-Gram+;antibact... at 35.00%
27	224aa, >Antimicrobial... at 43.30%
28	226aa, >Antimicrobial... at 61.50%
29	255aa, >Antimicrobial... at 58.82%
30	255aa, >Antimicrobial... at 60.78%
31	255aa, >Antimicrobial... *
32	247aa, >Antimicrobial... at 48.58%
33	229aa, >Antimicrobial... at 43.23%
34	255aa, >Antimicrobial... at 53.53%
35	249aa, >Antimicrobial... at 48.59%
36	255aa, >Antimicrobial... at 58.04%
37	255aa, >Antimicrobial... at 57.65%
38	255aa, >Antimicrobial... at 72.16%
39	255aa, >Antimicrobial... *
40	235aa, >Antimicrobial... at 41.70%
41	255aa, >Antimicrobial... at 44.71%
42	247aa, >Antimicrobial... at 48.18%
43	255aa, >Antimicrobial... at 56.47%
44	235aa, >Antimicrobial... at 47.23%
45	245aa, >Antimicrobial... at 48.98%
46	236aa, >Antimicrobial... at 42.37%
47	255aa, >Antimicrobial... at 69.80%
48	255aa, >Antimicrobial... at 55.69%
49	234aa, >Antimicrobial... at 43.59%
50	227aa, >Antimicrobial... at 40.53%
51	255aa, >Antimicrobial... at 41.18%
52	233aa, >Antimicrobial... at 48.93%
53	247aa, >Antimicrobial... at 50.61%
54	247aa, >Antimicrobial... at 50.20%
55	245aa, >Antimicrobial... at 54.29%
56	243aa, >Antimicrobial... at 46.09%
57	255aa, >Antimicrobial... at 57.25%
58	244aa, >Antimicrobial... at 52.87%
59	255aa, >Antimicrobial... at 41.57%
60	255aa, >Antimicrobial... at 56.86%
61	246aa, >Antimicrobial... at 50.00%
62	255aa, >Antimicrobial... at 42.35%
63	255aa, >Antimicrobial... at 64.71%
64	249aa, >Antimicrobial... at 42.97%

```
65    233aa, >Antimicrobial... at 46.78%
66    255aa, >Antimicrobial... at 41.57%
67    255aa, >Antimicrobial... at 56.86%
68    255aa, >Antimicrobial... at 41.96%
69    255aa, >Antimicrobial... at 41.57%
70    255aa, >Antimicrobial... at 41.57%
71    233aa, >Antimicrobial... at 48.93%
72    251aa, >Antimicrobial... at 50.20%
73    250aa, >Antimicrobial... at 50.40%
74    248aa, >Antimicrobial... at 53.63%
75    255aa, >Antimicrobial... at 54.51%
76    255aa, >Antimicrobial... at 58.82%
77    255aa, >Antimicrobial... at 58.43%
78    250aa, >Antimicrobial... at 49.60%
79    255aa, >Antimicrobial... at 60.00%
80    255aa, >Antimicrobial... at 59.22%
81    236aa, >Antimicrobial... at 48.31%
82    255aa, >Antimicrobial... at 40.39%
83    255aa, >Antimicrobial... at 47.84%
84    255aa, >Antimicrobial... at 43.53%
85    249aa, >Antimicrobial... at 51.41%
86    255aa, >Antimicrobial... at 55.69%
87    255aa, >Antimicrobial... at 53.73%
88    253aa, >Antimicrobial... at 48.22%
89    247aa, >Antimicrobial... at 50.61%
90    254aa, >Antimicrobial... at 46.85%
91    241aa, >Antimicrobial... at 48.96%
92    239aa, >Antimicrobial... at 50.21%
93    255aa, >Antimicrobial... at 43.92%
94    234aa, >Antimicrobial... at 50.85%
95    255aa, >Antimicrobial... at 48.63%
96    255aa, >Antimicrobial... at 64.31%
97    255aa, >Antimicrobial... *
98    226aa, >Antimicrobial... at 48.67%
99    231aa, >Antimicrobial... at 40.26%
100   255aa, >Antimicrobial... at 47.84%
101   255aa, >Antimicrobial... at 60.78%
102   255aa, >Antimicrobial... at 35.00%
103   255aa, >Antimicrobial... at 62.75%
104   248aa, >Antimicrobial... at 45.56%
105   234aa, >Antimicrobial... at 47.86%
106   233aa, >Antimicrobial... at 42.92%
107   255aa, >Antimicrobial... at 43.92%
108   255aa, >Antimicrobial... at 56.86%
109   255aa, >Antimicrobial... at 62.75%
110   255aa, >Antimicrobial... at 55.69%
111   255aa, >Antimicrobial... at 45.49%
112   248aa, >Antimicrobial... at 42.74%
113   255aa, >Antimicrobial... at 45.88%
114   255aa, >Antimicrobial... at 62.35%
115   255aa, >Antimicrobial... at 58.82%
116   244aa, >Antimicrobial... at 47.54%
117   255aa, >Antimicrobial... at 63.14%
118   255aa, >Antimicrobial... at 58.82%
```

119 255aa, >Antimicrobial... at 54.90%
120 233aa, >Antimicrobial... at 42.92%
121 255aa, >Antimicrobial... at 58.43%
122 255aa, >Antimicrobial... at 67.45%
123 240aa, >Antimicrobial... at 54.58%
124 255aa, >Antimicrobial... at 54.51%
125 251aa, >Antimicrobial... at 48.21%
126 251aa, >Antimicrobial... at 50.60%
127 253aa, >Antimicrobial... at 40.32%
128 255aa, >Antimicrobial... at 56.08%
129 255aa, >Antimicrobial... at 56.08%
130 255aa, >Antimicrobial... at 58.04%
131 255aa, >Antimicrobial... at 58.04%
132 255aa, >Antimicrobial... at 45.88%
133 243aa, >Antimicrobial... at 47.33%
134 255aa, >Antimicrobial... at 52.16%
135 255aa, >Antimicrobial... at 58.43%
136 255aa, >Antimicrobial... at 71.76%
137 229aa, >Antimicrobial... at 42.79%
138 227aa, >Antimicrobial... at 44.49%
139 255aa, >Antimicrobial... at 54.90%
140 252aa, >Antimicrobial... at 40.48%
141 254aa, >Antimicrobial... at 40.55%
142 228aa, >Antimicrobial... at 45.61%
143 251aa, >Antimicrobial... at 41.04%
144 255aa, >Antimicrobial... at 66.67%
145 251aa, >Antimicrobial... at 40.64%
146 225aa, >Antifungal... at 45.33%
147 248aa, >Antimicrobial... at 42.34%
148 242aa, >Antimicrobial... at 50.00%
149 251aa, >Antimicrobial... at 62.15%
150 232aa, >Antimicrobial... at 40.95%
151 226aa, >Antimicrobial... at 50.88%
152 233aa, >Antimicrobial... at 47.64%
153 255aa, >Antimicrobial... at 62.75%
154 255aa, >Antimicrobial... at 54.90%
155 253aa, >Antimicrobial... at 43.87%
156 251aa, >Antimicrobial... at 42.63%
157 227aa, >Antimicrobial... at 43.17%
158 223aa, >Antimicrobial... at 41.26%
159 255aa, >Antimicrobial... at 61.57%
160 255aa, >Antimicrobial... at 49.41%
161 246aa, >Antimicrobial... at 45.53%
162 248aa, >Antimicrobial... at 47.98%
163 230aa, >Antimicrobial... at 45.65%
164 255aa, >Antimicrobial... at 43.53%
165 242aa, >Antimicrobial... at 57.44%
166 255aa, >Antimicrobial... at 61.57%
167 248aa, >Antimicrobial... at 47.18%
168 240aa, >Antimicrobial... at 45.00%
169 239aa, >Antimicrobial... at 43.93%
170 239aa, >Antimicrobial... at 44.35%
171 248aa, >Antimicrobial... at 42.34%
172 248aa, >Antimicrobial... at 41.13%

173 255aa, >Antimicrobial... at 60.00%
174 255aa, >Antimicrobial... at 60.00%
175 255aa, >Antimicrobial... *
176 255aa, >Antimicrobial... at 64.31%
177 255aa, >Antimicrobial... at 63.92%
178 255aa, >Antimicrobial... at 61.57%
179 253aa, >Antimicrobial... at 60.47%
180 255aa, >Antimicrobial... at 55.69%
181 255aa, >Antimicrobial... at 55.29%
182 255aa, >Antimicrobial... at 56.86%
183 255aa, >Antimicrobial... at 61.18%
184 255aa, >Antimicrobial... at 45.88%
185 255aa, >Antimicrobial... at 56.08%
186 255aa, >Antimicrobial... at 60.78%
187 255aa, >Antimicrobial... at 58.82%
188 255aa, >Antimicrobial... at 59.61%
189 255aa, >Antimicrobial... at 61.18%
190 255aa, >Antimicrobial... at 60.39%
191 255aa, >Antimicrobial... at 64.71%
192 255aa, >Antimicrobial... at 61.18%
193 255aa, >Antimicrobial... at 56.86%
194 255aa, >Antimicrobial... at 56.47%
195 232aa, >Antimicrobial... at 45.26%
196 255aa, >Antimicrobial... at 45.49%
197 249aa, >Antimicrobial... at 48.19%
198 255aa, >Antimicrobial... at 50.98%
199 242aa, >Antimicrobial... at 51.65%
200 232aa, >Antimicrobial... at 47.84%
201 248aa, >Antimicrobial... at 47.18%
202 246aa, >Antimicrobial... at 48.78%
203 255aa, >Antimicrobial... *
204 255aa, >Antimicrobial... at 46.67%
205 255aa, >Antimicrobial... at 52.94%
206 255aa, >Antimicrobial... at 45.10%
207 238aa, >Antimicrobial... at 49.16%
208 238aa, >Antimicrobial... at 48.74%
209 255aa, >Antimicrobial... at 54.51%
210 242aa, >Antimicrobial... at 52.07%
211 255aa, >Antimicrobial... at 49.02%
212 255aa, >Antimicrobial... at 57.65%
213 255aa, >Antimicrobial... at 53.33%
214 255aa, >Antimicrobial... at 54.12%
215 255aa, >Antimicrobial... at 47.06%
216 182aa, >Antimicrobial... at 40.11%
217 255aa, >Antimicrobial... at 55.29%
218 255aa, >Antimicrobial... at 55.29%
219 255aa, >Antimicrobial... at 53.73%
220 233aa, >Antimicrobial... at 46.78%
221 255aa, >Antimicrobial... at 54.90%
222 255aa, >Antimicrobial... at 54.90%
223 255aa, >Antimicrobial... at 57.65%
224 228aa, >Antimicrobial... at 40.35%
225 244aa, >Antimicrobial... at 49.59%
226 234aa, >Antimicrobial... at 47.01%

227 181aa, >Antimicrobial... *
228 255aa, >Antimicrobial... at 40.88%
229 255aa, >Antimicrobial... at 58.04%
230 255aa, >Antimicrobial... at 44.31%
231 255aa, >Antimicrobial... at 60.00%
232 255aa, >Antimicrobial... at 59.61%
233 255aa, >Antimicrobial... at 60.78%
234 255aa, >Antimicrobial... at 61.18%
235 255aa, >Antimicrobial... at 58.43%
236 234aa, >Antimicrobial... at 45.73%
237 255aa, >Antimicrobial... at 51.76%
238 255aa, >Antimicrobial... at 54.90%
239 247aa, >Antimicrobial... at 42.91%
240 255aa, >Antimicrobial... at 41.18%
241 255aa, >Antimicrobial... at 55.29%
242 247aa, >Antimicrobial... at 40.08%
243 246aa, >Antimicrobial... at 40.65%
244 244aa, >Antimicrobial... at 40.16%
245 255aa, >Antimicrobial... at 51.37%
246 234aa, >Antimicrobial... at 40.60%
247 255aa, >Antimicrobial... at 55.29%
248 245aa, >Antimicrobial... at 40.82%
249 222aa, >Antimicrobial... at 44.59%
250 255aa, >Antimicrobial... at 54.12%
251 244aa, >Antimicrobial... at 50.82%
252 248aa, >Antimicrobial... at 43.95%
253 255aa, >Antimicrobial... at 42.75%
254 255aa, >Antimicrobial... at 40.39%
255 255aa, >Antimicrobial... at 47.06%
256 255aa, >Antimicrobial... at 40.78%
257 247aa, >Antimicrobial... at 47.77%
258 248aa, >Antimicrobial... at 49.19%
259 252aa, >Antimicrobial... at 52.38%
260 254aa, >Antimicrobial... at 42.91%
261 220aa, >Antimicrobial... at 40.91%
262 232aa, >Antimicrobial... at 46.55%
263 255aa, >Antimicrobial... at 59.22%
264 255aa, >Antimicrobial... at 50.98%
265 228aa, >Antimicrobial... at 41.23%
266 250aa, >Antimicrobial... at 46.80%
267 255aa, >Antimicrobial... at 51.76%
268 255aa, >Antimicrobial... at 51.76%
269 242aa, >Antimicrobial... at 45.45%
270 246aa, >Antimicrobial... at 53.66%
271 246aa, >Antimicrobial... at 52.03%
272 245aa, >Antifungal... at 47.35%
273 242aa, >Antimicrobial... at 47.93%
274 255aa, >Antimicrobial... at 56.86%
275 255aa, >Antimicrobial... at 60.00%
276 244aa, >Antimicrobial... at 47.95%
277 243aa, >Antimicrobial... at 52.67%
278 243aa, >Antimicrobial... at 49.38%
279 247aa, >Antimicrobial... at 49.39%
280 255aa, >Antimicrobial... at 46.27%

281 242aa, >Antimicrobial... *
282 255aa, >Antimicrobial... at 49.41%
283 255aa, >Antimicrobial... at 50.98%
284 255aa, >Antimicrobial... at 50.20%
285 248aa, >Antimicrobial... at 43.95%
286 246aa, >Antimicrobial... at 45.53%
287 245aa, >Antimicrobial... at 35.00%
288 255aa, >Antimicrobial... at 58.43%
289 255aa, >Antimicrobial... at 40.78%
290 255aa, >Antimicrobial... at 41.18%
291 248aa, >Antimicrobial... at 43.15%
292 255aa, >Antimicrobial... at 58.43%
293 229aa, >Antimicrobial... at 47.60%
294 231aa, >Antimicrobial... at 46.32%
295 149aa, >Antimicrobial... at 53.69%
296 244aa, >Antimicrobial... at 44.26%
297 244aa, >Antimicrobial... at 43.85%
298 255aa, >Antimicrobial... at 57.06%
299 230aa, >Antimicrobial... at 46.96%
300 255aa, >Antimicrobial... at 53.73%
301 255aa, >Antimicrobial... at 54.51%
302 154aa, >Antimicrobial... at 42.86%
303 255aa, >Antimicrobial... at 54.12%
304 255aa, >Antimicrobial... at 52.55%
305 255aa, >Antimicrobial... at 52.16%
306 248aa, >Antimicrobial... at 51.21%
307 246aa, >Antimicrobial... at 40.65%
308 255aa, >Antimicrobial... at 53.33%
309 255aa, >Antimicrobial... at 53.33%
310 255aa, >Antimicrobial... at 60.00%
311 255aa, >Antimicrobial... at 61.96%
312 230aa, >Antimicrobial... at 41.30%
313 255aa, >Antimicrobial... at 60.39%
314 255aa, >Antimicrobial... at 42.35%
315 255aa, >Antimicrobial... at 43.14%
316 255aa, >Antimicrobial... *
317 250aa, >Antimicrobial... at 47.20%
318 251aa, >Antimicrobial... at 51.79%
319 255aa, >Antimicrobial... at 44.71%
320 171aa, >Antimicrobial... at 40.94%
321 238aa, >Antimicrobial... at 41.18%
322 252aa, >Antimicrobial... at 44.05%
323 248aa, >Antimicrobial... at 45.97%
324 255aa, >Antimicrobial... at 45.10%
325 255aa, >Antimicrobial... at 54.12%
326 248aa, >Antimicrobial... at 47.18%
327 255aa, >Antimicrobial... at 35.00%
328 214aa, >Antimicrobial... at 46.73%
329 151aa, >Antimicrobial... at 45.03%
330 12aa, >antimicrobial... at 41.67%
331 177aa, >Antimicrobial... at 45.76%
332 233aa, >Antimicrobial... at 52.36%
333 180aa, >Antimicrobial... at 42.22%
334 13aa, >Antimicrobial... at 46.15%

```
335   255aa, >Antimicrobial... at 58.82%
336   165aa, >Antimicrobial... at 42.42%
337   26aa, >Antimicrobial... at 53.85%
338   255aa, >Antimicrobial... at 58.43%
339   16aa, >antimicrobial... at 43.75%
340   11aa, >antibacterial06638... at 45.45%
341   20aa, >antibacterial06663... at 45.00%
342   30aa, >antibacterial06947... at 35.00%
343   12aa, >antibacterial08028... at 41.67%
344   18aa, >antibacterial08043... at 50.00%
345   13aa, >antibacterial08158... at 53.85%
346   20aa, >antibacterial08255... at 35.00%
347   15aa, >antibacterial08659... at 35.00%
348   21aa, >antibacterial09060... at 42.86%
349   20aa, >antibacterial09384... at 35.00%
350   15aa, >antibacterial09836... at 35.00%
351   11aa, >antibacterial09870... at 45.45%
352   11aa, >antibacterial10510... at 45.45%
353   26aa, >antibacterial10592... at 42.31%
354   18aa, >antibacterial10700... at 44.44%
355   30aa, >antibacterial10750... at 35.00%
356   12aa, >antibacterial10906... at 41.67%
357   37aa, >antibacterial10910... at 40.54%
358   55aa, >antibacterial10911... at 41.82%
359   14aa, >antibacterial11159... at 42.86%
360   11aa, >antibacterial11203... at 45.45%
361   11aa, >antibacterial11205... at 45.45%
362   14aa, >antibacterial11231... at 42.86%
363   14aa, >antibacterial11236... at 42.86%
364   14aa, >antibacterial11243... at 50.00%
365   15aa, >antibacterial11369... *
366   22aa, >antibacterial11430... at 40.91%
367   19aa, >antibacterial11456... at 52.63%
368   19aa, >antibacterial11459... at 47.37%
369   11aa, >antibacterial11543... *
370   12aa, >antibacterial11722... at 41.67%
371   17aa, >antibacterial11973... at 41.18%
372   12aa, >antibacterial12159... at 41.67%
373   12aa, >antibacterial12473... at 58.33%
374   15aa, >antibacterial12632... at 35.00%
375   12aa, >antibacterial12661... at 41.67%
376   20aa, >antibacterial12684... at 35.00%
377   18aa, >antibacterial12745... at 44.44%
378   15aa, >antibacterial12869... at 35.00%
379   12aa, >antibacterial12913... at 41.67%
380   22aa, >antibacterial13173... at 45.45%
381   17aa, >antibacterial13240... at 41.18%
382   18aa, >antibacterial13361... at 44.44%
>Cluster 20
0     17aa, >anti-Gram+;Antibact... at 41.18%
1     11aa, >antibacterial... at 54.55%
2     255aa, >Antibacterial;Antif... *
3     255aa, >Antibacterial... at 50.20%
4     13aa, >antibacterial;antim... at 53.85%
```

5 16aa, >antibacterial;antif... at 43.75%
6 13aa, >Antibacterial;antim... at 46.15%
7 18aa, >Antibacterial;Antif... at 44.44%
8 13aa, >antimicrobial... at 46.15%
9 13aa, >antibacterial;antim... *
10 14aa, >anti-Gram+;antibact... at 50.00%
11 24aa, >antimicrobial... at 45.83%
12 13aa, >antimicrobial... at 46.15%
13 19aa, >antibacterial;antim... at 42.11%
14 13aa, >antimicrobial... at 46.15%
15 14aa, >Antibacterial;antim... at 42.86%
16 24aa, >antimicrobial... at 41.67%
17 31aa, >anti-Gram+;antibact... at 41.94%
18 11aa, >antibacterial;Antif... at 45.45%
19 22aa, >antimicrobial... at 45.45%
20 16aa, >Antibacterial... at 43.75%
21 18aa, >antibacterial;antif... at 44.44%
22 11aa, >Antiviral... at 45.45%
23 20aa, >Antifungal... at 50.00%
24 15aa, >Antifungal;Antimicr... at 53.33%
25 40aa, >Antimicrobial... at 35.00%
26 16aa, >anti-Gram+;Antibact... at 43.75%
27 16aa, >antimicrobial... at 43.75%
28 255aa, >Antibacterial... at 60.78%
29 33aa, >antimicrobial... at 42.42%
30 15aa, >anti-Gram+;Gram-... at 35.00%
31 20aa, >antibacterial06530... at 35.00%
32 14aa, >antibacterial06667... at 42.86%
33 18aa, >antibacterial06972... at 50.00%
34 23aa, >antibacterial07468... at 43.48%
35 21aa, >antibacterial07673... at 42.86%
36 14aa, >antibacterial07768... at 42.86%
37 18aa, >antibacterial07779... at 50.00%
38 24aa, >antibacterial07823... at 45.83%
39 20aa, >antibacterial07939... at 35.00%
40 22aa, >antibacterial08050... at 40.91%
41 20aa, >antibacterial08063... at 35.00%
42 24aa, >antibacterial08127... at 41.67%
43 22aa, >antibacterial08537... at 40.91%
44 14aa, >antibacterial08835... at 50.00%
45 13aa, >antibacterial08899... at 46.15%
46 13aa, >antibacterial08900... at 53.85%
47 13aa, >antibacterial09137... at 53.85%
48 11aa, >antibacterial09185... at 54.55%
49 11aa, >antibacterial09186... at 45.45%
50 21aa, >antibacterial09238... at 42.86%
51 15aa, >antibacterial09268... at 35.00%
52 15aa, >antibacterial09273... at 46.67%
53 15aa, >antibacterial09274... at 46.67%
54 18aa, >antibacterial09327... at 44.44%
55 11aa, >antibacterial09346... at 45.45%
56 20aa, >antibacterial09364... at 50.00%
57 20aa, >antibacterial09383... at 35.00%
58 20aa, >antibacterial09416... at 45.00%

59 20aa, >antibacterial09425... at 45.00%
60 20aa, >antibacterial09437... at 35.00%
61 11aa, >antibacterial09576... at 45.45%
62 15aa, >antibacterial09679... at 46.67%
63 11aa, >antibacterial09747... at 45.45%
64 12aa, >antibacterial09779... at 41.67%
65 13aa, >antibacterial09862... at 46.15%
66 13aa, >antibacterial09864... at 46.15%
67 11aa, >antibacterial09872... at 63.64%
68 11aa, >antibacterial09881... *
69 11aa, >antibacterial09885... at 45.45%
70 11aa, >antibacterial09889... at 45.45%
71 11aa, >antibacterial09922... at 45.45%
72 20aa, >antibacterial09926... at 35.00%
73 12aa, >antibacterial10000... at 41.67%
74 12aa, >antibacterial10005... at 50.00%
75 21aa, >antibacterial10049... at 42.86%
76 17aa, >antibacterial10177... at 41.18%
77 12aa, >antibacterial10234... at 41.67%
78 16aa, >antibacterial10258... at 43.75%
79 19aa, >antibacterial10347... *
80 19aa, >antibacterial10383... at 42.11%
81 13aa, >antibacterial10432... at 46.15%
82 14aa, >antibacterial10478... at 42.86%
83 22aa, >antibacterial10627... at 40.91%
84 13aa, >antibacterial10644... at 46.15%
85 13aa, >antibacterial10687... at 69.23%
86 18aa, >antibacterial10786... at 44.44%
87 20aa, >antibacterial10954... at 45.00%
88 15aa, >antibacterial11153... *
89 15aa, >antibacterial11670... at 35.00%
90 13aa, >antibacterial11957... at 46.15%
91 13aa, >antibacterial12313... at 46.15%
92 18aa, >antibacterial12444... at 44.44%
93 12aa, >antibacterial12465... at 50.00%
94 12aa, >antibacterial12472... at 41.67%
95 14aa, >antibacterial12525... at 64.29%
96 14aa, >antibacterial12526... at 64.29%
97 13aa, >antibacterial12553... at 46.15%
98 12aa, >antibacterial12587... *
99 21aa, >antibacterial12644... at 42.86%
100 15aa, >antibacterial12837... at 53.33%
101 12aa, >antibacterial12879... at 41.67%
102 18aa, >antibacterial12895... at 50.00%
103 15aa, >antibacterial12987... at 46.67%
104 20aa, >antibacterial13217... at 45.00%
105 14aa, >antibacterial13223... at 42.86%
106 20aa, >antibacterial13413... at 50.00%
>Cluster 21
0 255aa, >Antibacterial... *
1 13aa, >antimicrobial... at 46.15%
2 13aa, >antibacterial;antif... at 46.15%
3 11aa, >Antimicrobial... at 45.45%
4 26aa, >antimicrobial... at 46.15%

```
5     20aa, >Antibacterial;Antif... at 35.00%
6     21aa, >Anti-Gram-;antibact... at 42.86%
7     12aa, >antimicrobial... at 41.67%
8     19aa, >antibacterial;Antif... at 47.37%
9     18aa, >antimicrobial... at 40.00%
10    16aa, >anti-Gram+;Antibact... at 43.75%
11    255aa, >Antibacterial... at 43.92%
12    11aa, >antimicrobial... at 45.45%
13    12aa, >antimicrobial... at 50.00%
14    14aa, >anti-Gram+;Antibact... at 42.86%
15    14aa, >antibacterial;antim... at 42.86%
16    18aa, >antibacterial06631... at 44.44%
17    11aa, >antibacterial06681... at 45.45%
18    11aa, >antibacterial07732... at 45.45%
19    15aa, >antibacterial08039... at 35.00%
20    15aa, >antibacterial08197... *
21    16aa, >antibacterial08431... at 50.00%
22    24aa, >antibacterial08559... at 41.67%
23    17aa, >antibacterial08576... at 41.18%
24    15aa, >antibacterial08673... at 53.33%
25    15aa, >antibacterial08915... at 46.67%
26    12aa, >antibacterial09032... at 41.67%
27    12aa, >antibacterial09221... at 41.67%
28    20aa, >antibacterial09392... at 35.00%
29    11aa, >antibacterial09468... at 45.45%
30    12aa, >antibacterial10133... at 41.67%
31    14aa, >antibacterial10195... at 42.86%
32    14aa, >antibacterial10208... at 42.86%
33    16aa, >antibacterial10491... at 43.75%
34    11aa, >antibacterial10563... at 45.45%
35    11aa, >antibacterial10583... at 45.45%
36    14aa, >antibacterial10864... at 42.86%
37    12aa, >antibacterial11166... at 41.67%
38    14aa, >antibacterial11250... at 42.86%
39    14aa, >antibacterial11540... at 42.86%
40    15aa, >antibacterial11597... at 46.67%
41    15aa, >antibacterial11751... at 46.67%
42    13aa, >antibacterial11790... at 46.15%
43    14aa, >antibacterial11964... *
44    14aa, >antibacterial11967... at 42.86%
45    13aa, >antibacterial12311... at 46.15%
46    15aa, >antibacterial12363... at 35.00%
47    12aa, >antibacterial12464... at 41.67%
48    20aa, >antibacterial12504... at 35.00%
49    15aa, >antibacterial12948... at 35.00%
>Cluster 22
0     40aa, >anti-Gram+;antibact... *
1     36aa, >antimicrobial... at 44.44%
2     36aa, >antimicrobial... at 44.44%
3     36aa, >antimicrobial... at 41.67%
4     36aa, >antimicrobial... at 47.22%
5     36aa, >antimicrobial... at 50.00%
6     36aa, >antimicrobial... at 41.67%
7     45aa, >anti-Gram+;Antifung... at 48.89%
```

8	20aa, >Antifungal... at 35.00%
9	27aa, >Antimicrobial... at 40.74%
10	36aa, >Antifungal;antimicr... at 41.67%
11	22aa, >Antifungal... at 40.91%
12	41aa, >antibacterial;antif... *
13	40aa, >antifungal;antimicr... at 67.50%
14	255aa, >Antifungal... *
15	41aa, >antibacterial;antif... at 70.73%
16	43aa, >antifungal;antimicr... at 65.12%
17	45aa, >Antifungal;antimicr... at 64.44%
18	41aa, >antibacterial;antif... at 68.29%
19	255aa, >Antifungal... at 70.20%
20	17aa, >Antimicrobial... at 41.18%
21	15aa, >Antimicrobial... at 35.00%
22	33aa, >antimicrobial... at 45.45%
23	33aa, >antimicrobial... at 48.48%
24	29aa, >antimicrobial... at 41.38%
25	25aa, >anti-Gram+;antibact... at 35.00%
26	23aa, >anti-Gram+;antimicr... at 43.48%
27	30aa, >antimicrobial... at 43.33%
28	29aa, >antimicrobial... at 44.83%
29	30aa, >anti-HIV;Antimicrob... at 43.33%
30	30aa, >Antimicrobial;Antim... at 46.67%
31	29aa, >antimicrobial... at 41.38%
32	29aa, >Antimicrobial... at 41.38%
33	30aa, >Antimicrobial;Antim... at 35.00%
34	30aa, >antimicrobial... at 43.33%
35	29aa, >Antimicrobial... at 41.38%
36	29aa, >Antimicrobial;Antim... at 41.38%
37	35aa, >Antimicrobial... at 35.00%
38	44aa, >antifungal;antimicr... at 59.09%
39	36aa, >antimicrobial... at 47.22%
40	36aa, >antimicrobial... at 41.67%
41	41aa, >antifungal;antimicr... at 46.34%
42	44aa, >antifungal;antimicr... *
43	40aa, >antibacterial;antif... at 47.50%
44	31aa, >Antimicrobial... at 41.94%
45	14aa, >antimicrobial... at 50.00%
46	16aa, >antimicrobial... at 43.75%
47	41aa, >antifungal;antimicr... at 63.41%
48	40aa, >Antibacterial;Antif... at 42.50%
49	42aa, >Antimicrobial... at 54.76%
50	255aa, >Antifungal... at 61.96%
51	45aa, >Antifungal... at 60.00%
52	36aa, >antimicrobial... at 52.78%
53	17aa, >antimicrobial... at 41.18%
54	243aa, >Antifungal... at 62.14%
55	29aa, >Antimicrobial... at 41.38%
56	29aa, >antimicrobial... at 41.38%
57	35aa, >antifungal;antimicr... at 42.86%
58	11aa, >Antibacterial... at 45.45%
59	37aa, >antifungal;antimicr... at 45.95%
60	16aa, >antibacterial06495... at 43.75%
61	29aa, >antibacterial06979... at 31.38%

62   41aa, >antibacterial07314... *
63   17aa, >antibacterial07694... at 41.18%
64   17aa, >antibacterial08492... at 41.18%
65   13aa, >antibacterial08724... at 46.15%
66   18aa, >antibacterial09006... at 44.44%
67   20aa, >antibacterial09086... at 50.00%
68   11aa, >antibacterial09170... at 45.45%
69   20aa, >antibacterial09442... at 35.00%
70   14aa, >antibacterial09631... at 42.86%
71   18aa, >antibacterial09848... at 50.00%
72   15aa, >antibacterial09923... at 35.00%
73   12aa, >antibacterial10011... at 41.67%
74   12aa, >antibacterial10012... at 41.67%
75   29aa, >antibacterial10782... at 41.38%
76   20aa, >antibacterial10922... at 35.00%
77   11aa, >antibacterial11126... at 45.45%
78   15aa, >antibacterial11277... at 35.00%
79   15aa, >antibacterial11352... at 35.00%
80   23aa, >antibacterial11390... at 43.48%
81   15aa, >antibacterial11433... at 35.00%
82   15aa, >antibacterial11576... at 46.67%
83   16aa, >antibacterial11637... at 56.25%
84   12aa, >antibacterial11663... at 41.67%
85   14aa, >antibacterial11692... at 42.86%
86   14aa, >antibacterial11694... at 50.00%
87   20aa, >antibacterial11718... at 35.00%
88   13aa, >antibacterial12034... at 46.15%
89   13aa, >antibacterial12046... at 46.15%
90   13aa, >antibacterial12052... at 46.15%
91   13aa, >antibacterial12260... at 46.15%
92   13aa, >antibacterial12261... at 46.15%
93   13aa, >antibacterial12270... at 46.15%
94   29aa, >antibacterial12729... at 41.38%
95   29aa, >antibacterial12730... at 44.83%
96   29aa, >antibacterial12800... at 41.38%
97   12aa, >antibacterial13116... at 41.67%
98   15aa, >antibacterial13178... at 46.67%
99   12aa, >antibacterial13195... at 41.67%
100   16aa, >antibacterial13310... at 43.75%
>Cluster 23
0   72aa, >antithrombotic... at 47.22%
1   255aa, >Antibacterial... *
2   24aa, >antibacterial;antif... at 41.67%
3   12aa, >antibacterial;antif... at 41.67%
4   22aa, >antibacterial;antim... at 40.91%
5   22aa, >antimicrobial... at 40.91%
6   68aa, >antithrombotic... at 47.06%
7   17aa, >anti-Gram+;Antibact... at 58.82%
8   30aa, >antimicrobial... at 35.00%
9   37aa, >Antibacterial;Gram-... at 43.24%
10   21aa, >antibacterial;antim... at 42.86%
11   12aa, >Antifungal... at 41.67%
12   12aa, >Antifungal... at 41.67%
13   13aa, >antimicrobial... *

14 84aa, >antithrombotic... at 40.48%
15 15aa, >Antibacterial;Antif... at 46.67%
16 13aa, >antibacterial07262... at 46.15%
17 11aa, >antibacterial07390... at 45.45%
18 15aa, >antibacterial07594... at 35.00%
19 21aa, >antibacterial07595... at 42.86%
20 13aa, >antibacterial07671... at 51.54%
21 17aa, >antibacterial07698... at 41.18%
22 14aa, >antibacterial07889... at 50.00%
23 14aa, >antibacterial07897... at 42.86%
24 13aa, >antibacterial08173... at 46.15%
25 22aa, >antibacterial08238... *
26 11aa, >antibacterial08310... at 45.45%
27 13aa, >antibacterial08523... at 46.15%
28 15aa, >antibacterial08633... at 35.00%
29 12aa, >antibacterial08787... at 50.00%
30 12aa, >antibacterial09019... at 41.67%
31 12aa, >antibacterial09030... at 41.67%
32 13aa, >antibacterial09231... at 46.15%
33 12aa, >antibacterial09239... at 41.67%
34 11aa, >antibacterial09356... at 45.45%
35 20aa, >antibacterial09448... at 35.00%
36 23aa, >antibacterial09507... at 43.48%
37 11aa, >antibacterial09623... at 45.45%
38 13aa, >antibacterial09730... at 46.15%
39 12aa, >antibacterial09991... at 41.67%
40 15aa, >antibacterial10072... at 35.00%
41 11aa, >antibacterial10184... *
42 11aa, >antibacterial10185... at 54.55%
43 12aa, >antibacterial10240... at 41.67%
44 11aa, >antibacterial10661... at 45.45%
45 12aa, >antibacterial11172... at 41.67%
46 14aa, >antibacterial11484... at 42.86%
47 11aa, >antibacterial11601... at 54.55%
48 18aa, >antibacterial11614... at 44.44%
49 18aa, >antibacterial12092... at 50.00%
50 12aa, >antibacterial12462... at 41.67%
51 18aa, >antibacterial12552... at 50.00%
52 18aa, >antibacterial12673... at 50.00%
53 20aa, >antibacterial12725... at 35.00%
54 22aa, >antibacterial12769... at 40.91%
55 15aa, >antibacterial12947... at 35.00%
56 12aa, >antibacterial12983... at 41.67%
57 15aa, >antibacterial13046... at 35.00%
58 12aa, >antibacterial13082... at 41.67%
59 13aa, >antibacterial13326... at 46.15%
60 12aa, >antibacterial13409... at 41.67%
>Cluster 24
0 218aa, >Antimicrobial... at 53.67%
1 201aa, >Antifungal... *
2 148aa, >Antifungal... at 47.30%
3 40aa, >Antifungal... at 57.50%
4 148aa, >Antifungal... at 52.03%
5 13aa, >antimicrobial... *

6	13aa, >antimicrobial... at 61.54%
7	13aa, >antimicrobial... at 61.54%
8	11aa, >Antibacterial;Antif... at 45.45%
9	13aa, >anti-Gram+;Antibact... at 53.85%
10	13aa, >Antifungal;Antimicr... at 46.15%
11	15aa, >antimicrobial... at 46.67%
12	30aa, >Antimicrobial;Antim... at 43.33%
13	30aa, >Antimicrobial;Antim... at 35.00%
14	30aa, >antimicrobial... at 43.33%
15	25aa, >anti-Gram+;anti-HIV... at 35.00%
16	17aa, >antimicrobial... at 41.18%
17	11aa, >antimicrobial... at 45.45%
18	13aa, >Antimicrobial... at 46.15%
19	16aa, >antibacterial;antim... at 43.75%
20	176aa, >Antimicrobial... at 53.41%
21	173aa, >Antimicrobial... at 46.82%
22	171aa, >Antimicrobial... at 47.95%
23	173aa, >Antimicrobial... at 46.82%
24	255aa, >Antimicrobial... *
25	255aa, >Antimicrobial... at 60.39%
26	255aa, >Antimicrobial... at 35.00%
27	246aa, >Antimicrobial... at 51.22%
28	255aa, >Antimicrobial... at 41.57%
29	223aa, >Antimicrobial... at 55.61%
30	246aa, >Antimicrobial... at 49.59%
31	173aa, >Antimicrobial... at 47.98%
32	179aa, >Antimicrobial... at 49.72%
33	180aa, >Antimicrobial... at 48.89%
34	178aa, >Antimicrobial... at 52.81%
35	183aa, >Antimicrobial... at 41.53%
36	177aa, >Antimicrobial... at 48.59%
37	245aa, >Antimicrobial... at 41.22%
38	229aa, >Antimicrobial... *
39	173aa, >Antimicrobial... at 46.24%
40	173aa, >Antimicrobial... at 47.98%
41	224aa, >Antimicrobial... at 47.77%
42	246aa, >Antimicrobial... at 48.37%
43	217aa, >Antimicrobial... at 43.78%
44	247aa, >Antimicrobial... at 49.80%
45	183aa, >Antimicrobial... at 40.98%
46	226aa, >Antimicrobial... at 53.98%
47	250aa, >Antimicrobial... at 45.20%
48	225aa, >Antimicrobial... at 54.67%
49	225aa, >Antimicrobial... at 57.33%
50	225aa, >Antimicrobial... at 58.67%
51	246aa, >Antimicrobial... at 48.78%
52	247aa, >Antimicrobial... at 46.56%
53	177aa, >Antimicrobial... at 46.33%
54	228aa, >Antimicrobial... at 42.11%
55	224aa, >Antimicrobial... at 58.04%
56	154aa, >Antimicrobial... at 51.95%
57	225aa, >Antimicrobial... at 52.89%
58	226aa, >Antimicrobial... at 51.33%
59	226aa, >Antimicrobial... at 51.33%

60	225aa, >Antimicrobial... at 55.56%
61	210aa, >Antimicrobial... at 45.71%
62	246aa, >Antimicrobial... at 43.09%
63	229aa, >Antimicrobial... at 45.85%
64	12aa, >antimicrobial... at 41.67%
65	225aa, >Antimicrobial... at 54.22%
66	244aa, >Antimicrobial... at 40.16%
67	233aa, >Antimicrobial... at 46.35%
68	251aa, >Antimicrobial... at 47.01%
69	251aa, >Antimicrobial... at 45.42%
70	250aa, >Antimicrobial... at 46.40%
71	229aa, >Antimicrobial... at 52.84%
72	175aa, >Antimicrobial... at 43.43%
73	225aa, >Antimicrobial... at 54.67%
74	226aa, >Antimicrobial... at 53.10%
75	230aa, >Antimicrobial... at 58.70%
76	251aa, >Antimicrobial... at 46.22%
77	225aa, >Antimicrobial... at 56.00%
78	224aa, >Antimicrobial... at 51.34%
79	220aa, >Antimicrobial... at 44.09%
80	226aa, >Antimicrobial... at 50.88%
81	225aa, >Antimicrobial... at 47.11%
82	222aa, >Antimicrobial... at 58.11%
83	255aa, >Antimicrobial... at 62.75%
84	172aa, >Antimicrobial... at 43.60%
85	240aa, >Antimicrobial... at 52.08%
86	15aa, >antimicrobial... at 35.00%
87	177aa, >Antimicrobial... at 52.54%
88	27aa, >antifungal;antimicr... at 48.15%
89	12aa, >antibacterial;antif... at 41.67%
90	17aa, >Antimicrobial... at 41.18%
91	14aa, >Antimicrobial... at 42.86%
92	15aa, >Antimicrobial... at 35.00%
93	30aa, >Antifungal... at 35.00%
94	15aa, >anti-Gram-;antibact... at 35.00%
95	180aa, >Antimicrobial... at 47.78%
96	30aa, >Antimicrobial... at 35.00%
97	198aa, >Antimicrobial... at 47.47%
98	22aa, >antibacterial07389... at 45.45%
99	16aa, >L02A001747... at 43.75%
100	26aa, >antibacterial07787... at 42.31%
101	20aa, >antibacterial07972... at 35.00%
102	17aa, >antibacterial08247... at 41.18%
103	14aa, >antibacterial08448... at 50.00%
104	12aa, >antibacterial08990... at 41.67%
105	13aa, >antibacterial09768... at 46.15%
106	19aa, >antibacterial09927... at 42.11%
107	15aa, >antibacterial09989... at 35.00%
108	13aa, >antibacterial10118... at 46.15%
109	24aa, >antibacterial10709... at 41.67%
110	31aa, >antibacterial10858... at 41.94%
111	15aa, >antibacterial11150... at 35.00%
112	18aa, >antibacterial11160... at 44.44%
113	15aa, >antibacterial11163... at 35.00%

```
114   17aa, >antibacterial11190... at 41.18%
115   14aa, >antibacterial11214... at 42.86%
116   15aa, >antibacterial11244... at 35.00%
117   18aa, >antibacterial11264... at 44.44%
118   15aa, >antibacterial11537... at 46.67%
119   12aa, >antibacterial11594... at 41.67%
120   20aa, >antibacterial11618... at 45.00%
121   15aa, >antibacterial11625... at 35.00%
122   20aa, >antibacterial11706... *
123   20aa, >antibacterial11769... at 35.00%
124   20aa, >antibacterial11825... at 50.00%
125   28aa, >antibacterial11853... at 46.43%
126   16aa, >antibacterial11858... at 43.75%
127   17aa, >antibacterial11929... at 41.18%
128   12aa, >antibacterial12447... at 41.67%
129   13aa, >antibacterial12864... at 46.15%
130   20aa, >antibacterial12880... at 35.00%
131   18aa, >antibacterial12894... at 44.44%
132   13aa, >antibacterial12901... at 46.15%
133   20aa, >antibacterial12910... at 35.00%
134   20aa, >antibacterial12954... at 35.00%
135   20aa, >antibacterial12990... at 45.00%
136   15aa, >antibacterial13045... at 46.67%
137   15aa, >antibacterial13059... at 35.00%
138   15aa, >antibacterial13226... at 46.67%
>Cluster 25
0     15aa, >Antifungal... at 35.00%
1     12aa, >antimicrobial... at 41.67%
2     13aa, >antimicrobial... at 46.15%
3     13aa, >antimicrobial... at 46.15%
4     27aa, >Antimicrobial... at 40.74%
5     24aa, >antimicrobial... at 41.67%
6     19aa, >antimicrobial... at 47.37%
7     29aa, >antibacterial;antim... at 41.38%
8     22aa, >antimicrobial... at 40.91%
9     14aa, >antibacterial... at 50.00%
10    21aa, >antimicrobial... at 47.62%
11    29aa, >antibacterial;antif... at 41.38%
12    26aa, >anti-Gram-... at 42.31%
13    15aa, >Antibacterial... at 35.00%
14    14aa, >anti-Gram+;antibact... at 42.86%
15    11aa, >Antibacterial;Antif... at 54.55%
16    255aa, >Antimicrobial... *
17    20aa, >antimicrobial... at 35.00%
18    14aa, >antibacterial... at 42.86%
19    12aa, >antibacterial06986... at 41.67%
20    20aa, >antibacterial07099... at 35.00%
21    11aa, >antibacterial07736... at 54.55%
22    18aa, >antibacterial07780... at 44.44%
23    13aa, >antibacterial08524... at 46.15%
24    19aa, >antibacterial08660... at 42.11%
25    13aa, >antibacterial08706... at 46.15%
26    13aa, >antibacterial08723... at 46.15%
27    13aa, >antibacterial08725... at 46.15%
```

28   15aa, >antibacterial08808... at 35.00%
29   15aa, >antibacterial08816... at 46.67%
30   14aa, >antibacterial08852... at 50.00%
31   13aa, >antibacterial08860... at 46.15%
32   12aa, >antibacterial08932... at 41.67%
33   22aa, >antibacterial09044... at 45.45%
34   14aa, >antibacterial09075... at 42.86%
35   14aa, >antibacterial09079... at 42.86%
36   13aa, >antibacterial09133... at 46.15%
37   13aa, >antibacterial09134... at 46.15%
38   13aa, >antibacterial09141... *
39   13aa, >antibacterial09142... at 53.85%
40   14aa, >antibacterial09630... at 42.86%
41   11aa, >antibacterial09823... at 45.45%
42   12aa, >antibacterial10014... at 41.67%
43   18aa, >antibacterial10209... *
44   26aa, >antibacterial10331... at 50.00%
45   15aa, >antibacterial10386... at 35.00%
46   26aa, >antibacterial10387... at 42.31%
47   12aa, >antibacterial10535... at 50.00%
48   14aa, >antibacterial10789... at 42.86%
49   56aa, >antibacterial10799... at 41.07%
50   16aa, >antibacterial10913... at 43.75%
51   20aa, >antibacterial11302... at 35.00%
52   15aa, >antibacterial11367... at 35.00%
53   36aa, >antibacterial11535... at 52.78%
54   15aa, >antibacterial11719... at 46.67%
55   35aa, >antibacterial12006... at 35.00%
56   13aa, >antibacterial12233... at 46.15%
57   36aa, >antibacterial12346... at 44.44%
58   26aa, >antibacterial12433... at 42.31%
59   12aa, >antibacterial12574... at 41.67%
60   12aa, >antibacterial12583... at 41.67%
61   42aa, >antibacterial12678... at 40.48%
62   15aa, >antibacterial12741... at 46.67%
63   18aa, >antibacterial12829... at 44.44%
64   14aa, >antibacterial13051... at 42.86%
65   32aa, >antibacterial13263... at 40.62%
66   20aa, >antibacterial13269... at 35.00%
67   35aa, >antibacterial13320... at 35.00%
68   37aa, >antibacterial13334... at 43.24%
69   36aa, >antibacterial13335... at 41.67%
70   36aa, >antibacterial13337... at 44.44%
71   15aa, >antibacterial13391... at 46.67%
>Cluster 26
0   22aa, >Antimicrobial... at 40.91%
1   13aa, >antibacterial;Antif... *
2   19aa, >Antifungal;antimicr... at 47.37%
3   14aa, >antimicrobial... at 42.86%
4   16aa, >antimicrobial... at 43.75%
5   31aa, >Antibacterial;Antif... at 41.94%
6   27aa, >anti-Gram+;antibact... at 40.74%
7   13aa, >Antibacterial;antim... at 46.15%
8   13aa, >Antimicrobial... at 46.15%

```
9     13aa, >antimicrobial... at 53.85%
10    255aa, >Antimicrobial... *
11    255aa, >Antimicrobial... at 71.76%
12    20aa, >antibacterial... at 45.00%
13    15aa, >antifungal;antimicr... at 46.67%
14    32aa, >Antimicrobial... at 40.62%
15    32aa, >antibacterial;antim... at 40.62%
16    20aa, >antibacterial06447... at 35.00%
17    14aa, >antibacterial07047... at 42.86%
18    14aa, >antibacterial07048... at 42.86%
19    30aa, >antibacterial07499... at 35.00%
20    28aa, >antibacterial07996... at 42.86%
21    13aa, >antibacterial08265... at 46.15%
22    17aa, >antibacterial08272... at 41.18%
23    13aa, >antibacterial08377... at 46.15%
24    26aa, >antibacterial08420... at 46.15%
25    23aa, >antibacterial08563... at 43.48%
26    19aa, >antibacterial08568... at 42.11%
27    15aa, >antibacterial08689... at 35.00%
28    17aa, >antibacterial08858... at 41.18%
29    16aa, >antibacterial08865... at 43.75%
30    18aa, >antibacterial08890... at 44.44%
31    12aa, >antibacterial08956... at 41.67%
32    12aa, >antibacterial08957... at 41.67%
33    12aa, >antibacterial08961... at 41.67%
34    20aa, >antibacterial09376... at 35.00%
35    12aa, >antibacterial09957... at 41.67%
36    13aa, >antibacterial10114... at 46.15%
37    14aa, >antibacterial10793... at 42.86%
38    15aa, >antibacterial11258... at 35.00%
39    20aa, >antibacterial11324... at 35.00%
40    20aa, >antibacterial11435... at 35.00%
41    15aa, >antibacterial11490... at 35.00%
42    27aa, >antibacterial11520... at 40.74%
43    20aa, >antibacterial11844... at 35.00%
44    25aa, >antibacterial11946... at 35.00%
45    17aa, >antibacterial12557... *
46    15aa, >antibacterial12858... at 35.00%
47    11aa, >antibacterial12925... at 45.45%
>Cluster 27
0     17aa, >antimicrobial... at 41.18%
1     16aa, >anti-Gram+;antibact... at 43.75%
2     30aa, >antimicrobial... at 35.00%
3     19aa, >Antimicrobial... at 42.11%
4     26aa, >antimicrobial... at 42.31%
5     14aa, >antimicrobial... at 42.86%
6     14aa, >antimicrobial... at 42.86%
7     17aa, >antimicrobial... *
8     13aa, >Antibacterial;antim... at 46.15%
9     24aa, >antimicrobial... at 41.67%
10    19aa, >anti-Gram+;antifung... at 42.11%
11    19aa, >anti-Gram+;antibact... at 47.37%
12    16aa, >antimicrobial... at 43.75%
13    16aa, >Antibacterial;Antif... at 43.75%
```

14 22aa, >antibacterial;antim... at 40.91%
15 21aa, >antimicrobial... at 42.86%
16 12aa, >Antifungal;Antimicr... at 41.67%
17 12aa, >Antifungal;Antimicr... at 41.67%
18 30aa, >antimicrobial... at 35.00%
19 14aa, >antibacterial... at 50.00%
20 11aa, >Antimicrobial... at 45.45%
21 255aa, >Antimicrobial... *
22 255aa, >Antimicrobial... at 54.51%
23 156aa, >Antimicrobial... at 51.28%
24 13aa, >antimicrobial... at 46.15%
25 13aa, >Antibacterial... at 46.15%
26 12aa, >Anti-Gram-... at 41.67%
27 13aa, >antibacterial06625... at 46.15%
28 21aa, >antibacterial06683... at 42.86%
29 12aa, >antibacterial07006... at 41.67%
30 14aa, >antibacterial07019... at 42.86%
31 12aa, >antibacterial07026... at 41.67%
32 13aa, >antibacterial07312... at 46.15%
33 20aa, >antibacterial07328... at 35.00%
34 18aa, >antibacterial07434... *
35 18aa, >antibacterial07435... at 50.00%
36 17aa, >antibacterial07555... at 41.18%
37 13aa, >antibacterial07911... at 46.15%
38 13aa, >antibacterial08147... at 46.15%
39 13aa, >antibacterial08153... at 53.85%
40 13aa, >antibacterial08156... at 46.15%
41 13aa, >antibacterial08159... at 53.85%
42 13aa, >antibacterial08160... at 53.85%
43 13aa, >antibacterial08166... at 46.15%
44 13aa, >antibacterial08178... at 53.85%
45 13aa, >antibacterial08180... at 46.15%
46 17aa, >antibacterial08248... at 41.18%
47 13aa, >antibacterial08312... at 46.15%
48 18aa, >antibacterial08596... at 44.44%
49 12aa, >antibacterial08942... at 50.00%
50 20aa, >antibacterial08958... at 35.00%
51 14aa, >antibacterial08998... at 50.00%
52 15aa, >antibacterial09014... at 35.00%
53 20aa, >antibacterial09997... at 35.00%
54 18aa, >antibacterial10055... at 44.44%
55 12aa, >antibacterial10217... at 41.67%
56 13aa, >antibacterial10482... at 46.15%
57 12aa, >antibacterial10489... at 41.67%
58 11aa, >antibacterial10551... at 45.45%
59 13aa, >antibacterial10763... at 46.15%
60 16aa, >antibacterial10857... at 43.75%
61 12aa, >antibacterial11174... at 41.67%
62 13aa, >antibacterial11175... *
63 18aa, >antibacterial11177... at 44.44%
64 14aa, >antibacterial11235... at 50.00%
65 18aa, >antibacterial11580... at 50.00%
66 16aa, >antibacterial11860... at 50.00%
67 18aa, >antibacterial12556... at 44.44%

68 20aa, >antibacterial12697... at 35.00%
69 18aa, >antibacterial12712... at 44.44%
70 15aa, >antibacterial12998... at 35.00%
71 15aa, >antibacterial13014... at 35.00%
72 20aa, >antibacterial13062... at 45.00%
73 20aa, >antibacterial13146... at 35.00%
74 20aa, >antibacterial13262... at 35.00%
75 20aa, >antibacterial13381... at 35.00%
>Cluster 28
0 13aa, >antimicrobial... at 46.15%
1 13aa, >antimicrobial... *
2 30aa, >Antimicrobial... at 43.33%
3 17aa, >antimicrobial... at 41.18%
4 11aa, >Antibacterial... at 45.45%
5 19aa, >Antimicrobial... at 42.11%
6 17aa, >Antimicrobial... at 41.18%
7 17aa, >antimicrobial... at 41.18%
8 15aa, >antimicrobial... at 35.00%
9 13aa, >antimicrobial... at 46.15%
10 13aa, >anti-Gram+;antibact... at 53.85%
11 20aa, >Antibacterial;Antif... at 35.00%
12 15aa, >antimicrobial... *
13 25aa, >antibacterial... at 44.00%
14 22aa, >anti-Gram+;antibact... at 40.91%
15 19aa, >anti-Gram+;antimicr... at 42.11%
16 13aa, >anti-Gram+;antibact... at 53.85%
17 25aa, >anti-Gram+;Antibact... at 35.00%
18 255aa, >Antimicrobial... *
19 13aa, >antimicrobial... at 53.85%
20 11aa, >Antibacterial;Antim... at 45.45%
21 16aa, >antibacterial;antim... at 43.75%
22 12aa, >antibacterial06989... at 41.67%
23 11aa, >antibacterial07660... at 54.55%
24 15aa, >antibacterial07765... at 35.00%
25 12aa, >antibacterial07801... at 41.67%
26 19aa, >antibacterial08059... at 42.11%
27 17aa, >antibacterial08065... at 41.18%
28 23aa, >antibacterial08094... at 43.48%
29 14aa, >antibacterial08408... at 50.00%
30 17aa, >antibacterial08455... at 47.06%
31 14aa, >antibacterial08709... at 42.86%
32 20aa, >antibacterial08919... at 35.00%
33 11aa, >antibacterial09343... at 45.45%
34 20aa, >antibacterial09360... at 45.00%
35 20aa, >antibacterial09420... at 35.00%
36 20aa, >antibacterial09432... at 45.00%
37 20aa, >antibacterial09439... at 35.00%
38 13aa, >antibacterial09589... at 53.85%
39 12aa, >antibacterial09811... at 41.67%
40 11aa, >antibacterial10509... at 45.45%
41 15aa, >antibacterial10570... at 53.33%
42 19aa, >antibacterial10602... at 42.11%
43 11aa, >antibacterial10674... at 45.45%
44 12aa, >antibacterial11179... at 41.67%

```
45    14aa, >antibacterial11249... at 42.86%
46    15aa, >antibacterial11261... at 35.00%
47    16aa, >antibacterial11275... at 43.75%
48    29aa, >antibacterial11382... at 41.38%
49    15aa, >antibacterial11760... at 35.00%
50    15aa, >antibacterial11840... at 35.00%
51    15aa, >antibacterial11953... at 46.67%
52    14aa, >antibacterial12005... at 42.86%
53    13aa, >antibacterial12202... at 46.15%
54    13aa, >antibacterial12214... at 46.15%
55    13aa, >antibacterial12218... at 46.15%
56    17aa, >antibacterial12385... *
57    12aa, >antibacterial12584... at 41.67%
58    12aa, >antibacterial12589... at 41.67%
59    12aa, >antibacterial12604... at 41.67%
60    14aa, >antibacterial12606... at 42.86%
61    13aa, >antibacterial12821... at 36.15%
62    13aa, >antibacterial12979... at 36.15%
63    20aa, >antibacterial13161... at 45.00%
>Cluster 29
0     15aa, >Antifungal;Antivira... *
1     30aa, >antimicrobial;antiv... at 35.00%
2     30aa, >antimicrobial;antiv... at 43.33%
3     34aa, >Antifungal;antimicr... at 50.00%
4     13aa, >Antibacterial... at 46.15%
5     27aa, >antimicrobial... at 44.44%
6     24aa, >Antimicrobial... at 45.83%
7     21aa, >antibacterial;Antif... at 42.86%
8     21aa, >antibacterial;antif... at 42.86%
9     29aa, >antimicrobial... at 41.38%
10    13aa, >antimicrobial;Antiv... at 46.15%
11    11aa, >Antimicrobial... at 45.45%
12    13aa, >anti-Gram+;antimicr... at 46.15%
13    11aa, >Antibacterial;Antim... at 45.45%
14    255aa, >Antimicrobial... *
15    236aa, >Antimicrobial... at 46.19%
16    255aa, >Antimicrobial... at 41.57%
17    255aa, >Antimicrobial... at 41.18%
18    245aa, >Antimicrobial... at 42.04%
19    247aa, >Antimicrobial... at 43.32%
20    255aa, >Antimicrobial... at 43.53%
21    255aa, >Antimicrobial... at 40.39%
22    245aa, >Antimicrobial... at 44.08%
23    245aa, >Antimicrobial... at 43.67%
24    255aa, >Antimicrobial... at 41.96%
25    255aa, >Antimicrobial... at 43.53%
26    245aa, >Antimicrobial... at 44.08%
27    255aa, >Antimicrobial... at 55.29%
28    11aa, >antithrombotic... at 45.45%
29    17aa, >anti-Gram-;antibact... at 41.18%
30    29aa, >antimicrobial... at 41.38%
31    14aa, >Antibacterial... at 42.86%
32    20aa, >antibacterial06492... at 35.00%
33    30aa, >L02A001785... *
```

```
34    12aa, >antibacterial07029... at 41.67%
35    18aa, >antibacterial07811... at 44.44%
36    30aa, >antibacterial07846... at 35.00%
37    24aa, >antibacterial08035... at 45.83%
38    22aa, >antibacterial08036... at 40.91%
39    19aa, >antibacterial08049... at 42.11%
40    13aa, >antibacterial08163... at 46.15%
41    13aa, >antibacterial08190... at 46.15%
42    13aa, >antibacterial08191... at 46.15%
43    15aa, >antibacterial08685... at 35.00%
44    12aa, >antibacterial08920... at 41.67%
45    20aa, >antibacterial09087... at 45.00%
46    20aa, >antibacterial09092... at 45.00%
47    20aa, >antibacterial09097... at 35.00%
48    19aa, >antibacterial09672... at 42.11%
49    13aa, >antibacterial10210... at 46.15%
50    29aa, >antibacterial10737... at 41.38%
51    19aa, >antibacterial10900... at 42.11%
52    15aa, >antibacterial11256... at 35.00%
53    30aa, >antibacterial11414... at 43.33%
54    14aa, >antibacterial11486... at 42.86%
55    19aa, >antibacterial11659... at 42.11%
56    24aa, >antibacterial11679... at 45.83%
57    16aa, >antibacterial11683... at 50.00%
58    17aa, >antibacterial11755... at 47.06%
59    20aa, >antibacterial11911... at 45.00%
60    22aa, >antibacterial12117... *
61    13aa, >antibacterial12252... at 46.15%
62    26aa, >antibacterial12362... at 46.15%
63    20aa, >antibacterial12394... at 35.00%
64    13aa, >antibacterial12512... at 53.85%
65    20aa, >antibacterial12695... at 45.00%
66    15aa, >antibacterial13040... at 53.33%
67    12aa, >antibacterial13049... at 41.67%
68    20aa, >antibacterial13163... at 35.00%
69    15aa, >antibacterial13213... at 35.00%
>Cluster 30
0     24aa, >Antibacterial... at 41.67%
1     19aa, >Antibacterial... *
2     17aa, >antibacterial;antim... at 41.18%
3     18aa, >antibacterial;antim... at 44.44%
4     12aa, >Antibacterial... at 41.67%
5     21aa, >antibacterial;Antif... at 42.86%
6     27aa, >antimicrobial.... at 40.74%
7     23aa, >Antimicrobial... *
8     255aa, >Antimicrobial... *
9     11aa, >antibacterial07733... at 45.45%
10    16aa, >antibacterial07739... at 43.75%
11    18aa, >antibacterial08056... at 44.44%
12    11aa, >antibacterial08227... at 45.45%
13    11aa, >antibacterial08296... at 45.45%
14    14aa, >antibacterial08641... at 50.00%
15    11aa, >antibacterial09181... at 45.45%
16    25aa, >antibacterial09345... at 35.00%
```

```
17    14aa, >antibacterial09467... at 42.86%
18    18aa, >antibacterial10417... at 44.44%
19    16aa, >antibacterial10492... at 43.75%
20    13aa, >antibacterial11271... *
21    15aa, >antibacterial11947... at 35.00%
22    16aa, >antibacterial12414... at 43.75%
23    34aa, >antibacterial12672... at 41.18%
24    15aa, >antibacterial12824... at 35.00%
25    14aa, >antibacterial13076... at 42.86%
26    25aa, >antibacterial13325... at 35.00%
>Cluster 31
0     17aa, >anti-Gram-... at 47.06%
1     24aa, >Antibacterial;Antif... at 41.67%
2     11aa, >Antimicrobial;Antim... *
3     24aa, >antimicrobial... at 45.83%
4     24aa, >antibacterial;Antif... at 41.67%
5     24aa, >antifungal;antimicr... at 41.67%
6     24aa, >anti-Gram+;Antibact... at 41.67%
7     11aa, >Antimicrobial;Antim... at 45.45%
8     25aa, >Antibacterial;antim... at 35.00%
9     18aa, >antimicrobial... at 44.44%
10    16aa, >anti-Gram-;antibact... at 50.00%
11    255aa, >Antibacterial... *
12    255aa, >Antimicrobial... at 72.94%
13    255aa, >Antimicrobial... at 41.96%
14    255aa, >Antimicrobial... at 75.29%
15    255aa, >Antimicrobial... at 56.08%
16    255aa, >Antimicrobial... at 49.41%
17    255aa, >Antimicrobial... at 43.14%
18    255aa, >Antimicrobial... at 54.12%
19    255aa, >Antimicrobial... at 51.37%
20    19aa, >antimicrobial... at 42.11%
21    20aa, >anti-Gram+;antibact... at 35.00%
22    16aa, >antimicrobial... at 43.75%
23    11aa, >antibacterial;antim... at 45.45%
24    14aa, >antimicrobial... at 42.86%
25    15aa, >antimicrobial... at 35.00%
26    13aa, >antimicrobial... at 46.15%
27    13aa, >antibacterial08148... *
28    13aa, >antibacterial08155... at 53.85%
29    13aa, >antibacterial08157... at 53.85%
30    13aa, >antibacterial08179... at 46.15%
31    15aa, >antibacterial08827... at 35.00%
32    18aa, >antibacterial09007... at 44.44%
33    18aa, >antibacterial09008... at 44.44%
34    14aa, >antibacterial09078... at 42.86%
35    17aa, >antibacterial09172... at 41.18%
36    20aa, >antibacterial09409... at 35.00%
37    20aa, >antibacterial09415... at 35.00%
38    16aa, >antibacterial09497... at 43.75%
39    14aa, >antibacterial09638... at 50.00%
40    15aa, >antibacterial09681... at 46.67%
41    11aa, >antibacterial09817... at 45.45%
42    15aa, >antibacterial10153... at 35.00%
```

```
43    15aa, >antibacterial10158... at 46.67%
44    15aa, >antibacterial10160... at 35.00%
45    15aa, >antibacterial10162... at 35.00%
46    15aa, >antibacterial10163... at 35.00%
47    15aa, >antibacterial10169... at 46.67%
48    15aa, >antibacterial10174... at 35.00%
49    14aa, >antibacterial10639... at 42.86%
50    11aa, >antibacterial11148... at 45.45%
51    15aa, >antibacterial11473... *
52    21aa, >antibacterial11514... at 42.86%
53    12aa, >antibacterial11527... at 50.00%
54    17aa, >antibacterial11651... at 41.18%
55    19aa, >antibacterial12412... at 42.11%
56    15aa, >antibacterial12611... at 35.00%
57    12aa, >antibacterial13410... at 50.00%
>Cluster 32
0     23aa, >antifungal;antimicr... at 43.48%
1     11aa, >antibacterial... *
2     13aa, >anti-Gram+;antibact... at 46.15%
3     21aa, >antimicrobial... at 42.86%
4     11aa, >Antibacterial;Antif... at 44.55%
5     24aa, >anti-Gram-;antibact... at 41.67%
6     21aa, >Antimicrobial... at 47.62%
7     255aa, >Antibacterial... *
8     17aa, >antimicrobial... at 41.18%
9     18aa, >antibacterial;Antif... at 44.44%
10    20aa, >anti-Gram+;antibact... at 35.00%
11    16aa, >antibacterial... at 43.75%
12    13aa, >antifungal;antimicr... at 46.15%
13    13aa, >antibacterial06479... at 46.15%
14    32aa, >antibacterial06795... at 40.62%
15    11aa, >antibacterial07696... at 45.45%
16    15aa, >antibacterial07716... at 35.00%
17    15aa, >antibacterial07717... at 35.00%
18    15aa, >antibacterial07719... at 35.00%
19    15aa, >antibacterial07720... at 35.00%
20    12aa, >antibacterial08117... *
21    12aa, >antibacterial08321... at 41.67%
22    12aa, >antibacterial08334... at 41.67%
23    20aa, >antibacterial08581... at 35.00%
24    18aa, >antibacterial08614... at 50.00%
25    18aa, >antibacterial08615... at 44.44%
26    18aa, >antibacterial08617... at 50.00%
27    17aa, >antibacterial08671... at 41.18%
28    15aa, >antibacterial08696... at 35.00%
29    17aa, >antibacterial08710... at 41.18%
30    12aa, >antibacterial08985... at 41.67%
31    12aa, >antibacterial08988... at 41.67%
32    17aa, >antibacterial09173... at 41.18%
33    19aa, >antibacterial10391... at 42.11%
34    11aa, >antibacterial10578... at 54.55%
35    30aa, >antibacterial10618... at 43.33%
36    15aa, >antibacterial11323... at 46.67%
37    15aa, >antibacterial11894... at 35.00%
```

```
38    15aa, >antibacterial12033... at 35.00%
39    11aa, >antibacterial12847... at 54.55%
40    20aa, >antibacterial12898... at 35.00%
41    20aa, >antibacterial13025... at 35.00%
42    20aa, >antibacterial13071... at 35.00%
43    20aa, >antibacterial13304... at 35.00%
44    12aa, >antibacterial13366... at 41.67%
>Cluster 33
0     15aa, >antimicrobial... at 35.00%
1     21aa, >anti-Gram-;Antimicr... at 47.62%
2     13aa, >anti-Gram+;antibact... at 46.15%
3     255aa, >Antimicrobial... *
4     11aa, >Antimicrobial... at 45.45%
5     19aa, >Antimicrobial... at 42.11%
6     13aa, >antibacterial07827... at 46.15%
7     13aa, >antibacterial08152... at 46.15%
8     15aa, >antibacterial08651... at 35.00%
9     11aa, >antibacterial09643... at 45.45%
10    12aa, >antibacterial10068... at 50.00%
11    13aa, >antibacterial10119... at 46.15%
12    32aa, >antibacterial10605... at 40.62%
13    16aa, >antibacterial11541... at 43.75%
14    20aa, >antibacterial12127... at 35.00%
15    13aa, >antibacterial12315... *
16    13aa, >antibacterial12329... at 46.15%
17    13aa, >antibacterial12330... at 46.15%
18    15aa, >antibacterial12820... at 35.00%
19    13aa, >antibacterial12960... at 46.15%
20    21aa, >antibacterial13024... at 42.86%
21    11aa, >antibacterial13342... at 45.45%
>Cluster 34
0     28aa, >anti-Gram+;antibact... at 42.86%
1     13aa, >antimicrobial... at 46.15%
2     13aa, >antimicrobial... at 46.15%
3     17aa, >Antibacterial;antim... at 47.06%
4     16aa, >Antibacterial... at 43.75%
5     20aa, >Antibacterial;antim... at 50.00%
6     26aa, >anti-Gram+;antibact... at 46.15%
7     11aa, >antimicrobial... at 54.55%
8     13aa, >antibacterial;Antif... at 53.85%
9     13aa, >antibacterial;antim... *
10    18aa, >antibacterial;antif... at 50.00%
11    20aa, >antibacterial;antim... at 35.00%
12    30aa, >antibacterial;antim... at 35.00%
13    27aa, >antimicrobial... at 40.74%
14    25aa, >anti-Gram+;antibact... at 35.00%
15    17aa, >antibacterial;antim... at 41.18%
16    15aa, >Antibacterial... at 35.00%
17    255aa, >Antiviral... *
18    255aa, >Antiviral... at 76.86%
19    255aa, >Antiviral... at 75.69%
20    255aa, >Antiviral... at 77.25%
21    15aa, >Antimicrobial... at 46.67%
22    12aa, >antibacterial06666... at 50.00%
```

23   20aa, >L02A001911... at 35.00%
24   19aa, >antibacterial07881... at 42.11%
25   25aa, >antibacterial08006... at 35.00%
26   11aa, >antibacterial08284... at 45.45%
27   14aa, >antibacterial08397... at 42.86%
28   14aa, >antibacterial08398... at 42.86%
29   20aa, >antibacterial08482... at 35.00%
30   12aa, >antibacterial08507... at 41.67%
31   12aa, >antibacterial08566... at 41.67%
32   12aa, >antibacterial09040... at 41.67%
33   12aa, >antibacterial09049... at 41.67%
34   14aa, >antibacterial09166... at 42.86%
35   11aa, >antibacterial09319... at 45.45%
36   20aa, >antibacterial09377... at 35.00%
37   13aa, >antibacterial09540... at 53.85%
38   18aa, >antibacterial09664... at 44.44%
39   20aa, >antibacterial10086... *
40   21aa, >antibacterial10454... at 42.86%
41   11aa, >antibacterial10508... at 45.45%
42   13aa, >antibacterial11418... at 53.85%
43   16aa, >antibacterial11515... at 43.75%
44   13aa, >antibacterial11627... at 53.85%
45   22aa, >antibacterial11889... at 40.91%
46   19aa, >antibacterial12024... at 42.11%
47   13aa, >antibacterial12077... at 53.85%
48   15aa, >antibacterial12411... at 35.00%
49   12aa, >antibacterial12572... at 41.67%
50   12aa, >antibacterial13119... at 41.67%
51   12aa, >antibacterial13214... at 41.67%
52   15aa, >antibacterial13264... at 35.00%
53   15aa, >antibacterial13374... at 46.67%
>Cluster 35
0   20aa, >antimicrobial... at 35.00%
1   21aa, >antimicrobial... at 42.86%
2   21aa, >antimicrobial... at 42.86%
3   21aa, >anti-Gram+;antibact... *
4   21aa, >anti-Gram+;antibact... at 47.62%
5   38aa, >Antibacterial;Antif... at 42.11%
6   21aa, >antimicrobial... at 42.86%
7   21aa, >Antibacterial;Antif... at 47.62%
8   15aa, >antibacterial... at 35.00%
9   20aa, >antibacterial;antim... at 45.00%
10   11aa, >Antibacterial... at 45.45%
11   255aa, >Antimicrobial... *
12   12aa, >anti-Gram-;antibact... at 50.00%
13   22aa, >antimicrobial... at 40.91%
14   22aa, >antibacterial;antim... at 45.45%
15   32aa, >anti-Gram+;antifung... at 43.75%
16   13aa, >antibacterial06527... at 46.15%
17   17aa, >antibacterial06565... at 41.18%
18   11aa, >antibacterial06620... at 45.45%
19   24aa, >antibacterial06652... at 41.67%
20   12aa, >antibacterial06987... at 50.00%
21   14aa, >antibacterial06995... at 42.86%

```
22    14aa, >antibacterial06996... at 42.86%
23    13aa, >antibacterial07000... at 46.15%
24    12aa, >antibacterial07004... at 41.67%
25    12aa, >antibacterial07005... at 50.00%
26    12aa, >antibacterial07012... at 50.00%
27    13aa, >antibacterial07569... at 46.15%
28    12aa, >antibacterial07571... at 41.67%
29    16aa, >antibacterial07682... at 43.75%
30    11aa, >antibacterial07713... at 45.45%
31    15aa, >antibacterial08718... at 35.00%
32    24aa, >antibacterial08844... at 45.83%
33    27aa, >antibacterial08924... at 40.74%
34    27aa, >antibacterial08925... at 40.74%
35    13aa, >antibacterial09584... at 46.15%
36    13aa, >antibacterial09601... *
37    20aa, >antibacterial10951... at 35.00%
38    20aa, >antibacterial10953... at 35.00%
39    13aa, >antibacterial11202... at 46.15%
40    18aa, >antibacterial11617... at 44.44%
41    21aa, >antibacterial11795... at 42.86%
42    15aa, >antibacterial11892... at 35.00%
43    13aa, >antibacterial12048... at 46.15%
44    13aa, >antibacterial12055... at 46.15%
45    13aa, >antibacterial12067... at 53.85%
46    13aa, >antibacterial12101... at 46.15%
47    20aa, >antibacterial12124... at 45.00%
48    13aa, >antibacterial12425... at 46.15%
49    18aa, >antibacterial12607... at 44.44%
50    20aa, >antibacterial12713... at 35.00%
51    29aa, >antibacterial12760... at 41.38%
52    19aa, >antibacterial13131... at 42.11%
53    20aa, >antibacterial13181... at 35.00%
>Cluster 36
0     30aa, >Antimicrobial... at 43.33%
1     21aa, >Antimicrobial... *
2     21aa, >anti-HIV;antimicrob... at 52.38%
3     21aa, >anti-HIV;Antimicrob... at 52.38%
4     17aa, >Antibacterial;antim... at 52.94%
5     24aa, >antimicrobial... at 41.67%
6     24aa, >Antimicrobial... at 41.67%
7     15aa, >antibacterial;antim... at 35.00%
8     24aa, >antimicrobial... at 41.67%
9     24aa, >antimicrobial... at 41.67%
10    24aa, >antibacterial;Antif... at 41.67%
11    24aa, >antibacterial;Antif... at 41.67%
12    24aa, >antibacterial;Antif... at 50.00%
13    24aa, >anti-Gram+;Antibact... at 41.67%
14    24aa, >antimicrobial... at 41.67%
15    17aa, >Antimicrobial... *
16    24aa, >antibacterial;Antif... at 41.67%
17    24aa, >anti-Gram+;Antibact... at 45.83%
18    20aa, >antibacterial;Antif... at 45.00%
19    14aa, >Antimicrobial... at 42.86%
20    28aa, >anti-Gram-;Antibact... at 46.43%
```

```
21    31aa, >Gram-... at 41.94%
22    255aa, >Antimicrobial... *
23    255aa, >Antimicrobial... at 42.75%
24    255aa, >Antimicrobial... at 52.16%
25    255aa, >Antimicrobial... at 46.67%
26    211aa, >Antimicrobial... at 45.50%
27    255aa, >Antimicrobial... at 52.16%
28    14aa, >Antibacterial;Antim... at 42.86%
29    43aa, >anti-Gram+;antibact... at 44.19%
30    13aa, >antibacterial06478... at 46.15%
31    40aa, >antibacterial07326... at 35.00%
32    14aa, >antibacterial07463... at 42.86%
33    16aa, >antibacterial07585... at 43.75%
34    15aa, >antibacterial07764... at 35.00%
35    20aa, >antibacterial07994... at 35.00%
36    18aa, >antibacterial08592... at 44.44%
37    18aa, >antibacterial08613... at 44.44%
38    14aa, >antibacterial08995... at 42.86%
39    13aa, >antibacterial09072... at 46.15%
40    24aa, >antibacterial09508... at 41.67%
41    24aa, >antibacterial09724... at 41.67%
42    12aa, >antibacterial10066... at 41.67%
43    15aa, >antibacterial10069... at 35.00%
44    29aa, >antibacterial11385... at 41.38%
45    24aa, >antibacterial11707... at 45.83%
46    24aa, >antibacterial11708... at 41.67%
47    27aa, >antibacterial11875... at 40.74%
48    15aa, >antibacterial11958... at 35.00%
49    20aa, >antibacterial12009... at 45.00%
50    25aa, >antibacterial12625... at 44.00%
51    25aa, >antibacterial12626... at 35.00%
52    18aa, >antibacterial13172... at 44.44%
53    20aa, >antibacterial13192... at 45.00%
>Cluster 37
0     13aa, >antimicrobial... *
1     18aa, >antimicrobial... at 44.44%
2     19aa, >anti-Gram+;antibact... at 42.11%
3     13aa, >antibacterial;antim... at 46.15%
4     12aa, >anti-Gram-;antibact... at 41.67%
5     18aa, >anti-Gram-;antibact... at 44.44%
6     21aa, >antimicrobial... at 42.86%
7     255aa, >Antimicrobial... *
8     21aa, >antimicrobial... at 47.62%
9     24aa, >Antimicrobial... at 41.67%
10    20aa, >antimicrobial... at 35.00%
11    22aa, >antibacterial06543... at 40.91%
12    20aa, >antibacterial06651... at 35.00%
13    28aa, >antibacterial07071... at 50.00%
14    25aa, >antibacterial07271... at 44.00%
15    25aa, >antibacterial07272... at 48.00%
16    12aa, >antibacterial07382... at 41.67%
17    13aa, >antibacterial07849... at 46.15%
18    11aa, >antibacterial08111... at 45.45%
19    17aa, >antibacterial08277... at 41.18%
```

```
20    11aa, >antibacterial08302... at 45.45%
21    12aa, >antibacterial08311... at 41.67%
22    14aa, >antibacterial08315... at 42.86%
23    17aa, >antibacterial08331... at 41.18%
24    16aa, >antibacterial08355... at 43.75%
25    14aa, >antibacterial08372... at 42.86%
26    14aa, >antibacterial08373... at 42.86%
27    13aa, >antibacterial08382... at 53.85%
28    13aa, >antibacterial08383... at 53.85%
29    14aa, >antibacterial08399... *
30    11aa, >antibacterial08804... at 45.45%
31    12aa, >antibacterial08955... at 41.67%
32    19aa, >antibacterial09180... at 42.11%
33    12aa, >antibacterial09270... at 41.67%
34    11aa, >antibacterial09311... at 45.45%
35    11aa, >antibacterial09330... at 45.45%
36    16aa, >antibacterial09803... at 43.75%
37    17aa, >antibacterial09913... at 41.18%
38    12aa, >antibacterial10006... at 41.67%
39    18aa, >antibacterial10228... at 44.44%
40    21aa, >antibacterial10289... *
41    11aa, >antibacterial10595... at 42.86%
42    13aa, >antibacterial11311... at 46.15%
43    20aa, >antibacterial11491... at 35.00%
44    20aa, >antibacterial11578... at 35.00%
45    20aa, >antibacterial11752... at 35.00%
46    20aa, >antibacterial11816... at 35.00%
47    15aa, >antibacterial12555... at 35.00%
48    15aa, >antibacterial12635... at 35.00%
49    15aa, >antibacterial12651... at 35.00%
50    14aa, >antibacterial12656... at 42.86%
51    15aa, >antibacterial12679... at 35.00%
52    15aa, >antibacterial12862... at 46.67%
53    15aa, >antibacterial13179... at 35.00%
54    18aa, >antibacterial13219... at 44.44%
55    12aa, >antibacterial13323... at 50.00%
>Cluster 38
0     30aa, >anti-Gram+;Antibact... at 35.00%
1     24aa, >antimicrobial... at 50.00%
2     18aa, >anti-Gram+;Antibact... at 44.44%
3     13aa, >antibacterial;antim... *
4     29aa, >antimicrobial... at 41.38%
5     21aa, >anti-Gram+;Antibact... at 42.86%
6     29aa, >antimicrobial... at 41.38%
7     19aa, >Antimicrobial... at 47.37%
8     13aa, >Antibacterial;antim... at 53.85%
9     255aa, >Antimicrobial... *
10    35aa, >anti-Gram+;Antifung... at 35.00%
11    17aa, >antibacterial;antim... at 41.18%
12    18aa, >antibacterial07557... at 44.44%
13    13aa, >antibacterial07747... at 46.15%
14    29aa, >antibacterial07792... at 41.38%
15    15aa, >antibacterial08692... at 35.00%
16    13aa, >antibacterial08893... at 46.15%
```

17   19aa, >antibacterial09286... at 42.11%
18   20aa, >antibacterial09385... at 35.00%
19   20aa, >antibacterial09406... at 45.00%
20   20aa, >antibacterial09417... at 45.00%
21   11aa, >antibacterial09698... *
22   12aa, >antibacterial09790... at 41.67%
23   12aa, >antibacterial10128... at 41.67%
24   22aa, >antibacterial10445... at 40.91%
25   12aa, >antibacterial10667... at 41.67%
26   17aa, >antibacterial10874... at 41.18%
27   13aa, >antibacterial11201... at 46.15%
28   13aa, >antibacterial12158... at 46.15%
29   13aa, >antibacterial12230... at 46.15%
30   25aa, >antibacterial12637... at 35.00%
31   20aa, >antibacterial13088... at 50.00%
>Cluster 39
0   25aa, >antibacterial;Antif... at 35.00%
1   13aa, >antimicrobial... at 53.85%
2   32aa, >antimicrobial... at 40.62%
3   15aa, >antimicrobial... *
4   255aa, >Antimicrobial... *
5   255aa, >Antimicrobial... at 57.65%
6   255aa, >Antimicrobial... at 44.31%
7   255aa, >Antimicrobial... at 59.22%
8   255aa, >Antimicrobial... at 57.25%
9   17aa, >Antimicrobial... at 41.18%
10   17aa, >antimicrobial... at 41.18%
11   13aa, >antimicrobial... at 46.15%
12   14aa, >antibacterial06966... at 50.00%
13   11aa, >antibacterial07712... at 45.45%
14   15aa, >antibacterial08317... at 35.00%
15   15aa, >antibacterial08320... at 35.00%
16   12aa, >antibacterial08465... at 41.67%
17   12aa, >antibacterial09518... at 66.67%
18   12aa, >antibacterial09520... at 50.00%
19   12aa, >antibacterial09521... at 41.67%
20   13aa, >antibacterial09732... at 46.15%
21   23aa, >antibacterial10893... at 43.48%
22   14aa, >antibacterial11237... at 42.86%
23   15aa, >antibacterial11259... at 35.00%
24   19aa, >antibacterial11391... at 42.11%
25   18aa, >antibacterial11416... *
26   15aa, >antibacterial11443... at 35.00%
27   20aa, >antibacterial11474... at 45.00%
28   14aa, >antibacterial11621... at 42.86%
29   13aa, >antibacterial11924... at 53.85%
30   12aa, >antibacterial12912... at 41.67%
>Cluster 40
0   13aa, >antimicrobial... *
1   31aa, >anti-Gram+;antibact... at 41.94%
2   16aa, >antibacterial;antif... at 43.75%
3   14aa, >antimicrobial... at 42.86%
4   14aa, >anti-Gram-;anti-HIV... at 42.86%
5   24aa, >Antifungal... at 41.67%

6 13aa, >antimicrobial... at 46.15%
7 20aa, >antibacterial;antim... at 45.00%
8 20aa, >antibacterial;antim... at 35.00%
9 24aa, >Antibacterial;Antif... at 41.67%
10 13aa, >anti-Gram+;Antibact... at 46.15%
11 17aa, >antibacterial;antim... at 41.18%
12 19aa, >Antibacterial... at 47.37%
13 20aa, >antibacterial;antim... at 35.00%
14 20aa, >Gram-... at 35.00%
15 28aa, >antibacterial;antim... at 42.86%
16 16aa, >antimicrobial... at 50.00%
17 248aa, >Antimicrobial... *
18 248aa, >Antimicrobial... at 63.31%
19 247aa, >Antimicrobial... at 64.37%
20 249aa, >Antimicrobial... at 62.65%
21 248aa, >Antimicrobial... at 64.92%
22 254aa, >Antimicrobial... at 57.09%
23 254aa, >Antimicrobial... at 64.17%
24 249aa, >Antimicrobial... at 67.07%
25 255aa, >Antimicrobial... *
26 254aa, >Antimicrobial... at 70.87%
27 255aa, >Antimicrobial... at 46.27%
28 252aa, >Antimicrobial... at 51.19%
29 60aa, >Antimicrobial... at 35.00%
30 255aa, >Antimicrobial... at 43.53%
31 255aa, >Antimicrobial... at 40.78%
32 255aa, >Antimicrobial... at 41.18%
33 255aa, >Antimicrobial... at 47.06%
34 14aa, >Antimicrobial... at 42.86%
35 30aa, >Antimicrobial;Antim... at 35.00%
36 20aa, >Antimicrobial... at 45.00%
37 30aa, >Antibacterial... at 35.00%
38 13aa, >antimicrobial... at 46.15%
39 30aa, >antibacterial06489... at 43.33%
40 17aa, >antibacterial07612... at 41.18%
41 20aa, >antibacterial07954... at 35.00%
42 11aa, >antibacterial08101... at 54.55%
43 11aa, >antibacterial08301... at 45.45%
44 15aa, >antibacterial08458... at 35.00%
45 19aa, >antibacterial08534... at 42.11%
46 20aa, >antibacterial08713... at 35.00%
47 16aa, >antibacterial09013... at 43.75%
48 16aa, >antibacterial09015... at 43.75%
49 30aa, >antibacterial09233... at 35.00%
50 12aa, >antibacterial10142... *
51 13aa, >antibacterial11124... at 46.15%
52 13aa, >antibacterial11200... at 53.85%
53 13aa, >antibacterial11373... *
54 13aa, >antibacterial11376... at 31.54%
55 16aa, >antibacterial11429... at 43.75%
56 13aa, >antibacterial11758... at 46.15%
57 16aa, >antibacterial11899... at 43.75%
58 15aa, >antibacterial12001... at 35.00%
59 17aa, >antibacterial12121... at 41.18%

```
60    17aa, >antibacterial12129... at 41.18%
61    13aa, >antibacterial12225... at 46.15%
62    15aa, >antibacterial12591... at 35.00%
63    15aa, >antibacterial12996... at 35.00%
64    17aa, >antibacterial13043... at 47.06%
65    19aa, >antibacterial13233... at 42.11%
66    18aa, >antibacterial13359... at 50.00%
>Cluster 41
0     20aa, >Antimicrobial... at 35.00%
1     15aa, >antibacterial... at 35.00%
2     13aa, >antifungal;antimicr... *
3     28aa, >anti-Gram-;Antibact... at 42.86%
4     25aa, >Antifungal... at 35.00%
5     26aa, >antibacterial... at 42.31%
6     255aa, >Antimicrobial... *
7     40aa, >antimicrobial... at 35.00%
8     24aa, >antimicrobial... at 41.67%
9     27aa, >antibacterial08931... at 40.74%
10    20aa, >antibacterial09418... at 35.00%
11    14aa, >antibacterial10476... at 42.86%
12    26aa, >antibacterial10693... at 46.15%
13    17aa, >antibacterial11189... at 41.18%
14    13aa, >antibacterial11441... at 46.15%
15    14aa, >antibacterial11485... at 42.86%
16    17aa, >antibacterial11653... at 47.06%
17    13aa, >antibacterial12151... at 46.15%
18    12aa, >antibacterial12474... at 41.67%
>Cluster 42
0     13aa, >Antifungal;Antimicr... at 46.15%
1     11aa, >anti-Gram+;antivira... *
2     11aa, >Antibacterial;Antif... at 45.45%
3     15aa, >anti-Gram+;Antibact... at 35.00%
4     17aa, >antimicrobial... at 41.18%
5     17aa, >Antibacterial... at 41.18%
6     15aa, >Antibacterial... at 46.67%
7     13aa, >Antibacterial... at 46.15%
8     13aa, >antimicrobial... at 46.15%
9     25aa, >anti-Gram+;Antimicr... at 44.00%
10    19aa, >antibacterial;antif... at 42.11%
11    27aa, >antimicrobial... at 48.15%
12    24aa, >antimicrobial... at 41.67%
13    21aa, >antibacterial;Antif... at 42.86%
14    21aa, >antimicrobial... at 47.62%
15    21aa, >antimicrobial... at 42.86%
16    28aa, >antibacterial... at 42.86%
17    23aa, >Antimicrobial... at 47.83%
18    21aa, >anti-Gram+;anti-HIV... at 47.62%
19    30aa, >antimicrobial... at 46.67%
20    27aa, >Antibacterial;Antif... at 44.44%
21    21aa, >antimicrobial... at 42.86%
22    12aa, >Antifungal;Antimicr... at 50.00%
23    25aa, >antibacterial;antim... at 35.00%
24    30aa, >anti-Gram+;Antibact... at 35.00%
25    255aa, >Antimicrobial... *
```

26	11aa, >antibacterial;antim... at 54.55%
27	12aa, >antimicrobial... at 38.33%
28	14aa, >anti-Gram+;Antibact... at 42.86%
29	27aa, >Antibacterial... at 40.74%
30	18aa, >antibacterial06557... at 44.44%
31	13aa, >antibacterial06594... at 46.15%
32	29aa, >antibacterial06639... at 48.28%
33	28aa, >antibacterial06644... at 46.43%
34	26aa, >antibacterial06674... at 46.15%
35	20aa, >antibacterial07651... at 35.00%
36	13aa, >antibacterial08029... at 46.15%
37	16aa, >antibacterial08058... at 50.00%
38	25aa, >antibacterial08112... at 44.00%
39	20aa, >antibacterial08389... at 35.00%
40	17aa, >antibacterial08390... at 47.06%
41	23aa, >antibacterial08419... at 43.48%
42	17aa, >antibacterial08456... at 47.06%
43	20aa, >antibacterial08476... at 35.00%
44	23aa, >antibacterial08558... at 52.17%
45	12aa, >antibacterial08564... at 50.00%
46	19aa, >antibacterial08661... at 42.11%
47	21aa, >antibacterial09237... at 42.86%
48	23aa, >antibacterial09475... *
49	28aa, >antibacterial09478... at 42.86%
50	25aa, >antibacterial09535... at 44.00%
51	16aa, >antibacterial09545... at 50.00%
52	18aa, >antibacterial09722... at 50.00%
53	13aa, >antibacterial09740... at 53.85%
54	30aa, >antibacterial10043... at 35.00%
55	11aa, >antibacterial10271... *
56	17aa, >antibacterial10411... at 41.18%
57	21aa, >antibacterial10455... at 42.86%
58	16aa, >antibacterial10497... at 43.75%
59	19aa, >antibacterial10636... at 42.11%
60	13aa, >antibacterial10715... at 46.15%
61	23aa, >antibacterial11136... at 43.48%
62	14aa, >antibacterial11230... at 42.86%
63	13aa, >antibacterial11468... at 46.15%
64	18aa, >antibacterial11592... at 50.00%
65	18aa, >antibacterial11616... at 44.44%
66	13aa, >antibacterial11776... at 53.85%
67	18aa, >antibacterial11820... at 50.00%
68	27aa, >antibacterial11835... at 40.74%
69	15aa, >antibacterial11971... at 35.00%
70	13aa, >antibacterial12041... at 46.15%
71	20aa, >antibacterial12095... at 45.00%
72	17aa, >antibacterial12115... at 47.06%
73	13aa, >antibacterial12220... at 53.85%
74	13aa, >antibacterial12254... at 46.15%
75	13aa, >antibacterial12259... at 46.15%
76	13aa, >antibacterial12269... at 46.15%
77	13aa, >antibacterial12271... at 46.15%
78	13aa, >antibacterial12272... at 46.15%
79	13aa, >antibacterial12273... at 46.15%

```
80    13aa, >antibacterial12274... at 46.15%
81    14aa, >antibacterial12282... at 42.86%
82    13aa, >antibacterial12288... at 46.15%
83    13aa, >antibacterial12327... at 46.15%
84    18aa, >antibacterial12353... at 44.44%
85    20aa, >antibacterial12488... at 35.00%
86    14aa, >antibacterial12515... at 42.86%
87    20aa, >antibacterial12654... at 35.00%
88    12aa, >antibacterial13121... *
89    20aa, >antibacterial13156... at 35.00%
90    19aa, >antibacterial13224... at 42.11%
91    12aa, >antibacterial13407... at 41.67%
>Cluster 43
0     28aa, >Antifungal;Gram-... at 42.86%
1     21aa, >antimicrobial... at 42.86%
2     25aa, >anti-Gram+;antifung... at 35.00%
3     22aa, >antimicrobial... at 45.45%
4     24aa, >antibacterial... at 45.83%
5     23aa, >antimicrobial... at 43.48%
6     13aa, >antimicrobial;antiv... *
7     13aa, >antibacterial;antim... at 46.15%
8     19aa, >Antibacterial;antim... at 42.11%
9     11aa, >anti-Gram+;antibact... at 45.45%
10    255aa, >Antimicrobial... *
11    16aa, >Antifungal... at 43.75%
12    12aa, >antibacterial06994... at 41.67%
13    12aa, >antibacterial06999... at 41.67%
14    11aa, >antibacterial07711... at 45.45%
15    20aa, >antibacterial08477... at 35.00%
16    20aa, >antibacterial08543... at 45.00%
17    13aa, >antibacterial08905... at 46.15%
18    20aa, >antibacterial09932... at 35.00%
19    13aa, >antibacterial10116... at 46.15%
20    27aa, >antibacterial10251... at 40.74%
21    30aa, >antibacterial10607... at 43.33%
22    22aa, >antibacterial10895... *
23    18aa, >antibacterial11343... at 44.44%
24    30aa, >antibacterial11354... at 43.33%
25    36aa, >antibacterial11487... at 41.67%
26    14aa, >antibacterial12088... at 42.86%
27    20aa, >antibacterial12630... at 35.00%
28    12aa, >antibacterial12657... at 41.67%
29    15aa, >antibacterial12835... at 46.67%
30    20aa, >antibacterial13053... at 45.00%
31    18aa, >antibacterial13054... at 44.44%
32    20aa, >antibacterial13235... at 35.00%
>Cluster 44
0     26aa, >antimicrobial... at 42.31%
1     17aa, >antimicrobial... at 41.18%
2     17aa, >antibacterial... at 41.18%
3     13aa, >anti-Gram+;Antibact... at 46.15%
4     20aa, >antibacterial... at 35.00%
5     34aa, >Antibacterial... at 41.18%
6     24aa, >anti-Gram+;Antibact... at 41.67%
```

7 12aa, >Antifungal;Antimicr... at 41.67%
8 253aa, >Antimicrobial... at 47.83%
9 255aa, >Antimicrobial... *
10 255aa, >Antimicrobial... at 46.67%
11 255aa, >Antimicrobial... at 55.29%
12 255aa, >Antimicrobial... at 53.92%
13 256aa, >Antimicrobial... at 55.69%
14 256aa, >Antimicrobial... at 55.69%
15 256aa, >Antimicrobial... at 54.51%
16 257aa, >Antimicrobial... at 50.59%
17 257aa, >Antimicrobial... at 57.65%
18 258aa, >Antimicrobial... at 52.94%
19 258aa, >Antibacterial;Antim... at 53.33%
20 258aa, >Antimicrobial... at 52.55%
21 18aa, >Antimicrobial... at 50.00%
22 13aa, >antibacterial06512... at 46.15%
23 24aa, >antibacterial06521... at 41.67%
24 18aa, >antibacterial06659... *
25 12aa, >antibacterial07024... at 41.67%
26 20aa, >antibacterial07355... at 35.00%
27 14aa, >antibacterial07458... at 42.86%
28 17aa, >antibacterial07909... at 41.18%
29 25aa, >antibacterial08068... at 35.00%
30 11aa, >antibacterial08798... at 45.45%
31 12aa, >antibacterial09022... at 50.00%
32 13aa, >antibacterial09232... at 46.15%
33 13aa, >antibacterial09280... at 46.15%
34 13aa, >antibacterial09281... at 46.15%
35 20aa, >antibacterial09367... at 35.00%
36 20aa, >antibacterial09444... at 45.00%
37 20aa, >antibacterial09933... at 35.00%
38 15aa, >antibacterial10156... at 35.00%
39 22aa, >antibacterial10175... at 40.91%
40 12aa, >antibacterial10239... at 41.67%
41 30aa, >antibacterial10617... at 43.33%
42 13aa, >antibacterial10694... at 46.15%
43 14aa, >antibacterial10840... at 42.86%
44 16aa, >antibacterial10908... at 50.00%
45 23aa, >antibacterial11325... at 43.48%
46 15aa, >antibacterial11392... at 46.67%
47 17aa, >antibacterial11393... at 41.18%
48 17aa, >antibacterial11424... at 41.18%
49 15aa, >antibacterial11629... at 35.00%
50 13aa, >antibacterial11697... at 46.15%
51 20aa, >antibacterial11878... *
52 12aa, >antibacterial11925... at 41.67%
53 15aa, >antibacterial11930... at 35.00%
54 12aa, >antibacterial11984... at 50.00%
55 20aa, >antibacterial12032... at 35.00%
56 13aa, >antibacterial12047... at 46.15%
57 13aa, >antibacterial12061... at 46.15%
58 12aa, >antibacterial12463... at 41.67%
59 15aa, >antibacterial12633... *
60 15aa, >antibacterial12638... at 53.33%

61 13aa, >antibacterial12860... at 46.15%
62 32aa, >antibacterial13044... at 46.88%
63 30aa, >antibacterial13230... at 43.33%
>Cluster 45
0 36aa, >antibacterial;antim... at 41.67%
1 20aa, >Antifungal... at 35.00%
2 16aa, >antibacterial;antim... at 43.75%
3 18aa, >antibacterial;antif... at 44.44%
4 21aa, >antimicrobial... at 42.86%
5 27aa, >antibacterial... at 40.74%
6 37aa, >anti-Gram+;antibact... at 40.54%
7 25aa, >antibacterial;antim... at 44.00%
8 255aa, >Antimicrobial... *
9 255aa, >Antimicrobial... at 48.63%
10 18aa, >Antimicrobial... at 44.44%
11 16aa, >Antimicrobial... at 43.75%
12 21aa, >anti-Gram+;antibact... at 47.62%
13 15aa, >antimicrobial... at 35.00%
14 26aa, >antibacterial06550... at 46.15%
15 21aa, >antibacterial07578... at 42.86%
16 34aa, >antibacterial07791... at 41.18%
17 29aa, >antibacterial07845... at 48.28%
18 15aa, >antibacterial07975... at 46.67%
19 13aa, >antibacterial09866... at 53.85%
20 20aa, >antibacterial10923... at 35.00%
21 11aa, >antibacterial11210... at 45.45%
22 22aa, >antibacterial11407... at 40.91%
23 16aa, >antibacterial11408... *
24 19aa, >antibacterial11454... at 42.11%
25 18aa, >antibacterial11754... at 55.56%
26 20aa, >antibacterial11896... at 35.00%
27 20aa, >antibacterial12030... at 35.00%
28 12aa, >antibacterial12946... *
29 18aa, >antibacterial13302... at 44.44%
30 12aa, >antibacterial13332... at 41.67%
31 20aa, >antibacterial13364... at 35.00%
>Cluster 46
0 16aa, >antimicrobial... at 43.75%
1 22aa, >anti-Gram+;antibact... at 40.91%
2 16aa, >Antimicrobial... at 43.75%
3 21aa, >antibacterial;antim... at 42.86%
4 21aa, >antimicrobial... *
5 21aa, >antimicrobial... at 47.62%
6 13aa, >antimicrobial... at 36.15%
7 255aa, >Antibacterial... *
8 44aa, >Antimicrobial... at 40.91%
9 17aa, >antibacterial06568... *
10 19aa, >antibacterial07350... at 42.11%
11 18aa, >antibacterial07396... at 50.00%
12 12aa, >antibacterial07917... at 50.00%
13 11aa, >antibacterial08229... at 45.45%
14 18aa, >antibacterial08442... at 44.44%
15 12aa, >antibacterial08964... at 41.67%
16 20aa, >antibacterial09436... at 35.00%

```
17    15aa, >antibacterial09837... at 46.67%
18    12aa, >antibacterial10534... at 41.67%
19    15aa, >antibacterial11570... at 35.00%
20    15aa, >antibacterial11644... at 35.00%
21    11aa, >antibacterial11687... at 45.45%
22    21aa, >antibacterial11739... at 42.86%
23    12aa, >antibacterial11927... at 41.67%
24    14aa, >antibacterial12035... at 42.86%
25    14aa, >antibacterial12090... at 42.86%
26    14aa, >antibacterial12091... at 42.86%
27    12aa, >antibacterial12978... *
28    21aa, >antibacterial13186... at 42.86%
29    15aa, >antibacterial13296... at 35.00%
30    12aa, >antibacterial13360... at 41.67%
>Cluster 47
0     21aa, >Antimicrobial... at 42.86%
1     13aa, >antimicrobial... at 46.15%
2     255aa, >Antimicrobial... *
3     14aa, >antimicrobial... at 42.86%
4     12aa, >anti-Gram+;antibact... at 41.67%
5     11aa, >antibacterial07855... at 45.45%
6     11aa, >antibacterial07862... at 45.45%
7     11aa, >antibacterial07863... at 45.45%
8     13aa, >antibacterial08186... at 46.15%
9     17aa, >antibacterial08675... at 41.18%
10    12aa, >antibacterial08934... *
11    12aa, >antibacterial09792... at 41.67%
12    17aa, >antibacterial11761... at 41.18%
13    15aa, >antibacterial11839... at 35.00%
14    29aa, >antibacterial11886... at 41.38%
15    11aa, >antibacterial12855... at 45.45%
16    12aa, >antibacterial13068... at 50.00%
>Cluster 48
0     25aa, >antimicrobial... at 44.00%
1     21aa, >anti-Gram+;antibact... *
2     23aa, >antibacterial;antim... at 43.48%
3     13aa, >anti-Gram+;Antibact... at 46.15%
4     23aa, >antimicrobial... at 43.48%
5     13aa, >antibacterial;antim... at 53.85%
6     24aa, >antimicrobial... at 45.83%
7     19aa, >Antibacterial;Antif... at 47.37%
8     254aa, >Antibacterial;Antiv... *
9     21aa, >antimicrobial... at 42.86%
10    34aa, >anti-Gram+;antibact... at 41.18%
11    33aa, >antimicrobial... at 42.42%
12    33aa, >Antimicrobial... at 48.48%
13    22aa, >Antimicrobial... at 45.45%
14    29aa, >antimicrobial... at 41.38%
15    29aa, >antimicrobial... at 41.38%
16    25aa, >anti-Gram+;antibact... at 35.00%
17    25aa, >Anti-Gram-;antibact... at 35.00%
18    25aa, >antimicrobial... at 44.00%
19    21aa, >Antibacterial... at 47.62%
20    14aa, >Antifungal;Gram-... at 57.14%
```

```
21    26aa, >antibacterial... at 46.15%
22    255aa, >Antibacterial... *
23    15aa, >antibacterial07526... at 35.00%
24    14aa, >antibacterial07579... at 50.00%
25    11aa, >antibacterial07869... at 45.45%
26    11aa, >antibacterial07870... at 45.45%
27    25aa, >antibacterial07884... at 44.00%
28    12aa, >antibacterial07902... at 41.67%
29    13aa, >antibacterial08187... at 53.85%
30    11aa, >antibacterial08362... at 45.45%
31    32aa, >antibacterial08570... at 40.62%
32    24aa, >antibacterial08782... *
33    17aa, >antibacterial08838... at 47.06%
34    17aa, >antibacterial09549... at 41.18%
35    15aa, >antibacterial09835... at 46.67%
36    15aa, >antibacterial09838... at 35.00%
37    11aa, >antibacterial09891... *
38    11aa, >antibacterial10593... at 54.55%
39    16aa, >antibacterial10856... at 43.75%
40    12aa, >antibacterial11720... at 41.67%
41    20aa, >antibacterial12069... at 35.00%
42    15aa, >antibacterial12174... at 35.00%
43    18aa, >antibacterial12628... at 50.00%
44    18aa, >antibacterial12692... at 50.00%
45    20aa, >antibacterial13194... at 35.00%
46    20aa, >antibacterial13260... at 35.00%
>Cluster 49
0     37aa, >Antimicrobial... *
1     45aa, >anti-Gram+;Antimicr... at 64.44%
2     71aa, >Antimicrobial... at 46.48%
3     44aa, >Antimicrobial... at 59.09%
4     22aa, >anti-Gram+;Antimicr... at 77.27%
5     27aa, >Antimicrobial... *
6     25aa, >Antifungal... at 44.00%
7     56aa, >Antimicrobial... at 41.07%
8     53aa, >Antimicrobial... at 54.72%
9     70aa, >Antimicrobial... at 35.00%
10    68aa, >Antimicrobial... at 44.12%
11    70aa, >Antimicrobial... at 51.43%
12    80aa, >Antimicrobial... at 50.00%
13    71aa, >Antimicrobial... at 42.25%
14    62aa, >Antimicrobial... at 51.61%
15    72aa, >Antimicrobial... at 50.00%
16    76aa, >Antimicrobial... at 46.05%
17    72aa, >Antimicrobial... at 58.33%
18    71aa, >Antimicrobial... at 49.30%
19    71aa, >Antimicrobial... at 49.30%
20    71aa, >Antimicrobial... at 42.25%
21    71aa, >Antimicrobial... at 45.07%
22    70aa, >Antimicrobial... at 50.00%
23    84aa, >Antimicrobial... at 40.48%
24    84aa, >Antimicrobial... at 40.48%
25    72aa, >Antimicrobial... at 48.61%
26    70aa, >Antimicrobial... at 42.86%
```

27 80aa, >Antimicrobial... at 50.00%
28 72aa, >Antimicrobial... at 40.28%
29 78aa, >Antimicrobial... at 44.87%
30 64aa, >Antimicrobial... at 48.44%
31 75aa, >Antimicrobial... at 48.00%
32 71aa, >Antimicrobial... at 56.34%
33 67aa, >Antimicrobial... at 47.76%
34 66aa, >Antimicrobial... at 48.48%
35 68aa, >Antimicrobial... at 44.12%
36 72aa, >Antibacterial;Antif... at 56.94%
37 70aa, >Antimicrobial... *
38 67aa, >Antimicrobial... at 49.25%
39 65aa, >Antimicrobial... at 55.38%
40 62aa, >Antimicrobial... at 53.23%
41 62aa, >Antimicrobial... at 54.84%
42 62aa, >Antimicrobial... at 58.06%
43 64aa, >Antimicrobial... at 56.25%
44 63aa, >Antimicrobial... at 53.97%
45 67aa, >Antimicrobial... at 49.25%
46 255aa, >Antimicrobial... *
47 63aa, >Antimicrobial... at 50.79%
48 79aa, >Antimicrobial... at 48.10%
49 64aa, >Antimicrobial... at 56.25%
50 64aa, >Antimicrobial... at 53.12%
51 255aa, >Antimicrobial... at 85.49%
52 86aa, >Antimicrobial... at 45.35%
53 70aa, >Antimicrobial... at 52.86%
54 62aa, >Antimicrobial... at 53.23%
55 59aa, >Antimicrobial... at 57.63%
56 145aa, >Antimicrobial... at 67.59%
57 189aa, >Antimicrobial... at 70.37%
58 233aa, >Antimicrobial... at 72.53%
59 255aa, >Antimicrobial... at 76.47%
60 199aa, >Antimicrobial... *
61 76aa, >Antimicrobial... at 51.32%
62 71aa, >Antimicrobial... at 61.97%
63 62aa, >Antimicrobial... at 56.45%
64 62aa, >Antimicrobial... at 61.29%
65 71aa, >Antimicrobial... at 50.70%
66 80aa, >Antimicrobial... at 56.25%
67 68aa, >Antimicrobial... at 55.88%
68 70aa, >Antimicrobial... at 52.86%
69 66aa, >Antimicrobial... at 53.03%
70 76aa, >Antimicrobial... at 50.00%
71 85aa, >Antimicrobial... at 35.00%
72 70aa, >Antibacterial... at 47.14%
73 70aa, >Antimicrobial... at 48.57%
74 68aa, >Antimicrobial... at 47.06%
75 70aa, >Antimicrobial... at 45.71%
76 73aa, >Antimicrobial... at 52.05%
77 78aa, >Antimicrobial... at 46.15%
78 75aa, >Antimicrobial... at 35.00%
79 72aa, >Antimicrobial... at 45.83%
80 70aa, >Antimicrobial... at 51.43%

81 67aa, >Antimicrobial... at 55.22%
82 71aa, >Antibacterial... at 42.25%
83 66aa, >Antimicrobial... at 51.52%
84 70aa, >Antimicrobial... at 41.43%
85 76aa, >Antimicrobial... at 44.74%
86 70aa, >Antimicrobial... at 42.86%
87 70aa, >Antimicrobial... at 45.71%
88 72aa, >Antimicrobial... at 48.61%
89 65aa, >Antimicrobial... at 44.62%
90 61aa, >Antimicrobial... at 75.41%
91 66aa, >Antimicrobial... at 50.00%
92 72aa, >Antimicrobial... at 52.78%
93 72aa, >Antimicrobial... at 50.00%
94 72aa, >Antimicrobial... at 40.28%
95 64aa, >Antimicrobial... at 51.56%
96 199aa, >Antimicrobial... *
97 76aa, >Antimicrobial... at 47.37%
98 76aa, >Antimicrobial... at 48.68%
99 72aa, >Antimicrobial... at 50.00%
100 66aa, >Antimicrobial... at 59.09%
101 80aa, >Antimicrobial... at 46.25%
102 78aa, >Antimicrobial... at 44.87%
103 67aa, >Antimicrobial... at 53.73%
104 71aa, >Antimicrobial... at 47.89%
105 70aa, >Antimicrobial... at 52.86%
106 70aa, >Antimicrobial... at 35.00%
107 70aa, >Antimicrobial... at 55.71%
108 72aa, >Antimicrobial... at 40.28%
109 191aa, >Antimicrobial... at 69.63%
110 209aa, >Antimicrobial... at 51.67%
111 72aa, >Antibacterial... at 41.67%
112 67aa, >Antimicrobial... at 41.79%
113 27aa, >Antimicrobial... *
114 42aa, >Antimicrobial... at 59.52%
115 40aa, >Antimicrobial... at 72.50%
116 25aa, >Antimicrobial... at 46.00%
117 40aa, >Antimicrobial... at 70.00%
118 14aa, >anti-Gram+;antibact... at 42.86%
119 28aa, >Antimicrobial... at 60.71%
120 41aa, >Antimicrobial... at 73.17%
121 21aa, >Antimicrobial... at 85.71%
122 40aa, >Antimicrobial... at 77.50%
123 36aa, >Antimicrobial... *
124 36aa, >Antimicrobial... at 45.00%
125 33aa, >Antimicrobial... at 75.76%
126 21aa, >Antimicrobial... *
127 36aa, >Antimicrobial... at 63.89%
128 44aa, >Antimicrobial... at 63.64%
129 44aa, >Antimicrobial... at 63.64%
130 20aa, >Anti-Gram-;Antibact... at 35.00%
131 15aa, >Antiviral... at 46.67%
132 20aa, >antibacterial06587... at 35.00%
133 61aa, >antibacterial06786... at 52.46%
134 66aa, >antibacterial06787... at 53.03%

```
135   74aa, >antibacterial06790... at 43.24%
136   74aa, >antibacterial07103... at 43.24%
137   74aa, >antibacterial07104... at 41.89%
138   20aa, >antibacterial07676... at 35.00%
139   13aa, >antibacterial07817... at 46.15%
140   13aa, >antibacterial07818... at 53.85%
141   13aa, >antibacterial07819... at 46.15%
142   24aa, >antibacterial08144... at 41.67%
143   15aa, >antibacterial08437... at 46.67%
144   15aa, >antibacterial08438... at 35.00%
145   11aa, >antibacterial08517... at 45.45%
146   11aa, >antibacterial08518... at 54.55%
147   19aa, >antibacterial08867... at 42.11%
148   19aa, >antibacterial08871... at 42.11%
149   16aa, >antibacterial08947... at 43.75%
150   16aa, >antibacterial08948... at 43.75%
151   16aa, >antibacterial08949... at 43.75%
152   15aa, >antibacterial09333... at 35.00%
153   13aa, >antibacterial09603... at 46.15%
154   12aa, >antibacterial10165... at 41.67%
155   14aa, >antibacterial10400... at 42.86%
156   13aa, >antibacterial10449... at 46.15%
157   62aa, >antibacterial10810... at 45.16%
158   65aa, >antibacterial10987... at 47.69%
159   62aa, >antibacterial10988... at 58.06%
160   61aa, >antibacterial10989... *
161   65aa, >antibacterial10990... at 50.77%
162   61aa, >antibacterial10991... at 52.46%
163   66aa, >antibacterial10992... at 48.48%
164   69aa, >antibacterial10993... at 47.83%
165   71aa, >antibacterial10994... at 47.89%
166   72aa, >antibacterial10995... at 50.00%
167   67aa, >antibacterial10997... at 53.73%
168   12aa, >antibacterial11165... at 41.67%
169   25aa, >antibacterial11272... at 76.00%
170   14aa, >antibacterial11510... at 64.29%
171   13aa, >antibacterial12196... at 46.15%
172   13aa, >antibacterial12197... at 46.15%
173   13aa, >antibacterial12198... at 46.15%
174   13aa, >antibacterial12211... at 46.15%
175   13aa, >antibacterial12212... at 46.15%
176   13aa, >antibacterial12213... at 46.15%
177   13aa, >antibacterial12215... at 53.85%
178   13aa, >antibacterial12217... at 46.15%
179   13aa, >antibacterial12228... at 46.15%
180   13aa, >antibacterial12229... at 46.15%
181   13aa, >antibacterial12231... at 46.15%
182   13aa, >antibacterial12250... at 46.15%
183   13aa, >antibacterial12251... at 46.15%
184   13aa, >antibacterial12253... at 61.54%
185   12aa, >antibacterial12335... at 41.67%
186   16aa, >antibacterial12890... at 43.75%
187   19aa, >antibacterial12907... at 42.11%
188   11aa, >antibacterial12937... *
```

```
189   11aa, >antibacterial12945... at 54.55%
190   12aa, >antibacterial13328... at 41.67%
>Cluster 50
0     21aa, >antibacterial... at 42.86%
1     255aa, >Antimicrobial... *
2     87aa, >Antimicrobial... at 44.83%
3     14aa, >antibacterial08738... *
4     20aa, >antibacterial09157... at 55.00%
5     11aa, >antibacterial09850... at 45.45%
6     19aa, >antibacterial11458... at 42.11%
7     14aa, >antibacterial11622... at 42.86%
8     14aa, >antibacterial12296... at 50.00%
9     13aa, >antibacterial12316... *
10    13aa, >antibacterial12317... at 46.15%
11    13aa, >antibacterial12318... at 46.15%
12    13aa, >antibacterial12321... at 46.15%
13    13aa, >antibacterial12323... at 46.15%
14    13aa, >antibacterial12328... at 46.15%
15    13aa, >antibacterial12333... at 46.15%
16    15aa, >antibacterial13267... at 46.67%
>Cluster 51
0     18aa, >anti-Gram+;antibact... at 44.44%
1     22aa, >Antifungal;antimicr... *
2     11aa, >antimicrobial... at 45.45%
3     15aa, >antimicrobial... at 35.00%
4     13aa, >antimicrobial... at 46.15%
5     40aa, >antimicrobial... at 42.50%
6     31aa, >antimicrobial... at 45.16%
7     14aa, >antimicrobial... at 42.86%
8     18aa, >antimicrobial... at 44.44%
9     13aa, >Antimicrobial... at 46.15%
10    27aa, >antimicrobial;antiv... at 40.74%
11    255aa, >Antimicrobial... *
12    21aa, >Antimicrobial... at 42.86%
13    20aa, >anti-Gram+;antibact... at 45.00%
14    20aa, >antibacterial... at 45.00%
15    23aa, >Antimicrobial... at 47.83%
16    15aa, >Antifungal;Antimicr... at 35.00%
17    32aa, >anti-Gram+;antibact... at 40.62%
18    28aa, >antibacterial... at 42.86%
19    13aa, >antibacterial06963... at 46.15%
20    11aa, >antibacterial07384... at 45.45%
21    18aa, >antibacterial07402... at 50.00%
22    18aa, >antibacterial07412... at 44.44%
23    30aa, >antibacterial07498... at 35.00%
24    17aa, >antibacterial07553... at 31.18%
25    24aa, >antibacterial07653... at 41.67%
26    13aa, >antibacterial07668... at 46.15%
27    13aa, >antibacterial07669... at 46.15%
28    13aa, >antibacterial07670... at 46.15%
29    13aa, >antibacterial07672... at 46.15%
30    20aa, >antibacterial07753... at 45.00%
31    14aa, >antibacterial07769... at 42.86%
32    14aa, >antibacterial07770... at 42.86%
```

33 14aa, >antibacterial07840... at 42.86%
34 14aa, >antibacterial07841... at 42.86%
35 15aa, >antibacterial07914... at 46.67%
36 12aa, >antibacterial07965... at 41.67%
37 17aa, >antibacterial08022... at 41.18%
38 11aa, >antibacterial08109... at 45.45%
39 17aa, >antibacterial08235... *
40 17aa, >antibacterial08270... at 41.18%
41 16aa, >antibacterial08307... at 43.75%
42 13aa, >antibacterial08380... at 53.85%
43 13aa, >antibacterial08384... at 46.15%
44 11aa, >antibacterial08522... at 45.45%
45 11aa, >antibacterial08631... at 45.45%
46 12aa, >antibacterial08837... at 41.67%
47 15aa, >antibacterial08859... at 35.00%
48 13aa, >antibacterial08897... at 46.15%
49 12aa, >antibacterial08945... at 41.67%
50 26aa, >antibacterial09206... at 46.15%
51 23aa, >antibacterial09225... at 43.48%
52 15aa, >antibacterial09269... at 46.67%
53 20aa, >antibacterial09419... at 45.00%
54 20aa, >antibacterial09431... at 35.00%
55 14aa, >antibacterial09457... at 42.86%
56 20aa, >antibacterial09458... *
57 13aa, >antibacterial09586... at 46.15%
58 15aa, >antibacterial09678... at 35.00%
59 18aa, >antibacterial09706... at 44.44%
60 21aa, >antibacterial09749... at 47.62%
61 12aa, >antibacterial09958... at 41.67%
62 20aa, >antibacterial10038... at 45.00%
63 13aa, >antibacterial10117... *
64 14aa, >antibacterial10206... at 50.00%
65 14aa, >antibacterial10332... at 42.86%
66 14aa, >antibacterial10334... at 50.00%
67 15aa, >antibacterial10357... *
68 15aa, >antibacterial10358... at 53.33%
69 24aa, >antibacterial10377... at 41.67%
70 23aa, >antibacterial10483... at 43.48%
71 16aa, >antibacterial10493... at 50.00%
72 13aa, >antibacterial10630... at 46.15%
73 18aa, >antibacterial10710... at 44.44%
74 20aa, >antibacterial10721... at 35.00%
75 14aa, >antibacterial10767... at 42.86%
76 14aa, >antibacterial10776... at 42.86%
77 20aa, >antibacterial11736... at 45.00%
78 13aa, >antibacterial11747... at 46.15%
79 12aa, >antibacterial12112... at 41.67%
80 13aa, >antibacterial12310... at 53.85%
81 17aa, >antibacterial12382... at 41.18%
82 12aa, >antibacterial12467... *
83 12aa, >antibacterial12468... at 41.67%
84 13aa, >antibacterial12469... at 41.67%
85 12aa, >antibacterial12470... at 41.67%
86 13aa, >antibacterial12471... at 41.67%

```
87    20aa, >antibacterial12536... at 45.00%
88    12aa, >antibacterial12588... at 41.67%
89    22aa, >antibacterial12710... at 40.91%
90    15aa, >antibacterial12950... at 46.67%
91    18aa, >antibacterial13092... at 44.44%
92    12aa, >antibacterial13168... at 50.00%
93    15aa, >antibacterial13266... at 35.00%
94    14aa, >antibacterial13319... at 42.86%
>Cluster 52
0     37aa, >antimicrobial... at 40.54%
1     27aa, >Antimicrobial... at 44.44%
2     19aa, >anti-Gram+;Antibact... at 42.11%
3     25aa, >antibacterial;Antif... at 44.00%
4     20aa, >Antibacterial... at 35.00%
5     22aa, >Antibacterial;antim... *
6     17aa, >anti-Gram+;Antimicr... at 41.18%
7     13aa, >antimicrobial... at 46.15%
8     13aa, >antimicrobial... *
9     24aa, >antibacterial;antim... at 41.67%
10    255aa, >Antimicrobial... *
11    16aa, >Antifungal;Antimicr... at 43.75%
12    15aa, >Antifungal;Antimicr... at 53.33%
13    15aa, >Antifungal;Antimicr... *
14    15aa, >Antifungal;Antimicr... at 53.33%
15    14aa, >Antibacterial... at 50.00%
16    11aa, >Antibacterial... at 45.45%
17    11aa, >antibacterial08212... at 45.45%
18    11aa, >antibacterial09369... at 54.55%
19    11aa, >antibacterial09371... at 45.45%
20    20aa, >antibacterial09410... at 45.00%
21    16aa, >antibacterial09499... at 50.00%
22    34aa, >antibacterial09868... at 41.18%
23    20aa, >antibacterial10037... at 35.00%
24    20aa, >antibacterial10040... at 35.00%
25    12aa, >antibacterial10655... at 41.67%
26    26aa, >antibacterial11155... at 42.31%
27    15aa, >antibacterial11294... at 35.00%
28    16aa, >antibacterial12739... at 50.00%
29    20aa, >antibacterial13303... at 45.00%
30    17aa, >antibacterial13392... at 41.18%
>Cluster 53
0     20aa, >Antibacterial;antim... at 35.00%
1     22aa, >anti-Gram+;antibact... at 45.45%
2     18aa, >Antibacterial;Antif... at 44.44%
3     24aa, >antimicrobial... *
4     22aa, >antibacterial;Antif... at 40.91%
5     23aa, >anti-Gram+;antibact... at 43.48%
6     25aa, >anti-Gram-;antibact... at 35.00%
7     26aa, >antimicrobial... at 42.31%
8     19aa, >antimicrobial... at 42.11%
9     26aa, >Antimicrobial... at 42.31%
10    26aa, >Antibacterial... at 42.31%
11    255aa, >Antibacterial... *
12    255aa, >Antibacterial... at 51.37%
```

```
13    255aa, >Antibacterial... at 46.67%
14    22aa, >Antimicrobial... at 45.45%
15    22aa, >Antimicrobial... at 40.91%
16    255aa, >Antibacterial... at 52.94%
17    18aa, >antibacterial07349... at 44.44%
18    18aa, >antibacterial07407... at 44.44%
19    15aa, >antibacterial08319... at 35.00%
20    16aa, >antibacterial08327... at 43.75%
21    17aa, >antibacterial08335... at 41.18%
22    22aa, >antibacterial08918... at 45.45%
23    21aa, >antibacterial09071... at 42.86%
24    20aa, >antibacterial09378... at 35.00%
25    14aa, >antibacterial09633... at 50.00%
26    13aa, >antibacterial09841... at 46.15%
27    20aa, >antibacterial10187... at 35.00%
28    21aa, >antibacterial10291... at 42.86%
29    19aa, >antibacterial10390... at 42.11%
30    13aa, >antibacterial10394... at 46.15%
31    13aa, >antibacterial10629... *
32    19aa, >antibacterial11587... at 42.11%
33    18aa, >antibacterial11649... *
34    27aa, >antibacterial11799... at 40.74%
35    24aa, >antibacterial12132... at 41.67%
36    13aa, >antibacterial12150... at 46.15%
37    16aa, >antibacterial12505... at 50.00%
38    20aa, >antibacterial12593... at 45.00%
39    15aa, >antibacterial12917... *
40    17aa, >antibacterial13069... at 41.18%
41    14aa, >antibacterial13244... at 42.86%
>Cluster 54
0     21aa, >anti-Gram+;antibact... at 42.86%
1     18aa, >antifungal;antimicr... at 44.44%
2     255aa, >Antibacterial... *
3     12aa, >antibacterial07017... at 41.67%
4     12aa, >antibacterial07023... at 41.67%
5     20aa, >antibacterial07950... at 45.00%
6     14aa, >antibacterial08549... at 42.86%
7     19aa, >antibacterial08608... at 42.11%
8     32aa, >antibacterial08878... at 40.62%
9     12aa, >antibacterial08954... at 41.67%
10    19aa, >antibacterial09750... *
11    15aa, >antibacterial11363... at 46.67%
12    21aa, >antibacterial11630... at 42.86%
13    22aa, >antibacterial11922... at 40.91%
14    19aa, >antibacterial13129... at 42.11%
>Cluster 55
0     255aa, >Antimicrobial... *
1     30aa, >antibacterial;antim... at 35.00%
2     12aa, >antibacterial06445... *
3     20aa, >antibacterial07919... at 35.00%
4     18aa, >antibacterial07969... at 50.00%
5     24aa, >antibacterial08282... at 41.67%
6     14aa, >antibacterial10335... at 50.00%
7     18aa, >antibacterial10703... at 50.00%
```

8   18aa, >antibacterial11161... at 44.44%
9   18aa, >antibacterial11941... *
10   15aa, >antibacterial12903... at 35.00%
>Cluster 56
0   21aa, >antimicrobial... at 42.86%
1   11aa, >antibacterial;antim... *
2   22aa, >antimicrobial... at 40.91%
3   22aa, >Antimicrobial... at 40.91%
4   17aa, >anti-Gram+;antibact... at 41.18%
5   255aa, >Antimicrobial... *
6   19aa, >antimicrobial... at 42.11%
7   21aa, >antimicrobial... at 47.62%
8   26aa, >Antibacterial... at 42.31%
9   22aa, >antibacterial;antim... at 45.45%
10   17aa, >antimicrobial... at 41.18%
11   17aa, >antimicrobial... at 41.18%
12   17aa, >antimicrobial... *
13   19aa, >antimicrobial... at 52.63%
14   24aa, >Antimicrobial... at 41.67%
15   11aa, >antibacterial06608... at 35.45%
16   11aa, >antibacterial07003... at 35.45%
17   18aa, >antibacterial07722... at 44.44%
18   13aa, >antibacterial08149... at 53.85%
19   25aa, >antibacterial09102... at 44.00%
20   25aa, >antibacterial09686... at 35.00%
21   17aa, >antibacterial10176... at 41.18%
22   17aa, >antibacterial10472... at 41.18%
23   11aa, >antibacterial10501... at 54.55%
24   15aa, >antibacterial11560... at 35.00%
25   24aa, >antibacterial11691... at 41.67%
26   20aa, >antibacterial11897... at 35.00%
27   13aa, >antibacterial12043... at 46.15%
28   17aa, >antibacterial12188... at 41.18%
29   17aa, >antibacterial12347... at 47.06%
30   11aa, >antibacterial12401... *
31   12aa, >antibacterial12476... at 50.00%
32   16aa, >antibacterial12500... *
33   18aa, >antibacterial12578... at 44.44%
34   30aa, >antibacterial12619... at 35.00%
35   20aa, >antibacterial12674... at 35.00%
36   14aa, >antibacterial12823... at 42.86%
37   14aa, >antibacterial12923... at 42.86%
38   19aa, >antibacterial13126... at 47.37%
>Cluster 57
0   14aa, >anti-Gram+;Gram-... at 42.86%
1   25aa, >Antibacterial... at 35.00%
2   13aa, >Antimicrobial... at 46.15%
3   21aa, >anti-Gram+;antibact... at 47.62%
4   11aa, >antimicrobial... *
5   13aa, >anti-Gram+;antibact... at 46.15%
6   13aa, >anti-Gram+;antibact... at 46.15%
7   30aa, >anti-Gram+;antibact... at 35.00%
8   255aa, >Antimicrobial... *
9   33aa, >antibacterial07548... at 45.45%

10 14aa, >antibacterial07679... at 42.86%
11 20aa, >antibacterial07943... at 35.00%
12 15aa, >antibacterial08199... at 53.33%
13 16aa, >antibacterial08325... *
14 18aa, >antibacterial08342... at 44.44%
15 16aa, >antibacterial08349... at 43.75%
16 16aa, >antibacterial08357... at 43.75%
17 17aa, >antibacterial08358... at 41.18%
18 16aa, >antibacterial08361... at 43.75%
19 12aa, >antibacterial08991... at 41.67%
20 11aa, >antibacterial09175... at 45.45%
21 11aa, >antibacterial09176... at 45.45%
22 11aa, >antibacterial09177... at 45.45%
23 19aa, >antibacterial09179... at 42.11%
24 12aa, >antibacterial09304... *
25 12aa, >antibacterial09305... at 50.00%
26 32aa, >antibacterial09500... at 43.75%
27 18aa, >antibacterial09812... at 44.44%
28 14aa, >antibacterial10201... at 42.86%
29 18aa, >antibacterial10701... at 44.44%
30 15aa, >antibacterial11664... at 35.00%
31 19aa, >antibacterial11780... at 42.11%
32 13aa, >antibacterial12309... at 46.15%
33 12aa, >antibacterial12475... at 41.67%
34 15aa, >antibacterial12581... at 35.00%
35 18aa, >antibacterial13094... at 44.44%
>Cluster 58
0 14aa, >anti-Gram+;antibact... at 42.86%
1 13aa, >antimicrobial... at 46.15%
2 19aa, >antimicrobial... at 42.11%
3 22aa, >anti-Gram+;antibact... at 40.91%
4 17aa, >Antimicrobial... at 41.18%
5 27aa, >anti-Gram+;antibact... at 40.74%
6 27aa, >antibacterial;antim... at 44.44%
7 20aa, >antibacterial;Antif... at 35.00%
8 37aa, >Antimicrobial;Gram-... at 40.54%
9 255aa, >Antimicrobial... *
10 255aa, >Antimicrobial... at 42.35%
11 255aa, >Antimicrobial... at 56.08%
12 255aa, >Antimicrobial... at 43.53%
13 32aa, >anti-Gram+;antibact... at 40.62%
14 12aa, >antibacterial06537... at 41.67%
15 15aa, >antibacterial07009... *
16 15aa, >antibacterial07520... at 46.67%
17 13aa, >antibacterial07565... at 46.15%
18 18aa, >antibacterial08425... at 44.44%
19 15aa, >antibacterial08435... at 35.00%
20 15aa, >antibacterial08436... at 46.67%
21 12aa, >antibacterial08500... at 41.67%
22 12aa, >antibacterial08966... at 41.67%
23 12aa, >antibacterial08967... at 50.00%
24 18aa, >antibacterial09194... at 44.44%
25 13aa, >antibacterial09590... *
26 14aa, >antibacterial09940... at 42.86%

```
27    12aa, >antibacterial10067... at 41.67%
28    43aa, >antibacterial10740... at 41.86%
29    19aa, >antibacterial10855... at 42.11%
30    15aa, >antibacterial10905... at 35.00%
31    20aa, >antibacterial11417... at 35.00%
32    17aa, >antibacterial12920... at 41.18%
>Cluster 59
0     12aa, >antibacterial;antim... *
1     23aa, >antimicrobial... at 43.48%
2     24aa, >antibacterial;antif... at 45.83%
3     24aa, >antimicrobial... at 41.67%
4     24aa, >antibacterial;Antif... *
5     24aa, >Antibacterial;Antif... at 41.67%
6     24aa, >Antibacterial;Antif... at 41.67%
7     24aa, >antibacterial;antim... at 41.67%
8     21aa, >anti-Gram+;Antibact... at 42.86%
9     21aa, >antimicrobial... at 42.86%
10    17aa, >antimicrobial... at 41.18%
11    255aa, >Antibacterial... *
12    32aa, >Antimicrobial... at 40.62%
13    16aa, >antimicrobial... at 43.75%
14    16aa, >antimicrobial... at 50.00%
15    16aa, >antimicrobial... at 43.75%
16    16aa, >antimicrobial... at 43.75%
17    13aa, >antibacterial07455... at 46.15%
18    18aa, >antibacterial08652... at 55.56%
19    11aa, >antibacterial09187... at 45.45%
20    12aa, >antibacterial10130... at 41.67%
21    13aa, >antibacterial10714... at 43.85%
22    14aa, >antibacterial11639... at 50.00%
23    18aa, >antibacterial11834... at 44.44%
24    15aa, >antibacterial11990... *
>Cluster 60
0     119aa, >Antimicrobial... at 47.06%
1     134aa, >Antimicrobial... at 40.30%
2     255aa, >Antimicrobial... *
3     126aa, >Antimicrobial... at 41.27%
4     255aa, >Antimicrobial... at 58.04%
5     255aa, >Antimicrobial... at 59.22%
6     255aa, >Antimicrobial... *
7     20aa, >Antibacterial... at 35.00%
8     30aa, >antibacterial;Antif... at 35.00%
9     255aa, >Antimicrobial... at 58.82%
10    255aa, >Antimicrobial... at 59.61%
11    255aa, >Antimicrobial... at 47.45%
12    32aa, >Anti-Gram-... at 40.62%
13    20aa, >antibacterial07621... at 35.00%
14    20aa, >antibacterial07752... at 45.00%
15    15aa, >antibacterial07976... at 35.00%
16    16aa, >antibacterial08407... at 43.75%
17    14aa, >antibacterial08498... at 42.86%
18    12aa, >antibacterial08499... at 41.67%
19    12aa, >antibacterial08501... at 41.67%
20    19aa, >antibacterial09211... at 47.37%
```

```
21    12aa, >antibacterial10057... at 41.67%
22    15aa, >antibacterial10585... at 35.00%
23    20aa, >antibacterial10960... at 35.00%
24    15aa, >antibacterial11571... at 35.00%
25    18aa, >antibacterial11895... at 44.44%
26    19aa, >antibacterial11934... at 42.11%
27    20aa, >antibacterial12484... at 35.00%
28    20aa, >antibacterial12487... at 35.00%
29    20aa, >antibacterial12490... at 35.00%
30    20aa, >antibacterial12492... at 35.00%
31    20aa, >antibacterial12494... at 35.00%
32    20aa, >antibacterial12496... at 35.00%
33    13aa, >antibacterial13289... *
>Cluster 61
0     24aa, >antimicrobial... at 45.83%
1     27aa, >antimicrobial... at 48.15%
2     21aa, >anti-Gram+;Gram-... at 47.62%
3     13aa, >anti-Gram+;Antibact... at 46.15%
4     27aa, >antimicrobial... at 40.74%
5     23aa, >antimicrobial... *
6     21aa, >antimicrobial... at 42.86%
7     23aa, >Antibacterial... at 43.48%
8     255aa, >Antimicrobial... *
9     11aa, >antibacterial;antim... at 45.45%
10    11aa, >antibacterial;antim... at 45.45%
11    24aa, >antimicrobial... at 41.67%
12    18aa, >antibacterial;antim... at 44.44%
13    25aa, >antibacterial07273... at 35.00%
14    25aa, >antibacterial07278... at 35.00%
15    20aa, >antibacterial07296... at 35.00%
16    20aa, >antibacterial07354... at 35.00%
17    20aa, >antibacterial07356... at 35.00%
18    21aa, >antibacterial07493... at 42.86%
19    14aa, >antibacterial08994... at 42.86%
20    20aa, >antibacterial09769... at 45.00%
21    15aa, >antibacterial09840... at 35.00%
22    14aa, >antibacterial10204... at 42.86%
23    22aa, >antibacterial10350... at 40.91%
24    18aa, >antibacterial10354... *
25    20aa, >antibacterial11316... at 35.00%
26    18aa, >antibacterial11330... at 44.44%
27    18aa, >antibacterial11492... at 50.00%
28    13aa, >antibacterial12275... at 46.15%
29    20aa, >antibacterial12482... at 45.00%
30    18aa, >antibacterial12532... at 50.00%
31    15aa, >antibacterial12838... at 35.00%
32    21aa, >antibacterial13018... *
33    20aa, >antibacterial13026... at 35.00%
34    12aa, >antibacterial13390... at 41.67%
>Cluster 62
0     24aa, >anti-Gram+;antibact... at 41.67%
1     27aa, >antifungal;antimicr... at 40.74%
2     25aa, >antimicrobial... at 48.00%
3     33aa, >antimicrobial... at 42.42%
```

```
4     29aa, >Antimicrobial... at 41.38%
5     28aa, >anti-Gram+;Antibact... at 42.86%
6     22aa, >antibacterial;antim... *
7     21aa, >antimicrobial... at 47.62%
8     255aa, >Antimicrobial... *
9     25aa, >antibacterial;antim... at 35.00%
10    12aa, >antimicrobial... at 41.67%
11    13aa, >antibacterial07705... at 84.62%
12    18aa, >antibacterial08973... at 50.00%
13    16aa, >antibacterial10495... *
14    13aa, >antibacterial12249... at 46.15%
15    13aa, >antibacterial12314... at 46.15%
16    15aa, >antibacterial12728... at 35.00%
17    21aa, >antibacterial13259... at 42.86%
>Cluster 63
0     20aa, >anti-Gram+;antibact... at 45.00%
1     24aa, >antimicrobial... at 41.67%
2     19aa, >Antimicrobial... at 47.37%
3     19aa, >Antimicrobial... at 42.11%
4     21aa, >antimicrobial... at 42.86%
5     36aa, >antimicrobial... at 47.22%
6     23aa, >anti-Gram+;antibact... at 43.48%
7     23aa, >Antimicrobial... *
8     23aa, >antimicrobial... at 47.83%
9     21aa, >antimicrobial... at 42.86%
10    21aa, >anti-Gram-;antibact... at 52.38%
11    21aa, >antimicrobial... at 52.38%
12    23aa, >antimicrobial... at 47.83%
13    28aa, >antimicrobial... at 46.43%
14    21aa, >antimicrobial... *
15    23aa, >antibacterial;Antif... at 47.83%
16    21aa, >antimicrobial... at 42.86%
17    38aa, >anti-Gram-;anti-HIV... at 44.74%
18    11aa, >Antifungal;antimicr... at 45.45%
19    255aa, >Antimicrobial... *
20    17aa, >Antibacterial... at 41.18%
21    33aa, >antibacterial06658... at 42.42%
22    27aa, >antibacterial07086... at 40.74%
23    44aa, >antibacterial07327... at 40.91%
24    21aa, >antibacterial07436... at 42.86%
25    11aa, >antibacterial08223... at 45.45%
26    11aa, >antibacterial08228... at 45.45%
27    16aa, >antibacterial08487... *
28    23aa, >antibacterial08514... at 43.48%
29    18aa, >antibacterial08594... at 44.44%
30    14aa, >antibacterial08644... at 42.86%
31    13aa, >antibacterial08904... at 46.15%
32    20aa, >antibacterial09114... at 35.00%
33    20aa, >antibacterial09115... at 35.00%
34    20aa, >antibacterial09119... at 35.00%
35    15aa, >antibacterial09552... at 35.00%
36    15aa, >antibacterial09605... at 35.00%
37    15aa, >antibacterial09609... at 35.00%
38    12aa, >antibacterial09961... at 41.67%
```

```
39    26aa, >antibacterial10103... at 42.31%
40    24aa, >antibacterial10194... at 41.67%
41    20aa, >antibacterial10227... at 35.00%
42    21aa, >antibacterial10286... *
43    21aa, >antibacterial10288... at 47.62%
44    26aa, >antibacterial10369... at 42.31%
45    21aa, >antibacterial10473... at 47.62%
46    14aa, >antibacterial10475... at 42.86%
47    25aa, >antibacterial10579... at 35.00%
48    25aa, >antibacterial11398... at 35.00%
49    25aa, >antibacterial11423... at 35.00%
50    21aa, >antibacterial11781... at 42.86%
51    23aa, >antibacterial11804... at 43.48%
52    23aa, >antibacterial11807... at 43.48%
53    27aa, >antibacterial11865... at 40.74%
54    19aa, >antibacterial11880... at 47.37%
55    12aa, >antibacterial11978... at 41.67%
56    20aa, >antibacterial12031... at 35.00%
57    18aa, >antibacterial12610... at 44.44%
58    14aa, >antibacterial12883... at 42.86%
59    20aa, >antibacterial13287... at 35.00%
60    14aa, >antibacterial13322... at 42.86%
61    20aa, >antibacterial13355... at 35.00%
62    36aa, >antibacterial13383... *
63    20aa, >antibacterial13389... at 35.00%
>Cluster 64
0     26aa, >Antimicrobial... *
1     11aa, >antibacterial... at 45.45%
2     12aa, >anti-Gram+;antibact... at 41.67%
3     13aa, >antimicrobial... at 46.15%
4     18aa, >antibacterial;Antif... at 44.44%
5     14aa, >antimicrobial... at 42.86%
6     255aa, >Antimicrobial... *
7     18aa, >antibacterial;Antif... at 44.44%
8     15aa, >antimicrobial... at 35.00%
9     15aa, >antimicrobial... at 35.00%
10    19aa, >antibacterial07299... at 42.11%
11    18aa, >antibacterial07334... at 44.44%
12    18aa, >antibacterial07335... at 50.00%
13    18aa, >antibacterial07336... at 44.44%
14    17aa, >antibacterial08329... at 41.18%
15    15aa, >antibacterial08531... at 35.00%
16    12aa, >antibacterial09257... at 41.67%
17    20aa, >antibacterial09401... at 35.00%
18    20aa, >antibacterial09931... at 35.00%
19    17aa, >antibacterial10626... at 41.18%
20    13aa, >antibacterial12040... at 46.15%
21    15aa, >antibacterial12113... at 35.00%
22    13aa, >antibacterial12676... *
>Cluster 65
0     11aa, >Antibacterial... at 45.45%
1     21aa, >antimicrobial... at 42.86%
2     30aa, >Antimicrobial... at 35.00%
3     20aa, >Antibacterial;Antif... at 55.00%
```

4 24aa, >antibacterial;antim... at 41.67%
5 25aa, >antibacterial;Antif... at 44.00%
6 255aa, >Antimicrobial... *
7 21aa, >antibacterial07369... at 42.86%
8 22aa, >antibacterial07381... at 45.45%
9 12aa, >antibacterial08116... at 41.67%
10 13aa, >antibacterial08161... at 46.15%
11 24aa, >antibacterial08278... at 41.67%
12 11aa, >antibacterial08519... at 45.45%
13 11aa, >antibacterial08520... at 45.45%
14 19aa, >antibacterial08535... at 42.11%
15 14aa, >antibacterial09070... at 42.86%
16 14aa, >antibacterial09259... at 42.86%
17 16aa, >antibacterial09846... at 43.75%
18 19aa, >antibacterial10100... at 42.11%
19 14aa, >antibacterial11951... *
20 18aa, >antibacterial12576... at 44.44%
21 20aa, >antibacterial12621... at 45.00%
22 18aa, >antibacterial12688... at 44.44%
23 20aa, >antibacterial12727... at 45.00%
24 20aa, >antibacterial12953... at 35.00%
25 18aa, >antibacterial13096... *
26 12aa, >antibacterial13411... at 50.00%
>Cluster 66
0 13aa, >anti-Gram+;Antimicr... *
1 20aa, >Antibacterial... at 35.00%
2 20aa, >anti-Gram+;Antibact... at 35.00%
3 12aa, >Antibacterial... at 41.67%
4 17aa, >anti-Gram+;Antibact... at 41.18%
5 28aa, >anti-Gram+;antibact... at 42.86%
6 11aa, >antibacterial;Antif... at 45.45%
7 255aa, >Antimicrobial... *
8 22aa, >antimicrobial... at 40.91%
9 215aa, >Antimicrobial... *
10 33aa, >Antimicrobial... at 48.48%
11 23aa, >antibacterial06453... at 43.48%
12 14aa, >antibacterial07518... at 42.86%
13 20aa, >antibacterial07537... at 35.00%
14 24aa, >antibacterial07723... at 41.67%
15 24aa, >antibacterial07724... at 41.67%
16 13aa, >antibacterial08080... at 46.15%
17 12aa, >antibacterial08120... *
18 15aa, >antibacterial08648... at 35.00%
19 12aa, >antibacterial08952... at 41.67%
20 19aa, >antibacterial09291... at 42.11%
21 11aa, >antibacterial09320... at 54.55%
22 18aa, >antibacterial09897... at 44.44%
23 11aa, >antibacterial10560... at 45.45%
24 17aa, >antibacterial10719... at 58.82%
25 22aa, >antibacterial10724... at 45.45%
26 14aa, >antibacterial12083... at 50.00%
27 12aa, >antibacterial12529... at 50.00%
28 17aa, >antibacterial12696... at 41.18%
29 22aa, >antibacterial12720... at 45.45%

30 16aa, >antibacterial13016... at 43.75%
31 30aa, >antibacterial13057... at 43.33%
32 19aa, >antibacterial13111... at 42.11%
33 19aa, >antibacterial13133... at 42.11%
34 20aa, >antibacterial13362... at 35.00%
>Cluster 67
0 23aa, >antibacterial;antif... *
1 33aa, >antibacterial;antif... at 42.42%
2 22aa, >antimicrobial... at 45.45%
3 255aa, >Antimicrobial... *
4 29aa, >antimicrobial... at 41.38%
5 23aa, >antibacterial08217... at 43.48%
6 18aa, >antibacterial09158... at 44.44%
7 28aa, >antibacterial09929... at 42.86%
8 18aa, >antibacterial10783... at 44.44%
9 25aa, >antibacterial11381... at 35.00%
10 12aa, >antibacterial11959... at 41.67%
11 20aa, >antibacterial12780... at 45.00%
12 20aa, >antibacterial12936... at 35.00%
13 12aa, >antibacterial13084... at 41.67%
14 38aa, >antibacterial13200... at 42.11%
>Cluster 68
0 13aa, >antimicrobial... *
1 19aa, >anti-Gram+;antibact... at 42.11%
2 27aa, >antimicrobial... at 40.74%
3 19aa, >anti-Gram+;Antimicr... at 42.11%
4 33aa, >anti-Gram+;Antibact... at 42.42%
5 25aa, >antibiotic... at 35.00%
6 255aa, >Antimicrobial... *
7 18aa, >Antimicrobial... at 44.44%
8 12aa, >antifungal;antimicr... at 41.67%
9 15aa, >antimicrobial... at 35.00%
10 17aa, >antibacterial06529... at 41.18%
11 11aa, >antibacterial07308... at 45.45%
12 17aa, >antibacterial07749... at 41.18%
13 16aa, >antibacterial08060... at 43.75%
14 22aa, >antibacterial08730... at 40.91%
15 12aa, >antibacterial08788... at 41.67%
16 20aa, >antibacterial09095... at 35.00%
17 14aa, >antibacterial10717... *
18 15aa, >antibacterial11478... at 35.00%
19 17aa, >antibacterial12022... at 47.06%
20 13aa, >antibacterial12291... at 46.15%
21 20aa, >antibacterial12884... at 45.00%
>Cluster 69
0 16aa, >antibacterial;antim... at 43.75%
1 24aa, >antimicrobial... at 45.83%
2 24aa, >antimicrobial... at 41.67%
3 20aa, >antimicrobial... at 35.00%
4 23aa, >antimicrobial... at 43.48%
5 24aa, >antibacterial;antim... at 41.67%
6 24aa, >active... at 45.83%
7 24aa, >antibacterial;Antif... at 45.83%
8 13aa, >antibacterial;antif... at 46.15%

```
9     255aa, >Antimicrobial... *
10    13aa, >Antimicrobial... at 46.15%
11    14aa, >antibacterial07027... at 42.86%
12    13aa, >antibacterial08409... at 46.15%
13    14aa, >antibacterial09260... at 50.00%
14    11aa, >antibacterial09310... at 45.45%
15    20aa, >antibacterial10034... at 35.00%
16    13aa, >antibacterial10462... *
17    13aa, >antibacterial10470... at 46.15%
18    23aa, >antibacterial10664... at 43.48%
19    20aa, >antibacterial10955... at 45.00%
20    18aa, >antibacterial11186... at 44.44%
21    13aa, >antibacterial11950... at 46.15%
22    21aa, >antibacterial12337... at 42.86%
23    26aa, >antibacterial12350... at 46.15%
24    15aa, >antibacterial12870... at 35.00%
25    12aa, >antibacterial12882... at 41.67%
26    15aa, >antibacterial13386... *
>Cluster 70
0     21aa, >antimicrobial... at 42.86%
1     31aa, >antimicrobial... at 41.94%
2     31aa, >antimicrobial... at 41.94%
3     30aa, >Antimicrobial;Antim... at 35.00%
4     30aa, >Antimicrobial;Antim... at 35.00%
5     23aa, >antimicrobial... *
6     30aa, >antimicrobial... at 35.00%
7     23aa, >Antimicrobial;Gram-... at 47.83%
8     13aa, >antimicrobial... at 46.15%
9     255aa, >Antimicrobial... *
10    15aa, >antibacterial06993... *
11    16aa, >antibacterial07007... at 43.75%
12    15aa, >antibacterial07522... at 35.00%
13    15aa, >antibacterial07913... at 35.00%
14    18aa, >antibacterial09198... at 44.44%
15    15aa, >antibacterial09736... at 35.00%
16    17aa, >antibacterial10447... at 41.18%
17    13aa, >antibacterial12064... at 46.15%
18    20aa, >antibacterial12377... at 35.00%
19    12aa, >antibacterial12624... at 41.67%
20    13aa, >antibacterial12813... at 46.15%
21    12aa, >antibacterial12859... at 41.67%
22    13aa, >antibacterial12982... at 53.85%
23    16aa, >antibacterial13227... at 50.00%
>Cluster 71
0     22aa, >anti-Gram+;antibact... at 40.91%
1     26aa, >antimicrobial... at 42.31%
2     255aa, >Antibacterial... *
3     64aa, >antibacterial06502... at 40.62%
4     15aa, >antibacterial07010... at 35.00%
5     19aa, >antibacterial08232... at 52.63%
6     20aa, >antibacterial08250... at 35.00%
7     24aa, >antibacterial08346... at 41.67%
8     19aa, >antibacterial08853... at 57.89%
9     19aa, >antibacterial08854... at 57.89%
```

```
10    19aa, >antibacterial08856... at 57.89%
11    19aa, >antibacterial08868... *
12    21aa, >antibacterial08870... at 63.16%
13    21aa, >antibacterial08874... at 52.63%
14    19aa, >antibacterial08876... at 57.89%
15    18aa, >antibacterial08877... at 57.89%
16    18aa, >antibacterial08943... *
17    18aa, >antibacterial09066... at 61.11%
18    21aa, >antibacterial09067... at 57.14%
19    19aa, >antibacterial09068... at 63.16%
20    18aa, >antibacterial09192... at 55.56%
21    18aa, >antibacterial09193... at 61.11%
22    18aa, >antibacterial09196... at 61.11%
23    28aa, >antibacterial09209... at 46.43%
24    26aa, >antibacterial09210... at 46.15%
25    20aa, >antibacterial09334... at 35.00%
26    13aa, >antibacterial09594... at 46.15%
27    13aa, >antibacterial09596... at 61.54%
28    18aa, >antibacterial09777... at 61.11%
29    18aa, >antibacterial09778... at 61.11%
30    18aa, >antibacterial09781... at 61.11%
31    16aa, >antibacterial10090... at 68.75%
32    19aa, >antibacterial10092... at 47.37%
33    19aa, >antibacterial10093... at 47.37%
34    19aa, >antibacterial10094... at 42.11%
35    19aa, >antibacterial10095... at 47.37%
36    19aa, >antibacterial10096... *
37    19aa, >antibacterial10097... at 52.63%
38    19aa, >antibacterial10098... at 57.89%
39    19aa, >antibacterial10099... at 52.63%
40    19aa, >antibacterial10101... at 57.89%
41    20aa, >antibacterial10143... *
42    19aa, >antibacterial10145... at 63.16%
43    23aa, >antibacterial10401... at 43.48%
44    23aa, >antibacterial10403... at 43.48%
45    20aa, >antibacterial10404... *
46    21aa, >antibacterial10405... at 61.90%
47    21aa, >antibacterial10406... at 57.14%
48    15aa, >antibacterial10584... at 35.00%
49    22aa, >antibacterial10653... at 54.55%
50    23aa, >antibacterial10654... at 54.55%
51    19aa, >antibacterial10668... at 52.63%
52    17aa, >antibacterial10892... at 47.06%
53    13aa, >antibacterial12811... at 46.15%
54    13aa, >antibacterial12812... at 46.15%
55    13aa, >antibacterial12814... at 53.85%
56    13aa, >antibacterial12815... at 53.85%
57    15aa, >antibacterial12819... at 53.33%
58    21aa, >antibacterial12997... at 42.86%
59    19aa, >antibacterial13228... at 63.16%
>Cluster 72
0     16aa, >antimicrobial... at 43.75%
1     11aa, >Antimicrobial;Antim... at 45.45%
2     18aa, >anti-Gram+;antibact... at 44.44%
```

```
3     22aa, >antimicrobial... at 40.91%
4     15aa, >Antibacterial;Antim... at 53.33%
5     255aa, >Antibacterial... *
6     255aa, >Antibacterial... at 66.08%
7     23aa, >antibacterial06650... at 43.48%
8     20aa, >antibacterial06653... at 35.00%
9     11aa, >antibacterial09625... at 45.45%
10    12aa, >antibacterial09784... at 41.67%
11    12aa, >antibacterial09786... at 41.67%
12    12aa, >antibacterial09787... at 50.00%
13    12aa, >antibacterial09788... at 41.67%
14    11aa, >antibacterial09830... *
15    20aa, >antibacterial09996... at 35.00%
>Cluster 73
0     21aa, >antimicrobial... at 42.86%
1     20aa, >antimicrobial... at 35.00%
2     18aa, >antimicrobial... at 44.44%
3     22aa, >antimicrobial... at 45.45%
4     25aa, >antimicrobial... at 44.00%
5     26aa, >Antimicrobial... at 42.31%
6     27aa, >anti-Gram+;antibact... at 40.74%
7     25aa, >antibacterial;Antif... at 35.00%
8     18aa, >anti-Gram+;antibact... *
9     28aa, >anti-Gram+;antibact... at 46.43%
10    29aa, >anti-Gram+;antibact... at 41.38%
11    28aa, >antimicrobial... at 46.43%
12    255aa, >Antimicrobial... *
13    20aa, >antimicrobial... at 35.00%
14    30aa, >Antimicrobial... at 35.00%
15    18aa, >antibacterial07725... at 44.44%
16    20aa, >antibacterial07743... at 45.00%
17    20aa, >antibacterial07957... at 35.00%
18    17aa, >antibacterial08092... *
19    18aa, >antibacterial08231... at 44.44%
20    20aa, >antibacterial08388... at 35.00%
21    28aa, >antibacterial08819... at 42.86%
22    12aa, >antibacterial09959... at 41.67%
23    12aa, >antibacterial09960... at 41.67%
24    19aa, >antibacterial11130... at 42.11%
25    20aa, >antibacterial11364... at 50.00%
26    27aa, >antibacterial11873... *
27    27aa, >antibacterial11874... at 40.74%
28    18aa, >antibacterial12144... at 44.44%
29    23aa, >antibacterial12764... at 43.48%
30    27aa, >antibacterial13295... at 44.44%
>Cluster 74
0     26aa, >anti-Gram+;antibact... at 42.31%
1     27aa, >antibacterial... at 44.44%
2     255aa, >Antimicrobial... *
3     23aa, >antimicrobial... at 43.48%
4     15aa, >antimicrobial... at 35.00%
5     14aa, >antibacterial06992... at 42.86%
6     12aa, >antibacterial07013... at 41.67%
7     26aa, >antibacterial07060... at 42.31%
```

8   25aa, >antibacterial08072... at 35.00%
9   12aa, >antibacterial09258... at 41.67%
10   11aa, >antibacterial09575... at 45.45%
11   13aa, >antibacterial09602... *
12   13aa, >antibacterial10283... at 46.15%
13   11aa, >antibacterial10594... at 45.45%
14   22aa, >antibacterial11254... at 45.45%
15   18aa, >antibacterial11818... at 44.44%
16   20aa, >antibacterial11883... at 35.00%
17   18aa, >antibacterial12893... at 44.44%
>Cluster 75
0   18aa, >antimicrobial... *
1   15aa, >antimicrobial... at 35.00%
2   255aa, >Antimicrobial... *
3   19aa, >antibacterial08861... at 47.37%
4   14aa, >antibacterial10120... at 42.86%
5   15aa, >antibacterial10421... at 46.67%
6   18aa, >antibacterial12900... at 44.44%
>Cluster 76
0   17aa, >antibacterial;antim... at 41.18%
1   26aa, >antibacterial... *
2   18aa, >Antimicrobial... *
3   24aa, >antibacterial;Antif... at 45.83%
4   25aa, >antibacterial;Antif... at 35.00%
5   16aa, >Antimicrobial... at 43.75%
6   14aa, >anti-Gram+;antibact... at 42.86%
7   25aa, >Antimicrobial... at 35.00%
8   28aa, >Antibacterial... at 46.43%
9   36aa, >antibacterial;antim... at 41.67%
10   255aa, >Antimicrobial... *
11   17aa, >Antifungal... at 47.06%
12   26aa, >anti-Gram+;Gram-... at 42.31%
13   13aa, >antibacterial... at 46.15%
14   13aa, >antibacterial07400... at 46.15%
15   22aa, >antibacterial07609... at 40.91%
16   11aa, >antibacterial07874... at 45.45%
17   20aa, >antibacterial08046... at 35.00%
18   19aa, >antibacterial08051... at 47.37%
19   20aa, >antibacterial09427... at 35.00%
20   24aa, >antibacterial09533... at 41.67%
21   18aa, >antibacterial09718... at 44.44%
22   15aa, >antibacterial09839... at 46.67%
23   18aa, >antibacterial12120... at 44.44%
>Cluster 77
0   14aa, >antibacterial;Antif... *
1   32aa, >antimicrobial... at 43.75%
2   30aa, >antimicrobial... at 35.00%
3   27aa, >antibacterial;antim... at 40.74%
4   35aa, >Antifungal;antimicr... at 54.29%
5   17aa, >anti-Gram+;Antimicr... at 41.18%
6   255aa, >Antimicrobial... *
7   13aa, >anti-Gram+;antibact... at 53.85%
8   29aa, >antibacterial06654... at 32.38%
9   26aa, >antibacterial07529... at 42.31%

10   18aa, >antibacterial08003... at 44.44%
11   25aa, >antibacterial08005... at 35.00%
12   13aa, >antibacterial08176... at 46.15%
13   20aa, >antibacterial09098... at 50.00%
14   12aa, >antibacterial09519... at 41.67%
15   21aa, >antibacterial10343... at 47.62%
16   21aa, >antibacterial10456... at 42.86%
17   19aa, >antibacterial10516... at 47.37%
18   14aa, >antibacterial10640... at 50.00%
19   20aa, >antibacterial10738... at 45.00%
20   20aa, >antibacterial10739... at 45.00%
21   15aa, >antibacterial11295... at 35.00%
22   21aa, >antibacterial11668... at 52.38%
23   15aa, >antibacterial11738... at 35.00%
24   17aa, >antibacterial11926... at 41.18%
25   17aa, >antibacterial11975... at 41.18%
26   15aa, >antibacterial11998... at 35.00%
27   15aa, >antibacterial12334... at 35.00%
28   20aa, >antibacterial12480... at 35.00%
29   20aa, >antibacterial12485... at 35.00%
30   20aa, >antibacterial12486... at 35.00%
31   15aa, >antibacterial13085... *
32   15aa, >antibacterial13180... at 46.67%
33   20aa, >antibacterial13367... at 35.00%
>Cluster 78
0   27aa, >anti-Gram+;antibact... at 40.74%
1   16aa, >anti-Gram+;antibact... at 43.75%
2   16aa, >antimicrobial... at 43.75%
3   24aa, >antimicrobial... at 41.67%
4   14aa, >anti-Gram+;Antibact... at 50.00%
5   255aa, >Antimicrobial... *
6   37aa, >anti-Gram+;antibact... at 40.54%
7   11aa, >antibacterial07857... at 45.45%
8   11aa, >antibacterial07867... at 45.45%
9   15aa, >antibacterial07977... at 35.00%
10   18aa, >antibacterial08509... at 44.44%
11   19aa, >antibacterial08666... *
12   19aa, >antibacterial11628... at 47.37%
13   18aa, >antibacterial13123... at 44.44%
>Cluster 79
0   13aa, >antimicrobial... *
1   27aa, >anti-Gram+;antibact... at 40.74%
2   29aa, >antimicrobial... at 41.38%
3   27aa, >antibacterial;Antif... at 40.74%
4   22aa, >Antibacterial;Antim... at 40.91%
5   26aa, >antimicrobial... at 46.15%
6   31aa, >antimicrobial... at 41.94%
7   23aa, >antimicrobial... at 43.48%
8   25aa, >antibacterial;antim... at 35.00%
9   25aa, >Antimicrobial... at 35.00%
10   255aa, >Antimicrobial... *
11   255aa, >Antimicrobial... at 67.06%
12   255aa, >Antibacterial... at 71.76%
13   18aa, >antibacterial07556... at 44.44%

```
14    20aa, >antibacterial08546... at 35.00%
15    20aa, >antibacterial09426... at 35.00%
16    16aa, >antibacterial10496... at 43.75%
17    25aa, >antibacterial10580... at 35.00%
18    20aa, >antibacterial10950... at 35.00%
19    16aa, >antibacterial11279... at 43.75%
20    27aa, >antibacterial11802... at 40.74%
21    18aa, >antibacterial12843... at 44.44%
>Cluster 80
0     24aa, >antimicrobial... at 41.67%
1     20aa, >antimicrobial... at 35.00%
2     21aa, >anti-Gram+;antibact... *
3     24aa, >antibacterial;Antif... at 41.67%
4     24aa, >antimicrobial... at 45.83%
5     15aa, >antimicrobial... at 35.00%
6     25aa, >Antimicrobial... at 44.00%
7     11aa, >antimicrobial... at 45.45%
8     255aa, >Antimicrobial... *
9     22aa, >anti-Gram-;antibact... at 40.91%
10    69aa, >Antimicrobial... *
11    25aa, >antibacterial06532... at 35.00%
12    20aa, >antibacterial07367... at 35.00%
13    20aa, >antibacterial07751... at 35.00%
14    17aa, >antibacterial08091... at 47.06%
15    18aa, >antibacterial08102... at 44.44%
16    18aa, >antibacterial08105... at 44.44%
17    32aa, >antibacterial08200... at 40.62%
18    25aa, >antibacterial08202... at 35.00%
19    17aa, >antibacterial08258... *
20    17aa, >antibacterial08330... at 52.94%
21    17aa, >antibacterial08336... at 47.06%
22    18aa, >antibacterial08340... at 44.44%
23    18aa, >antibacterial08341... *
24    17aa, >antibacterial08351... at 52.94%
25    17aa, >antibacterial08353... at 47.06%
26    21aa, >antibacterial08451... at 47.62%
27    15aa, >antibacterial08686... at 35.00%
28    15aa, >antibacterial08759... at 35.00%
29    11aa, >antibacterial08908... at 45.45%
30    27aa, >antibacterial08927... at 40.74%
31    12aa, >antibacterial08962... at 41.67%
32    20aa, >antibacterial09112... at 35.00%
33    18aa, >antibacterial09162... at 50.00%
34    11aa, >antibacterial09168... at 54.55%
35    11aa, >antibacterial09188... at 54.55%
36    11aa, >antibacterial09338... at 45.45%
37    29aa, >antibacterial09505... at 41.38%
38    24aa, >antibacterial09532... at 41.67%
39    25aa, >antibacterial09534... at 35.00%
40    25aa, >antibacterial09536... at 44.00%
41    15aa, >antibacterial09713... at 35.00%
42    16aa, >antibacterial10280... at 43.75%
43    26aa, >antibacterial10293... at 42.31%
44    22aa, >antibacterial10303... at 40.91%
```

45 18aa, >antibacterial10414... at 44.44%
46 13aa, >antibacterial10513... at 46.15%
47 13aa, >antibacterial10549... at 46.15%
48 14aa, >antibacterial10554... at 42.86%
49 18aa, >antibacterial10573... at 44.44%
50 11aa, >antibacterial10672... at 45.45%
51 11aa, >antibacterial10673... at 45.45%
52 15aa, >antibacterial10796... at 35.00%
53 13aa, >antibacterial10838... *
54 20aa, >antibacterial10842... at 35.00%
55 25aa, >antibacterial10852... at 48.00%
56 26aa, >antibacterial11613... at 50.00%
57 27aa, >antibacterial11669... at 40.74%
58 15aa, >antibacterial12599... at 35.00%
>Cluster 81
0 68aa, >anti-Gram+;Gram-... *
1 24aa, >anti-Gram+;Antibact... at 45.83%
2 21aa, >antimicrobial... at 47.62%
3 20aa, >antimicrobial... at 35.00%
4 21aa, >antimicrobial... at 42.86%
5 27aa, >antibacterial;antim... at 40.74%
6 22aa, >antimicrobial... at 45.45%
7 27aa, >Antimicrobial... at 40.74%
8 248aa, >Antimicrobial... at 52.82%
9 248aa, >Antimicrobial... at 53.23%
10 133aa, >Antimicrobial... at 41.35%
11 255aa, >Antimicrobial... *
12 15aa, >Antibacterial... at 46.67%
13 13aa, >Antimicrobial... at 46.15%
14 21aa, >Antimicrobial... at 47.62%
15 17aa, >antibacterial08268... at 41.18%
16 20aa, >antibacterial09287... at 35.00%
17 20aa, >antibacterial09397... at 35.00%
18 15aa, >antibacterial11270... at 35.00%
19 15aa, >antibacterial12405... *
20 14aa, >antibacterial13074... at 42.86%
>Cluster 82
0 29aa, >anti-Gram+;antibact... at 34.83%
1 28aa, >antibacterial;antim... at 32.86%
2 28aa, >antimicrobial... at 32.86%
3 28aa, >anti-Gram-;antifung... at 42.86%
4 31aa, >antimicrobial... at 41.94%
5 20aa, >anti-Gram-;antibact... at 45.00%
6 255aa, >Antimicrobial... *
7 255aa, >Antimicrobial... at 70.98%
8 31aa, >antimicrobial... at 41.94%
9 26aa, >antibacterial06554... at 42.31%
10 12aa, >antibacterial06619... at 35.00%
11 21aa, >antibacterial06978... at 42.86%
12 18aa, >antibacterial07918... at 44.44%
13 17aa, >antibacterial08021... at 41.18%
14 13aa, >antibacterial08525... *
15 34aa, >antibacterial08881... at 44.12%
16 25aa, >antibacterial09265... at 35.00%

17   13aa, >antibacterial09282... at 53.85%
18   18aa, >antibacterial09710... at 44.44%
19   14aa, >antibacterial10200... at 42.86%
20   30aa, >antibacterial10255... at 30.00%
21   22aa, >antibacterial10727... at 30.91%
22   19aa, >antibacterial10762... at 32.11%
23   24aa, >antibacterial11678... at 31.67%
24   13aa, >antibacterial12154... at 36.15%
25   18aa, >antibacterial12538... *
26   16aa, >antibacterial12816... at 33.75%
27   18aa, >antibacterial12977... at 34.44%
>Cluster 83
0   40aa, >antimicrobial;Antim... at 42.50%
1   13aa, >antibacterial;antim... at 46.15%
2   19aa, >antibacterial;antif... at 42.11%
3   19aa, >Antimicrobial... *
4   24aa, >antimicrobial... at 41.67%
5   255aa, >Antimicrobial... *
6   18aa, >Antibacterial;Antif... at 44.44%
7   21aa, >antibacterial07898... at 47.62%
8   11aa, >antibacterial08220... at 45.45%
9   11aa, >antibacterial08565... at 54.55%
10   11aa, >antibacterial09626... at 45.45%
11   15aa, >antibacterial10348... at 35.00%
12   22aa, >antibacterial10523... at 40.91%
13   12aa, >antibacterial11786... *
14   15aa, >antibacterial11954... at 43.33%
15   13aa, >antibacterial12042... at 46.15%
16   17aa, >antibacterial12376... at 52.94%
17   18aa, >antibacterial12731... at 44.44%
18   17aa, >antibacterial13371... at 41.18%
>Cluster 84
0   20aa, >antimicrobial... *
1   255aa, >Antimicrobial... *
2   13aa, >antimicrobial... at 46.15%
3   23aa, >antimicrobial... at 43.48%
4   12aa, >antibacterial08027... at 41.67%
5   23aa, >antibacterial08719... at 43.48%
6   21aa, >antibacterial09182... at 42.86%
7   21aa, >antibacterial09195... at 42.86%
8   29aa, >antibacterial11118... at 41.38%
9   17aa, >antibacterial11734... at 41.18%
10   20aa, >antibacterial12825... at 45.00%
11   19aa, >antibacterial13132... at 42.11%
>Cluster 85
0   14aa, >antimicrobial... *
1   20aa, >antimicrobial... at 35.00%
2   17aa, >Antibacterial... at 41.18%
3   13aa, >Antibacterial... at 46.15%
4   255aa, >Antimicrobial... *
5   25aa, >antibacterial07748... at 35.00%
6   12aa, >antibacterial07901... *
7   11aa, >antibacterial08427... at 45.45%
8   21aa, >antibacterial09308... at 42.86%

```
9     11aa, >antibacterial09315... at 45.45%
10    20aa, >antibacterial09390... at 45.00%
11    14aa, >antibacterial09487... at 42.86%
12    13aa, >antibacterial09595... at 46.15%
13    13aa, >antibacterial09600... at 46.15%
14    13aa, >antibacterial10054... at 46.15%
15    15aa, >antibacterial10418... at 35.00%
16    28aa, >antibacterial10453... at 46.43%
17    13aa, >antibacterial10468... at 53.85%
18    13aa, >antibacterial10484... at 46.15%
19    13aa, >antibacterial10486... at 46.15%
20    12aa, >antibacterial10487... at 41.67%
21    14aa, >antibacterial10867... at 42.86%
22    18aa, >antibacterial11273... at 44.44%
23    12aa, >antibacterial11461... at 41.67%
24    13aa, >antibacterial11725... at 46.15%
25    13aa, >antibacterial12360... at 46.15%
26    15aa, >antibacterial13380... at 35.00%
>Cluster 86
0     19aa, >antibacterial;antif... at 42.11%
1     29aa, >Antimicrobial... at 44.83%
2     20aa, >Antibacterial... *
3     255aa, >Antimicrobial... *
4     18aa, >antibacterial07044... at 44.44%
5     13aa, >antibacterial07650... at 46.15%
6     11aa, >antibacterial07730... at 45.45%
7     20aa, >antibacterial07952... at 45.00%
8     13aa, >antibacterial08031... at 46.15%
9     13aa, >antibacterial08291... at 46.15%
10    22aa, >antibacterial08461... at 40.91%
11    13aa, >antibacterial08907... at 46.15%
12    11aa, >antibacterial09154... at 45.45%
13    11aa, >antibacterial09183... at 45.45%
14    20aa, >antibacterial09395... at 45.00%
15    20aa, >antibacterial09422... at 55.00%
16    20aa, >antibacterial09430... at 45.00%
17    27aa, >antibacterial09752... at 40.74%
18    18aa, >antibacterial11902... at 44.44%
19    22aa, >antibacterial11956... at 40.91%
20    24aa, >antibacterial12700... *
21    20aa, >antibacterial13307... at 35.00%
>Cluster 87
0     26aa, >antimicrobial... at 42.31%
1     24aa, >antimicrobial... at 43.83%
2     24aa, >antibacterial;Antif... at 41.67%
3     20aa, >antibacterial;antim... at 35.00%
4     24aa, >antimicrobial... at 41.67%
5     255aa, >Antimicrobial... *
6     30aa, >Antimicrobial... at 35.00%
7     21aa, >antimicrobial... at 42.86%
8     19aa, >anti-Gram+;antibact... at 42.11%
9     20aa, >antibacterial07357... at 35.00%
10    14aa, >antibacterial07984... at 42.86%
11    20aa, >antibacterial09423... *
```

```
12    20aa, >antibacterial09455... at 35.00%
13    17aa, >antibacterial11444... at 41.18%
14    11aa, >antibacterial11522... at 45.45%
15    36aa, >antibacterial12660... at 41.67%
>Cluster 88
0     30aa, >Antibacterial;Antim... at 35.00%
1     30aa, >Antifungal... at 35.00%
2     28aa, >anti-Gram+;antibact... at 42.86%
3     255aa, >Antimicrobial... *
4     255aa, >Antimicrobial... at 68.24%
5     255aa, >Antimicrobial... at 69.02%
6     21aa, >antibacterial07597... at 42.86%
7     21aa, >antibacterial07598... at 42.86%
8     21aa, >antibacterial07601... at 42.86%
9     11aa, >antibacterial09577... *
10    26aa, >antibacterial10102... at 42.31%
11    13aa, >antibacterial10425... at 46.15%
12    13aa, >antibacterial10435... at 46.15%
13    13aa, >antibacterial10553... at 53.85%
14    18aa, >antibacterial10787... at 44.44%
15    23aa, >antibacterial12831... at 43.48%
16    25aa, >antibacterial12841... at 35.00%
17    12aa, >antibacterial13281... at 41.67%
>Cluster 89
0     33aa, >antibacterial... at 42.42%
1     19aa, >Antibacterial;Antim... at 42.11%
2     17aa, >antimicrobial... *
3     23aa, >anti-Gram+;antibact... at 47.83%
4     255aa, >Antibacterial... *
5     21aa, >Antimicrobial... at 42.86%
6     255aa, >Antibacterial... at 47.06%
7     255aa, >Antibacterial... at 48.24%
8     255aa, >Antibacterial... at 61.18%
9     255aa, >Antibacterial... at 59.22%
10    11aa, >antibacterial06633... at 54.55%
11    18aa, >antibacterial06648... at 55.56%
12    15aa, >antibacterial08193... at 35.00%
13    12aa, >antibacterial08590... at 41.67%
14    12aa, >antibacterial08591... at 58.33%
15    12aa, >antibacterial08789... at 41.67%
16    13aa, >antibacterial08901... at 53.85%
17    15aa, >antibacterial09530... *
18    17aa, >antibacterial10442... at 41.18%
19    37aa, >antibacterial10683... at 40.54%
20    16aa, >antibacterial11113... at 43.75%
21    18aa, >antibacterial11137... at 44.44%
22    13aa, >antibacterial11304... at 46.15%
23    18aa, >antibacterial11314... at 44.44%
24    23aa, >antibacterial11328... at 47.83%
25    13aa, >antibacterial12361... at 53.85%
26    28aa, >antibacterial12744... at 42.86%
27    20aa, >antibacterial12932... at 35.00%
28    20aa, >antibacterial13189... at 45.00%
>Cluster 90
```

```
0     30aa, >antifungal;antimicr... at 35.00%
1     26aa, >antimicrobial... at 42.31%
2     241aa, >Antibacterial... *
3     255aa, >Antibacterial... *
4     11aa, >Antifungal... at 45.45%
5     20aa, >antibacterial06685... at 35.00%
6     27aa, >antibacterial07173... at 40.74%
7     37aa, >antibacterial07377... at 40.54%
8     24aa, >antibacterial07824... at 41.67%
9     18aa, >antibacterial09069... at 44.44%
10    13aa, >antibacterial09285... at 46.15%
11    27aa, >antibacterial09754... at 40.74%
12    23aa, >antibacterial10041... at 43.48%
13    27aa, >antibacterial11837... *
14    21aa, >antibacterial11843... at 47.62%
15    29aa, >antibacterial12863... at 44.83%
>Cluster 91
0     255aa, >Antibacterial;Antif... *
1     25aa, >antibacterial06964... at 35.00%
2     25aa, >antibacterial07379... at 35.00%
3     11aa, >antibacterial07737... at 45.45%
4     18aa, >antibacterial07887... at 44.44%
5     11aa, >antibacterial08225... at 45.45%
6     14aa, >antibacterial08449... at 42.86%
7     16aa, >antibacterial08845... at 43.75%
8     17aa, >antibacterial09169... at 47.06%
9     11aa, >antibacterial09350... *
10    20aa, >antibacterial09355... at 35.00%
11    19aa, >antibacterial09928... at 42.11%
12    12aa, >antibacterial10146... at 41.67%
13    12aa, >antibacterial10159... at 41.67%
14    15aa, >antibacterial10463... at 53.33%
15    15aa, >antibacterial10466... at 35.00%
>Cluster 92
0     24aa, >antimicrobial... at 41.67%
1     254aa, >Antimicrobial... *
2     13aa, >antibacterial;antim... *
3     24aa, >anti-Gram+;antibact... at 41.67%
4     24aa, >anti-Gram+;antibact... at 41.67%
5     24aa, >antimicrobial... at 45.83%
6     13aa, >antimicrobial... at 46.15%
7     13aa, >anti-Gram+;Antibact... at 46.15%
8     13aa, >antimicrobial... at 46.15%
9     27aa, >Antibacterial;Antif... at 40.74%
10    19aa, >anti-Gram+;Antibact... *
11    25aa, >anti-Gram+;anti-HIV... at 35.00%
12    25aa, >Antibacterial;antim... at 35.00%
13    26aa, >Antibacterial;antim... at 42.31%
14    21aa, >antibacterial;antim... *
15    28aa, >Antimicrobial... at 50.00%
16    31aa, >antibacterial;antif... at 41.94%
17    25aa, >anti-Gram+;Antibact... at 35.00%
18    26aa, >antibacterial;Antif... at 46.15%
19    27aa, >antibacterial;antim... at 44.44%
```

```
20    25aa, >anti-Gram-;antibact... at 35.00%
21    20aa, >antibacterial;Antif... at 35.00%
22    25aa, >antibacterial;antim... at 35.00%
23    20aa, >Antimicrobial... at 45.00%
24    22aa, >antibacterial06968... at 40.91%
25    18aa, >antibacterial08658... at 44.44%
26    24aa, >antibacterial08817... at 41.67%
27    26aa, >antibacterial09205... at 32.31%
28    26aa, >antibacterial09207... at 32.31%
29    27aa, >antibacterial09208... at 32.31%
30    12aa, >antibacterial09245... at 41.67%
31    18aa, >antibacterial09726... at 44.44%
32    11aa, >antibacterial09824... at 45.45%
33    20aa, >antibacterial09995... at 35.00%
34    14aa, >antibacterial10555... at 42.86%
35    14aa, >antibacterial10556... at 42.86%
36    30aa, >antibacterial11836... at 35.00%
>Cluster 93
0     11aa, >Anti-Gram-... *
1     37aa, >Antibacterial... at 40.54%
2     17aa, >Antibacterial;Antif... at 41.18%
3     18aa, >antibacterial;Antif... at 44.44%
4     21aa, >antimicrobial... at 42.86%
5     21aa, >antimicrobial... at 42.86%
6     18aa, >antibacterial;Antif... at 44.44%
7     22aa, >anti-Gram-;antifung... at 45.45%
8     252aa, >Antimicrobial... *
9     40aa, >Antibacterial;antim... at 85.00%
10    94aa, >anti-Gram+;Antibact... *
11    18aa, >antibacterial;antif... at 44.44%
12    18aa, >anti-Gram+;Gram-... at 44.44%
13    32aa, >Anti-Gram-... at 40.62%
14    22aa, >antibacterial06560... at 40.91%
15    18aa, >antibacterial06567... at 44.44%
16    14aa, >antibacterial06610... at 50.00%
17    18aa, >antibacterial06971... at 44.44%
18    15aa, >antibacterial06997... at 35.00%
19    12aa, >antibacterial07001... at 41.67%
20    15aa, >antibacterial07008... at 35.00%
21    12aa, >antibacterial07053... at 41.67%
22    15aa, >antibacterial07403... at 35.00%
23    18aa, >antibacterial07440... at 44.44%
24    18aa, >antibacterial07816... at 44.44%
25    12aa, >antibacterial08508... at 41.67%
26    20aa, >antibacterial08630... at 50.00%
27    18aa, >antibacterial09203... at 50.00%
28    13aa, >antibacterial09215... at 46.15%
29    13aa, >antibacterial09217... at 46.15%
30    26aa, >antibacterial09318... at 46.15%
31    18aa, >antibacterial09329... at 50.00%
32    13aa, >antibacterial09587... at 46.15%
33    13aa, >antibacterial09598... at 46.15%
34    13aa, >antibacterial09807... at 53.85%
35    13aa, >antibacterial09808... at 46.15%
```

```
36    13aa, >antibacterial09809... at 46.15%
37    13aa, >antibacterial09842... at 46.15%
38    18aa, >antibacterial09874... at 50.00%
39    13aa, >antibacterial09882... *
40    18aa, >antibacterial09942... at 50.00%
41    12aa, >antibacterial10245... at 48.33%
42    14aa, >antibacterial10897... at 42.86%
43    12aa, >antibacterial11428... at 41.67%
44    13aa, >antibacterial11746... at 46.15%
45    18aa, >antibacterial11773... at 44.44%
46    13aa, >antibacterial12037... *
47    13aa, >antibacterial12038... at 53.85%
48    13aa, >antibacterial12051... at 46.15%
49    13aa, >antibacterial12054... at 46.15%
50    13aa, >antibacterial12062... *
51    13aa, >antibacterial12063... at 46.15%
52    13aa, >antibacterial12395... at 46.15%
53    18aa, >antibacterial12650... at 44.44%
54    28aa, >antibacterial12756... at 42.86%
55    13aa, >antibacterial13327... at 46.15%
>Cluster 94
0     22aa, >Antifungal... at 45.45%
1     251aa, >Antimicrobial... *
2     18aa, >antimicrobial... at 50.00%
3     18aa, >antibacterial10056... at 44.44%
4     18aa, >antibacterial10965... at 44.44%
5     16aa, >antibacterial11114... at 43.75%
6     13aa, >antibacterial12146... at 36.15%
7     12aa, >antibacterial12332... at 46.15%
8     12aa, >antibacterial12881... at 41.67%
>Cluster 95
0     249aa, >Antibacterial;Antif... *
1     43aa, >antimicrobial... at 41.86%
2     26aa, >antimicrobial... at 50.00%
3     13aa, >antimicrobial... *
4     17aa, >antimicrobial... at 47.06%
5     23aa, >anti-Gram+;antimicr... at 43.48%
6     27aa, >antimicrobial... at 40.74%
7     14aa, >Antimicrobial... at 42.86%
8     13aa, >antibacterial06525... at 46.15%
9     18aa, >antibacterial08104... at 44.44%
10    11aa, >antibacterial09339... at 54.55%
11    13aa, >antibacterial09579... at 46.15%
12    18aa, >antibacterial09751... at 44.44%
13    18aa, >antibacterial10127... at 44.44%
14    22aa, >antibacterial10670... at 40.91%
15    19aa, >antibacterial10904... at 42.11%
16    26aa, >antibacterial11361... at 42.31%
17    15aa, >antibacterial11420... at 35.00%
18    14aa, >antibacterial11539... at 42.86%
19    14aa, >antibacterial11619... at 50.00%
20    13aa, >antibacterial11724... at 46.15%
21    20aa, >antibacterial11756... at 35.00%
22    14aa, >antibacterial12000... at 42.86%
```

```
23    12aa, >antibacterial12466... at 41.67%
24    18aa, >antibacterial12627... *
25    29aa, >antibacterial12757... at 44.83%
26    20aa, >antibacterial12933... at 35.00%
27    14aa, >antibacterial13125... at 42.86%
28    15aa, >antibacterial13184... at 35.00%
29    14aa, >antibacterial13372... at 42.86%
>Cluster 96
0     249aa, >Antifungal... *
1     22aa, >Antimicrobial... at 40.91%
2     24aa, >antibacterial;Antif... at 41.67%
3     13aa, >anti-Gram+;antibact... at 46.15%
4     26aa, >antibacterial07783... at 42.31%
5     20aa, >antibacterial09093... at 35.00%
6     20aa, >antibacterial09096... at 35.00%
7     14aa, >antibacterial09896... at 42.86%
8     13aa, >antibacterial10088... *
9     12aa, >antibacterial10134... at 41.67%
10    35aa, >antibacterial11309... at 45.71%
11    23aa, >antibacterial11848... at 43.48%
12    29aa, >antibacterial12508... at 41.38%
13    24aa, >antibacterial12513... at 41.67%
14    14aa, >antibacterial12528... at 42.86%
15    17aa, >antibacterial13050... at 41.18%
>Cluster 97
0     30aa, >anti-Gram+;antibact... at 35.00%
1     29aa, >Antimicrobial... at 41.38%
2     248aa, >Antimicrobial... *
3     234aa, >Antimicrobial... at 41.03%
4     234aa, >Antimicrobial... at 42.31%
5     33aa, >antimicrobial... at 45.45%
6     17aa, >antibacterial06566... at 47.06%
7     18aa, >antibacterial07922... *
8     24aa, >antibacterial10047... at 41.67%
9     19aa, >antibacterial11389... at 42.11%
10    24aa, >antibacterial11643... at 41.67%
>Cluster 98
0     21aa, >Antimicrobial... at 42.86%
1     23aa, >antimicrobial... *
2     13aa, >anti-Gram+;antibact... at 53.85%
3     24aa, >antimicrobial... at 41.67%
4     13aa, >anti-Gram+;antibact... at 46.15%
5     43aa, >Antimicrobial... at 55.81%
6     42aa, >anti-Gram+;Antibact... *
7     13aa, >antifungal... at 46.15%
8     22aa, >anti-Gram-;antibact... at 40.91%
9     248aa, >Antimicrobial... *
10    65aa, >Antimicrobial... at 41.54%
11    14aa, >antimicrobial... at 42.86%
12    39aa, >Antimicrobial... at 48.72%
13    33aa, >antimicrobial... at 45.45%
14    13aa, >antibacterial06606... at 46.15%
15    21aa, >antibacterial07397... at 42.86%
16    14aa, >antibacterial07404... at 42.86%
```

17   25aa, >antibacterial08085... at 35.00%
18   11aa, >antibacterial08289... at 63.64%
19   11aa, >antibacterial08299... at 45.45%
20   12aa, >antibacterial08374... at 41.67%
21   16aa, >antibacterial08538... at 43.75%
22   13aa, >antibacterial08903... at 46.15%
23   15aa, >antibacterial09278... at 46.67%
24   18aa, >antibacterial10111... at 44.44%
25   11aa, >antibacterial10559... at 54.55%
26   22aa, >antibacterial10720... at 50.00%
27   17aa, >antibacterial10909... at 41.18%
28   21aa, >antibacterial11129... at 42.86%
29   13aa, >antibacterial12066... at 46.15%
30   15aa, >antibacterial12116... at 53.33%
>Cluster 99
0   21aa, >antimicrobial... at 42.86%
1   22aa, >anti-Gram+;Gram-... at 40.91%
2   27aa, >antibacterial;antim... at 40.74%
3   25aa, >antibacterial... at 35.00%
4   245aa, >Antibacterial... *
5   245aa, >Antibacterial... at 77.96%
6   244aa, >Antibacterial... at 77.84%
7   246aa, >Antibacterial... at 76.14%
8   244aa, >Antibacterial... at 77.46%
9   246aa, >Antibacterial... *
10   246aa, >Antibacterial... at 84.08%
11   20aa, >antimicrobial... at 45.00%
12   16aa, >antimicrobial... at 43.75%
13   18aa, >anti-Gram-;antibact... at 44.44%
14   14aa, >antibacterial;antim... at 42.86%
15   19aa, >antibacterial08528... at 42.11%
16   21aa, >antibacterial08625... at 42.86%
17   14aa, >antibacterial10202... at 42.86%
18   13aa, >antibacterial12224... at 46.15%
19   13aa, >antibacterial12247... *
20   13aa, >antibacterial12320... at 53.85%
21   28aa, >antibacterial12776... at 42.86%
>Cluster 100
0   21aa, >antimicrobial... at 42.86%
1   18aa, >antimicrobial... at 44.44%
2   245aa, >Antibacterial... *
3   245aa, >Antibacterial... at 78.37%
4   17aa, >antimicrobial... at 41.18%
5   26aa, >antibacterial06645... at 42.31%
6   42aa, >antibacterial07506... at 40.48%
7   21aa, >antibacterial08917... at 42.86%
8   13aa, >antibacterial09810... at 46.15%
9   16aa, >antibacterial10765... at 43.75%
10   16aa, >antibacterial10875... at 50.00%
11   21aa, >antibacterial10949... at 42.86%
12   13aa, >antibacterial11372... *
13   20aa, >antibacterial12099... at 50.00%
14   11aa, >antibacterial12871... at 45.45%
15   18aa, >antibacterial12904... at 44.44%

```
16    12aa, >antibacterial13151... at 50.00%
17    20aa, >antibacterial13419... at 35.00%
>Cluster 101
0     20aa, >antimicrobial... at 35.00%
1     21aa, >anti-Gram+;antifung... at 47.62%
2     21aa, >antimicrobial... at 42.86%
3     20aa, >antimicrobial... at 45.00%
4     25aa, >Antibacterial... at 35.00%
5     244aa, >Antimicrobial... *
6     36aa, >anti-Gram+;Antimicr... at 44.44%
7     13aa, >Antibacterial... *
8     13aa, >Antibacterial... at 53.85%
9     40aa, >antibacterial... at 45.00%
10    22aa, >anti-Gram+;antibact... at 40.91%
11    16aa, >anti-Gram+;Antibact... at 50.00%
12    20aa, >antibacterial07084... at 35.00%
13    21aa, >antibacterial07395... at 42.86%
14    27aa, >antibacterial08345... at 40.74%
15    34aa, >antibacterial08560... at 41.18%
16    23aa, >antibacterial09905... at 47.83%
17    28aa, >antibacterial10042... at 50.00%
18    24aa, >antibacterial10046... at 41.67%
19    18aa, >antibacterial10229... *
20    26aa, >antibacterial10649... at 42.31%
21    18aa, >antibacterial10669... at 44.44%
22    13aa, >antibacterial12049... at 46.15%
23    13aa, >antibacterial12059... at 46.15%
24    29aa, >antibacterial12763... at 41.38%
25    22aa, >antibacterial12851... at 45.45%
>Cluster 102
0     17aa, >antimicrobial... at 41.18%
1     19aa, >antibacterial... at 42.11%
2     30aa, >antimicrobial... at 35.00%
3     241aa, >Antimicrobial... *
4     24aa, >Anti-Gram-;antibact... at 41.67%
5     30aa, >antimicrobial;antiv... at 35.00%
6     16aa, >antimicrobial... at 43.75%
7     18aa, >antibacterial06547... at 50.00%
8     14aa, >antibacterial06609... at 42.86%
9     18aa, >antibacterial06970... at 44.44%
10    18aa, >antibacterial06973... at 50.00%
11    18aa, >antibacterial06974... at 44.44%
12    14aa, >antibacterial08473... at 42.86%
13    29aa, >antibacterial09129... at 41.38%
14    14aa, >antibacterial09261... at 42.86%
15    18aa, >antibacterial09901... *
16    26aa, >antibacterial09967... at 42.31%
17    12aa, >antibacterial09976... at 41.67%
18    16aa, >antibacterial10273... at 43.75%
19    12aa, >antibacterial10488... at 41.67%
20    13aa, >antibacterial12200... at 46.15%
21    18aa, >antibacterial13008... at 55.56%
22    15aa, >antibacterial13357... at 35.00%
>Cluster 103
```

```
0     29aa, >Antibacterial;antim... at 41.38%
1     67aa, >Antifungal;Antimicr... at 44.78%
2     119aa, >Antibacterial... at 40.34%
3     119aa, >Antibacterial... at 42.02%
4     241aa, >Antibacterial... *
5     15aa, >antibacterial13205... at 35.00%
>Cluster 104
0     21aa, >antibacterial;Antif... at 42.86%
1     11aa, >antimicrobial... at 45.45%
2     235aa, >Antibacterial... *
3     19aa, >anti-Gram+;antibact... at 42.11%
4     12aa, >Antimicrobial... *
5     16aa, >antimicrobial... at 43.75%
6     13aa, >Antibacterial... at 46.15%
7     25aa, >antibacterial07277... at 44.00%
8     19aa, >antibacterial07333... at 42.11%
9     20aa, >antibacterial07358... at 45.00%
10    21aa, >antibacterial07392... at 42.86%
11    21aa, >antibacterial07394... at 42.86%
12    21aa, >antibacterial07599... at 42.86%
13    26aa, >antibacterial07805... at 42.31%
14    18aa, >antibacterial07809... *
15    20aa, >antibacterial08584... at 35.00%
16    21aa, >antibacterial09297... at 42.86%
17    14aa, >antibacterial09831... at 42.86%
18    21aa, >antibacterial10085... at 42.86%
19    21aa, >antibacterial10285... at 42.86%
20    11aa, >antibacterial10675... at 45.45%
21    18aa, >antibacterial10702... at 44.44%
22    29aa, >antibacterial11395... at 41.38%
23    27aa, >antibacterial11775... at 40.74%
24    15aa, >antibacterial12007... at 35.00%
25    13aa, >antibacterial12415... at 46.15%
26    15aa, >antibacterial12498... at 35.00%
27    15aa, >antibacterial12579... at 46.67%
28    15aa, >antibacterial12683... at 35.00%
>Cluster 105
0     13aa, >Anti-Gram-... *
1     26aa, >Antibacterial... at 42.31%
2     24aa, >antibacterial;antim... at 41.67%
3     11aa, >Antimicrobial... at 45.45%
4     233aa, >Antimicrobial... *
5     12aa, >antibacterial06669... at 41.67%
6     13aa, >antibacterial07002... at 46.15%
7     17aa, >antibacterial07554... at 41.18%
8     13aa, >antibacterial07568... at 46.15%
9     21aa, >antibacterial07600... at 42.86%
10    20aa, >antibacterial07755... at 55.00%
11    20aa, >antibacterial07848... at 55.00%
12    11aa, >antibacterial07875... at 54.55%
13    14aa, >antibacterial07912... at 42.86%
14    21aa, >antibacterial07945... at 42.86%
15    20aa, >antibacterial08054... at 35.00%
16    11aa, >antibacterial08521... at 45.45%
```

```
17    20aa, >antibacterial08849... at 35.00%
18    12aa, >antibacterial08992... at 41.67%
19    11aa, >antibacterial09189... at 45.45%
20    14aa, >antibacterial09490... at 42.86%
21    19aa, >antibacterial09492... at 42.11%
22    16aa, >antibacterial10122... at 43.75%
23    14aa, >antibacterial10123... at 50.00%
24    18aa, >antibacterial10125... at 44.44%
25    15aa, >antibacterial10149... at 35.00%
26    15aa, >antibacterial10173... at 35.00%
27    18aa, >antibacterial10330... *
28    11aa, >antibacterial10565... at 45.45%
29    18aa, >antibacterial10657... at 44.44%
30    18aa, >antibacterial10730... at 44.44%
31    18aa, >antibacterial11110... at 44.44%
32    15aa, >antibacterial11470... at 46.67%
33    37aa, >antibacterial11851... at 43.24%
34    13aa, >antibacterial12319... at 53.85%
35    12aa, >antibacterial12891... at 41.67%
36    16aa, >antibacterial12993... at 43.75%
37    21aa, >antibacterial13127... at 42.86%
38    12aa, >antibacterial13329... at 50.00%
>Cluster 106
0     231aa, >Antibacterial... *
1     27aa, >antibacterial;antim... at 40.74%
2     24aa, >anti-Gram+;antibact... at 45.83%
3     29aa, >antibacterial;antim... at 44.83%
4     13aa, >antibacterial07771... at 46.15%
5     14aa, >antibacterial10089... at 42.86%
6     30aa, >antibacterial10265... at 35.00%
7     36aa, >antibacterial12104... at 41.67%
>Cluster 107
0     67aa, >Antimicrobial... at 71.64%
1     67aa, >Antimicrobial... *
2     65aa, >Antimicrobial... at 53.85%
3     59aa, >Antimicrobial... at 62.71%
4     66aa, >Antimicrobial... at 57.27%
5     163aa, >Antimicrobial... at 45.40%
6     133aa, >Antimicrobial... at 61.65%
7     155aa, >Antimicrobial... at 67.74%
8     168aa, >Antimicrobial... at 54.76%
9     224aa, >Antimicrobial... at 47.32%
10    224aa, >Antimicrobial... at 64.29%
11    182aa, >Antimicrobial... at 67.03%
12    148aa, >Antimicrobial... at 79.73%
13    144aa, >Antimicrobial... at 77.78%
14    168aa, >Antimicrobial... at 66.07%
15    159aa, >Antimicrobial... at 68.55%
16    155aa, >Antimicrobial... at 74.19%
17    162aa, >Antimicrobial... at 77.78%
18    173aa, >Antimicrobial... at 63.58%
19    164aa, >Antimicrobial... at 64.63%
20    190aa, >Antimicrobial... at 70.53%
21    190aa, >Antimicrobial... at 71.58%
```

22 158aa, >Antimicrobial... at 67.72%
23 125aa, >Antimicrobial... *
24 158aa, >Antimicrobial... at 70.89%
25 159aa, >Antimicrobial... at 69.18%
26 174aa, >Antimicrobial... at 57.47%
27 185aa, >Antimicrobial... at 54.59%
28 159aa, >Antimicrobial... at 67.92%
29 229aa, >Antimicrobial... *
30 174aa, >Antimicrobial... *
31 135aa, >Antimicrobial... *
32 144aa, >Antimicrobial... at 79.86%
33 144aa, >Antimicrobial... at 79.86%
34 157aa, >Antimicrobial... at 64.33%
35 155aa, >Antimicrobial... at 66.45%
36 170aa, >Antimicrobial... at 52.94%
37 176aa, >Antimicrobial... at 52.27%
38 173aa, >Antimicrobial... at 53.76%
39 173aa, >Antimicrobial... at 53.18%
40 172aa, >Antimicrobial... at 52.33%
41 173aa, >Antimicrobial... at 53.18%
42 175aa, >Antimicrobial... at 43.43%
43 174aa, >Antimicrobial... at 45.40%
44 175aa, >Antimicrobial... at 45.71%
45 172aa, >Antimicrobial... at 47.67%
46 171aa, >Antimicrobial... at 54.97%
47 35aa, >anti-Gram+;antifung... at 35.00%
48 76aa, >Antimicrobial... at 51.32%
49 43aa, >antibacterial;antim... *
50 46aa, >Antibacterial... at 67.39%
51 99aa, >Antibacterial... at 44.44%
52 67aa, >Antimicrobial... at 71.64%
53 30aa, >Antimicrobial... at 35.00%
54 19aa, >antibacterial06498... at 42.11%
55 13aa, >antibacterial06612... at 46.15%
56 96aa, >antibacterial07311... *
57 22aa, >antibacterial07318... at 50.00%
58 23aa, >antibacterial07319... at 43.48%
59 43aa, >antibacterial07347... at 76.74%
60 60aa, >antibacterial07399... at 35.00%
61 30aa, >antibacterial07516... at 53.33%
62 30aa, >antibacterial07587... at 53.33%
63 17aa, >antibacterial07701... at 41.18%
64 14aa, >antibacterial07989... at 50.00%
65 17aa, >antibacterial08447... at 41.18%
66 32aa, >antibacterial08569... at 65.62%
67 32aa, >antibacterial08573... at 65.62%
68 32aa, >antibacterial08574... at 40.62%
69 11aa, >antibacterial09178... at 45.45%
70 26aa, >antibacterial10105... at 42.31%
71 13aa, >antibacterial10628... at 46.15%
72 12aa, >antibacterial10695... at 50.00%
73 99aa, >antibacterial11116... *
74 13aa, >antibacterial12065... at 46.15%
75 13aa, >antibacterial12156... at 46.15%

```
76    19aa, >antibacterial12629... at 42.11%
>Cluster 108
0     17aa, >anti-Gram+;Antibact... *
1     17aa, >antimicrobial... at 41.18%
2     24aa, >antimicrobial... at 41.67%
3     40aa, >Antimicrobial... at 42.50%
4     29aa, >antimicrobial... at 41.38%
5     227aa, >Antimicrobial... *
6     24aa, >antimicrobial... at 45.83%
7     24aa, >antimicrobial... at 41.67%
8     16aa, >antimicrobial... at 43.75%
9     16aa, >antimicrobial... at 43.75%
10    15aa, >antimicrobial... at 35.00%
11    24aa, >antibacterial08276... at 41.67%
12    18aa, >antibacterial08344... at 44.44%
13    16aa, >antibacterial08489... at 43.75%
14    17aa, >antibacterial09171... at 41.18%
15    25aa, >antibacterial09688... at 35.00%
16    22aa, >antibacterial10063... at 40.91%
17    19aa, >antibacterial10091... at 47.37%
18    26aa, >antibacterial10342... at 42.31%
19    25aa, >antibacterial11717... at 35.00%
20    13aa, >antibacterial11742... *
21    26aa, >antibacterial12168... at 42.31%
22    14aa, >antibacterial12392... at 42.86%
>Cluster 109
0     36aa, >antibacterial;antim... at 44.44%
1     25aa, >anti-Gram+;antibact... at 35.00%
2     26aa, >Antimicrobial... *
3     24aa, >antibacterial;antim... at 41.67%
4     226aa, >Antimicrobial... *
5     20aa, >antimicrobial... at 35.00%
6     15aa, >antibacterial... at 35.00%
7     32aa, >Antimicrobial... at 43.75%
8     24aa, >anti-Gram+;antibact... *
9     20aa, >antibacterial06981... at 45.00%
10    11aa, >antibacterial07373... at 45.45%
11    29aa, >antibacterial07457... at 41.38%
12    13aa, >antibacterial07803... at 53.85%
13    20aa, >antibacterial07940... at 45.00%
14    24aa, >antibacterial08132... at 41.67%
15    22aa, >antibacterial08275... at 40.91%
16    24aa, >antibacterial08281... at 41.67%
17    26aa, >antibacterial08600... at 42.31%
18    17aa, >antibacterial08623... at 41.18%
19    19aa, >antibacterial08670... at 47.37%
20    26aa, >antibacterial09056... at 50.00%
21    18aa, >antibacterial09619... at 44.44%
22    15aa, >antibacterial10152... at 35.00%
23    26aa, >antibacterial10336... at 46.15%
24    26aa, >antibacterial10337... at 42.31%
25    20aa, >antibacterial12788... at 35.00%
>Cluster 110
0     33aa, >antimicrobial... at 42.42%
```

```
1     33aa, >antimicrobial... at 45.45%
2     21aa, >antimicrobial... at 47.62%
3     225aa, >Antimicrobial... *
4     28aa, >antibacterial07688... *
5     28aa, >antibacterial07691... at 46.43%
6     28aa, >antibacterial07692... at 46.43%
7     35aa, >antibacterial10886... at 45.71%
8     18aa, >antibacterial12068... at 44.44%
9     20aa, >antibacterial12658... at 50.00%
>Cluster 111
0     32aa, >anti-Gram+;antibact... at 40.62%
1     48aa, >Antimicrobial... *
2     46aa, >Antimicrobial... at 71.74%
3     42aa, >Antimicrobial... at 80.95%
4     40aa, >Antimicrobial... *
5     48aa, >Antimicrobial... at 79.17%
6     223aa, >Antimicrobial... *
7     49aa, >Antimicrobial... at 79.59%
8     46aa, >Antimicrobial... at 49.57%
9     49aa, >Antibacterial... at 69.39%
10    49aa, >Antimicrobial... at 55.51%
11    49aa, >Antibacterial;Antim... at 57.35%
12    49aa, >Antimicrobial... at 63.47%
13    15aa, >antimicrobial... at 35.00%
14    24aa, >antifungal;antimicr... at 41.67%
15    52aa, >Antimicrobial... at 78.85%
16    43aa, >Antimicrobial... at 81.40%
17    52aa, >Antimicrobial... at 65.38%
18    52aa, >Antimicrobial... at 67.31%
19    68aa, >Antimicrobial... at 50.00%
20    18aa, >antibacterial;Antif... *
21    18aa, >antibacterial06548... at 50.00%
22    18aa, >antibacterial07807... at 44.44%
23    12aa, >antibacterial08376... at 41.67%
24    12aa, >antibacterial08430... at 50.00%
25    11aa, >antibacterial10321... at 45.45%
26    20aa, >antibacterial11996... at 35.00%
27    13aa, >antibacterial12044... at 46.15%
>Cluster 112
0     30aa, >antiviral... at 43.33%
1     21aa, >antimicrobial... at 52.38%
2     21aa, >anti-Gram+;Antibact... at 42.86%
3     222aa, >Antibacterial... *
4     219aa, >Antibacterial... at 68.95%
5     30aa, >Antifungal... at 35.00%
6     18aa, >antibacterial;antif... at 44.44%
7     18aa, >antibacterial;Antif... at 44.44%
8     20aa, >antibacterial;antim... at 35.00%
9     15aa, >antimicrobial... at 35.00%
10    16aa, >antimicrobial... at 43.75%
11    25aa, >antibacterial07276... *
12    18aa, >antibacterial07337... at 50.00%
13    18aa, >antibacterial07348... at 44.44%
14    11aa, >antibacterial08831... at 45.45%
```

15   19aa, >antibacterial09145... at 42.11%
16   30aa, >antibacterial11394... at 43.33%
17   25aa, >antibacterial11434... at 35.00%
18   18aa, >antibacterial11762... at 44.44%
19   23aa, >antibacterial11933... at 43.48%
20   11aa, >antibacterial13365... at 45.45%
>Cluster 113
0   19aa, >antimicrobial... at 42.11%
1   14aa, >anti-Gram+;antibact... *
2   30aa, >antibacterial;Antif... at 43.33%
3   29aa, >antimicrobial... at 41.38%
4   21aa, >antimicrobial... at 42.86%
5   189aa, >Antimicrobial... at 43.92%
6   208aa, >Antimicrobial... at 64.90%
7   202aa, >Antimicrobial... at 69.31%
8   159aa, >Antimicrobial... at 59.12%
9   207aa, >Antimicrobial... at 82.13%
10   198aa, >Antimicrobial... *
11   216aa, >Antimicrobial... at 82.41%
12   183aa, >Antimicrobial... at 83.06%
13   207aa, >Antimicrobial... at 77.29%
14   54aa, >Antimicrobial... at 42.59%
15   187aa, >Antimicrobial... at 73.80%
16   222aa, >Antimicrobial... *
17   40aa, >Antifungal;Gram-... at 42.50%
18   37aa, >anti-Gram+;antibact... at 45.95%
19   164aa, >Antimicrobial... at 70.12%
20   22aa, >antimicrobial... at 40.91%
21   48aa, >antibacterial07325... at 41.67%
22   16aa, >L02A001748... at 43.75%
23   25aa, >antibacterial08208... at 35.00%
24   14aa, >antibacterial09669... at 42.86%
25   16aa, >antibacterial09924... at 43.75%
26   58aa, >antibacterial10837... at 51.72%
27   18aa, >antibacterial10964... at 44.44%
28   20aa, >antibacterial11319... at 35.00%
29   25aa, >antibacterial11729... at 35.00%
30   16aa, >antibacterial13280... *
>Cluster 114
0   20aa, >antimicrobial... at 35.00%
1   222aa, >Antifungal... *
2   16aa, >antibacterial08457... at 43.75%
3   20aa, >antibacterial11432... at 50.00%
4   22aa, >antibacterial12575... at 45.45%
>Cluster 115
0   19aa, >anti-Gram+;Antibact... at 42.11%
1   30aa, >antimicrobial... at 35.00%
2   221aa, >Antibacterial... *
3   14aa, >antibacterial;antim... at 42.86%
4   12aa, >antibacterial09255... at 41.67%
5   12aa, >antibacterial09256... at 41.67%
6   13aa, >antibacterial10426... at 46.15%
7   12aa, >antibacterial12372... at 50.00%
8   18aa, >antibacterial12443... *

9   12aa, >antibacterial12570... at 41.67%
10   15aa, >antibacterial13193... at 35.00%
>Cluster 116
0   27aa, >antimicrobial... at 40.74%
1   20aa, >Antifungal;antimicr... at 35.00%
2   30aa, >anti-Gram+;antifung... at 35.00%
3   17aa, >anti-Gram+;antibact... at 41.18%
4   20aa, >Antibacterial... at 35.00%
5   220aa, >Antimicrobial... *
6   18aa, >Antimicrobial... at 44.44%
7   24aa, >antibacterial06590... at 45.83%
8   29aa, >antibacterial07680... at 41.38%
9   16aa, >antibacterial08359... at 50.00%
10   27aa, >antibacterial08914... at 40.74%
11   21aa, >antibacterial09317... at 47.62%
12   21aa, >antibacterial10327... at 42.86%
13   30aa, >antibacterial10481... at 35.00%
14   33aa, >antibacterial10569... at 45.45%
15   14aa, >antibacterial10794... at 50.00%
16   17aa, >antibacterial11733... at 52.94%
17   14aa, >antibacterial12086... at 50.00%
18   18aa, >antibacterial12160... at 44.44%
19   15aa, >antibacterial12662... at 35.00%
20   20aa, >antibacterial13311... at 35.00%
>Cluster 117
0   28aa, >Antimicrobial... at 42.86%
1   13aa, >antimicrobial... at 46.15%
2   27aa, >antimicrobial... at 44.44%
3   215aa, >Antibacterial... *
4   215aa, >Antibacterial... at 67.44%
5   28aa, >antibacterial06794... at 42.86%
6   19aa, >antibacterial08386... at 42.11%
7   16aa, >antibacterial08488... at 50.00%
8   20aa, >antibacterial09452... at 35.00%
9   19aa, >antibacterial09645... at 42.11%
10   20aa, >antibacterial10032... at 35.00%
11   19aa, >antibacterial12164... at 42.11%
12   26aa, >antibacterial12750... at 42.31%
>Cluster 118
0   16aa, >antimicrobial... at 56.25%
1   23aa, >anti-Gram+;antibact... at 43.48%
2   28aa, >Antibacterial;antim... at 46.43%
3   213aa, >Antimicrobial... *
4   25aa, >antibacterial;antim... at 35.00%
5   27aa, >antibacterial07652... at 40.74%
6   18aa, >antibacterial09227... at 44.44%
7   27aa, >antibacterial09945... at 40.74%
8   23aa, >antibacterial10051... at 43.48%
9   31aa, >antibacterial10608... at 41.94%
10   25aa, >antibacterial11545... at 35.00%
11   30aa, >antibacterial11562... at 35.00%
>Cluster 119
0   22aa, >Antibacterial... at 40.91%
1   11aa, >anti-Gram+;Antimicr... at 35.45%

```
2     18aa, >antimicrobial... at 34.44%
3     19aa, >antibacterial... at 42.11%
4     13aa, >Antimicrobial... at 36.15%
5     29aa, >Gram-... at 41.38%
6     32aa, >Antimicrobial... *
7     156aa, >Antimicrobial... at 50.00%
8     147aa, >Antimicrobial... at 40.14%
9     154aa, >Antimicrobial... at 62.99%
10    154aa, >Antimicrobial... *
11    213aa, >Antimicrobial... *
12    148aa, >Antimicrobial... at 41.22%
13    23aa, >antibacterial;antim... at 47.83%
14    24aa, >anti-Gram+;antibact... at 41.67%
15    33aa, >Antifungal;Gram-... at 42.42%
16    12aa, >antibacterial06614... at 41.67%
17    14aa, >antibacterial06618... at 42.86%
18    12aa, >antibacterial07022... at 50.00%
19    15aa, >antibacterial07025... at 35.00%
20    15aa, >antibacterial07718... at 35.00%
21    15aa, >antibacterial07721... at 35.00%
22    13aa, >antibacterial09597... at 46.15%
23    14aa, >antibacterial10577... at 50.00%
24    18aa, >antibacterial10963... at 50.00%
25    31aa, >antibacterial11351... at 41.94%
26    16aa, >antibacterial11634... at 43.75%
27    20aa, >antibacterial11830... at 35.00%
>Cluster 120
0     162aa, >Antimicrobial... *
1     13aa, >Antibacterial;antim... at 46.15%
2     13aa, >Antibacterial... at 46.15%
3     209aa, >Antimicrobial... at 77.51%
4     209aa, >Antimicrobial... at 83.25%
5     211aa, >Antimicrobial... *
6     208aa, >Antimicrobial... at 78.37%
7     20aa, >antibacterial08201... at 45.00%
8     17aa, >antibacterial09289... at 41.18%
9     20aa, >antibacterial09399... at 35.00%
10    11aa, >antibacterial10402... at 54.55%
11    20aa, >antibacterial13019... at 45.00%
>Cluster 121
0     30aa, >Antiviral... at 43.33%
1     30aa, >Antimicrobial... at 63.33%
2     30aa, >antimicrobial... at 56.67%
3     30aa, >antimicrobial... at 56.67%
4     32aa, >antimicrobial... at 40.62%
5     33aa, >antimicrobial... at 42.42%
6     30aa, >Antimicrobial... at 46.67%
7     30aa, >anti-HIV;antiviral... at 46.67%
8     30aa, >anti-HIV;antimicrob... at 43.33%
9     30aa, >antimicrobial... at 53.33%
10    31aa, >Antimicrobial;Antim... at 77.42%
11    29aa, >Antimicrobial;Antim... *
12    29aa, >Antimicrobial;Antim... at 68.97%
13    29aa, >antimicrobial... *
```

```
14    32aa, >antimicrobial... at 46.88%
15    30aa, >Antimicrobial... at 43.33%
16    30aa, >anti-Gram+;antibact... at 43.33%
17    31aa, >Antimicrobial... at 41.94%
18    29aa, >antimicrobial;Antim... at 68.97%
19    29aa, >antimicrobial... at 72.41%
20    153aa, >Antimicrobial... at 78.43%
21    155aa, >Antimicrobial... at 80.65%
22    107aa, >Antimicrobial... at 64.49%
23    210aa, >Antimicrobial... *
24    78aa, >Antimicrobial... at 44.87%
25    63aa, >Antimicrobial... at 44.44%
26    153aa, >Antimicrobial... at 46.41%
27    153aa, >Antimicrobial... at 45.75%
28    30aa, >Antimicrobial... at 46.67%
29    106aa, >Antimicrobial... at 64.15%
30    30aa, >antimicrobial;antiv... at 46.67%
31    29aa, >Antimicrobial... at 55.17%
32    28aa, >antibacterial07338... at 42.86%
33    30aa, >antibacterial07793... at 43.33%
34    12aa, >antibacterial07837... at 41.67%
35    32aa, >antibacterial10752... *
36    29aa, >antibacterial10754... at 65.52%
37    32aa, >antibacterial11410... at 43.75%
38    27aa, >antibacterial11800... at 40.74%
39    24aa, >antibacterial12622... at 41.67%
>Cluster 122
0     102aa, >Antimicrobial... at 41.18%
1     93aa, >anti-Gram+;Antibact... at 51.61%
2     40aa, >antimicrobial... at 35.00%
3     37aa, >antibacterial;antim... at 45.95%
4     208aa, >Antimicrobial... *
5     136aa, >Antimicrobial... at 45.59%
6     132aa, >Antimicrobial... at 40.91%
7     161aa, >Antimicrobial... *
8     32aa, >Antimicrobial... at 46.88%
9     17aa, >antimicrobial... at 41.18%
10    37aa, >Antimicrobial... at 40.54%
11    42aa, >antibacterial;antim... at 40.48%
12    37aa, >antibacterial;antif... at 40.54%
13    42aa, >antibacterial;antim... at 40.48%
14    31aa, >Antibacterial;Antif... at 41.94%
15    17aa, >antibacterial06601... *
16    15aa, >antibacterial06611... at 35.00%
17    26aa, >antibacterial07346... at 42.31%
18    23aa, >antibacterial08368... at 43.48%
19    23aa, >antibacterial08375... at 43.48%
20    22aa, >antibacterial10665... at 50.00%
21    20aa, >antibacterial10831... at 35.00%
>Cluster 123
0     13aa, >antimicrobial... at 46.15%
1     208aa, >Antimicrobial... *
2     13aa, >antimicrobial... at 46.15%
3     26aa, >antibacterial07934... at 46.15%
```

```
4     15aa, >antibacterial08037... at 35.00%
5     26aa, >antibacterial09005... at 53.85%
6     21aa, >antibacterial10052... at 47.62%
7     22aa, >antibacterial10108... at 45.45%
8     30aa, >antibacterial10611... at 35.00%
9     26aa, >antibacterial10616... at 42.31%
>Cluster 124
0     207aa, >Antimicrobial... *
1     24aa, >anti-Gram-;Antibact... at 45.83%
2     20aa, >anti-Gram+;antibact... at 35.00%
3     25aa, >antibacterial;antim... at 44.00%
4     25aa, >antimicrobial... at 35.00%
5     25aa, >Antibacterial;antim... at 44.00%
6     22aa, >antimicrobial... at 40.91%
7     21aa, >anti-Gram+;antimicr... at 42.86%
8     18aa, >Antimicrobial... at 44.44%
9     38aa, >antibacterial07378... at 42.11%
10    20aa, >antibacterial09089... *
11    12aa, >antibacterial09240... at 50.00%
12    20aa, >antibacterial09454... at 35.00%
13    16aa, >antibacterial09496... at 43.75%
14    19aa, >antibacterial10382... at 42.11%
15    20aa, >antibacterial10525... at 35.00%
16    31aa, >antibacterial11712... at 41.94%
17    13aa, >antibacterial11741... at 46.15%
18    17aa, >antibacterial11989... at 41.18%
>Cluster 125
0     207aa, >Antimicrobial... *
1     18aa, >antibacterial06556... at 44.44%
2     14aa, >antibacterial07742... at 42.86%
3     16aa, >antibacterial08468... at 50.00%
4     15aa, >antibacterial13058... at 35.00%
5     13aa, >antibacterial13073... at 46.15%
6     19aa, >antibacterial13137... at 42.11%
>Cluster 126
0     27aa, >anti-Gram-;Antibact... at 40.74%
1     31aa, >anti-Gram-;antibact... at 45.16%
2     17aa, >antimicrobial... at 41.18%
3     20aa, >Antibacterial... at 45.00%
4     15aa, >antimicrobial... at 46.67%
5     180aa, >Antimicrobial... at 44.44%
6     205aa, >Antimicrobial... *
7     175aa, >Antimicrobial... at 48.00%
8     16aa, >antimicrobial... *
9     30aa, >antibacterial06461... at 43.33%
10    29aa, >antibacterial06570... at 41.38%
11    22aa, >antibacterial07034... at 40.91%
12    21aa, >antibacterial08274... at 42.86%
13    11aa, >antibacterial08287... at 45.45%
14    11aa, >antibacterial08426... at 45.45%
15    12aa, >antibacterial08428... at 50.00%
16    17aa, >antibacterial09156... at 41.18%
17    25aa, >antibacterial10373... at 44.00%
18    33aa, >antibacterial11121... at 42.42%
```

19   21aa, >antibacterial11282... at 42.86%
20   15aa, >antibacterial11297... at 35.00%
21   27aa, >antibacterial11657... at 44.44%
22   15aa, >antibacterial11789... at 46.67%
23   18aa, >antibacterial13091... at 50.00%
>Cluster 127
0   13aa, >Antifungal;antibact... *
1   29aa, >antimicrobial... at 41.38%
2   29aa, >antimicrobial... at 41.38%
3   25aa, >Antibacterial;antim... at 35.00%
4   24aa, >antimicrobial... at 41.67%
5   17aa, >Antimicrobial... at 41.18%
6   18aa, >antimicrobial... at 44.44%
7   203aa, >Antimicrobial... *
8   17aa, >antibacterial08392... at 41.18%
9   18aa, >antibacterial08851... at 44.44%
10   15aa, >antibacterial09734... at 35.00%
11   13aa, >antibacterial10460... at 46.15%
12   18aa, >antibacterial12539... at 44.44%
13   18aa, >antibacterial12540... at 50.00%
14   15aa, >antibacterial12639... at 35.00%
15   20aa, >antibacterial13152... at 35.00%
16   20aa, >antibacterial13167... at 35.00%
>Cluster 128
0   32aa, >anti-Gram+;Antibact... at 43.75%
1   42aa, >anti-Gram+;antibact... *
2   44aa, >antimicrobial... at 60.45%
3   25aa, >Antibacterial;antim... at 35.00%
4   202aa, >Antimicrobial... *
5   26aa, >anti-Gram+;antibact... at 46.15%
6   19aa, >anti-Gram-;antibact... at 42.11%
7   15aa, >antibacterial07046... at 35.00%
8   26aa, >antibacterial07431... at 42.31%
9   16aa, >antibacterial07521... at 63.75%
10   13aa, >antibacterial07905... at 43.85%
11   24aa, >antibacterial08143... at 41.67%
12   35aa, >antibacterial09472... at 42.86%
13   24aa, >antibacterial10506... at 41.67%
14   24aa, >antibacterial11519... at 41.67%
15   13aa, >antibacterial12053... at 46.15%
>Cluster 129
0   35aa, >anti-Gram+;antibact... at 35.00%
1   27aa, >Antimicrobial... at 40.74%
2   27aa, >Antimicrobial... at 44.44%
3   30aa, >antibacterial;antim... *
4   202aa, >Antimicrobial... *
5   19aa, >antimicrobial... at 42.11%
6   19aa, >antimicrobial... at 42.11%
7   19aa, >antimicrobial... at 42.11%
8   23aa, >antibacterial06961... at 43.48%
9   12aa, >antibacterial06988... at 41.67%
10   13aa, >antibacterial06990... at 46.15%
11   12aa, >antibacterial07016... at 41.67%
12   20aa, >antibacterial07633... at 45.00%

```
13    26aa, >antimicrobial... *
14    26aa, >antimicrobial... at 50.00%
15    25aa, >antibacterial07839... at 35.00%
16    23aa, >antibacterial07895... at 43.48%
17    17aa, >antibacterial07907... at 41.18%
18    15aa, >antibacterial08683... at 35.00%
19    15aa, >antibacterial08702... at 35.00%
20    13aa, >antibacterial09583... at 46.15%
21    13aa, >antibacterial09585... at 46.15%
22    26aa, >antibacterial09760... at 42.31%
23    14aa, >antibacterial10199... at 42.86%
24    21aa, >antibacterial11283... at 42.86%
25    14aa, >antibacterial11475... at 42.86%
26    40aa, >antibacterial12110... at 35.00%
27    26aa, >antibacterial12417... at 42.31%
28    26aa, >antibacterial12435... at 42.31%
29    29aa, >antibacterial12732... at 41.38%
30    18aa, >antibacterial13098... at 44.44%
>Cluster 130
0     191aa, >Antimicrobial... at 40.84%
1     193aa, >Antimicrobial... *
2     195aa, >Antimicrobial... at 86.67%
3     195aa, >Antimicrobial... *
4     174aa, >Antimicrobial... at 79.66%
5     202aa, >Antimicrobial... *
6     181aa, >Antimicrobial... at 77.29%
7     198aa, >Antimicrobial... at 69.39%
8     45aa, >antibacterial06635... *
9     25aa, >antibacterial07693... at 35.00%
10    25aa, >antibacterial07702... at 35.00%
11    32aa, >antibacterial08462... at 40.62%
12    15aa, >antibacterial08627... at 35.00%
13    30aa, >antibacterial08720... at 35.00%
14    18aa, >antibacterial09705... at 34.44%
>Cluster 131
0     201aa, >Antimicrobial... *
>Cluster 132
0     16aa, >antimicrobial... at 43.75%
1     200aa, >Antifungal... *
>Cluster 133
0     27aa, >Antifungal;antimicr... at 40.74%
1     199aa, >Antimicrobial... *
2     16aa, >antibacterial07850... at 43.75%
3     13aa, >antibacterial08379... at 46.15%
4     23aa, >antibacterial09941... at 43.48%
5     20aa, >antibacterial10031... at 35.00%
6     14aa, >antibacterial10385... at 42.86%
7     20aa, >antibacterial10498... at 35.00%
8     19aa, >antibacterial12018... at 42.11%
9     14aa, >antibacterial12393... at 42.86%
10    18aa, >antibacterial12613... at 50.00%
>Cluster 134
0     30aa, >antimicrobial... at 43.33%
1     198aa, >Antibacterial... *
```

```
2     15aa, >antibacterial10171... at 35.00%
>Cluster 135
0     23aa, >antimicrobial... at 43.48%
1     21aa, >anti-Gram+;antibact... at 52.38%
2     198aa, >Antimicrobial... *
3     14aa, >anti-Gram+;antibact... at 42.86%
4     12aa, >antibacterial10408... at 50.00%
5     35aa, >antibacterial11368... at 35.00%
6     23aa, >antibacterial12535... at 43.48%
7     15aa, >antibacterial13268... at 35.00%
>Cluster 136
0     25aa, >anti-Gram+;antibact... at 44.00%
1     183aa, >anti-Gram+;Gram-... at 58.47%
2     194aa, >Antibacterial... at 72.68%
3     195aa, >Antimicrobial... *
4     185aa, >Antibacterial... at 52.97%
5     18aa, >antibacterial08057... at 44.44%
6     13aa, >antibacterial10064... at 46.15%
7     30aa, >antibacterial10734... at 35.00%
8     13aa, >antibacterial11744... at 46.15%
9     26aa, >antibacterial12441... at 42.31%
10    20aa, >antibacterial12826... at 35.00%
>Cluster 137
0     19aa, >antifungal;antimicr... *
1     25aa, >antimicrobial... at 35.00%
2     193aa, >Antimicrobial... *
3     24aa, >antibacterial;antif... at 41.67%
4     14aa, >Antibacterial;Antif... at 42.86%
5     22aa, >antibacterial06702... at 45.45%
6     18aa, >antibacterial08093... at 44.44%
7     24aa, >antibacterial08125... at 41.67%
8     24aa, >antibacterial08128... at 41.67%
9     24aa, >antibacterial08141... at 50.00%
10    13aa, >antibacterial08381... at 53.85%
11    23aa, >antibacterial09058... at 43.48%
12    26aa, >antibacterial09794... *
13    26aa, >antibacterial09795... at 42.31%
14    27aa, >antibacterial09797... at 42.31%
15    27aa, >antibacterial09798... at 42.31%
16    28aa, >antibacterial09799... at 42.31%
17    18aa, >antibacterial10126... at 44.44%
18    19aa, >antibacterial10635... at 47.37%
19    30aa, >antibacterial11448... at 35.00%
20    22aa, >antibacterial12590... at 40.91%
21    18aa, >antibacterial12623... at 44.44%
22    13aa, >antibacterial13290... at 46.15%
>Cluster 138
0     30aa, >antibacterial;antif... *
1     20aa, >Antibacterial... at 45.00%
2     115aa, >Antimicrobial... at 45.22%
3     145aa, >Antimicrobial... at 55.86%
4     139aa, >Antimicrobial... at 45.32%
5     193aa, >Antimicrobial... *
6     148aa, >anti-Gram+;Gram-... *
```

7   12aa, >antibacterial07959... at 41.67%
8   12aa, >antibacterial08283... at 50.00%
9   12aa, >antibacterial08288... at 50.00%
10   16aa, >antibacterial08324... at 50.00%
11   17aa, >antibacterial08328... at 47.06%
12   18aa, >antibacterial08338... at 34.44%
13   18aa, >antibacterial08703... at 34.44%
14   11aa, >antibacterial09815... at 54.55%
15   11aa, >antibacterial09816... at 45.45%
16   14aa, >antibacterial10775... at 42.86%
17   33aa, >antibacterial11777... at 42.42%
18   13aa, >antibacterial12297... at 46.15%
>Cluster 139
0   191aa, >Antibacterial;Antif... *
1   35aa, >antibacterial;antif... at 35.00%
2   27aa, >Antimicrobial... at 44.44%
3   23aa, >antibacterial08951... at 43.48%
4   20aa, >antibacterial09459... at 35.00%
5   24aa, >antibacterial10107... at 45.83%
6   13aa, >antibacterial10459... at 46.15%
7   13aa, >antibacterial10698... at 46.15%
8   25aa, >antibacterial11196... at 35.00%
9   35aa, >antibacterial11471... at 35.00%
10   34aa, >antibacterial12190... at 44.12%
>Cluster 140
0   29aa, >antimicrobial... at 41.38%
1   41aa, >Antimicrobial... at 43.90%
2   29aa, >Antimicrobial... at 41.38%
3   19aa, >antibacterial;antif... at 42.11%
4   24aa, >anti-Gram+;anti-HIV... at 41.67%
5   50aa, >antiviral... at 35.00%
6   125aa, >Antifungal... *
7   191aa, >Antifungal... *
8   23aa, >antibacterial06586... at 43.48%
9   12aa, >antibacterial07020... at 41.67%
10   29aa, >antibacterial07500... at 44.83%
11   14aa, >antibacterial07822... at 42.86%
12   11aa, >antibacterial10540... at 54.55%
13   18aa, >antibacterial12021... at 50.00%
14   20aa, >antibacterial13124... at 35.00%
15   29aa, >antibacterial13220... at 41.38%
>Cluster 141
0   95aa, >Antimicrobial... at 60.00%
1   188aa, >Antibacterial... *
2   188aa, >Antibacterial... at 84.04%
3   20aa, >antimicrobial... at 45.00%
4   82aa, >Antimicrobial... at 59.76%
5   19aa, >antibacterial06558... at 47.37%
6   13aa, >antibacterial10643... at 46.15%
7   19aa, >antibacterial10684... at 47.37%
8   20aa, >antibacterial13174... at 35.00%
>Cluster 142
0   108aa, >Antimicrobial... at 49.07%
1   38aa, >Antimicrobial... *

```
2     37aa, >Antimicrobial... at 40.54%
3     14aa, >anti-Gram-;antibact... at 42.86%
4     18aa, >antibacterial;Antif... at 50.00%
5     52aa, >Antimicrobial... at 51.92%
6     39aa, >Antimicrobial... at 51.28%
7     22aa, >anti-Gram+;antibact... at 40.91%
8     44aa, >Antimicrobial... at 40.91%
9     186aa, >Antimicrobial... *
10    22aa, >antibacterial;antim... at 40.91%
11    60aa, >Antimicrobial... at 48.33%
12    45aa, >Antimicrobial... at 35.00%
13    18aa, >antibacterial;antif... at 50.00%
14    18aa, >antibacterial;Antif... at 44.44%
15    45aa, >Antimicrobial... at 35.00%
16    15aa, >antibacterial06603... at 35.00%
17    18aa, >antibacterial07813... at 44.44%
18    20aa, >antibacterial07944... at 45.00%
19    16aa, >antibacterial08352... at 43.75%
20    18aa, >antibacterial09107... *
21    18aa, >antibacterial09707... at 44.44%
22    19aa, >antibacterial10050... at 44.44%
23    21aa, >antibacterial10287... at 42.86%
24    14aa, >antibacterial13185... at 50.00%
>Cluster 143
0     23aa, >antimicrobial... at 43.48%
1     186aa, >Antimicrobial... *
2     185aa, >Antimicrobial... at 85.41%
3     18aa, >antibacterial09898... at 44.44%
4     20aa, >antibacterial12711... at 45.00%
>Cluster 144
0     24aa, >anti-Gram+;Antibact... at 41.67%
1     185aa, >Antimicrobial... *
2     19aa, >antibacterial06682... at 42.11%
3     22aa, >antibacterial08441... at 45.45%
4     19aa, >antibacterial08453... at 52.63%
>Cluster 145
0     25aa, >antimicrobial... at 35.00%
1     29aa, >anti-Gram+;antibact... at 41.38%
2     182aa, >Antimicrobial... *
3     22aa, >antibacterial06533... at 40.91%
4     20aa, >antibacterial11784... at 35.00%
>Cluster 146
0     31aa, >antimicrobial;antiv... at 70.97%
1     31aa, >antimicrobial... at 74.19%
2     178aa, >Antimicrobial... *
3     31aa, >antimicrobial... at 61.29%
4     29aa, >Antibacterial;antim... at 44.83%
5     33aa, >antimicrobial... at 45.45%
6     30aa, >Antimicrobial;Antim... at 46.67%
7     21aa, >antimicrobial... at 42.86%
8     30aa, >antimicrobial... at 46.67%
9     32aa, >antimicrobial... at 56.25%
10    31aa, >Antibacterial;Antim... *
11    18aa, >antibacterial;antim... at 44.44%
```

```
12    30aa, >antimicrobial... at 63.33%
13    30aa, >Antimicrobial;Antim... *
14    29aa, >Antimicrobial... at 82.76%
15    31aa, >anti-HIV;antiviral... at 64.52%
16    26aa, >antibacterial... at 50.00%
17    144aa, >Antimicrobial... at 78.19%
18    179aa, >Antimicrobial... *
19    116aa, >Antimicrobial... at 50.86%
20    115aa, >Antimicrobial... at 55.65%
21    113aa, >Antimicrobial... at 51.33%
22    113aa, >Antimicrobial... at 52.21%
23    114aa, >Antimicrobial... at 58.77%
24    113aa, >Antimicrobial... at 47.79%
25    28aa, >antimicrobial;antiv... at 32.86%
26    31aa, >Antimicrobial;Antim... at 37.74%
27    16aa, >antibacterial;antim... at 36.25%
28    31aa, >antibacterial06488... at 43.87%
29    31aa, >antibacterial06490... at 58.06%
30    30aa, >antibacterial06941... at 56.67%
31    31aa, >L02A001810... at 77.42%
32    30aa, >antibacterial06950... at 80.00%
33    20aa, >antibacterial07035... at 45.00%
34    11aa, >antibacterial08224... at 45.45%
35    30aa, >antibacterial11413... at 70.00%
36    31aa, >antibacterial11439... at 51.61%
>Cluster 147
0     179aa, >Antimicrobial... *
1     37aa, >antimicrobial... at 48.65%
2     41aa, >Gram-... at 41.46%
3     16aa, >antibacterial08414... at 43.75%
4     20aa, >antibacterial09118... at 35.00%
5     17aa, >antibacterial09495... at 41.18%
6     32aa, >antibacterial10499... at 43.75%
7     18aa, >antibacterial13210... at 50.00%
>Cluster 148
0     14aa, >anti-Gram+;Gram-... at 42.86%
1     178aa, >Antimicrobial... *
2     37aa, >Antimicrobial... at 40.54%
3     18aa, >antibacterial06508... at 44.44%
4     13aa, >antibacterial07576... at 46.15%
5     36aa, >antibacterial11365... at 41.67%
6     28aa, >antibacterial12746... at 42.86%
>Cluster 149
0     177aa, >Antibacterial... *
1     17aa, >antibacterial08242... at 41.18%
2     17aa, >antibacterial08246... at 41.18%
3     17aa, >antibacterial08249... at 47.06%
4     13aa, >antibacterial10465... at 46.15%
>Cluster 150
0     169aa, >Antimicrobial... at 63.91%
1     25aa, >anti-Gram+;Gram-... at 44.00%
2     160aa, >Antimicrobial... at 60.00%
3     164aa, >Antimicrobial... at 50.00%
4     162aa, >Antimicrobial... at 56.17%
```

```
5     36aa, >antibacterial;antim... at 44.44%
6     175aa, >Antimicrobial... *
7     111aa, >Antimicrobial... at 65.77%
8     131aa, >Antimicrobial... at 44.27%
9     20aa, >antibacterial07763... at 35.00%
10    22aa, >antibacterial08717... at 40.91%
11    26aa, >antibacterial11378... at 46.15%
>Cluster 151
0     130aa, >Antibacterial... at 53.08%
1     155aa, >Anti-Gram-... at 54.19%
2     155aa, >Anti-Gram-... at 52.26%
3     153aa, >Anti-Gram-... at 54.25%
4     21aa, >anti-Gram+;Gram-... at 47.62%
5     161aa, >Anti-Gram-... at 51.55%
6     156aa, >Antimicrobial... *
7     18aa, >antibacterial07888... at 44.44%
8     19aa, >antibacterial10389... at 42.11%
9     18aa, >antibacterial13064... at 44.44%
10    163aa, >antibacterial13430... at 60.74%
11    174aa, >antibacterial13431... *
>Cluster 152
0     166aa, >Antibacterial... at 72.29%
1     30aa, >antimicrobial... at 35.00%
2     37aa, >Antibacterial;Antim... at 40.54%
3     169aa, >Antibacterial... at 77.51%
4     172aa, >Antibacterial... *
5     13aa, >antimicrobial... at 53.85%
6     11aa, >antibacterial07625... at 63.64%
7     19aa, >antibacterial08585... at 42.11%
8     35aa, >antibacterial09331... at 35.00%
9     11aa, >antibacterial10660... at 45.45%
>Cluster 153
0     25aa, >antibacterial;antif... at 35.00%
1     30aa, >anti-Gram+;antibact... at 43.33%
2     171aa, >Antimicrobial... *
3     169aa, >Antimicrobial... at 67.46%
4     171aa, >Antimicrobial... at 69.59%
5     18aa, >antibacterial08593... at 44.44%
>Cluster 154
0     23aa, >antibacterial;Antif... at 43.48%
1     20aa, >antimicrobial... at 45.00%
2     26aa, >antibacterial;Antif... at 42.31%
3     171aa, >Anti-Gram-... *
4     18aa, >Antimicrobial... at 50.00%
5     18aa, >Antimicrobial... at 44.44%
6     14aa, >Anti-Gram-... at 42.86%
7     15aa, >Antibacterial... *
8     20aa, >antibacterial07596... at 45.00%
9     11aa, >antibacterial07859... at 45.45%
10    25aa, >antibacterial08008... at 44.00%
11    25aa, >antibacterial08010... at 44.00%
12    16aa, >antibacterial08323... at 43.75%
13    23aa, >antibacterial09222... at 47.83%
14    11aa, >antibacterial09349... at 45.45%
```

```
15    13aa, >antibacterial09843... at 46.15%
16    25aa, >antibacterial09877... at 35.00%
17    14aa, >antibacterial10207... at 42.86%
18    15aa, >antibacterial10723... at 35.00%
19    23aa, >antibacterial11606... at 43.48%
20    13aa, >antibacterial11811... *
21    22aa, >antibacterial11823... at 40.91%
22    18aa, >antibacterial11906... at 44.44%
23    11aa, >antibacterial12384... at 45.45%
24    17aa, >antibacterial12689... at 41.18%
>Cluster 155
0     169aa, >Antibacterial... *
1     17aa, >antibacterial08023... at 41.18%
2     17aa, >antibacterial08676... at 41.18%
3     18aa, >antibacterial11530... at 44.44%
4     15aa, >antibacterial12125... at 46.67%
>Cluster 156
0     30aa, >antimicrobial... at 35.00%
1     30aa, >Antibacterial;Antif... at 50.00%
2     13aa, >antimicrobial... *
3     122aa, >Antibacterial... at 59.02%
4     18aa, >Antibacterial;Antif... at 50.00%
5     117aa, >Antimicrobial... at 59.83%
6     25aa, >anti-Gram+;Antibact... at 35.00%
7     33aa, >antibacterial;antif... at 42.42%
8     164aa, >Antibacterial... at 69.51%
9     169aa, >Antibacterial... *
10    117aa, >Antibacterial... at 64.10%
11    117aa, >Antimicrobial... at 69.23%
12    22aa, >antibacterial08622... at 40.91%
13    21aa, >antibacterial08624... at 42.86%
14    19aa, >antibacterial09143... at 47.37%
15    19aa, >antibacterial09146... at 47.37%
16    25aa, >antibacterial10845... at 44.00%
17    19aa, >antibacterial11955... at 42.11%
>Cluster 157
0     40aa, >antibacterial;antim... at 35.00%
1     24aa, >antimicrobial... at 41.67%
2     33aa, >antimicrobial... at 42.42%
3     35aa, >Antimicrobial... at 35.00%
4     23aa, >anti-Gram+;antibact... at 43.48%
5     24aa, >anti-Gram+;antibact... at 45.83%
6     130aa, >Antimicrobial... at 41.54%
7     116aa, >Antimicrobial... *
8     116aa, >Antimicrobial... at 57.76%
9     115aa, >Antimicrobial... at 46.09%
10    123aa, >Antimicrobial... at 58.54%
11    130aa, >Antimicrobial... at 58.46%
12    123aa, >Antimicrobial... at 60.98%
13    166aa, >Antimicrobial... *
14    122aa, >Antimicrobial... at 51.64%
15    113aa, >Antimicrobial... at 41.59%
16    119aa, >Antimicrobial... at 45.38%
17    119aa, >Antimicrobial... at 47.06%
```

```
18    61aa, >Antimicrobial... *
19    25aa, >antibacterial06678... at 35.00%
20    79aa, >antibacterial07269... at 44.30%
21    18aa, >antibacterial07808... at 44.44%
22    13aa, >antibacterial10550... at 46.15%
23    17aa, >antibacterial11521... at 41.18%
24    27aa, >antibacterial13031... at 40.74%
>Cluster 158
0     147aa, >Antimicrobial... at 46.26%
1     164aa, >Antibacterial... *
2     144aa, >Antibacterial... at 62.07%
3     145aa, >Antimicrobial... at 44.14%
4     39aa, >Antimicrobial... at 43.59%
5     18aa, >antimicrobial... at 44.44%
6     40aa, >Antimicrobial... at 35.00%
7     13aa, >antibacterial06604... at 46.15%
8     11aa, >antibacterial08286... at 45.45%
9     18aa, >antibacterial08337... at 50.00%
10    17aa, >antibacterial08350... at 41.18%
11    16aa, >antibacterial08360... *
12    18aa, >antibacterial09325... at 44.44%
13    13aa, >antibacterial10469... at 46.15%
>Cluster 159
0     13aa, >Antibacterial... at 46.15%
1     164aa, >Antimicrobial... *
2     18aa, >antimicrobial... at 44.44%
3     33aa, >Gram-... at 45.45%
4     25aa, >antibacterial07416... at 35.00%
5     13aa, >antibacterial07507... at 46.15%
6     22aa, >antibacterial07789... at 40.91%
7     16aa, >antibacterial08322... at 50.00%
8     17aa, >antibacterial08332... at 41.18%
9     18aa, >antibacterial08339... at 44.44%
10    18aa, >antibacterial08626... *
11    23aa, >antibacterial12380... at 43.48%
12    13aa, >antibacterial12980... at 46.15%
>Cluster 160
0     14aa, >antimicrobial... at 42.86%
1     24aa, >anti-Gram+;Antibact... at 41.67%
2     163aa, >Antibacterial... *
3     24aa, >Antibacterial... at 41.67%
4     24aa, >antimicrobial... at 41.67%
5     24aa, >anti-Gram+;antibact... at 41.67%
6     18aa, >antimicrobial... at 44.44%
7     18aa, >antibacterial09854... at 44.44%
8     27aa, >antibacterial10446... at 40.74%
9     24aa, >antibacterial13246... at 41.67%
10    24aa, >antibacterial13248... at 41.67%
11    20aa, >antibacterial13399... at 35.00%
>Cluster 161
0     30aa, >antimicrobial... at 35.00%
1     23aa, >antimicrobial... at 47.83%
2     22aa, >antimicrobial... at 40.91%
3     160aa, >Antimicrobial... *
```

4   24aa, >antibacterial07741... at 41.67%
>Cluster 162
0   21aa, >anti-Gram+;antibact... at 52.38%
1   40aa, >anti-Gram+;Antibact... at 42.50%
2   25aa, >antibacterial;antif... at 44.00%
3   159aa, >Antimicrobial... *
4   74aa, >Antimicrobial... at 44.59%
5   19aa, >antibacterial;antim... at 42.11%
6   41aa, >Antimicrobial... at 56.10%
7   73aa, >Antimicrobial... at 41.10%
8   40aa, >Antimicrobial... *
9   91aa, >Antimicrobial... at 56.04%
10   69aa, >Antimicrobial... at 43.48%
11   41aa, >Antimicrobial... at 48.78%
12   36aa, >Antimicrobial... at 47.22%
13   39aa, >Anti-Gram-... at 41.03%
14   41aa, >Antimicrobial... at 41.46%
15   45aa, >Antimicrobial... at 42.22%
16   16aa, >anti-Gram-;antibact... at 50.00%
17   21aa, >antibacterial11064... at 42.86%
>Cluster 163
0   14aa, >antibacterial... at 42.86%
1   24aa, >antimicrobial... at 45.83%
2   24aa, >antimicrobial... at 41.67%
3   21aa, >antimicrobial... at 47.62%
4   131aa, >Antibacterial... at 43.51%
5   130aa, >Antibacterial... at 46.92%
6   130aa, >Antibacterial... at 45.38%
7   130aa, >Antibacterial... at 43.85%
8   130aa, >Antibacterial... at 45.38%
9   130aa, >Antibacterial... at 46.15%
10   129aa, >Antibacterial... at 44.96%
11   125aa, >Antibacterial... at 45.60%
12   130aa, >Antibacterial... at 46.15%
13   130aa, >Antibacterial... at 45.38%
14   130aa, >Antibacterial... at 50.00%
15   129aa, >Antibacterial... *
16   129aa, >Antibacterial... at 45.74%
17   130aa, >Antibacterial... at 47.69%
18   120aa, >Antibacterial... at 43.33%
19   129aa, >Antibacterial... at 40.31%
20   120aa, >Antibacterial... at 43.33%
21   129aa, >Antibacterial... at 44.96%
22   121aa, >antifungal... at 42.15%
23   119aa, >Antibacterial... at 43.70%
24   130aa, >Antibacterial... at 41.54%
25   130aa, >Antibacterial... at 46.15%
26   129aa, >Antibacterial... at 41.09%
27   129aa, >Antibacterial... at 41.09%
28   130aa, >Antibacterial... at 47.69%
29   130aa, >anti-Gram+;Antibact... at 46.92%
30   128aa, >Antibacterial... at 47.66%
31   129aa, >Antibacterial... at 45.74%
32   130aa, >Antibacterial... at 47.69%

```
33    129aa, >Antibacterial... *
34    129aa, >Antibacterial... at 44.96%
35    130aa, >Antibacterial... at 43.85%
36    130aa, >Antibacterial... at 43.08%
37    130aa, >Antibacterial;Antif... at 51.54%
38    129aa, >Antibacterial... at 46.51%
39    129aa, >Antimicrobial... at 45.74%
40    129aa, >Antibacterial... at 44.19%
41    129aa, >Antibacterial... at 46.51%
42    129aa, >Antibacterial... at 47.29%
43    130aa, >Antibacterial;Antif... at 50.00%
44    129aa, >Antibacterial... at 44.96%
45    158aa, >Antibacterial... *
46    128aa, >Antibacterial... at 43.75%
47    49aa, >Antibacterial... at 40.82%
48    26aa, >antibacterial07784... at 42.31%
49    18aa, >antibacterial09551... at 44.44%
50    24aa, >antibacterial09723... at 41.67%
>Cluster 164
0     35aa, >Antimicrobial... at 42.86%
1     147aa, >Antimicrobial... at 40.82%
2     30aa, >antimicrobial... at 35.00%
3     23aa, >Antifungal... at 43.48%
4     158aa, >Antimicrobial... *
5     21aa, >antimicrobial... at 47.62%
6     19aa, >anti-Gram-;antibact... at 47.37%
7     19aa, >antibacterial06563... at 42.11%
8     22aa, >antibacterial10706... at 40.91%
>Cluster 165
0     16aa, >Antibacterial... at 56.25%
1     157aa, >Antibacterial... *
2     26aa, >antibacterial11599... at 42.31%
>Cluster 166
0     23aa, >Antimicrobial... at 47.83%
1     157aa, >Antimicrobial... *
2     23aa, >antibacterial11359... at 43.48%
3     19aa, >antibacterial13130... at 52.63%
4     19aa, >antibacterial13134... at 52.63%
5     19aa, >antibacterial13136... at 47.37%
6     19aa, >antibacterial13139... at 42.11%
7     19aa, >antibacterial13140... at 52.63%
8     19aa, >antibacterial13141... at 42.11%
9     19aa, >antibacterial13142... at 52.63%
10    12aa, >antibacterial13403... at 41.67%
>Cluster 167
0     18aa, >antibacterial;Antif... at 44.44%
1     21aa, >antimicrobial... at 42.86%
2     157aa, >Antimicrobial... *
3     141aa, >Antimicrobial... at 40.43%
4     73aa, >Antimicrobial... at 41.10%
5     35aa, >antibacterial08401... at 35.00%
6     28aa, >antibacterial10726... at 42.86%
7     18aa, >antibacterial10788... at 44.44%
8     20aa, >antibacterial11995... at 35.00%
```

>Cluster 168
0	120aa, >anti-Gram-... *
1	109aa, >anti-Gram-... at 67.89%
2	133aa, >anti-Gram-;Antibact... at 62.41%
3	13aa, >antimicrobial... at 46.15%
4	21aa, >antimicrobial... at 42.86%
5	21aa, >antimicrobial... at 42.86%
6	155aa, >antiviral... *
7	49aa, >antibacterial07322... at 46.94%
8	20aa, >antibacterial07814... at 45.00%
9	13aa, >antibacterial09035... at 53.85%
10	20aa, >antibacterial09124... at 35.00%
11	20aa, >antibacterial09127... at 35.00%
12	14aa, >antibacterial09465... at 50.00%
13	19aa, >antibacterial09925... at 52.63%
14	20aa, >antibacterial10622... at 35.00%
15	18aa, >antibacterial10939... at 44.44%
16	18aa, >antibacterial10945... at 44.44%
17	19aa, >antibacterial11115... *
18	20aa, >antibacterial11120... at 44.44%
>Cluster 169
0	23aa, >Antibacterial;Antim... at 43.48%
1	152aa, >Antimicrobial... *
>Cluster 170
0	152aa, >Gram-... *
1	13aa, >antibacterial10430... at 46.15%
2	12aa, >antibacterial11489... at 41.67%
>Cluster 171
0	42aa, >anti-Gram+;antibact... at 40.48%
1	151aa, >Antimicrobial... *
2	28aa, >anti-Gram+;antibact... at 42.86%
3	17aa, >antibacterial08243... at 41.18%
4	18aa, >antibacterial08405... at 50.00%
5	21aa, >antibacterial08716... at 42.86%
6	18aa, >antibacterial12827... at 50.00%
>Cluster 172
0	150aa, >Antibacterial... *
1	150aa, >Antimicrobial... at 67.33%
2	148aa, >Antimicrobial... at 56.08%
3	148aa, >Antimicrobial... at 52.03%
4	149aa, >Antimicrobial... at 73.83%
5	145aa, >Antimicrobial... at 66.21%
6	150aa, >Antimicrobial... at 78.00%
7	148aa, >Antimicrobial... at 51.35%
8	20aa, >antibacterial07951... at 35.00%
9	28aa, >antibacterial12755... at 42.86%
>Cluster 173
0	149aa, >Antibacterial... at 67.11%
1	150aa, >Antibacterial... *
2	149aa, >anti-Gram+;Antibact... at 70.47%
3	28aa, >antimicrobial... at 42.86%
4	138aa, >Antibacterial... at 72.03%
5	137aa, >Antibacterial... at 70.80%
6	30aa, >L02A001809... at 35.00%

```
7     30aa, >antibacterial13164... at 33.33%
>Cluster 174
0     24aa, >antimicrobial... at 45.83%
1     31aa, >antimicrobial... at 45.16%
2     78aa, >Antimicrobial... at 69.23%
3     32aa, >antimicrobial... at 40.62%
4     31aa, >antimicrobial... at 41.94%
5     18aa, >antibacterial... at 44.44%
6     148aa, >Antimicrobial... *
7     17aa, >anti-Gram+;antibact... at 41.18%
8     79aa, >Antimicrobial... at 64.56%
9     145aa, >Antimicrobial... at 57.24%
10    145aa, >Antimicrobial... at 53.10%
11    145aa, >Antimicrobial... at 59.31%
12    143aa, >Antimicrobial... at 53.85%
13    78aa, >antibacterial06983... at 62.82%
14    19aa, >antibacterial08657... at 42.11%
15    13aa, >antibacterial11469... at 53.85%
16    18aa, >antibacterial11898... at 44.44%
17    19aa, >antibacterial12015... at 47.37%
18    19aa, >antibacterial12017... *
19    21aa, >antibacterial12171... at 42.86%
>Cluster 175
0     31aa, >antimicrobial... at 45.16%
1     30aa, >Antiviral... at 46.67%
2     27aa, >antimicrobial... at 51.85%
3     45aa, >Antimicrobial... at 42.22%
4     46aa, >antimicrobial... *
5     41aa, >antibacterial;Antim... at 56.10%
6     46aa, >Antibacterial... at 58.70%
7     46aa, >Antibacterial... at 58.70%
8     46aa, >Antimicrobial... at 54.35%
9     46aa, >Antimicrobial;Antim... at 56.52%
10    46aa, >Antimicrobial;Antim... at 52.17%
11    47aa, >Antibacterial;Antim... at 53.19%
12    44aa, >anti-Gram+;antibact... at 61.36%
13    45aa, >anti-Gram+;antibact... at 53.33%
14    135aa, >Antimicrobial... at 49.63%
15    148aa, >Antimicrobial... *
16    112aa, >Antimicrobial... *
17    135aa, >Antimicrobial... at 53.33%
18    135aa, >Antimicrobial... at 48.15%
19    135aa, >Antimicrobial... at 48.89%
20    13aa, >antimicrobial... at 46.15%
21    46aa, >antibacterial07054... at 54.35%
22    20aa, >antibacterial07941... at 35.00%
23    24aa, >antibacterial08497... at 41.67%
24    16aa, >antibacterial08984... at 43.75%
25    45aa, >antibacterial09164... at 35.00%
26    25aa, >antibacterial11105... at 35.00%
27    15aa, >antibacterial11598... at 46.67%
>Cluster 176
0     26aa, >Antimicrobial... at 50.00%
1     35aa, >Antibacterial... at 35.00%
```

```
2     27aa, >anti-Gram+;Antimicr... at 41.85%
3     27aa, >antibacterial;Antif... at 38.15%
4     27aa, >anti-Gram-;antibact... at 51.85%
5     26aa, >antibacterial;Antif... at 32.31%
6     114aa, >Antimicrobial... at 79.86%
7     129aa, >Antimicrobial... at 81.94%
8     134aa, >Antimicrobial... at 87.50%
9     136aa, >Antimicrobial... *
10    141aa, >Antimicrobial... at 75.52%
11    144aa, >Antimicrobial... at 79.17%
12    144aa, >Antimicrobial... at 79.86%
13    148aa, >Antimicrobial... *
14    78aa, >Antimicrobial... *
15    145aa, >Antimicrobial... at 80.34%
16    144aa, >Antimicrobial... at 83.33%
17    144aa, >Antimicrobial... at 81.94%
18    134aa, >Antimicrobial... at 82.84%
19    148aa, >Antimicrobial... at 82.43%
20    71aa, >Antimicrobial... at 80.28%
21    144aa, >Antimicrobial... *
22    145aa, >Antimicrobial... at 82.76%
23    144aa, >Antimicrobial... at 81.25%
24    145aa, >Antimicrobial... at 80.28%
25    144aa, >Antimicrobial... at 79.86%
26    142aa, >Antimicrobial... at 80.28%
27    148aa, >Antimicrobial... *
28    143aa, >Antimicrobial... at 83.22%
29    144aa, >Antimicrobial... at 79.17%
30    139aa, >Antimicrobial... at 81.29%
31    145aa, >Antimicrobial... at 84.83%
32    113aa, >Antimicrobial... at 71.68%
33    142aa, >Antimicrobial... at 80.99%
34    144aa, >Antimicrobial... at 82.64%
35    144aa, >Antimicrobial... at 77.08%
36    144aa, >Antimicrobial... at 72.22%
37    22aa, >antibacterial08061... at 30.91%
38    27aa, >antibacterial10398... *
39    36aa, >antibacterial10770... at 44.44%
40    18aa, >antibacterial11178... at 40.00%
41    27aa, >antibacterial11801... *
42    27aa, >antibacterial11810... at 52.96%
43    26aa, >antibacterial12861... at 50.00%
>Cluster 177
0     17aa, >antimicrobial... at 41.18%
1     101aa, >Antimicrobial... *
2     89aa, >Antimicrobial... at 52.81%
3     95aa, >Antimicrobial... at 44.21%
4     146aa, >Antimicrobial... *
5     113aa, >Antimicrobial... at 40.71%
6     87aa, >Antimicrobial... at 59.77%
7     91aa, >Antimicrobial... at 69.23%
8     106aa, >Antimicrobial... at 45.28%
9     14aa, >antibacterial10197... at 42.86%
10    27aa, >antibacterial12179... at 40.74%
```

11   31aa, >antibacterial13378... at 41.94%
>Cluster 178
0   32aa, >antimicrobial... at 40.62%
1   32aa, >antimicrobial... at 43.75%
2   33aa, >anti-Gram-;antibact... at 42.42%
3   32aa, >antimicrobial... at 40.62%
4   41aa, >antibacterial;antim... at 48.78%
5   23aa, >antibacterial;antim... at 52.17%
6   23aa, >antimicrobial.... at 52.17%
7   20aa, >antibacterial;antim... at 45.00%
8   30aa, >antibacterial;antim... *
9   25aa, >antibacterial;Antif... at 35.00%
10   20aa, >anti-Gram+;antibact... at 35.00%
11   49aa, >antibacterial;antim... at 53.06%
12   42aa, >antibacterial;antim... at 52.38%
13   29aa, >antimicrobial... at 44.83%
14   146aa, >Antibacterial... *
15   40aa, >antibacterial;antim... at 45.00%
16   18aa, >antibacterial07045... at 44.44%
17   15aa, >antibacterial08194... at 35.00%
18   22aa, >antibacterial09043... at 59.09%
19   22aa, >antibacterial09045... at 59.09%
20   66aa, >antibacterial09159... at 42.42%
21   33aa, >antibacterial09867... at 45.45%
22   27aa, >antibacterial11333... at 40.74%
>Cluster 179
0   26aa, >antibacterial... at 42.31%
1   145aa, >Antimicrobial... *
2   12aa, >antimicrobial... at 50.00%
3   38aa, >antibacterial07622... at 44.74%
4   17aa, >antibacterial07697... at 41.18%
5   19aa, >antibacterial07760... at 47.37%
6   24aa, >antibacterial08136... at 45.83%
7   13aa, >antibacterial09218... at 46.15%
8   16aa, >antibacterial09847... at 43.75%
9   14aa, >antibacterial10865... at 42.86%
10   19aa, >antibacterial11310... at 42.11%
>Cluster 180
0   143aa, >Antifungal... *
1   44aa, >antibacterial06623... at 40.91%
2   22aa, >antibacterial06649... at 40.91%
3   13aa, >antibacterial11284... at 46.15%
4   33aa, >antibacterial11767... at 42.42%
5   15aa, >antibacterial12098... at 46.67%
6   25aa, >antibacterial12691... at 44.00%
>Cluster 181
0   13aa, >antimicrobial... at 46.15%
1   24aa, >anti-Gram+;Antibact... at 41.67%
2   24aa, >anti-Gram+;antibact... at 41.67%
3   28aa, >antimicrobial... at 42.86%
4   143aa, >antifungal... *
5   22aa, >antibacterial08583... at 45.45%
6   13aa, >antibacterial09759... at 46.15%
>Cluster 182

```
0     142aa, >Antibacterial... *
1     19aa, >antibacterial07408... at 42.11%
2     16aa, >antibacterial07745... at 43.75%
3     17aa, >antibacterial07750... at 41.18%
4     19aa, >antibacterial09491... at 42.11%
>Cluster 183
0     24aa, >anti-Gram+;antibact... at 41.67%
1     103aa, >Antimicrobial... at 41.75%
2     29aa, >antimicrobial... at 44.83%
3     130aa, >Antimicrobial... at 46.92%
4     46aa, >antibacterial;Antim... at 56.52%
5     45aa, >Antimicrobial... at 53.33%
6     46aa, >Antibacterial;Antif... *
7     112aa, >Antimicrobial... at 47.32%
8     137aa, >Antimicrobial... at 68.61%
9     137aa, >Antimicrobial... at 66.42%
10    138aa, >Antimicrobial... at 59.42%
11    135aa, >Antimicrobial... *
12    135aa, >Antimicrobial... at 53.33%
13    135aa, >Antimicrobial... at 51.85%
14    137aa, >Antimicrobial... at 67.15%
15    142aa, >Antimicrobial... *
16    137aa, >Antimicrobial... at 71.53%
17    136aa, >Antimicrobial... at 68.38%
18    114aa, >Antimicrobial... at 41.23%
19    134aa, >Antimicrobial... at 44.78%
20    134aa, >Antimicrobial... at 40.30%
21    133aa, >Antimicrobial... at 45.86%
22    140aa, >Antimicrobial... at 43.57%
23    140aa, >Antimicrobial... at 46.43%
24    121aa, >Antimicrobial... at 42.15%
25    133aa, >Antimicrobial... at 48.87%
26    137aa, >Antimicrobial... at 54.01%
27    137aa, >Antimicrobial... at 54.74%
28    137aa, >Antimicrobial... at 47.45%
29    137aa, >Antimicrobial... at 49.64%
30    136aa, >Antimicrobial... at 47.79%
31    137aa, >Antimicrobial... at 50.36%
32    136aa, >Antimicrobial... at 52.21%
33    136aa, >Antimicrobial... at 48.53%
34    139aa, >Antimicrobial... at 53.24%
35    139aa, >Antimicrobial... at 53.96%
36    114aa, >Antimicrobial... at 45.61%
37    126aa, >Antimicrobial... at 56.35%
38    11aa, >antibacterial07872... at 54.55%
39    11aa, >antibacterial07873... at 45.45%
40    18aa, >antibacterial11466... at 50.00%
>Cluster 184
0     19aa, >Antimicrobial... at 42.11%
1     142aa, >Antibacterial... *
2     19aa, >antibacterial;antim... at 42.11%
3     20aa, >antibacterial10938... at 45.00%
4     24aa, >antibacterial11677... at 41.67%
>Cluster 185
```

```
0     142aa, >Antimicrobial... *
>Cluster 186
0     141aa, >Antibacterial... *
1     18aa, >Antibacterial;Antif... at 44.44%
2     20aa, >antibacterial;antim... at 50.00%
3     14aa, >antibacterial07332... at 42.86%
4     17aa, >antibacterial08244... at 41.18%
>Cluster 187
0     28aa, >antimicrobial... at 46.43%
1     31aa, >antimicrobial... at 61.29%
2     93aa, >Antimicrobial... at 47.31%
3     31aa, >antimicrobial... at 45.16%
4     31aa, >antimicrobial... *
5     28aa, >antibacterial;antim... at 46.43%
6     33aa, >antimicrobial;antiv... at 51.52%
7     30aa, >antimicrobial... at 50.00%
8     22aa, >Antibacterial... at 40.91%
9     31aa, >antimicrobial... at 74.19%
10    31aa, >antimicrobial... at 64.52%
11    31aa, >antimicrobial... *
12    31aa, >antimicrobial... at 64.52%
13    29aa, >antimicrobial... at 62.07%
14    28aa, >Anti-Gram-;antibact... at 64.29%
15    30aa, >antimicrobial;antiv... at 66.67%
16    25aa, >anti-Gram-... at 44.00%
17    31aa, >antimicrobial;antiv... at 61.29%
18    30aa, >antimicrobial... at 63.33%
19    30aa, >antimicrobial... at 70.00%
20    31aa, >Antimicrobial... at 61.29%
21    128aa, >Antimicrobial... at 80.47%
22    141aa, >Antimicrobial... *
23    64aa, >Antimicrobial... at 48.44%
24    31aa, >antibacterial06553... at 61.29%
25    30aa, >antibacterial06949... at 66.67%
26    26aa, >antibacterial07059... at 42.31%
27    32aa, >L02A001915... at 56.25%
28    31aa, >antibacterial07447... at 61.29%
29    18aa, >antibacterial08993... at 50.00%
30    21aa, >antibacterial09946... at 42.86%
31    15aa, >antibacterial10440... at 35.00%
32    13aa, >antibacterial11793... at 53.85%
>Cluster 188
0     22aa, >anti-Gram+;antibact... at 40.91%
1     21aa, >Anti-Gram-;antibact... at 42.86%
2     27aa, >antibacterial;antim... *
3     32aa, >antibacterial;antim... at 40.62%
4     23aa, >anti-Gram+;Antibact... at 43.48%
5     24aa, >Antibacterial;Antif... at 41.67%
6     24aa, >anti-Gram+;Antibact... at 41.67%
7     36aa, >antibacterial;antim... at 69.44%
8     46aa, >antibacterial;antim... at 67.39%
9     46aa, >antibacterial;antim... at 71.74%
10    42aa, >anti-Gram-;antibact... at 66.67%
11    40aa, >antibacterial;antim... at 67.50%
```

```
12    141aa, >Antibacterial... *
13    24aa, >antibacterial08279... at 41.67%
14    26aa, >antibacterial08347... at 50.00%
15    42aa, >antibacterial10652... at 54.76%
16    18aa, >antibacterial11184... at 44.44%
>Cluster 189
0     26aa, >antibiotic;Antimicr... at 46.15%
1     140aa, >Gram-... *
2     13aa, >antibacterial07802... at 46.15%
3     16aa, >antibacterial12109... at 43.75%
4     21aa, >antibacterial12354... at 42.86%
5     21aa, >antibacterial12409... at 42.86%
>Cluster 190
0     24aa, >anti-Gram+;antibact... at 45.83%
1     32aa, >anti-Gram+;Antibact... at 40.62%
2     23aa, >antimicrobial... at 43.48%
3     25aa, >antibacterial;antim... at 44.00%
4     140aa, >anti-Gram+;antifung... *
5     39aa, >Antimicrobial... at 41.03%
6     23aa, >antibacterial06647... at 33.48%
7     26aa, >antibacterial07785... at 46.15%
8     22aa, >antibacterial08758... at 30.91%
9     16aa, >antibacterial11850... at 43.75%
10    27aa, >antibacterial12135... at 40.74%
>Cluster 191
0     19aa, >antimicrobial... at 37.37%
1     87aa, >Antimicrobial... at 45.98%
2     96aa, >Antimicrobial... at 45.83%
3     81aa, >Antimicrobial... at 55.56%
4     41aa, >Antimicrobial... at 41.46%
5     118aa, >Antimicrobial... at 77.12%
6     137aa, >Antimicrobial... at 71.97%
7     137aa, >Antimicrobial... *
8     127aa, >Antimicrobial... at 39.48%
9     140aa, >Antimicrobial... *
10    139aa, >Antimicrobial... at 74.17%
11    11aa, >antibacterial08921... at 32.45%
12    18aa, >antibacterial09711... at 31.44%
13    29aa, >antibacterial12775... at 34.83%
>Cluster 192
0     30aa, >Antifungal;Gram-... at 43.33%
1     138aa, >Antimicrobial... *
2     30aa, >antibacterial10294... at 43.33%
3     45aa, >antibacterial10298... at 44.44%
4     15aa, >antibacterial11666... at 36.67%
>Cluster 193
0     137aa, >anti-Gram+;antifung... *
1     27aa, >Antifungal;Antimicr... at 44.44%
2     24aa, >antibacterial08134... at 45.83%
3     19aa, >antibacterial08203... at 32.11%
4     19aa, >antibacterial08205... at 32.11%
5     18aa, >antibacterial10374... at 44.44%
6     25aa, >antibacterial11427... at 35.00%
>Cluster 194
```

```
0     136aa, >anti-Gram-... *
1     27aa, >antibacterial06675... at 40.74%
2     26aa, >antibacterial06677... at 42.31%
3     25aa, >antibacterial07778... at 44.00%
4     25aa, >antibacterial08846... at 44.00%
5     13aa, >antibacterial08895... *
6     11aa, >antibacterial09332... at 45.45%
7     27aa, >antibacterial09336... at 40.74%
8     11aa, >antibacterial09819... at 45.45%
9     24aa, >antibacterial12014... at 41.67%
>Cluster 195
0     34aa, >Antimicrobial... at 41.18%
1     44aa, >Antimicrobial... at 40.91%
2     39aa, >Antimicrobial... at 43.59%
3     50aa, >Antimicrobial... at 44.00%
4     28aa, >antimicrobial... at 42.86%
5     33aa, >anti-Gram+;antibact... at 42.42%
6     43aa, >Antibacterial;Antim... at 58.14%
7     37aa, >anti-Gram+;Antibact... at 36.76%
8     43aa, >Antimicrobial... at 83.72%
9     43aa, >Antimicrobial... *
10    36aa, >antimicrobial... at 44.44%
11    35aa, >Antimicrobial... at 42.86%
12    66aa, >Antimicrobial... at 45.45%
13    61aa, >Antimicrobial... at 59.02%
14    61aa, >Antimicrobial... at 52.46%
15    63aa, >Antimicrobial... at 42.86%
16    136aa, >Antimicrobial... *
17    71aa, >Antimicrobial... at 49.30%
18    62aa, >Antimicrobial... at 40.32%
19    62aa, >Antimicrobial... at 41.94%
20    58aa, >Antimicrobial... at 74.14%
21    55aa, >Antimicrobial... at 41.82%
22    71aa, >Antimicrobial... at 43.66%
23    71aa, >Antimicrobial... at 43.66%
24    62aa, >Antimicrobial... at 59.68%
25    67aa, >Antimicrobial... at 43.28%
26    73aa, >Antimicrobial... at 45.21%
27    70aa, >Antimicrobial... at 45.71%
28    62aa, >Antimicrobial... at 58.06%
29    62aa, >Antimicrobial... at 40.32%
30    59aa, >Antimicrobial... at 40.68%
31    103aa, >Antimicrobial... *
32    61aa, >Antimicrobial... *
33    43aa, >anti-Gram+;antibact... at 48.84%
34    39aa, >antibacterial;antim... at 53.85%
35    49aa, >Antimicrobial... at 44.90%
36    41aa, >antibacterial07063... at 65.85%
37    11aa, >antibacterial07871... at 45.45%
38    44aa, >antibacterial08510... at 52.27%
39    44aa, >antibacterial08869... at 68.18%
40    47aa, >antibacterial09763... at 53.19%
41    21aa, >antibacterial10236... at 47.62%
42    29aa, >antibacterial11383... at 48.28%
```

43   20aa, >antibacterial11974... at 35.00%
44   36aa, >antibacterial12451... at 47.22%
45   33aa, >antibacterial12620... at 45.45%
46   20aa, >antibacterial13104... at 35.00%
>Cluster 196
0   29aa, >anti-Gram+;antibact... at 41.38%
1   22aa, >anti-Gram+;antibact... at 40.91%
2   30aa, >anti-Gram+;antibact... at 50.00%
3   34aa, >antibacterial;antim... *
4   84aa, >Antimicrobial... at 45.24%
5   136aa, >Antimicrobial... *
6   38aa, >anti-Gram+;antibact... at 42.11%
7   29aa, >antibacterial06491... at 41.38%
8   28aa, >antibacterial06632... at 60.71%
9   21aa, >antibacterial06671... at 42.86%
10   32aa, >antibacterial06687... at 46.88%
11   17aa, >antibacterial07465... at 41.18%
12   24aa, >antibacterial08129... at 41.67%
13   30aa, >antibacterial08207... at 63.33%
14   26aa, >antibacterial09761... at 42.31%
15   22aa, >antibacterial10621... at 40.91%
16   18aa, >antibacterial10632... at 44.44%
17   18aa, >antibacterial10658... at 50.00%
18   37aa, >antibacterial11612... at 43.24%
19   29aa, >antibacterial12170... at 41.38%
20   21aa, >antibacterial12371... at 42.86%
21   23aa, >antibacterial13238... at 43.48%
>Cluster 197
0   28aa, >anti-Gram+;antibact... at 42.86%
1   56aa, >Antimicrobial... at 51.79%
2   135aa, >Antimicrobial... *
3   56aa, >Antimicrobial... at 55.36%
4   52aa, >Antimicrobial... at 42.31%
5   47aa, >Antimicrobial... at 42.55%
6   56aa, >Antimicrobial... *
7   34aa, >anti-Gram+;antibact... at 41.18%
8   55aa, >Antimicrobial... at 50.91%
9   55aa, >Antimicrobial... at 47.27%
10   46aa, >antibacterial;antim... at 43.48%
11   46aa, >Antimicrobial... at 47.83%
12   51aa, >Antimicrobial... at 45.10%
13   53aa, >Antimicrobial... at 52.83%
14   56aa, >Antimicrobial... at 50.00%
15   56aa, >Antimicrobial... at 48.21%
16   57aa, >Antimicrobial... at 43.86%
17   57aa, >Antimicrobial... at 43.86%
18   54aa, >Antimicrobial... at 46.30%
19   57aa, >Antimicrobial... at 42.11%
>Cluster 198
0   24aa, >antibacterial;Antif... at 45.83%
1   19aa, >antimicrobial... at 42.11%
2   134aa, >Antimicrobial... at 54.48%
3   134aa, >Antimicrobial... at 51.49%
4   135aa, >Antimicrobial... *

```
5     133aa, >Antimicrobial... at 47.37%
6     135aa, >Antimicrobial... at 57.78%
7     125aa, >Antimicrobial... at 52.80%
8     134aa, >Antimicrobial... at 52.99%
9     134aa, >Antimicrobial... at 41.79%
10    125aa, >Antimicrobial... at 49.60%
11    48aa, >antibacterial;antim... at 43.75%
12    118aa, >Antimicrobial... at 45.76%
13    127aa, >Antimicrobial... at 52.76%
14    13aa, >antibacterial08313... at 46.15%
15    19aa, >antibacterial09149... at 47.37%
16    20aa, >antibacterial12600... at 35.00%
>Cluster 199
0     21aa, >antimicrobial... at 32.86%
1     21aa, >antimicrobial... at 32.86%
2     32aa, >antimicrobial... at 48.75%
3     31aa, >Antimicrobial... at 48.06%
4     28aa, >antimicrobial... at 36.43%
5     28aa, >antimicrobial... at 40.00%
6     24aa, >anti-Gram+;Antibact... at 30.00%
7     29aa, >Antimicrobial;Antim... at 34.83%
8     29aa, >antimicrobial... at 34.83%
9     31aa, >antibacterial;antim... at 35.16%
10    31aa, >Antimicrobial... *
11    30aa, >Antimicrobial;Antim... at 76.67%
12    31aa, >antimicrobial... at 83.87%
13    31aa, >antimicrobial... at 67.74%
14    29aa, >antimicrobial... at 55.17%
15    31aa, >Antimicrobial;Antim... at 67.74%
16    28aa, >antimicrobial... at 50.00%
17    32aa, >antimicrobial... at 40.62%
18    17aa, >anti-Gram+;antibact... at 41.18%
19    130aa, >Antimicrobial... at 70.77%
20    78aa, >Antimicrobial... at 41.03%
21    77aa, >Antimicrobial... at 42.86%
22    77aa, >Antimicrobial... at 44.16%
23    124aa, >Antimicrobial... at 63.71%
24    123aa, >Antimicrobial... at 65.04%
25    122aa, >Antimicrobial... *
26    132aa, >Antimicrobial... at 53.03%
27    135aa, >Antimicrobial... *
28    32aa, >Antimicrobial... at 53.12%
29    29aa, >antimicrobial... at 41.38%
30    30aa, >antibacterial06940... at 73.33%
31    18aa, >antibacterial11523... at 44.44%
>Cluster 200
0     135aa, >Antifungal... *
1     16aa, >antimicrobial... at 50.00%
2     21aa, >antibacterial08621... at 47.62%
>Cluster 201
0     127aa, >anti-Gram+;Antibact... at 44.88%
1     128aa, >Antibacterial;Antif... at 42.97%
2     134aa, >antiviral... *
3     131aa, >antiviral... at 47.33%
```

4   127aa, >anti-Gram+;Gram-... at 44.88%
5   127aa, >anti-Gram+;Gram-... at 47.24%
6   27aa, >antibacterial09888... at 59.26%
7   30aa, >antibacterial09890... at 53.33%
8   133aa, >antibacterial13429... at 65.41%
>Cluster 202
0   133aa, >Antibacterial... *
1   17aa, >antibacterial09504... at 41.18%
2   40aa, >antibacterial11480... at 35.00%
>Cluster 203
0   20aa, >antimicrobial... at 35.00%
1   25aa, >antimicrobial... at 48.00%
2   21aa, >Antimicrobial... at 47.62%
3   133aa, >Antimicrobial... *
4   16aa, >antibacterial07955... at 43.75%
5   20aa, >antibacterial12795... at 35.00%
>Cluster 204
0   132aa, >Antimicrobial... *
1   16aa, >antimicrobial... at 43.75%
2   40aa, >Antibacterial;Antif... at 47.50%
3   11aa, >antibacterial11199... at 45.45%
>Cluster 205
0   30aa, >anti-Gram+;Antibact... at 46.67%
1   21aa, >antimicrobial... at 42.86%
2   46aa, >Antibacterial;Antif... at 45.65%
3   126aa, >Antimicrobial... at 62.70%
4   57aa, >Antimicrobial... *
5   93aa, >Antimicrobial... at 46.24%
6   93aa, >Antimicrobial... at 44.09%
7   93aa, >Antimicrobial... at 47.31%
8   97aa, >Antimicrobial... at 40.21%
9   94aa, >Antimicrobial... at 43.62%
10   94aa, >Antimicrobial... at 40.43%
11   89aa, >Antimicrobial... at 40.45%
12   89aa, >Antimicrobial... at 43.82%
13   132aa, >Antimicrobial... *
14   34aa, >Antimicrobial... at 52.94%
15   92aa, >antibacterial06840... at 42.39%
16   93aa, >antibacterial06841... at 47.31%
17   93aa, >antibacterial06843... at 40.86%
18   93aa, >antibacterial06881... at 43.01%
19   87aa, >antibacterial06885... at 43.68%
20   95aa, >antibacterial06887... at 41.05%
21   92aa, >antibacterial07055... at 43.48%
22   93aa, >antibacterial07110... at 44.09%
23   93aa, >antibacterial07112... at 44.09%
24   93aa, >antibacterial07113... at 41.94%
25   91aa, >antibacterial07212... at 76.92%
26   96aa, >antibacterial07213... *
27   14aa, >antibacterial08314... at 42.86%
28   15aa, >antibacterial08532... at 35.00%
29   22aa, >antibacterial10248... at 45.45%
30   30aa, >antibacterial10736... at 46.67%
31   33aa, >antibacterial10757... at 45.45%

```
32    30aa, >antibacterial10759... at 35.00%
33    92aa, >antibacterial11032... at 47.83%
34    25aa, >antibacterial11436... at 44.00%
>Cluster 206
0     131aa, >antibacterial... *
1     15aa, >Gram-... at 35.00%
2     21aa, >antibacterial07459... at 42.86%
>Cluster 207
0     23aa, >anti-Gram+;antifung... at 43.48%
1     26aa, >anti-Gram+;antibact... at 42.31%
2     23aa, >antibacterial;antim... at 43.48%
3     29aa, >antimicrobial... at 41.38%
4     131aa, >Antimicrobial... *
5     31aa, >antibacterial10252... at 45.16%
6     28aa, >antibacterial10262... at 42.86%
>Cluster 208
0     18aa, >antibacterial... at 55.56%
1     131aa, >Antibacterial;Antif... *
2     131aa, >anti-Gram+;antifung... at 44.27%
3     18aa, >antibacterial06545... at 44.44%
4     53aa, >antibacterial07323... at 41.51%
5     18aa, >antibacterial07362... at 50.00%
6     25aa, >antibacterial07415... at 35.00%
7     18aa, >antibacterial09900... at 50.00%
8     35aa, >antibacterial10844... at 45.71%
9     19aa, >antibacterial11661... at 42.11%
>Cluster 209
0     102aa, >Antibacterial... at 40.20%
1     18aa, >anti-Gram+;antibact... at 44.44%
2     123aa, >Antimicrobial... at 45.53%
3     95aa, >anti-Gram-;antifung... at 44.21%
4     98aa, >Antimicrobial... at 45.92%
5     98aa, >anti-Gram+;antifung... at 51.02%
6     97aa, >Antibacterial... at 42.27%
7     131aa, >Antibacterial... *
8     24aa, >antibacterial07806... at 50.00%
9     94aa, >antibacterial09737... at 57.45%
10    24aa, >antibacterial10364... at 50.00%
11    24aa, >antibacterial10367... at 45.83%
12    95aa, >antibacterial10883... at 35.00%
13    80aa, >antibacterial10978... at 47.50%
>Cluster 210
0     74aa, >Antimicrobial... at 68.92%
1     75aa, >Antimicrobial... at 68.00%
2     78aa, >Antimicrobial... at 70.51%
3     74aa, >Antimicrobial... at 63.51%
4     74aa, >Antimicrobial... at 64.86%
5     130aa, >Antimicrobial... *
6     36aa, >Antimicrobial... at 41.67%
7     64aa, >antibacterial06894... at 42.19%
8     64aa, >antibacterial07219... at 40.62%
9     36aa, >antibacterial09629... at 47.22%
10    23aa, >antibacterial09806... at 47.83%
11    28aa, >antibacterial12777... at 42.86%
```

```
>Cluster 211
0     126aa, >Antibacterial... at 46.03%
1     127aa, >Antibacterial... at 51.97%
2     128aa, >Antibacterial... at 53.91%
3     129aa, >Antimicrobial... at 55.81%
4     130aa, >Antibacterial... *
5     50aa, >Antifungal... at 35.00%
6     20aa, >antibacterial12403... at 35.00%
>Cluster 212
0     43aa, >Antibacterial;Antim... at 41.86%
1     107aa, >Antimicrobial... at 49.53%
2     125aa, >Antimicrobial... at 50.40%
3     124aa, >Antimicrobial... at 54.84%
4     128aa, >Antimicrobial... *
5     96aa, >Antimicrobial... at 48.96%
6     18aa, >antibacterial;antif... at 44.44%
7     19aa, >antibacterial07664... at 42.11%
8     18aa, >antibacterial07815... at 44.44%
9     20aa, >antibacterial07920... at 35.00%
10    18aa, >antibacterial08371... at 44.44%
11    20aa, >antibacterial09120... at 35.00%
12    18aa, >antibacterial09618... at 44.44%
13    18aa, >antibacterial09620... at 44.44%
>Cluster 213
0     128aa, >Antimicrobial... *
1     125aa, >Antimicrobial... at 68.80%
2     22aa, >antibacterial11638... at 40.91%
3     20aa, >antibacterial12163... at 50.00%
>Cluster 214
0     13aa, >antimicrobial... at 53.85%
1     23aa, >Antimicrobial... at 43.48%
2     40aa, >antimicrobial... at 35.00%
3     50aa, >anti-Gram+;antibact... at 35.00%
4     104aa, >Antibacterial... at 49.04%
5     92aa, >anti-Gram+;Antibact... at 63.04%
6     128aa, >anti-Gram+... *
7     37aa, >anti-Gram+;antibact... at 43.24%
8     20aa, >antibacterial09111... at 35.00%
9     20aa, >antibacterial09121... at 35.00%
10    20aa, >antibacterial09125... at 35.00%
11    19aa, >antibacterial09979... at 42.11%
12    37aa, >antibacterial10048... at 40.54%
>Cluster 215
0     118aa, >Antibacterial... at 56.78%
1     119aa, >Antibacterial;Antif... at 53.78%
2     119aa, >Antibacterial;Antif... *
3     21aa, >antibacterial;antim... at 42.86%
4     30aa, >antimicrobial... at 46.67%
5     128aa, >Antibacterial;Antif... *
6     13aa, >antibacterial08079... at 53.85%
7     19aa, >antibacterial09147... at 42.11%
>Cluster 216
0     127aa, >Antimicrobial... *
1     72aa, >antibacterial07476... at 66.67%
```

```
2     72aa, >antibacterial07478... *
3     13aa, >antibacterial07566... at 53.85%
4     20aa, >antibacterial08084... at 50.00%
>Cluster 217
0     27aa, >Antifungal... at 40.74%
1     25aa, >antibacterial;Antif... at 44.00%
2     32aa, >Antibacterial;Antif... at 40.62%
3     30aa, >antibacterial06571... *
4     24aa, >antibacterial08257... at 41.67%
5     18aa, >antibacterial08266... at 50.00%
6     17aa, >antibacterial08460... at 47.06%
7     18aa, >antibacterial08616... at 44.44%
8     22aa, >antibacterial09046... at 45.45%
9     13aa, >antibacterial09581... at 46.15%
10    11aa, >antibacterial09939... at 45.45%
11    14aa, >antibacterial10205... at 42.86%
12    13aa, >antibacterial10393... at 46.15%
13    13aa, >antibacterial12058... at 46.15%
14    26aa, >antibacterial12436... at 42.31%
15    15aa, >antibacterial13202... at 46.67%
16    127aa, >antibacterial13426... *
>Cluster 218
0     21aa, >antibacterial;antif... at 52.38%
1     114aa, >anti-Gram+;Antibact... at 62.28%
2     24aa, >antibacterial08396... at 41.67%
3     14aa, >antibacterial08470... at 42.86%
4     23aa, >antibacterial08884... at 43.48%
5     19aa, >antibacterial11457... at 42.11%
6     127aa, >antibacterial13432... *
>Cluster 219
0     25aa, >anti-Gram-;antibact... at 35.00%
1     126aa, >Antimicrobial... *
2     46aa, >antibacterial08983... at 41.30%
3     20aa, >antibacterial09379... at 35.00%
>Cluster 220
0     23aa, >antimicrobial... at 43.48%
1     126aa, >anti-Gram-... *
2     115aa, >Antibacterial... at 72.80%
3     28aa, >antibacterial06642... at 50.00%
4     32aa, >antibacterial07484... at 40.62%
5     18aa, >antibacterial08512... at 44.44%
6     20aa, >antibacterial12931... at 35.00%
>Cluster 221
0     115aa, >Antimicrobial... at 46.09%
1     126aa, >Antimicrobial... *
2     74aa, >antibacterial07475... at 55.41%
3     20aa, >antibacterial12707... at 45.00%
>Cluster 222
0     63aa, >Antibacterial... *
1     63aa, >Antibacterial... at 71.43%
2     59aa, >Antimicrobial... at 67.80%
3     83aa, >Antimicrobial... at 54.22%
4     83aa, >Antimicrobial... at 50.60%
5     83aa, >Antimicrobial... at 53.01%
```

```
6     81aa, >Antimicrobial... at 60.49%
7     84aa, >Antimicrobial... at 60.71%
8     84aa, >Antimicrobial... at 60.71%
9     126aa, >Antimicrobial... *
10    63aa, >Antimicrobial... at 69.84%
11    63aa, >Antimicrobial... at 73.02%
12    103aa, >Antimicrobial... at 48.54%
13    26aa, >antibacterial06680... at 42.31%
14    83aa, >antibacterial06859... at 56.63%
15    26aa, >antibacterial07061... at 50.00%
16    18aa, >antibacterial08053... at 55.56%
17    18aa, >antibacterial09486... at 44.44%
18    13aa, >antibacterial09582... at 46.15%
19    14aa, >antibacterial10198... at 42.86%
20    19aa, >antibacterial12430... at 42.11%
21    26aa, >antibacterial12442... at 50.00%
>Cluster 223
0     40aa, >antibacterial;antim... at 35.00%
1     125aa, >Antimicrobial... *
2     20aa, >anti-Gram+;antibact... at 45.00%
3     28aa, >antibacterial12749... at 42.86%
>Cluster 224
0     57aa, >Antibacterial... at 47.37%
1     30aa, >Anti-Gram-;antibact... at 35.00%
2     88aa, >Antibacterial... at 54.55%
3     88aa, >Antibacterial... at 59.09%
4     125aa, >Antibacterial... *
5     88aa, >Antibacterial... at 54.55%
6     88aa, >Antibacterial... at 55.68%
>Cluster 225
0     28aa, >antimicrobial... at 42.86%
1     29aa, >antimicrobial... at 41.38%
2     29aa, >antimicrobial... at 41.38%
3     120aa, >anti-Gram+;Antimicr... at 64.17%
4     97aa, >anti-Gram+;antifung... at 74.23%
5     30aa, >antibacterial07497... at 46.67%
6     20aa, >antibacterial07552... at 45.00%
7     47aa, >antibacterial10221... at 44.68%
8     19aa, >antibacterial11442... at 42.11%
9     125aa, >antibacterial13423... *
>Cluster 226
0     124aa, >Antimicrobial... *
1     67aa, >Antimicrobial... at 46.27%
2     37aa, >anti-Gram+;antibact... at 40.54%
3     24aa, >antibacterial07069... at 41.67%
4     24aa, >antibacterial08133... at 41.67%
5     17aa, >antibacterial10368... at 41.18%
6     13aa, >antibacterial10471... at 46.15%
7     15aa, >antibacterial11453... at 35.00%
8     13aa, >antibacterial12056... at 46.15%
9     13aa, >antibacterial12981... at 46.15%
>Cluster 227
0     35aa, >antimicrobial... at 35.00%
1     46aa, >antibacterial;Antim... at 47.83%
```

2   46aa, >Antibacterial... at 45.65%
3   121aa, >Antimicrobial... at 79.34%
4   124aa, >Antimicrobial... *
5   46aa, >Antimicrobial... at 50.00%
>Cluster 228
0   122aa, >Antibacterial... at 50.82%
1   122aa, >Antibacterial... at 48.36%
2   124aa, >anti-Gram+;Gram-... *
3   118aa, >Antibacterial... at 40.68%
4   21aa, >antimicrobial... at 47.62%
5   121aa, >anti-Gram+;Gram-... at 46.28%
6   121aa, >anti-Gram+;Antibact... at 51.24%
7   122aa, >Antibacterial... at 50.00%
8   122aa, >Antibacterial... at 45.90%
9   12aa, >antibacterial07900... at 41.67%
10   25aa, >antibacterial10299... at 35.00%
11   25aa, >antibacterial10711... at 35.00%
12   19aa, >antibacterial11405... at 47.37%
>Cluster 229
0   87aa, >Antimicrobial... at 42.53%
1   42aa, >Antimicrobial... at 50.00%
2   42aa, >Antimicrobial... at 34.76%
3   104aa, >Antimicrobial... at 45.19%
4   19aa, >anti-Gram+;antibact... at 42.11%
5   53aa, >Antimicrobial... at 52.83%
6   81aa, >Antimicrobial... at 40.74%
7   122aa, >Antimicrobial... at 44.26%
8   123aa, >Antimicrobial... *
9   123aa, >Antimicrobial... at 82.11%
10   123aa, >Antimicrobial... at 81.30%
11   69aa, >Antimicrobial... at 42.03%
12   67aa, >Antimicrobial... at 33.28%
13   123aa, >Antimicrobial... at 40.65%
14   102aa, >anti-Gram-... *
15   100aa, >Antimicrobial... at 81.26%
16   103aa, >Antimicrobial... at 78.35%
17   83aa, >antibacterial07183... at 48.19%
18   32aa, >antibacterial11463... at 46.88%
19   24aa, >antibacterial12448... at 45.83%
20   27aa, >antibacterial12779... at 40.74%
21   15aa, >antibacterial13170... at 33.10%
>Cluster 230
0   39aa, >anti-Gram+;antibact... *
1   29aa, >Gram-... at 41.38%
2   38aa, >anti-Gram+;Gram-... at 68.42%
3   123aa, >anti-Gram+... *
4   31aa, >antibacterial07401... at 41.94%
5   34aa, >antibacterial08561... at 41.18%
6   27aa, >antibacterial10257... at 34.44%
7   18aa, >antibacterial10704... at 34.44%
8   52aa, >antibacterial10825... at 74.62%
9   20aa, >antibacterial11817... at 35.00%
10   21aa, >antibacterial12876... at 37.14%
>Cluster 231

```
0     122aa, >Antibacterial... *
1     50aa, >antibacterial;antim... at 42.00%
2     37aa, >anti-Gram+;antibact... at 43.24%
3     23aa, >antimicrobial... at 43.48%
4     14aa, >antibacterial07756... at 42.86%
5     20aa, >antibacterial11320... at 35.00%
6     24aa, >antibacterial11779... at 45.83%
>Cluster 232
0     122aa, >Antimicrobial... *
1     96aa, >Antimicrobial... at 40.62%
2     16aa, >antibacterial12111... at 43.75%
>Cluster 233
0     121aa, >anti-Gram+... *
1     35aa, >anti-Gram+;antibact... at 35.00%
2     12aa, >antibacterial12699... at 41.67%
>Cluster 234
0     32aa, >Antimicrobial... at 46.88%
1     25aa, >Antibacterial;Gram-... at 44.00%
2     32aa, >antimicrobial... at 46.88%
3     37aa, >anti-HIV;antiviral... at 45.95%
4     28aa, >antimicrobial... at 50.00%
5     30aa, >Antimicrobial... at 53.33%
6     30aa, >Antimicrobial... at 53.33%
7     34aa, >antimicrobial... at 44.12%
8     34aa, >antibacterial... at 41.18%
9     29aa, >anti-Gram-;antibact... at 65.52%
10    29aa, >antimicrobial... *
11    31aa, >anti-Gram+;anti-HIV... at 45.16%
12    30aa, >Antimicrobial;Antim... at 80.00%
13    121aa, >Antimicrobial... *
14    27aa, >Antimicrobial... at 44.44%
15    31aa, >antibacterial06486... at 48.39%
16    37aa, >antibacterial07505... at 45.95%
17    30aa, >antibacterial09128... at 83.33%
18    33aa, >antibacterial10751... at 45.45%
19    29aa, >antibacterial11445... at 62.07%
20    25aa, >antibacterial11863... at 35.00%
21    29aa, >antibacterial13221... *
>Cluster 235
0     121aa, >anti-HIV;antiviral... *
1     20aa, >antibacterial07442... at 35.00%
>Cluster 236
0     120aa, >Antimicrobial... *
1     120aa, >Antimicrobial... at 80.00%
2     27aa, >antibacterial09764... at 40.74%
>Cluster 237
0     102aa, >Antimicrobial... at 56.86%
1     106aa, >Antimicrobial... at 59.43%
2     106aa, >Antimicrobial... at 50.94%
3     106aa, >Antimicrobial... at 54.72%
4     27aa, >Antimicrobial... at 44.44%
5     95aa, >Antimicrobial... at 50.53%
6     98aa, >Antimicrobial... at 45.92%
7     115aa, >Antimicrobial... at 58.26%
```

```
8     111aa, >Antimicrobial... at 48.65%
9     113aa, >Antimicrobial... at 57.52%
10    118aa, >Antimicrobial... at 55.93%
11    120aa, >Antimicrobial... *
12    100aa, >Antimicrobial... at 49.00%
13    19aa, >antibacterial08645... at 42.11%
14    23aa, >antibacterial09073... at 47.83%
15    20aa, >antibacterial09393... at 35.00%
16    24aa, >antibacterial09704... at 41.67%
17    20aa, >antibacterial10036... *
18    23aa, >antibacterial12365... at 43.48%
19    27aa, >antibacterial12892... at 44.44%
>Cluster 238
0     83aa, >anti-Gram-... at 46.99%
1     82aa, >Antibacterial... at 42.68%
2     120aa, >Gram-... *
3     22aa, >Gram-... at 40.91%
>Cluster 239
0     36aa, >Antimicrobial... at 72.22%
1     119aa, >Antimicrobial... *
2     50aa, >anti-Gram+;antibact... at 46.00%
3     99aa, >antibacterial06798... at 40.40%
4     19aa, >antibacterial09144... at 47.37%
5     15aa, >antibacterial11588... at 53.33%
>Cluster 240
0     38aa, >Antimicrobial... at 47.37%
1     98aa, >Antimicrobial... at 40.82%
2     104aa, >Antimicrobial... at 40.38%
3     117aa, >Antimicrobial... at 66.67%
4     119aa, >anti-Gram+;Gram-... *
5     87aa, >Antimicrobial... at 57.47%
6     33aa, >antibacterial07590... at 45.45%
7     18aa, >antibacterial08106... at 44.44%
8     14aa, >antibacterial10380... at 42.86%
9     20aa, >antibacterial12889... at 35.00%
10    18aa, >antibacterial13251... at 50.00%
>Cluster 241
0     31aa, >Anti-Gram-... at 41.94%
1     119aa, >Antibacterial... *
2     32aa, >anti-Gram+;antibact... at 40.62%
3     13aa, >antibacterial07015... at 46.15%
4     18aa, >antibacterial07810... at 55.56%
5     32aa, >antibacterial08553... at 40.62%
6     20aa, >antibacterial09301... at 50.00%
7     21aa, >antibacterial09506... at 42.86%
8     18aa, >antibacterial10784... at 44.44%
9     18aa, >antibacterial10785... at 50.00%
10    14aa, >antibacterial10866... at 50.00%
>Cluster 242
0     112aa, >Antimicrobial... at 40.18%
1     21aa, >antibacterial;antim... at 42.86%
2     104aa, >Antimicrobial... at 41.35%
3     36aa, >Gram-... at 47.22%
4     118aa, >Antimicrobial... *
```

5   20aa, >antibacterial09387... at 45.00%
6   20aa, >antibacterial12161... at 35.00%
>Cluster 243
0   118aa, >Antimicrobial... *
1   41aa, >Antimicrobial... at 43.90%
2   24aa, >antibacterial12984... at 41.67%
>Cluster 244
0   40aa, >Antimicrobial... at 35.00%
1   117aa, >Antimicrobial... *
2   49aa, >antithrombotic... at 57.14%
3   32aa, >Antimicrobial... at 45.62%
4   29aa, >antibacterial... at 48.28%
5   24aa, >antimicrobial... at 41.67%
6   43aa, >antithrombotic... at 44.42%
7   25aa, >Antimicrobial... *
8   27aa, >antimicrobial... at 40.74%
9   26aa, >antibacterial;antib... at 39.23%
10   39aa, >antimicrobial... at 43.59%
11   29aa, >antimicrobial... at 58.62%
12   67aa, >Antimicrobial... at 44.78%
13   26aa, >antibacterial06551... at 34.62%
14   26aa, >antibacterial06676... at 41.54%
15   70aa, >antibacterial06819... at 48.57%
16   30aa, >antibacterial07058... at 56.67%
17   23aa, >L01A000169... at 56.52%
18   23aa, >antibacterial07961... at 52.17%
19   27aa, >antibacterial07981... at 48.15%
20   30aa, >antibacterial09036... at 56.67%
21   21aa, >antibacterial09471... at 42.86%
22   26aa, >antibacterial10222... at 42.31%
23   29aa, >antibacterial10300... at 44.83%
24   28aa, >antibacterial10301... at 46.43%
25   26aa, >antibacterial10521... *
26   30aa, >antibacterial10733... at 35.00%
27   34aa, >antibacterial10932... at 54.71%
28   37aa, >antibacterial12426... at 43.24%
>Cluster 245
0   117aa, >Antibacterial... *
1   34aa, >antimicrobial... at 41.18%
2   18aa, >antibacterial06540... at 44.44%
3   21aa, >antibacterial08620... at 42.86%
4   21aa, >antibacterial08922... at 42.86%
5   18aa, >antibacterial10705... at 44.44%
6   17aa, >antibacterial10901... at 41.18%
7   16aa, >antibacterial12942... at 43.75%
>Cluster 246
0   24aa, >antimicrobial... at 41.67%
1   117aa, >Gram-... *
2   28aa, >antibacterial08575... at 42.86%
>Cluster 247
0   84aa, >Antibacterial;Antif... at 47.62%
1   115aa, >Antimicrobial... at 47.83%
2   117aa, >Antibacterial;Antif... *
3   92aa, >Antimicrobial... at 61.96%

```
4     79aa, >Antibacterial... at 43.04%
5     29aa, >Antimicrobial... at 41.38%
6     11aa, >antibacterial10282... at 45.45%
7     32aa, >antibacterial10880... at 40.62%
8     33aa, >antibacterial11176... at 42.42%
>Cluster 248
0     116aa, >Antibacterial... *
>Cluster 249
0     32aa, >anti-Gram+;antimicr... at 40.62%
1     31aa, >antimicrobial... at 41.94%
2     28aa, >antimicrobial... at 42.86%
3     115aa, >Antimicrobial... *
4     111aa, >Antimicrobial... at 61.26%
5     111aa, >Antimicrobial... at 62.16%
6     13aa, >antibacterial10843... at 46.15%
>Cluster 250
0     91aa, >Antimicrobial... at 82.42%
1     84aa, >Antimicrobial... *
2     115aa, >Antimicrobial... *
3     91aa, >Antimicrobial... at 42.86%
4     91aa, >Antimicrobial... at 60.44%
5     91aa, >anti-Gram+;antifung... at 58.24%
6     88aa, >Antimicrobial... at 56.82%
7     98aa, >Antimicrobial... at 60.20%
8     21aa, >antibacterial07847... at 42.86%
>Cluster 251
0     114aa, >anti-HIV;antiviral... *
1     14aa, >antibacterial09895... at 42.86%
>Cluster 252
0     42aa, >Antibacterial... at 42.86%
1     114aa, >Antimicrobial... *
2     111aa, >Antimicrobial... at 80.99%
3     108aa, >Antimicrobial... at 76.74%
>Cluster 253
0     114aa, >Antifungal... *
1     95aa, >antibacterial10813... at 45.26%
>Cluster 254
0     114aa, >Antibacterial... *
1     28aa, >antibacterial06500... at 42.86%
2     16aa, >antibacterial12617... at 43.75%
>Cluster 255
0     45aa, >antibacterial;Antif... at 35.00%
1     47aa, >Antibacterial;Antif... at 40.43%
2     26aa, >Antibacterial;Antif... at 46.15%
3     34aa, >antibacterial;antim... at 44.12%
4     30aa, >anti-Gram+;Antibact... at 43.33%
5     113aa, >Antimicrobial... *
6     108aa, >Antimicrobial... at 79.63%
7     20aa, >Antibacterial... at 35.00%
8     29aa, >antibacterial;antim... at 41.38%
9     18aa, >antibacterial08267... *
10    18aa, >antibacterial08679... at 44.44%
11    18aa, >antibacterial09485... at 44.44%
12    11aa, >antibacterial09820... at 45.45%
```

13   24aa, >antibacterial10003... at 41.67%
14   28aa, >antibacterial10310... at 42.86%
15   21aa, >antibacterial10898... at 42.86%
16   28aa, >antibacterial12972... at 42.86%
>Cluster 256
0   20aa, >antimicrobial... at 35.00%
1   27aa, >antimicrobial... at 44.44%
2   24aa, >Antimicrobial... at 41.67%
3   19aa, >anti-Gram+;Antibact... at 42.11%
4   30aa, >antimicrobial... at 35.00%
5   87aa, >Antimicrobial... at 50.57%
6   112aa, >Antimicrobial... *
7   29aa, >anti-Gram+;antimicr... at 44.83%
8   18aa, >antibacterial11948... at 44.44%
>Cluster 257
0   112aa, >Antibacterial... *
>Cluster 258
0   44aa, >Antimicrobial... at 65.91%
1   88aa, >Antimicrobial... at 45.45%
2   50aa, >Antimicrobial... *
3   111aa, >Antimicrobial... *
4   44aa, >Antimicrobial... at 56.82%
5   49aa, >Antimicrobial... at 40.82%
6   19aa, >anti-Gram-;antibact... at 42.11%
7   81aa, >Antimicrobial... at 40.74%
8   72aa, >Antimicrobial... at 43.06%
9   73aa, >Antimicrobial... at 41.10%
10   73aa, >Antimicrobial... at 43.84%
11   72aa, >Antimicrobial... at 40.28%
12   73aa, >Antimicrobial... at 42.47%
13   72aa, >Antimicrobial... at 41.67%
14   72aa, >Antimicrobial... at 40.28%
15   54aa, >Antimicrobial... at 77.78%
16   19aa, >antibacterial... at 42.11%
17   52aa, >Antimicrobial... at 57.69%
18   42aa, >Antimicrobial... at 52.38%
19   18aa, >antibacterial07409... at 44.44%
20   18aa, >antibacterial07413... at 44.44%
21   39aa, >antibacterial07904... *
22   39aa, >antibacterial07906... at 58.97%
23   18aa, >antibacterial08076... at 61.11%
24   17aa, >antibacterial08241... at 41.18%
25   22aa, >antibacterial10186... at 59.09%
26   23aa, >antibacterial10878... at 47.83%
27   72aa, >antibacterial11068... at 43.06%
>Cluster 259
0   21aa, >antimicrobial... at 47.62%
1   110aa, >Antibacterial... at 75.45%
2   111aa, >Antimicrobial... *
3   36aa, >Antibacterial;Antim... at 41.67%
4   90aa, >Antimicrobial... at 80.00%
5   88aa, >antibacterial06559... at 62.50%
>Cluster 260
0   44aa, >Antimicrobial... at 40.91%

1   40aa, >Antimicrobial... at 35.00%
2   39aa, >antimicrobial... at 43.59%
3   40aa, >antibacterial;antim... at 42.50%
4   39aa, >Antimicrobial... at 46.15%
5   40aa, >anti-Gram+;antibact... at 42.50%
6   39aa, >Antimicrobial... at 48.72%
7   46aa, >anti-Gram+;antibact... at 45.65%
8   40aa, >antimicrobial... at 45.00%
9   40aa, >antimicrobial... at 35.00%
10   24aa, >antibacterial;Antif... at 41.67%
11   40aa, >Antibacterial;antim... at 50.00%
12   43aa, >Antibacterial;antim... at 44.19%
13   29aa, >anti-Gram+;Antibact... at 41.38%
14   36aa, >antimicrobial;Antim... at 41.67%
15   36aa, >antimicrobial;Antim... at 44.44%
16   111aa, >Antimicrobial... *
17   29aa, >Antimicrobial;Antiv... at 41.38%
18   42aa, >antifungal;antimicr... at 40.48%
19   29aa, >Antibacterial... *
20   39aa, >Antimicrobial... at 46.15%
21   40aa, >Antimicrobial... at 47.50%
22   51aa, >Antimicrobial... at 41.18%
23   43aa, >antibacterial07546... at 41.86%
24   40aa, >antibacterial10748... at 42.50%
>Cluster 261
0   51aa, >Antibacterial... at 66.67%
1   24aa, >antibacterial;Antif... at 41.67%
2   51aa, >Antibacterial... at 66.67%
3   42aa, >antimicrobial... at 40.48%
4   52aa, >Antibacterial... at 65.38%
5   111aa, >Antibacterial... *
6   25aa, >antibacterial;antim... at 48.00%
7   52aa, >Antibacterial... at 73.08%
8   26aa, >antibacterial10839... at 42.31%
9   31aa, >antibacterial11438... at 45.16%
>Cluster 262
0   111aa, >antibacterial13427... *
>Cluster 263
0   110aa, >Antimicrobial... *
>Cluster 264
0   30aa, >anti-Gram+... at 35.00%
1   110aa, >Antimicrobial... *
2   18aa, >antibacterial12789... at 44.44%
>Cluster 265
0   93aa, >Gram-... at 43.01%
1   38aa, >Antibacterial... at 42.11%
2   91aa, >anti-Gram+;Gram-... at 69.23%
3   109aa, >Antimicrobial... *
4   13aa, >antimicrobial... at 46.15%
>Cluster 266
0   108aa, >Antifungal... *
1   40aa, >anti-Gram+;anti-HIV... at 87.50%
2   19aa, >antibacterial12943... at 42.11%
>Cluster 267

0   108aa, >Antibacterial... *
1   18aa, >antibacterial08074... at 44.44%
>Cluster 268
0   27aa, >antimicrobial... at 40.74%
1   24aa, >anti-Gram+;antibact... at 41.67%
2   26aa, >antimicrobial... at 42.31%
3   108aa, >Antimicrobial... *
4   105aa, >Antimicrobial... at 69.52%
5   31aa, >antibacterial07387... at 41.94%
6   25aa, >antibacterial08004... *
7   26aa, >antibacterial08007... at 78.00%
8   26aa, >antibacterial08009... at 78.00%
9   24aa, >antibacterial08011... at 80.00%
10   20aa, >antibacterial11566... at 35.00%
>Cluster 269
0   43aa, >Antimicrobial... at 41.86%
1   62aa, >Antimicrobial... at 72.58%
2   62aa, >Antibacterial... at 74.19%
3   37aa, >anti-Gram-;antibact... at 64.86%
4   31aa, >antimicrobial... at 41.94%
5   35aa, >Antimicrobial... *
6   35aa, >Antimicrobial... at 80.00%
7   108aa, >Antibacterial... *
8   68aa, >antibacterial06893... at 57.35%
9   52aa, >antibacterial07295... at 71.15%
10   24aa, >antibacterial10946... at 41.67%
11   80aa, >antibacterial11038... at 78.75%
12   29aa, >antibacterial13027... at 41.38%
>Cluster 270
0   107aa, >Antibacterial... *
1   30aa, >Antibacterial... at 35.00%
>Cluster 271
0   18aa, >antibacterial... at 44.44%
1   24aa, >antimicrobial... at 45.83%
2   107aa, >Antimicrobial... *
>Cluster 272
0   24aa, >antimicrobial... at 41.67%
1   107aa, >Antimicrobial... *
2   20aa, >antibacterial07921... at 45.00%
3   23aa, >antibacterial07925... at 43.48%
4   25aa, >antibacterial11431... at 35.00%
5   20aa, >antibacterial12612... at 35.00%
6   18aa, >antibacterial12849... at 44.44%
>Cluster 273
0   57aa, >anti-Gram+;anti-HIV... at 47.37%
1   16aa, >antimicrobial... at 56.25%
2   16aa, >antibacterial06607... at 43.75%
3   107aa, >antibacterial13428... *
>Cluster 274
0   26aa, >antimicrobial... at 42.31%
1   77aa, >Antimicrobial... at 48.05%
2   75aa, >Antimicrobial... at 35.00%
3   78aa, >Antimicrobial... at 53.85%
4   78aa, >Antimicrobial... at 50.00%

```
5     42aa, >Antimicrobial... at 80.95%
6     92aa, >Antimicrobial... at 44.57%
7     94aa, >Antimicrobial... at 50.00%
8     106aa, >Antimicrobial... *
9     96aa, >Antimicrobial... at 42.71%
10    40aa, >Antimicrobial... at 35.00%
11    41aa, >anti-Gram-... at 58.54%
12    77aa, >Antimicrobial... at 40.26%
13    11aa, >antimicrobial... at 45.45%
14    43aa, >Antimicrobial... at 53.49%
15    60aa, >Antimicrobial... at 45.00%
16    44aa, >Antimicrobial... *
17    65aa, >Antimicrobial... at 41.54%
18    41aa, >antibacterial07235... at 63.41%
19    46aa, >antibacterial12664... at 73.91%
>Cluster 275
0     106aa, >anti-Gram+... *
>Cluster 276
0     102aa, >Antimicrobial... *
1     64aa, >Antimicrobial... at 42.19%
2     77aa, >Antimicrobial... at 72.73%
3     63aa, >Antimicrobial... at 65.08%
4     76aa, >Antimicrobial... at 36.84%
5     78aa, >Antimicrobial... at 48.72%
6     105aa, >Antimicrobial... *
7     75aa, >Antimicrobial... at 35.00%
8     77aa, >Antimicrobial... at 83.12%
9     77aa, >Antimicrobial... at 81.82%
10    77aa, >Antimicrobial... *
11    77aa, >Antimicrobial... at 84.42%
12    77aa, >Antimicrobial... at 81.82%
13    77aa, >Antimicrobial... at 83.12%
14    77aa, >Antimicrobial... at 79.22%
15    77aa, >Antimicrobial... at 84.42%
16    77aa, >Antimicrobial... at 85.71%
17    59aa, >Antimicrobial... *
18    59aa, >Antimicrobial... at 80.14%
19    76aa, >Antimicrobial... at 82.89%
20    75aa, >Antimicrobial... *
21    76aa, >Antimicrobial... at 84.21%
22    77aa, >antibacterial07256... at 80.52%
23    11aa, >antibacterial09335... at 45.45%
24    81aa, >antibacterial11093... at 66.67%
25    77aa, >antibacterial11094... *
>Cluster 277
0     24aa, >Antifungal;Antimicr... at 41.67%
1     82aa, >anti-Gram+;Antibact... *
2     102aa, >Antimicrobial... at 78.43%
3     67aa, >Antimicrobial... at 40.30%
4     104aa, >Antimicrobial... *
5     104aa, >Antimicrobial... at 74.04%
6     66aa, >Antibacterial;Antif... at 42.42%
7     25aa, >antibacterial;antif... at 35.00%
8     13aa, >antimicrobial... at 31.54%
```

9   55aa, >anti-Gram+;Antibact... at 41.82%
>Cluster 278
0   104aa, >Antimicrobial... *
1   21aa, >Antimicrobial... at 37.62%
2   13aa, >antibacterial10851... at 53.85%
3   18aa, >antibacterial13103... at 44.44%
>Cluster 279
0   104aa, >Antimicrobial... *
1   23aa, >antibacterial11791... at 43.48%
>Cluster 280
0   23aa, >anti-Gram+;antibact... at 43.48%
1   23aa, >anti-Gram+;antimicr... at 43.48%
2   104aa, >anti-Gram+;Antibact... *
3   15aa, >antibacterial13253... at 35.00%
>Cluster 281
0   103aa, >Antimicrobial... *
1   96aa, >Antimicrobial... at 62.50%
2   18aa, >antibacterial06536... at 44.44%
3   29aa, >antibacterial07844... at 41.38%
>Cluster 282
0   103aa, >Antimicrobial... *
>Cluster 283
0   84aa, >Antimicrobial... at 52.38%
1   49aa, >Antimicrobial... at 42.86%
2   103aa, >Antimicrobial... *
3   61aa, >Antimicrobial... at 40.98%
4   75aa, >Antimicrobial... at 46.67%
5   75aa, >Antimicrobial... at 41.33%
6   13aa, >antimicrobial... at 46.15%
7   27aa, >antibacterial06679... at 40.74%
8   35aa, >antibacterial07439... at 35.00%
9   29aa, >antibacterial10307... at 41.38%
10   39aa, >antibacterial12618... at 41.03%
>Cluster 284
0   37aa, >anti-Gram+;antibact... at 43.24%
1   19aa, >anti-Gram+;antibact... at 42.11%
2   20aa, >anti-Gram+;antibact... at 45.00%
3   103aa, >Antimicrobial... *
4   103aa, >Antimicrobial... at 77.67%
5   101aa, >Antimicrobial... at 64.36%
6   31aa, >antibacterial... at 77.42%
7   22aa, >antimicrobial... at 40.91%
8   22aa, >antimicrobial... at 40.91%
9   18aa, >antibacterial07410... at 44.44%
10   19aa, >antibacterial07411... at 42.11%
11   36aa, >antibacterial07550... at 58.33%
12   18aa, >antibacterial07812... *
13   18aa, >antibacterial08411... at 44.44%
14   22aa, >antibacterial08416... at 40.91%
15   19aa, >antibacterial09550... at 44.44%
16   19aa, >antibacterial09714... at 44.44%
17   13aa, >antibacterial13292... at 46.15%
>Cluster 285
0   43aa, >Antibacterial;antim... at 65.12%

1 45aa, >Antibacterial;Antim... at 42.22%
2 42aa, >Antibacterial;Antim... at 76.19%
3 48aa, >Antimicrobial... at 43.75%
4 27aa, >Antimicrobial... at 40.74%
5 103aa, >Antibacterial... *
6 85aa, >Antibacterial... at 80.59%
7 22aa, >antibacterial06592... at 40.91%
8 92aa, >antibacterial06814... at 47.83%
9 43aa, >antibacterial07547... at 62.79%
10 24aa, >antibacterial07665... at 41.67%
>Cluster 286
0 27aa, >antimicrobial... at 44.44%
1 103aa, >anti-Gram-... *
2 28aa, >antibacterial07065... at 42.86%
3 24aa, >antibacterial09879... at 41.67%
4 15aa, >antibacterial10168... at 35.00%
5 37aa, >antibacterial12390... at 45.95%
>Cluster 287
0 73aa, >anti-Gram+;Antibact... at 47.95%
1 73aa, >anti-Gram+;Antibact... at 42.47%
2 73aa, >anti-Gram+;Antibact... at 42.47%
3 20aa, >Antifungal;antivira... at 45.00%
4 18aa, >Antimicrobial... at 44.44%
5 28aa, >antibacterial06691... at 42.86%
6 103aa, >antibacterial13425... *
>Cluster 288
0 100aa, >anti-Gram+... at 64.00%
1 102aa, >Antibacterial... *
2 98aa, >anti-Gram+;Antimicr... at 89.14%
>Cluster 289
0 102aa, >Antimicrobial... *
1 41aa, >anti-Gram+;antibact... at 41.46%
>Cluster 290
0 24aa, >antimicrobial... at 41.67%
1 46aa, >anti-Gram+;Antibact... at 41.30%
2 46aa, >antimicrobial... at 41.30%
3 102aa, >Antimicrobial... *
4 102aa, >Antimicrobial... at 75.49%
5 32aa, >antibacterial08494... at 40.62%
6 26aa, >antibacterial09001... at 42.31%
7 30aa, >antibacterial10610... at 43.33%
>Cluster 291
0 25aa, >anti-Gram+;antibact... at 35.00%
1 78aa, >Antimicrobial... at 64.10%
2 85aa, >Antimicrobial... at 55.29%
3 93aa, >Antimicrobial... at 67.74%
4 57aa, >Antimicrobial... at 50.88%
5 62aa, >Antimicrobial... at 40.32%
6 64aa, >Antimicrobial... at 42.19%
7 67aa, >Antimicrobial... at 52.24%
8 66aa, >Antimicrobial... at 50.00%
9 81aa, >Antimicrobial... at 50.62%
10 68aa, >Antimicrobial... at 60.29%
11 71aa, >Antimicrobial... at 56.34%

```
12    71aa, >Antimicrobial... at 64.79%
13    74aa, >Antimicrobial... *
14    102aa, >Antimicrobial... *
15    30aa, >antibacterial13299... at 35.00%
>Cluster 292
0     101aa, >anti-Gram-;antibact... *
1     19aa, >antibacterial08579... at 42.11%
2     13aa, >antibacterial09216... at 46.15%
3     13aa, >antibacterial09219... at 46.15%
4     21aa, >antibacterial09296... at 71.43%
5     21aa, >antibacterial09302... at 47.62%
6     19aa, >antibacterial09303... at 47.37%
7     14aa, >antibacterial10203... at 50.00%
>Cluster 293
0     22aa, >anti-Gram+;antibact... at 40.91%
1     101aa, >anti-HIV;antiviral... *
>Cluster 294
0     45aa, >Antimicrobial... at 42.22%
1     41aa, >antifungal;antimicr... at 41.46%
2     43aa, >Antibacterial;antim... at 41.86%
3     43aa, >Antimicrobial... *
4     43aa, >anti-Gram+;antibact... at 53.49%
5     43aa, >Antimicrobial... at 51.16%
6     43aa, >Antibacterial... at 53.49%
7     43aa, >Antimicrobial... at 51.16%
8     40aa, >antimicrobial... at 57.50%
9     45aa, >Antimicrobial... at 55.56%
10    43aa, >Antibacterial... at 53.49%
11    40aa, >antimicrobial... at 57.50%
12    48aa, >Antimicrobial... at 47.92%
13    37aa, >Antimicrobial... at 40.54%
14    38aa, >Antimicrobial... at 42.11%
15    23aa, >antimicrobial... at 43.48%
16    42aa, >Antimicrobial... at 52.38%
17    47aa, >antifungal;antimicr... at 42.55%
18    101aa, >Antimicrobial... *
19    101aa, >Antimicrobial... at 72.28%
20    97aa, >Antimicrobial... at 42.27%
21    52aa, >anti-Gram+;Antibact... at 57.69%
22    47aa, >antifungal;antimicr... at 40.43%
23    32aa, >Antimicrobial... at 46.88%
24    43aa, >Antimicrobial... at 60.47%
25    51aa, >Antimicrobial... at 70.59%
26    51aa, >Antimicrobial... *
27    51aa, >Antimicrobial... at 72.55%
28    43aa, >Antimicrobial... at 62.79%
29    42aa, >Antimicrobial... at 57.14%
30    52aa, >anti-Gram+;Antibact... *
31    28aa, >antibacterial... at 42.86%
32    94aa, >antibacterial06811... at 43.62%
33    97aa, >antibacterial06812... at 43.30%
34    79aa, >antibacterial06817... at 41.77%
35    84aa, >antibacterial06857... at 41.67%
36    94aa, >antibacterial06866... at 40.43%
```

37 57aa, >antibacterial06953... at 47.37%
38 79aa, >antibacterial07226... at 44.30%
39 96aa, >antibacterial07249... at 59.38%
40 43aa, >antibacterial07544... *
41 43aa, >antibacterial07545... at 58.14%
42 43aa, >antibacterial10745... at 46.51%
43 45aa, >antibacterial10746... at 42.22%
44 41aa, >antibacterial10747... at 53.66%
45 40aa, >antibacterial10749... at 45.00%
46 65aa, >antibacterial10884... at 52.31%
47 95aa, >antibacterial11007... at 58.95%
48 98aa, >antibacterial11084... at 40.82%
49 99aa, >antibacterial11087... at 40.40%
50 21aa, >antibacterial12647... at 47.62%
>Cluster 295
0 25aa, >Antimicrobial... at 35.00%
1 101aa, >Antimicrobial... *
2 28aa, >antibacterial07570... at 42.86%
3 18aa, >antibacterial08892... at 44.44%
4 21aa, >antibacterial09348... at 42.86%
5 39aa, >antibacterial12721... at 43.59%
>Cluster 296
0 94aa, >Antimicrobial... at 50.00%
1 94aa, >Antimicrobial... at 44.68%
2 97aa, >Antimicrobial... at 46.39%
3 101aa, >Antimicrobial... *
4 101aa, >Antimicrobial... at 74.26%
5 94aa, >Antimicrobial... at 52.13%
6 94aa, >Antimicrobial... at 45.74%
7 93aa, >Antimicrobial... at 45.16%
8 94aa, >Antimicrobial... at 47.87%
9 94aa, >Antimicrobial... at 51.06%
10 94aa, >Antimicrobial... at 46.81%
11 94aa, >Antimicrobial... at 44.68%
12 94aa, >Antimicrobial... at 44.68%
13 84aa, >Antimicrobial... at 41.67%
14 94aa, >Antimicrobial... at 75.53%
15 100aa, >Antimicrobial... at 44.00%
16 94aa, >Antimicrobial... at 51.06%
17 100aa, >Antimicrobial... at 47.00%
18 100aa, >Antimicrobial... at 46.00%
19 100aa, >Antimicrobial... at 44.00%
20 96aa, >Antimicrobial... at 83.33%
21 81aa, >Antimicrobial... *
22 27aa, >antibacterial;antim... at 44.44%
23 27aa, >antimicrobial... at 40.74%
24 34aa, >antimicrobial... at 44.12%
25 59aa, >antibacterial06467... at 47.46%
26 43aa, >antibacterial07182... at 46.51%
27 88aa, >antibacterial07194... at 45.45%
28 76aa, >antibacterial07195... at 46.05%
29 76aa, >antibacterial07196... at 46.05%
30 87aa, >antibacterial07197... at 45.98%
31 94aa, >antibacterial07199... at 43.62%

```
32    94aa, >antibacterial07200... at 45.74%
33    95aa, >antibacterial07201... at 44.21%
34    95aa, >antibacterial07202... at 35.00%
35    93aa, >antibacterial07208... at 44.09%
36    94aa, >antibacterial07209... at 43.62%
37    94aa, >antibacterial07210... at 47.87%
38    94aa, >antibacterial07211... at 48.94%
39    34aa, >antibacterial07488... at 47.06%
40    21aa, >antibacterial10076... at 47.62%
41    12aa, >antibacterial10512... at 41.67%
42    30aa, >antibacterial10735... *
43    76aa, >antibacterial11083... at 44.74%
44    94aa, >antibacterial11085... at 50.00%
45    97aa, >antibacterial11086... at 49.48%
46    20aa, >antibacterial11888... at 35.00%
47    15aa, >antibacterial12008... at 35.00%
48    22aa, >antibacterial12010... at 40.91%
>Cluster 297
0     27aa, >Antifungal;antimicr... at 40.74%
1     37aa, >antimicrobial... at 40.54%
2     101aa, >Antimicrobial... *
3     30aa, >antibacterial11353... at 35.00%
4     101aa, >antibacterial13424... at 78.22%
>Cluster 298
0     100aa, >Antimicrobial... *
1     100aa, >Antimicrobial... at 86.00%
2     96aa, >Antimicrobial... at 53.12%
3     96aa, >Antimicrobial... at 58.33%
4     96aa, >Antimicrobial... at 56.25%
5     96aa, >Antimicrobial... at 56.25%
6     100aa, >Antimicrobial... at 82.00%
7     96aa, >Antimicrobial... at 84.38%
8     100aa, >Antimicrobial... at 69.00%
9     100aa, >Antimicrobial... at 69.00%
10    99aa, >Antimicrobial... at 80.00%
11    98aa, >Antimicrobial... at 82.00%
12    101aa, >Antimicrobial... at 69.00%
13    102aa, >Antimicrobial... at 83.00%
14    100aa, >Antimicrobial... at 69.00%
15    78aa, >Antimicrobial... at 85.00%
16    80aa, >Antimicrobial... at 80.00%
17    103aa, >Antimicrobial... *
18    96aa, >Antimicrobial... at 81.00%
19    105aa, >Antimicrobial... at 83.00%
20    40aa, >antimicrobial... at 67.50%
21    40aa, >antimicrobial... *
22    40aa, >antibacterial06583... at 55.00%
23    96aa, >antibacterial06807... at 55.21%
24    19aa, >antibacterial08547... *
25    28aa, >antibacterial09527... at 42.86%
>Cluster 299
0     26aa, >antimicrobial... at 42.31%
1     51aa, >Antimicrobial... at 62.16%
2     51aa, >Antimicrobial... *
```

```
3     51aa, >Antimicrobial... at 60.78%
4     51aa, >Antibacterial;Antim... at 66.67%
5     51aa, >Antimicrobial... at 66.67%
6     43aa, >Antimicrobial... at 55.81%
7     35aa, >Antimicrobial;Antim... at 42.86%
8     78aa, >Antimicrobial... at 83.59%
9     78aa, >Antimicrobial... *
10    78aa, >Antimicrobial... at 84.62%
11    79aa, >Antimicrobial... at 60.76%
12    63aa, >Antimicrobial... at 60.32%
13    41aa, >Antimicrobial... at 41.46%
14    100aa, >Antimicrobial... *
15    78aa, >Antimicrobial... at 75.64%
16    42aa, >Antimicrobial... at 42.86%
17    34aa, >Antimicrobial... at 76.47%
18    85aa, >antibacterial06861... at 58.82%
19    63aa, >antibacterial07143... at 53.97%
20    35aa, >antibacterial07487... at 35.00%
21    79aa, >antibacterial11092... at 64.56%
>Cluster 300
0     99aa, >anti-Gram-;Antibact... *
1     95aa, >anti-Gram+;Gram-... at 42.11%
2     23aa, >Antimicrobial... at 43.48%
3     80aa, >anti-Gram-;Antibact... at 46.25%
4     16aa, >antibacterial08866... at 43.75%
5     98aa, >antibacterial10045... at 40.82%
6     24aa, >antibacterial10366... *
>Cluster 301
0     62aa, >Antibacterial;Antim... at 45.16%
1     62aa, >Antibacterial... at 51.61%
2     99aa, >Antimicrobial... *
3     66aa, >Antimicrobial... at 59.09%
4     64aa, >Antimicrobial... at 64.06%
5     65aa, >Antimicrobial... at 58.46%
6     66aa, >Antimicrobial... at 62.12%
7     56aa, >Antimicrobial... at 67.86%
8     66aa, >Antimicrobial... at 59.09%
9     66aa, >Antimicrobial... at 56.06%
10    58aa, >Antimicrobial... *
11    77aa, >Antimicrobial... at 54.55%
12    82aa, >antibacterial06830... at 43.90%
13    18aa, >antibacterial13002... at 44.44%
>Cluster 302
0     99aa, >anti-Gram+;Gram-... *
>Cluster 303
0     47aa, >Antimicrobial... at 61.70%
1     45aa, >Antimicrobial... at 80.00%
2     40aa, >Antimicrobial... at 35.00%
3     84aa, >Antimicrobial... at 58.33%
4     69aa, >Antimicrobial... *
5     99aa, >Antimicrobial... *
6     83aa, >Antimicrobial... at 44.58%
7     67aa, >Antimicrobial... at 59.70%
8     67aa, >Antimicrobial... at 61.19%
```

9   83aa, >Antimicrobial... at 49.40%
10   89aa, >Antimicrobial... at 47.19%
11   69aa, >Antimicrobial... at 52.32%
12   63aa, >Antimicrobial... at 60.32%
13   20aa, >antimicrobial... at 35.00%
14   37aa, >Antimicrobial... at 59.46%
15   48aa, >Antimicrobial... at 60.42%
16   40aa, >Antimicrobial... at 37.50%
17   47aa, >Antimicrobial... at 70.21%
18   48aa, >Antimicrobial... at 64.58%
19   45aa, >Antimicrobial... *
20   67aa, >antibacterial06874... at 53.73%
21   18aa, >antibacterial09719... at 34.44%
22   67aa, >antibacterial11009... at 53.73%
23   98aa, >antibacterial11013... at 68.37%
24   66aa, >antibacterial11018... at 56.06%
25   69aa, >antibacterial11019... at 62.32%
26   69aa, >antibacterial11020... at 68.12%
>Cluster 304
0   21aa, >Antimicrobial... at 52.38%
1   42aa, >Antibacterial... *
2   50aa, >Antimicrobial... at 50.00%
3   82aa, >Antimicrobial... at 47.56%
4   82aa, >Antimicrobial... at 31.46%
5   85aa, >Antimicrobial... at 30.00%
6   83aa, >Antimicrobial... at 30.96%
7   86aa, >Antimicrobial... at 41.86%
8   87aa, >Antimicrobial... at 41.38%
9   89aa, >Antimicrobial... at 43.82%
10   90aa, >Antimicrobial... at 35.00%
11   21aa, >Antimicrobial... at 47.62%
12   86aa, >Antimicrobial... at 40.70%
13   65aa, >Antimicrobial... at 35.00%
14   99aa, >Antibacterial... *
15   42aa, >Antimicrobial... at 32.86%
16   59aa, >Antimicrobial... at 34.07%
17   84aa, >antibacterial06741... at 30.48%
18   29aa, >antibacterial08554... at 41.38%
19   16aa, >antibacterial09944... at 56.25%
20   29aa, >antibacterial10371... at 41.38%
21   81aa, >antibacterial11014... at 40.74%
>Cluster 305
0   33aa, >antimicrobial... at 42.42%
1   33aa, >Antimicrobial... at 42.42%
2   99aa, >Antimicrobial... *
3   89aa, >Antimicrobial... at 57.30%
4   90aa, >Antimicrobial... at 50.00%
5   24aa, >antibacterial09473... at 45.83%
6   31aa, >antibacterial10606... at 41.94%
>Cluster 306
0   99aa, >Antibacterial... *
1   13aa, >antibacterial13011... at 53.85%
2   26aa, >antibacterial13033... at 46.15%
>Cluster 307

```
0     66aa, >Antimicrobial... *
1     66aa, >Antimicrobial... at 80.30%
2     66aa, >Antimicrobial... at 68.18%
3     66aa, >Antimicrobial... at 51.52%
4     67aa, >Antimicrobial... at 40.30%
5     66aa, >Antimicrobial... at 42.42%
6     67aa, >Antimicrobial... at 59.70%
7     75aa, >antibacterial06514... at 61.33%
8     99aa, >antibacterial06588... *
9     97aa, >antibacterial06708... at 46.39%
10    84aa, >antibacterial06719... at 57.14%
11    77aa, >antibacterial06792... at 61.04%
12    70aa, >antibacterial07321... at 42.86%
13    78aa, >antibacterial07496... at 61.54%
14    20aa, >antibacterial07677... at 45.00%
15    18aa, >antibacterial07908... at 50.00%
16    20aa, >antibacterial09113... at 35.00%
17    25aa, >antibacterial12798... at 35.00%
>Cluster 308
0     37aa, >Antimicrobial... at 40.54%
1     41aa, >Antibacterial;Antif... at 41.46%
2     64aa, >Antimicrobial... at 46.88%
3     66aa, >anti-Gram+;antifung... *
4     79aa, >anti-Gram+;Gram-... *
5     66aa, >Antimicrobial... at 45.45%
6     66aa, >anti-Gram+;antifung... at 40.91%
7     66aa, >Antimicrobial... at 71.21%
8     74aa, >antibacterial06441... at 60.81%
9     76aa, >antibacterial06448... at 68.42%
10    36aa, >antibacterial06506... at 41.67%
11    66aa, >antibacterial06573... at 81.82%
12    62aa, >antibacterial06628... at 51.61%
13    76aa, >antibacterial06703... at 73.68%
14    96aa, >antibacterial06721... at 46.88%
15    94aa, >antibacterial06722... at 53.19%
16    99aa, >antibacterial06740... *
17    98aa, >antibacterial06839... at 40.82%
18    88aa, >antibacterial06844... at 43.18%
19    93aa, >antibacterial06848... at 46.24%
20    95aa, >antibacterial06850... at 46.32%
21    90aa, >antibacterial06858... at 35.00%
22    90aa, >antibacterial06912... at 68.89%
23    59aa, >antibacterial06951... at 42.37%
24    75aa, >antibacterial07083... at 73.33%
25    94aa, >antibacterial07106... at 47.87%
26    54aa, >antibacterial07253... at 72.22%
27    80aa, >antibacterial07592... at 55.00%
>Cluster 309
0     99aa, >antibacterial10741... *
1     40aa, >antibacterial11938... at 35.00%
>Cluster 310
0     79aa, >anti-Gram+;Gram-... at 59.49%
1     99aa, >antibacterial10903... *
>Cluster 311
```

0   98aa, >antibacterial... *
1   23aa, >antibacterial08215... at 43.48%
2   41aa, >antibacterial08459... at 41.46%
>Cluster 312
0   98aa, >Antimicrobial... *
1   77aa, >anti-Gram+;Antibact... at 82.21%
2   16aa, >antibacterial08413... at 43.75%
>Cluster 313
0   56aa, >Antimicrobial... at 44.64%
1   54aa, >Antimicrobial... at 51.85%
2   35aa, >Antimicrobial... *
3   41aa, >Antimicrobial... at 48.78%
4   95aa, >Antimicrobial... at 47.37%
5   89aa, >Antimicrobial... at 52.81%
6   90aa, >Antimicrobial... at 60.00%
7   90aa, >Antimicrobial... at 64.44%
8   90aa, >Antimicrobial... at 63.33%
9   90aa, >Antimicrobial... at 56.67%
10   90aa, >Antimicrobial... at 62.22%
11   90aa, >Antimicrobial... at 64.44%
12   98aa, >Antimicrobial... *
13   85aa, >Antimicrobial... at 45.88%
14   90aa, >Antimicrobial... at 62.22%
15   88aa, >Antimicrobial... at 60.23%
16   88aa, >Antimicrobial... at 61.36%
17   90aa, >Antimicrobial... at 57.78%
18   93aa, >Antimicrobial... at 47.31%
19   89aa, >Antimicrobial... at 40.45%
20   89aa, >Antimicrobial... at 42.70%
21   83aa, >Antimicrobial... at 44.58%
22   84aa, >Antimicrobial... at 41.67%
23   85aa, >Antimicrobial... at 48.24%
24   92aa, >Antimicrobial... at 46.74%
25   88aa, >Antimicrobial... at 40.91%
26   87aa, >Antimicrobial... at 42.53%
27   86aa, >Antimicrobial... at 43.02%
28   86aa, >Antimicrobial... at 41.86%
29   93aa, >Antimicrobial... at 40.86%
30   88aa, >Antimicrobial... at 45.45%
31   86aa, >Antimicrobial... at 44.19%
32   95aa, >Antimicrobial... at 35.00%
33   88aa, >Antimicrobial... at 43.18%
34   32aa, >Antimicrobial... at 50.00%
35   86aa, >Antimicrobial... at 45.35%
36   90aa, >Antimicrobial... at 51.11%
37   96aa, >Antimicrobial... at 55.21%
38   88aa, >Antimicrobial... *
39   93aa, >Antimicrobial... at 46.24%
40   52aa, >Antimicrobial... at 59.62%
41   61aa, >antibacterial06692... at 49.18%
42   85aa, >antibacterial06879... at 41.18%
43   96aa, >antibacterial07190... at 45.83%
44   87aa, >antibacterial09845... at 41.38%
45   81aa, >antibacterial11021... at 43.21%

46   85aa, >antibacterial11022... at 44.71%
47   88aa, >antibacterial11024... at 43.18%
48   95aa, >antibacterial11025... at 41.05%
49   88aa, >antibacterial11027... at 44.32%
50   88aa, >antibacterial11028... at 42.05%
51   84aa, >antibacterial11029... at 40.48%
52   84aa, >antibacterial11030... at 42.86%
53   84aa, >antibacterial11031... at 46.43%
>Cluster 314
0   98aa, >Antibacterial... *
1   13aa, >antibacterial06596... at 46.15%
2   24aa, >antibacterial06977... at 45.83%
>Cluster 315
0   24aa, >antimicrobial... at 41.67%
1   98aa, >anti-Gram+;Antibact... *
2   11aa, >antibacterial09818... at 45.45%
>Cluster 316
0   33aa, >antimicrobial... at 42.42%
1   34aa, >antibacterial... at 41.18%
2   77aa, >anti-Gram+;Antibact... at 46.75%
3   98aa, >antibacterial06805... *
4   96aa, >antibacterial07155... at 60.42%
5   96aa, >antibacterial07156... at 62.50%
>Cluster 317
0   35aa, >antimicrobial... at 35.00%
1   38aa, >anti-Gram+;antibact... at 42.11%
2   38aa, >antimicrobial... at 42.11%
3   38aa, >antimicrobial... at 42.11%
4   38aa, >antibacterial;antim... at 44.74%
5   36aa, >antimicrobial... at 41.67%
6   37aa, >antimicrobial... at 40.54%
7   38aa, >antimicrobial... at 42.11%
8   38aa, >antibacterial;antim... at 44.74%
9   34aa, >anti-Gram+;antimicr... at 44.12%
10   42aa, >antimicrobial... at 47.62%
11   34aa, >anti-Gram+;antimicr... at 50.00%
12   44aa, >Antibacterial;antim... at 40.91%
13   42aa, >antimicrobial... at 42.86%
14   41aa, >antifungal;antimicr... at 51.22%
15   94aa, >antibacterial06808... at 42.55%
16   94aa, >antibacterial06809... at 40.43%
17   83aa, >antibacterial06810... at 48.19%
18   98aa, >antibacterial06867... *
19   98aa, >antibacterial06903... at 50.00%
20   46aa, >antibacterial07541... *
21   18aa, >antibacterial07948... at 44.44%
>Cluster 318
0   24aa, >antimicrobial... at 41.67%
1   24aa, >antibacterial;antif... at 41.67%
2   23aa, >antimicrobial... at 52.17%
3   20aa, >antimicrobial... at 35.00%
4   66aa, >Antimicrobial... at 40.91%
5   96aa, >antibacterial06804... at 45.83%
6   88aa, >antibacterial06845... at 43.18%

7   88aa, >antibacterial06890... at 45.45%
8   90aa, >antibacterial07109... at 45.56%
9   88aa, >antibacterial07114... at 45.45%
10   98aa, >antibacterial07222... *
11   50aa, >antibacterial07266... at 35.00%
12   63aa, >antibacterial10943... at 44.44%
13   26aa, >antibacterial11396... at 42.31%
14   25aa, >antibacterial11415... at 44.00%
>Cluster 319
0   97aa, >Antiviral... *
1   20aa, >antibacterial11322... at 35.00%
2   13aa, >antibacterial12246... at 46.15%
>Cluster 320
0   59aa, >anti-Gram+;Antibact... at 44.07%
1   41aa, >antimicrobial... at 41.46%
2   97aa, >Antibacterial... *
3   76aa, >Antimicrobial... at 42.11%
>Cluster 321
0   33aa, >anti-Gram+;antibact... at 42.42%
1   97aa, >Antimicrobial... *
2   33aa, >antimicrobial... at 42.42%
>Cluster 322
0   61aa, >Antimicrobial... at 85.25%
1   97aa, >Antimicrobial... *
2   58aa, >Antimicrobial... at 50.00%
3   52aa, >Antimicrobial... at 51.92%
4   13aa, >antibacterial11654... at 46.15%
>Cluster 323
0   97aa, >Antimicrobial... *
1   20aa, >antibacterial08062... at 45.00%
>Cluster 324
0   97aa, >anti-Gram+;Antibact... *
1   97aa, >anti-Gram+;Gram-... at 64.95%
2   97aa, >Antibacterial... at 63.92%
3   26aa, >antibacterial09201... at 42.31%
4   66aa, >antibacterial09738... at 53.03%
5   24aa, >antibacterial10044... at 41.67%
6   14aa, >antibacterial10642... at 42.86%
7   11aa, >antibacterial10662... at 54.55%
8   80aa, >antibacterial11073... at 67.50%
9   13aa, >antibacterial12050... at 46.15%
10   13aa, >antibacterial12057... *
>Cluster 325
0   18aa, >Antimicrobial... at 44.44%
1   94aa, >antibacterial06733... at 43.62%
2   97aa, >antibacterial06748... *
3   93aa, >antibacterial06751... at 47.31%
4   95aa, >antibacterial06752... at 44.21%
5   86aa, >antibacterial07234... at 43.02%
6   87aa, >antibacterial07298... at 41.38%
7   20aa, >antibacterial07634... at 35.00%
>Cluster 326
0   29aa, >anti-Gram+;antifung... at 44.83%
1   50aa, >antibacterial06470... at 50.00%

2 13aa, >antibacterial06605... at 46.15%
3 46aa, >antibacterial07051... at 52.17%
4 46aa, >antibacterial07052... at 50.00%
5 46aa, >antibacterial07075... at 43.48%
6 46aa, >antibacterial07079... at 45.65%
7 46aa, >antibacterial07282... at 43.48%
8 97aa, >antibacterial07542... *
9 71aa, >antibacterial07618... at 42.25%
10 24aa, >antibacterial08131... at 41.67%
11 29aa, >antibacterial09884... at 41.38%
12 18aa, >antibacterial10339... at 44.44%
13 13aa, >antibacterial10428... at 53.85%
14 18aa, >antibacterial10543... *
15 27aa, >antibacterial13147... at 44.44%
>Cluster 327
0 75aa, >Antibacterial... at 79.00%
1 97aa, >antibacterial10809... *
2 28aa, >antibacterial13003... at 42.86%
>Cluster 328
0 30aa, >antibacterial08495... at 35.00%
1 30aa, >antibacterial08496... at 35.00%
2 97aa, >antibacterial10899... *
3 21aa, >antibacterial12834... at 42.86%
>Cluster 329
0 78aa, >Antimicrobial... at 55.13%
1 52aa, >Antimicrobial... at 50.00%
2 36aa, >antimicrobial... at 41.67%
3 55aa, >Antibacterial;Antim... at 54.55%
4 80aa, >Antimicrobial... at 46.25%
5 73aa, >Antimicrobial... at 53.42%
6 79aa, >Antimicrobial... at 50.63%
7 67aa, >Antimicrobial... at 58.21%
8 80aa, >Antimicrobial... at 50.00%
9 94aa, >Antimicrobial... at 50.00%
10 96aa, >Antimicrobial... *
11 78aa, >Antimicrobial... at 55.13%
12 36aa, >antibacterial09012... at 41.67%
>Cluster 330
0 96aa, >Anti-Gram-... *
>Cluster 331
0 31aa, >antimicrobial... at 41.94%
1 96aa, >Antimicrobial... *
>Cluster 332
0 96aa, >anti-Gram+;Antibact... *
1 15aa, >antibacterial10441... at 35.00%
>Cluster 333
0 73aa, >Antimicrobial... at 42.47%
1 96aa, >antibacterial06880... *
2 84aa, >antibacterial07593... at 51.19%
>Cluster 334
0 96aa, >antibacterial07220... *
1 23aa, >antibacterial07655... at 43.48%
>Cluster 335
0 26aa, >Antibacterial;Antif... at 42.31%

1   93aa, >antibacterial06725... at 40.86%
2   96aa, >antibacterial07241... *
3   95aa, >antibacterial07248... at 80.00%
4   73aa, >antibacterial07250... at 45.21%
>Cluster 336
0   95aa, >anti-HIV;antiviral... *
>Cluster 337
0   95aa, >Antimicrobial... *
1   74aa, >Antimicrobial... at 47.30%
>Cluster 338
0   95aa, >Antimicrobial... *
1   12aa, >antibacterial08969... at 41.67%
2   12aa, >antibacterial08971... at 41.67%
>Cluster 339
0   95aa, >Antimicrobial... *
>Cluster 340
0   13aa, >antimicrobial... at 53.85%
1   32aa, >antimicrobial... at 43.75%
2   91aa, >antibacterial06476... at 61.54%
3   91aa, >antibacterial06564... *
4   92aa, >antibacterial06757... at 51.09%
5   92aa, >antibacterial06759... at 61.96%
6   92aa, >antibacterial06760... at 59.78%
7   91aa, >antibacterial06761... at 63.74%
8   88aa, >antibacterial06762... at 60.23%
9   92aa, >antibacterial06763... at 55.43%
10   78aa, >antibacterial06764... at 44.87%
11   92aa, >antibacterial06796... at 63.04%
12   95aa, >antibacterial06797... *
13   94aa, >antibacterial06799... at 46.81%
14   92aa, >antibacterial07144... at 55.43%
15   93aa, >antibacterial07145... at 59.14%
16   83aa, >antibacterial07225... at 43.37%
17   87aa, >antibacterial07227... at 50.57%
18   92aa, >antibacterial07228... at 56.52%
19   93aa, >antibacterial07229... at 51.61%
20   94aa, >antibacterial07230... at 50.00%
21   91aa, >antibacterial07301... at 57.14%
22   90aa, >antibacterial07302... at 63.33%
23   92aa, >antibacterial07508... at 51.09%
24   91aa, >antibacterial07509... at 60.44%
25   91aa, >antibacterial07510... at 56.04%
26   91aa, >antibacterial07511... at 57.14%
27   91aa, >antibacterial07512... at 54.95%
28   91aa, >antibacterial07513... at 59.34%
29   14aa, >antibacterial09919... at 42.86%
30   23aa, >antibacterial11646... at 43.48%
>Cluster 341
0   76aa, >Antimicrobial... at 84.21%
1   76aa, >Antimicrobial... at 89.47%
2   51aa, >Antimicrobial... at 41.18%
3   19aa, >anti-Gram+;antibact... at 52.63%
4   67aa, >Antimicrobial... at 43.28%
5   67aa, >Antimicrobial... at 40.30%

```
6     95aa, >antibacterial06816... *
7     33aa, >antibacterial07543... at 42.42%
8     29aa, >antibacterial10263... at 41.38%
>Cluster 342
0     38aa, >antifungal;antimicr... at 42.11%
1     38aa, >antifungal... at 44.74%
2     49aa, >anti-Gram+;antibact... at 42.86%
3     42aa, >anti-Gram+;antibact... at 40.48%
4     37aa, >anti-Gram+;Antibact... *
5     38aa, >anti-Gram+;antibact... at 37.89%
6     39aa, >antimicrobial... at 46.15%
7     39aa, >antibacterial;antim... at 48.72%
8     38aa, >antimicrobial... at 33.16%
9     39aa, >antimicrobial... at 41.03%
10    39aa, >antimicrobial... at 36.15%
11    37aa, >antimicrobial... at 40.54%
12    73aa, >antibacterial07125... at 42.47%
13    73aa, >antibacterial07126... at 42.47%
14    40aa, >antibacterial08370... at 82.50%
15    40aa, >antibacterial08792... *
16    40aa, >antibacterial08794... at 80.00%
17    11aa, >antibacterial09466... at 45.45%
18    17aa, >antibacterial10179... at 41.18%
19    40aa, >antibacterial10274... at 72.50%
20    37aa, >antibacterial10378... at 67.57%
21    40aa, >antibacterial10480... at 35.00%
22    55aa, >antibacterial10681... at 35.00%
23    36aa, >antibacterial10772... at 55.56%
24    95aa, >antibacterial10821... *
25    42aa, >antibacterial12388... at 72.86%
>Cluster 343
0     37aa, >anti-Gram+;Antibact... at 40.54%
1     32aa, >antimicrobial... at 40.62%
2     68aa, >Antibacterial... at 42.65%
3     76aa, >Antiviral... at 72.37%
4     76aa, >Antibacterial;Antif... *
5     94aa, >antibacterial07133... at 73.40%
6     21aa, >antibacterial09715... at 47.62%
7     25aa, >antibacterial10306... at 35.00%
8     95aa, >antibacterial11058... *
9     95aa, >antibacterial11063... at 78.95%
10    91aa, >antibacterial11069... at 40.66%
>Cluster 344
0     91aa, >Antibacterial;Antif... at 49.45%
1     90aa, >Antimicrobial... at 48.89%
2     93aa, >Antibacterial... at 49.46%
3     93aa, >Antimicrobial... at 50.54%
4     90aa, >Antimicrobial... at 43.33%
5     93aa, >Antimicrobial... at 49.46%
6     90aa, >Antibacterial;Antif... at 52.22%
7     90aa, >Antimicrobial... at 54.44%
8     90aa, >Antimicrobial... at 45.56%
9     93aa, >Antibacterial... at 45.16%
10    94aa, >Antimicrobial... *
```

```
11    92aa, >Antimicrobial... at 48.91%
12    93aa, >Antimicrobial... at 48.39%
13    90aa, >Antibacterial;Antif... at 43.33%
14    90aa, >Antimicrobial... at 44.44%
15    92aa, >Antimicrobial... at 47.83%
16    91aa, >Antimicrobial... at 53.85%
17    93aa, >Antibacterial... at 40.86%
18    94aa, >Antimicrobial... at 40.43%
19    93aa, >Antimicrobial... at 48.39%
20    90aa, >Antifungal;Antimicr... at 47.78%
21    91aa, >Antimicrobial... at 51.65%
22    91aa, >Antifungal... at 48.35%
23    92aa, >Antimicrobial... at 50.00%
24    32aa, >Antifungal... at 43.75%
25    23aa, >Antifungal... *
26    91aa, >Antimicrobial... at 42.86%
27    13aa, >antimicrobial... *
28    93aa, >Antimicrobial... at 43.01%
29    17aa, >antibacterial07398... at 41.18%
30    25aa, >antibacterial07623... at 35.00%
>Cluster 345
0     25aa, >anti-Gram+;Gram-... at 35.00%
1     34aa, >anti-Gram+;antibact... at 41.18%
2     94aa, >Antibacterial... *
3     27aa, >antibacterial;antif... at 40.74%
4     14aa, >antibacterial08916... at 42.86%
5     18aa, >antibacterial09326... at 44.44%
6     18aa, >antibacterial09709... at 44.44%
7     13aa, >antibacterial12060... at 46.15%
>Cluster 346
0     36aa, >Antibacterial;Antim... at 44.44%
1     79aa, >Antimicrobial... at 56.96%
2     82aa, >Antimicrobial... at 56.10%
3     80aa, >Antimicrobial... at 61.25%
4     63aa, >Antimicrobial... at 61.90%
5     82aa, >Antimicrobial... at 54.88%
6     81aa, >Antimicrobial... at 53.09%
7     81aa, >Antimicrobial... at 55.56%
8     68aa, >Antimicrobial... at 61.76%
9     38aa, >Antimicrobial... at 44.74%
10    58aa, >Antimicrobial... at 62.07%
11    91aa, >Antimicrobial... at 52.75%
12    94aa, >Antimicrobial... *
13    81aa, >Antimicrobial... at 56.79%
14    23aa, >antibacterial08097... at 43.48%
15    20aa, >antibacterial11596... at 35.00%
>Cluster 347
0     47aa, >anti-Gram+;antibact... *
1     33aa, >Antimicrobial... at 42.42%
2     94aa, >Antimicrobial... *
3     94aa, >Antimicrobial... at 85.11%
4     91aa, >Antimicrobial... at 43.96%
5     33aa, >anti-Gram+;antibact... at 42.42%
>Cluster 348
```

```
0     27aa, >antibacterial;antim... at 44.44%
1     36aa, >antimicrobial... at 44.44%
2     94aa, >Antimicrobial... *
3     94aa, >Antimicrobial... at 63.83%
4     94aa, >Antimicrobial... at 61.70%
5     94aa, >Antimicrobial... at 53.19%
6     39aa, >antimicrobial... at 51.28%
7     24aa, >antibacterial10365... at 41.67%
>Cluster 349
0     94aa, >Antibacterial... *
>Cluster 350
0     55aa, >Antifungal... at 43.64%
1     33aa, >Antimicrobial... at 42.42%
2     21aa, >Antibacterial... at 42.86%
3     57aa, >antifungal... at 45.61%
4     55aa, >Antifungal... at 35.00%
5     50aa, >antimicrobial... at 35.00%
6     94aa, >antibacterial06826... *
7     55aa, >antibacterial08467... at 43.64%
8     42aa, >antibacterial10848... at 40.48%
>Cluster 351
0     18aa, >antibacterial;antim... at 44.44%
1     94aa, >antibacterial07191... *
2     23aa, >antibacterial07960... at 47.83%
3     35aa, >antibacterial08400... at 35.00%
4     46aa, >antibacterial10833... at 83.48%
>Cluster 352
0     94aa, >antibacterial07294... *
1     18aa, >antibacterial10129... at 50.00%
2     29aa, >antibacterial11636... at 41.38%
>Cluster 353
0     24aa, >antimicrobial... at 41.67%
1     94aa, >antibacterial10807... *
2     86aa, >antibacterial10808... at 67.44%
3     87aa, >antibacterial10902... at 66.67%
>Cluster 354
0     48aa, >Antimicrobial... at 45.83%
1     53aa, >Antimicrobial... at 43.40%
2     66aa, >Antimicrobial... at 54.55%
3     75aa, >Antimicrobial... *
4     78aa, >Antimicrobial... at 52.56%
5     66aa, >Antimicrobial... at 57.58%
6     71aa, >Antimicrobial... at 57.75%
7     81aa, >Antimicrobial... at 50.62%
8     81aa, >Antimicrobial... at 50.62%
9     76aa, >Antimicrobial... at 55.26%
10    79aa, >Antimicrobial... at 45.57%
11    73aa, >antibacterial06710... at 52.05%
12    77aa, >antibacterial06711... at 51.95%
13    77aa, >antibacterial06712... at 51.95%
14    72aa, >antibacterial06713... at 56.94%
15    72aa, >antibacterial06715... at 58.33%
16    75aa, >antibacterial06745... at 52.00%
17    71aa, >antibacterial07087... at 54.93%
```

```
18    66aa, >antibacterial07221... at 51.52%
19    64aa, >antibacterial07240... at 57.81%
20    66aa, >antibacterial10804... at 59.09%
21    57aa, >antibacterial10966... at 59.65%
22    66aa, >antibacterial10970... at 56.06%
23    81aa, >antibacterial10971... at 54.32%
24    77aa, >antibacterial10973... at 55.84%
25    73aa, >antibacterial10975... at 57.53%
26    94aa, >antibacterial10980... *
27    91aa, >antibacterial10981... at 72.53%
28    67aa, >antibacterial10982... at 67.16%
>Cluster 355
0     93aa, >Antifungal;Antimicr... *
1     33aa, >antimicrobial... at 45.45%
2     33aa, >antimicrobial... at 45.45%
3     33aa, >antimicrobial... at 42.42%
4     90aa, >Antifungal... at 35.00%
5     91aa, >Antibacterial;Antif... at 40.66%
6     92aa, >Antimicrobial... at 46.74%
7     13aa, >antibacterial08030... at 46.15%
>Cluster 356
0     93aa, >Antibacterial... *
1     87aa, >Antibacterial... at 41.38%
>Cluster 357
0     28aa, >Antibacterial... at 57.14%
1     30aa, >antimicrobial... at 43.33%
2     32aa, >antimicrobial... at 56.25%
3     31aa, >antimicrobial... *
4     33aa, >antimicrobial... at 45.45%
5     31aa, >anti-HIV;Antimicrob... at 41.94%
6     31aa, >Antibacterial;Antim... at 61.29%
7     28aa, >anti-HIV;antimicrob... at 42.86%
8     31aa, >Antimicrobial... at 58.06%
9     31aa, >antimicrobial... at 61.29%
10    93aa, >Antimicrobial... *
11    31aa, >antibacterial06485... at 58.06%
>Cluster 358
0     93aa, >antifungal... *
>Cluster 359
0     38aa, >antimicrobial... at 42.11%
1     38aa, >antimicrobial... at 42.11%
2     93aa, >Antimicrobial... *
3     32aa, >antibacterial07420... at 40.62%
>Cluster 360
0     93aa, >Antimicrobial... *
1     90aa, >Antimicrobial... at 54.44%
>Cluster 361
0     13aa, >antimicrobial... at 46.15%
1     36aa, >antibacterial;antim... at 44.44%
2     93aa, >Antibacterial;Antif... *
3     46aa, >antimicrobial... at 41.30%
4     13aa, >antibacterial06598... at 46.15%
5     29aa, >antibacterial07359... at 44.83%
6     29aa, >antibacterial07360... at 44.83%
```

>Cluster 362
0   28aa, >antimicrobial... at 42.86%
1   21aa, >Antifungal... at 42.86%
2   77aa, >Antibacterial... at 49.35%
3   76aa, >Antibacterial... at 55.26%
4   77aa, >anti-Gram+;Antibact... at 42.86%
5   29aa, >antibacterial;Antif... at 44.83%
6   33aa, >antibacterial... at 45.45%
7   34aa, >antibacterial... at 55.88%
8   23aa, >antibacterial06452... at 43.48%
9   93aa, >antibacterial06793... *
10   86aa, >antibacterial07129... at 83.72%
11   74aa, >antibacterial07449... at 44.59%
12   77aa, >antibacterial10399... at 44.16%
13   70aa, >antibacterial10761... at 52.86%
>Cluster 363
0   75aa, >Antibacterial... at 83.33%
1   75aa, >Antibacterial;Antim... *
2   75aa, >Antimicrobial... at 82.67%
3   46aa, >Antimicrobial... at 81.30%
4   76aa, >Antimicrobial... at 44.74%
5   76aa, >Antimicrobial... at 50.00%
6   77aa, >Antimicrobial... at 51.95%
7   76aa, >Antimicrobial... at 52.63%
8   28aa, >antibacterial;Antif... at 42.86%
9   75aa, >Antimicrobial... at 54.67%
10   75aa, >Antimicrobial... at 46.67%
11   75aa, >Antibacterial;Antim... at 45.33%
12   75aa, >Antibacterial... at 49.33%
13   88aa, >Antimicrobial... at 85.23%
14   79aa, >Antimicrobial... at 48.10%
15   75aa, >Antimicrobial... at 52.00%
16   73aa, >Antimicrobial... at 57.53%
17   75aa, >Antimicrobial... at 44.00%
18   77aa, >Antimicrobial... at 54.55%
19   76aa, >Antimicrobial... at 52.63%
20   93aa, >antibacterial06846... *
>Cluster 364
0   37aa, >Antimicrobial... at 40.54%
1   34aa, >anti-Gram+;Antibact... at 41.18%
2   60aa, >Antibacterial... *
3   60aa, >Antibacterial... at 56.67%
4   67aa, >Antibacterial... at 41.79%
5   89aa, >Antibacterial... at 40.45%
6   71aa, >Antibacterial... at 42.25%
7   76aa, >Antibacterial... at 53.95%
8   38aa, >anti-Gram+;antifung... at 42.11%
9   43aa, >antifungal;antimicr... at 41.86%
10   85aa, >antibacterial06791... at 56.47%
11   93aa, >antibacterial06873... *
12   39aa, >antibacterial08785... at 46.15%
13   26aa, >antibacterial11425... at 42.31%
14   38aa, >antibacterial11792... at 42.11%
>Cluster 365

```
0     20aa, >antibacterial08544... at 35.00%
1     93aa, >antibacterial10859... *
2     25aa, >antibacterial11451... at 35.00%
>Cluster 366
0     20aa, >antibacterial06684... at 35.00%
1     20aa, >antibacterial07485... at 35.00%
2     21aa, >antibacterial09523... at 42.86%
3     84aa, >antibacterial10819... at 54.76%
4     88aa, >antibacterial10820... at 45.45%
5     82aa, >antibacterial11036... at 53.66%
6     93aa, >antibacterial11039... *
7     36aa, >antibacterial11544... at 41.67%
8     37aa, >antibacterial13231... at 43.24%
>Cluster 367
0     11aa, >antibacterial08025... at 45.45%
1     19aa, >antibacterial09622... at 47.37%
2     93aa, >antibacterial11041... *
>Cluster 368
0     35aa, >Antimicrobial... at 35.00%
1     27aa, >antibacterial;antif... at 40.74%
2     87aa, >antibacterial07214... at 41.38%
3     93aa, >antibacterial11088... *
>Cluster 369
0     92aa, >Antimicrobial... *
>Cluster 370
0     45aa, >Antifungal... at 42.22%
1     42aa, >antimicrobial... at 40.48%
2     52aa, >anti-Gram+;antibact... at 46.15%
3     48aa, >antifungal;antimicr... *
4     29aa, >Antimicrobial... at 41.38%
5     47aa, >Antimicrobial... at 42.55%
6     48aa, >antibacterial... at 47.92%
7     47aa, >antifungal;antimicr... at 44.68%
8     47aa, >Antimicrobial... at 46.81%
9     47aa, >Antimicrobial... at 40.43%
10    47aa, >anti-Gram+;antibact... at 42.55%
11    50aa, >Antimicrobial... at 35.00%
12    77aa, >Antimicrobial... at 63.64%
13    78aa, >Antimicrobial... *
14    92aa, >Antimicrobial... *
15    76aa, >Antimicrobial... at 46.05%
16    51aa, >Antifungal;antimicr... at 45.10%
17    48aa, >anti-Gram+;antibact... at 64.58%
18    49aa, >antimicrobial... at 51.02%
19    47aa, >antimicrobial... at 44.68%
20    46aa, >Antimicrobial... at 54.35%
21    12aa, >anti-Gram+;antibact... at 41.67%
22    74aa, >antibacterial06717... at 52.16%
23    75aa, >antibacterial06718... at 44.00%
24    80aa, >antibacterial06754... at 51.25%
25    77aa, >antibacterial06776... *
26    74aa, >antibacterial06779... at 47.30%
27    73aa, >antibacterial06780... at 46.58%
28    77aa, >antibacterial06820... at 57.14%
```

```
29    80aa, >antibacterial06852... at 43.75%
30    73aa, >antibacterial06853... at 52.05%
31    77aa, >antibacterial06854... at 61.04%
32    77aa, >antibacterial06856... at 58.44%
33    78aa, >antibacterial06869... at 58.97%
34    76aa, >antibacterial06895... at 53.95%
35    47aa, >antibacterial07364... at 51.06%
36    49aa, >antibacterial07418... at 48.98%
37    22aa, >antibacterial07577... at 59.09%
38    24aa, >antibacterial08333... at 41.67%
39    46aa, >antibacterial10574... at 50.00%
40    24aa, >antibacterial11881... at 41.67%
>Cluster 371
0     92aa, >Antimicrobial... *
>Cluster 372
0     30aa, >anti-Gram-;antibact... at 43.33%
1     16aa, >antibacterial... at 50.00%
2     92aa, >Gram-... *
3     36aa, >antibacterial10760... at 41.67%
4     19aa, >antibacterial13078... at 42.11%
>Cluster 373
0     24aa, >anti-Gram+;Antibact... at 41.67%
1     29aa, >antibacterial;antif... at 41.38%
2     30aa, >antimicrobial;antiv... at 35.00%
3     24aa, >Antibacterial;Antif... at 45.83%
4     92aa, >Antimicrobial... *
>Cluster 374
0     37aa, >anti-Gram+;antibact... at 40.54%
1     70aa, >antibacterial06770... at 50.00%
2     64aa, >antibacterial06901... at 46.88%
3     72aa, >antibacterial06902... at 48.61%
4     77aa, >antibacterial06931... at 41.56%
5     75aa, >antibacterial07128... at 70.67%
6     71aa, >antibacterial07231... at 54.93%
7     92aa, >antibacterial07257... *
8     13aa, >antibacterial11745... at 46.15%
9     18aa, >antibacterial13095... at 44.44%
>Cluster 375
0     26aa, >antimicrobial... at 46.15%
1     58aa, >antibacterial08293... at 68.93%
2     59aa, >antibacterial08294... at 81.36%
3     58aa, >antibacterial09909... at 79.31%
4     92aa, >antibacterial11066... *
>Cluster 376
0     91aa, >Antifungal... *
1     24aa, >antibacterial;Antif... at 41.67%
2     47aa, >antibacterial07324... at 46.81%
>Cluster 377
0     24aa, >anti-Gram+;antibact... at 45.83%
1     24aa, >antibacterial;antif... at 41.67%
2     42aa, >Antibacterial... at 40.48%
3     65aa, >Antibacterial... *
4     65aa, >Antibacterial... at 82.31%
5     91aa, >Antimicrobial... *
```

6 87aa, >antibacterial07184... at 59.77%
7 38aa, >antibacterial07938... at 42.11%
8 39aa, >antibacterial08309... at 41.03%
>Cluster 378
0 26aa, >antibacterial;Antif... at 53.85%
1 63aa, >Antimicrobial... at 55.56%
2 21aa, >Antimicrobial... *
3 44aa, >antimicrobial... at 50.00%
4 46aa, >antimicrobial... at 47.83%
5 46aa, >antimicrobial... at 47.83%
6 46aa, >antimicrobial... at 45.65%
7 46aa, >anti-Gram+;antibact... at 43.48%
8 46aa, >anti-Gram+;antibact... at 52.17%
9 46aa, >Antimicrobial... at 52.17%
10 46aa, >antimicrobial... at 54.35%
11 46aa, >anti-Gram+;antibact... at 50.00%
12 46aa, >anti-Gram+;antibact... at 50.00%
13 44aa, >antimicrobial... at 45.45%
14 46aa, >anti-Gram+;Antibact... at 45.65%
15 44aa, >antimicrobial... at 50.00%
16 44aa, >antimicrobial... at 50.00%
17 46aa, >anti-Gram+;Antibact... at 47.83%
18 56aa, >Antimicrobial... at 46.43%
19 32aa, >anti-Gram+;Antibact... at 40.62%
20 33aa, >antibacterial;antim... at 45.45%
21 35aa, >antibacterial;antim... at 35.00%
22 46aa, >anti-Gram+;anti-HIV... at 47.83%
23 46aa, >anti-Gram+;Antibact... at 45.65%
24 46aa, >antimicrobial... at 56.52%
25 46aa, >Antimicrobial... *
26 46aa, >antimicrobial... at 60.87%
27 46aa, >antimicrobial... at 54.35%
28 46aa, >anti-Gram+;antibact... at 45.65%
29 46aa, >anti-Gram+;Antibact... at 58.70%
30 28aa, >antimicrobial... at 46.43%
31 70aa, >Antimicrobial... at 52.86%
32 70aa, >Antimicrobial... at 54.29%
33 84aa, >Antimicrobial... at 66.67%
34 75aa, >Antimicrobial... at 41.33%
35 84aa, >Antimicrobial... at 71.43%
36 84aa, >Antimicrobial... at 66.67%
37 84aa, >Antimicrobial... at 67.86%
38 84aa, >Antimicrobial... *
39 91aa, >Antimicrobial... *
40 86aa, >Antimicrobial... at 61.63%
41 85aa, >Antimicrobial... at 64.71%
42 84aa, >antibacterial06785... at 61.90%
43 20aa, >antibacterial10625... at 45.00%
44 23aa, >antibacterial11574... at 43.48%
>Cluster 379
0 76aa, >anti-Gram+... at 43.42%
1 91aa, >Antimicrobial... *
>Cluster 380
0 58aa, >anti-Gram+... at 63.79%

1 19aa, >anti-Gram+;antifung... at 42.11%
2 91aa, >Antimicrobial... *
3 91aa, >Antimicrobial... at 49.45%
4 21aa, >antibacterial;antim... at 47.62%
5 13aa, >antibacterial10431... at 46.15%
6 13aa, >antibacterial10433... at 46.15%
7 13aa, >antibacterial12331... at 46.15%
>Cluster 381
0 39aa, >Antimicrobial... at 41.03%
1 86aa, >Antimicrobial... at 40.70%
2 87aa, >Antimicrobial... at 40.23%
3 91aa, >Antimicrobial... *
>Cluster 382
0 16aa, >antibacterial06585... at 43.75%
1 13aa, >antibacterial06615... at 46.15%
2 91aa, >antibacterial06842... *
3 91aa, >antibacterial07107... at 80.22%
4 91aa, >antibacterial07108... at 86.81%
>Cluster 383
0 23aa, >antimicrobial... at 43.48%
1 91aa, >antibacterial07148... *
>Cluster 384
0 90aa, >Antimicrobial... *
1 24aa, >antibacterial12677... at 41.67%
>Cluster 385
0 90aa, >anti-Gram+;Antibact... *
1 18aa, >antibacterial;Antif... at 44.44%
>Cluster 386
0 56aa, >Antimicrobial... *
1 88aa, >Antimicrobial... at 40.91%
2 88aa, >Antimicrobial... at 51.14%
3 89aa, >Antimicrobial... at 49.44%
4 89aa, >Antimicrobial... at 50.56%
5 88aa, >Antimicrobial... at 45.45%
6 86aa, >Antimicrobial... at 55.81%
7 88aa, >Antimicrobial... at 59.09%
8 88aa, >Antimicrobial... at 67.05%
9 66aa, >Antimicrobial... at 40.91%
10 55aa, >Antimicrobial... at 80.91%
11 88aa, >Antimicrobial... at 83.18%
12 90aa, >Antimicrobial... *
13 88aa, >Antimicrobial... at 83.18%
14 87aa, >Antimicrobial... at 43.68%
15 89aa, >Antimicrobial... at 43.82%
16 86aa, >Antimicrobial... at 48.84%
17 89aa, >Antimicrobial... at 41.57%
18 88aa, >Antimicrobial... at 50.00%
19 88aa, >Antimicrobial... at 50.00%
20 20aa, >antibacterial;antim... at 50.00%
21 43aa, >Antimicrobial... *
22 88aa, >antibacterial06877... at 44.32%
23 87aa, >antibacterial06878... at 45.98%
24 88aa, >antibacterial11023... at 59.09%
25 88aa, >antibacterial11026... at 56.82%

```
>Cluster 387
0     84aa, >Antimicrobial... at 67.86%
1     81aa, >Antimicrobial... at 70.37%
2     77aa, >Antimicrobial... at 77.92%
3     90aa, >Antimicrobial... *
4     87aa, >Antimicrobial... at 62.07%
5     83aa, >Antimicrobial... at 67.47%
>Cluster 388
0     33aa, >antibacterial;antim... at 42.42%
1     88aa, >antibacterial06456... at 46.59%
2     88aa, >antibacterial06460... at 48.86%
3     90aa, >antibacterial06738... *
4     90aa, >antibacterial06739... at 57.78%
5     89aa, >antibacterial06744... at 51.69%
6     88aa, >antibacterial06801... at 40.91%
7     88aa, >antibacterial06802... at 42.05%
8     86aa, >antibacterial06827... at 44.19%
9     89aa, >antibacterial07098... at 53.93%
10    86aa, >antibacterial07147... at 46.51%
11    89aa, >antibacterial07224... at 43.82%
12    88aa, >antibacterial07297... at 53.41%
13    88aa, >antibacterial07479... at 51.14%
14    88aa, >antibacterial07480... at 52.27%
15    88aa, >antibacterial07481... at 48.86%
16    88aa, >antibacterial07482... at 53.41%
17    24aa, >antibacterial08130... at 50.00%
18    24aa, >antibacterial08138... *
19    24aa, >antibacterial08140... at 45.83%
20    22aa, >antibacterial08729... at 40.91%
21    24aa, >antibacterial10317... at 41.67%
22    21aa, >antibacterial10599... at 47.62%
>Cluster 389
0     47aa, >antimicrobial... at 40.43%
1     90aa, >antibacterial10983... *
>Cluster 390
0     31aa, >Antimicrobial... at 45.16%
1     32aa, >antimicrobial... at 53.12%
2     30aa, >antimicrobial... at 46.67%
3     31aa, >Antimicrobial;Antim... at 58.06%
4     31aa, >antibacterial;antim... at 54.84%
5     30aa, >antimicrobial... at 35.00%
6     30aa, >antimicrobial... at 35.00%
7     78aa, >Antimicrobial... at 69.23%
8     89aa, >Antimicrobial... *
9     79aa, >Antimicrobial... *
10    31aa, >antibacterial10753... at 54.84%
11    20aa, >antibacterial10918... at 50.00%
12    59aa, >antibacterial11044... at 40.68%
13    23aa, >antibacterial11849... at 43.48%
>Cluster 391
0     89aa, >Antimicrobial... *
>Cluster 392
0     71aa, >anti-Gram+;Antimicr... at 46.48%
1     23aa, >anti-Gram+;antibact... at 43.48%
```

2   76aa, >Antimicrobial... at 46.05%
3   77aa, >Antimicrobial... at 40.26%
4   81aa, >Antimicrobial... at 58.02%
5   85aa, >Antimicrobial... at 47.06%
6   89aa, >Antimicrobial... *
7   65aa, >Antimicrobial... at 35.00%
8   76aa, >Antimicrobial... at 48.68%
9   34aa, >antibacterial10603... at 41.18%
10   18aa, >antibacterial11262... at 50.00%
11   23aa, >antibacterial11809... at 43.48%
>Cluster 393
0   27aa, >antimicrobial... at 44.44%
1   34aa, >antimicrobial... at 41.18%
2   11aa, >antimicrobial... at 45.45%
3   89aa, >antibacterial06482... *
4   79aa, >antibacterial06906... at 46.84%
5   89aa, >antibacterial07185... at 44.94%
6   88aa, >antibacterial07186... at 47.73%
7   76aa, >antibacterial07239... at 48.68%
8   82aa, >antibacterial07254... at 46.34%
9   82aa, >antibacterial07255... at 51.22%
10   23aa, >antibacterial08681... *
>Cluster 394
0   46aa, >antibacterial;antim... at 41.30%
1   27aa, >antimicrobial... at 40.74%
2   29aa, >antimicrobial... at 41.38%
3   24aa, >Gram-... at 41.67%
4   89aa, >antibacterial06746... *
5   88aa, >antibacterial06753... at 40.91%
6   21aa, >antibacterial07391... at 42.86%
7   20aa, >antibacterial08982... at 35.00%
8   16aa, >antibacterial10494... at 43.75%
9   26aa, >antibacterial11370... at 50.00%
10   19aa, >antibacterial12016... at 42.11%
>Cluster 395
0   19aa, >antibacterial;Antif... at 42.11%
1   24aa, >anti-Gram+;antibact... at 41.67%
2   18aa, >anti-Gram+;antibact... at 44.44%
3   89aa, >antibacterial06823... *
4   72aa, >antibacterial06824... *
5   68aa, >antibacterial06825... at 45.59%
6   68aa, >antibacterial07105... at 47.06%
7   68aa, >antibacterial10816... at 41.18%
8   68aa, >antibacterial11002... at 45.59%
9   13aa, >antibacterial12145... at 36.15%
>Cluster 396
0   22aa, >antimicrobial... at 40.91%
1   35aa, >antibacterial06523... at 35.00%
2   35aa, >antibacterial07371... at 35.00%
3   89aa, >antibacterial09483... *
>Cluster 397
0   84aa, >antibacterial07252... at 83.02%
1   89aa, >antibacterial10828... *
>Cluster 398

0 55aa, >Gram-... at 41.82%
1 89aa, >antibacterial10832... *
2 70aa, >antibacterial11065... at 61.43%
>Cluster 399
0 89aa, >antibacterial10979... *
>Cluster 400
0 43aa, >Antimicrobial... at 41.86%
1 48aa, >Antimicrobial... at 41.67%
2 42aa, >Antimicrobial... at 40.48%
3 34aa, >anti-Gram+;Antibact... at 41.18%
4 45aa, >antimicrobial... at 35.00%
5 61aa, >Antimicrobial... at 81.97%
6 64aa, >antibacterial07158... at 40.62%
7 89aa, >antibacterial11072... *
8 26aa, >antibacterial13090... at 46.15%
>Cluster 401
0 88aa, >Gram-... *
1 13aa, >antibacterial12152... at 46.15%
>Cluster 402
0 33aa, >Antimicrobial... at 42.42%
1 24aa, >Antibacterial... at 50.00%
2 38aa, >Antimicrobial... at 42.11%
3 88aa, >Antimicrobial... *
4 21aa, >antibacterial08910... at 52.38%
>Cluster 403
0 81aa, >Antimicrobial... at 80.25%
1 88aa, >Antimicrobial... *
>Cluster 404
0 33aa, >antibacterial... at 45.45%
1 25aa, >Anti-Gram-;antibact... at 44.00%
2 88aa, >antibacterial06749... *
3 87aa, >antibacterial06750... at 77.01%
4 12aa, >antibacterial07581... at 41.67%
5 24aa, >antibacterial08122... at 41.67%
6 24aa, >antibacterial08135... at 41.67%
7 18aa, >antibacterial09488... at 50.00%
>Cluster 405
0 88aa, >antibacterial06766... *
>Cluster 406
0 20aa, >antiviral... at 35.00%
1 58aa, >Antimicrobial... at 44.83%
2 22aa, >antimicrobial... at 45.45%
3 83aa, >Antimicrobial... at 57.83%
4 60aa, >Antimicrobial... at 41.67%
5 86aa, >antibacterial06907... at 52.33%
6 88aa, >antibacterial06908... *
7 20aa, >antibacterial09116... at 35.00%
8 20aa, >antibacterial13175... at 35.00%
>Cluster 407
0 33aa, >Antibacterial;Antif... at 42.42%
1 88aa, >antibacterial07135... *
2 35aa, >antibacterial10571... at 48.57%
>Cluster 408
0 83aa, >antibacterial07149... at 65.06%

1   88aa, >antibacterial07150... *
2   81aa, >antibacterial07151... at 46.91%
3   82aa, >antibacterial07152... at 54.88%
>Cluster 409
0   35aa, >antibacterial;antim... at 35.00%
1   27aa, >antibacterial06705... at 40.74%
2   82aa, >antibacterial06800... at 52.44%
3   77aa, >antibacterial06904... at 41.56%
4   62aa, >antibacterial06905... at 66.13%
5   85aa, >antibacterial07243... at 81.18%
6   88aa, >antibacterial07244... *
7   88aa, >antibacterial07245... at 81.82%
8   77aa, >antibacterial07246... at 53.25%
9   82aa, >antibacterial07247... at 51.22%
>Cluster 410
0   25aa, >Antibacterial;antim... at 56.00%
1   25aa, >antimicrobial... *
2   24aa, >antifungal;antimicr... at 75.00%
3   24aa, >antimicrobial... at 79.17%
4   88aa, >antibacterial07258... *
5   81aa, >antibacterial07259... at 86.42%
6   64aa, >antibacterial07260... at 53.12%
7   15aa, >antibacterial11356... at 35.00%
>Cluster 411
0   88aa, >antibacterial07495... *
1   20aa, >antibacterial07946... at 35.00%
>Cluster 412
0   88aa, >antibacterial09677... *
>Cluster 413
0   88aa, >antibacterial11000... *
>Cluster 414
0   87aa, >Antibacterial... *
>Cluster 415
0   87aa, >Antimicrobial... *
>Cluster 416
0   87aa, >Antimicrobial... *
1   83aa, >Antimicrobial... at 51.81%
2   51aa, >Antibacterial;Gram-... at 41.18%
3   11aa, >antibacterial13245... at 45.45%
>Cluster 417
0   87aa, >Antimicrobial... *
1   50aa, >Antimicrobial... at 42.00%
>Cluster 418
0   87aa, >anti-Gram-... *
1   18aa, >antibacterial07043... at 44.44%
2   26aa, >antibacterial11460... at 42.31%
>Cluster 419
0   32aa, >anti-Gram+;antibact... at 50.00%
1   28aa, >anti-Gram+;Antibact... at 42.86%
2   17aa, >antibacterial08393... at 41.18%
3   87aa, >antibacterial10827... *
>Cluster 420
0   13aa, >antibacterial06599... at 46.15%
1   33aa, >antibacterial08219... at 42.42%

```
2     19aa, >antibacterial09220... at 42.11%
3     87aa, >antibacterial10861... *
>Cluster 421
0     31aa, >antimicrobial... at 45.16%
1     36aa, >antibacterial... at 41.67%
2     87aa, >antibacterial11004... *
>Cluster 422
0     67aa, >Antimicrobial... at 61.19%
1     82aa, >Antibacterial;Antif... *
2     74aa, >Antimicrobial... at 63.51%
3     72aa, >Antimicrobial... at 66.67%
4     72aa, >Antimicrobial... at 59.72%
5     75aa, >Antimicrobial... at 81.33%
6     71aa, >Antimicrobial... at 71.83%
7     67aa, >Antimicrobial... at 65.67%
8     79aa, >Antimicrobial... at 65.82%
9     71aa, >Antimicrobial... at 66.20%
10    80aa, >Antimicrobial... at 60.00%
11    72aa, >Antimicrobial... at 63.89%
12    42aa, >antibacterial08795... at 40.48%
13    22aa, >antibacterial09615... at 40.91%
14    72aa, >antibacterial11075... at 68.06%
15    73aa, >antibacterial11076... at 67.12%
16    73aa, >antibacterial11077... at 65.75%
17    71aa, >antibacterial11078... at 59.15%
18    87aa, >antibacterial11079... *
19    79aa, >antibacterial11080... *
20    71aa, >antibacterial11081... at 78.87%
21    79aa, >antibacterial11082... at 65.82%
>Cluster 423
0     86aa, >anti-Gram+;Antibact... *
1     50aa, >Antimicrobial... at 35.00%
2     30aa, >antibacterial11513... at 35.00%
>Cluster 424
0     79aa, >Antimicrobial... at 59.49%
1     86aa, >Antimicrobial... *
2     33aa, >antimicrobial... at 42.42%
>Cluster 425
0     86aa, >Antifungal... *
>Cluster 426
0     27aa, >antimicrobial... at 40.74%
1     83aa, >Antimicrobial... at 66.27%
2     81aa, >Antimicrobial... at 75.31%
3     83aa, >Antimicrobial... at 61.45%
4     86aa, >Antimicrobial... *
5     83aa, >Antimicrobial... at 79.52%
6     84aa, >Antimicrobial... at 64.29%
7     83aa, >Antimicrobial... at 79.52%
8     83aa, >Antimicrobial... at 62.65%
9     83aa, >Antimicrobial... at 69.88%
10    81aa, >Antimicrobial... at 70.37%
11    82aa, >Antimicrobial... at 78.05%
12    82aa, >Antimicrobial... at 73.17%
13    82aa, >Antimicrobial... *
```

```
14    83aa, >Antimicrobial... at 56.63%
15    54aa, >Antimicrobial... at 46.30%
16    54aa, >Antimicrobial... at 70.37%
17    54aa, >Antimicrobial... at 75.93%
18    83aa, >antibacterial06742... at 54.22%
19    82aa, >antibacterial06743... at 74.39%
>Cluster 427
0     86aa, >Antimicrobial... *
1     65aa, >Antimicrobial... at 61.54%
2     18aa, >antibacterial11531... at 44.44%
>Cluster 428
0     86aa, >antibacterial06465... *
1     86aa, >antibacterial06466... at 73.26%
>Cluster 429
0     86aa, >antibacterial06818... *
1     26aa, >antibacterial09000... at 42.31%
2     24aa, >antibacterial10363... at 41.67%
>Cluster 430
0     86aa, >antibacterial06847... *
1     24aa, >antibacterial07472... at 41.67%
2     20aa, >antibacterial09105... at 35.00%
>Cluster 431
0     38aa, >antimicrobial... at 42.11%
1     86aa, >antibacterial06871... *
2     82aa, >antibacterial06872... at 65.85%
>Cluster 432
0     37aa, >antibacterial;antim... at 43.24%
1     42aa, >antimicrobial... at 40.48%
2     86aa, >antibacterial06919... *
>Cluster 433
0     81aa, >antibacterial06720... at 44.44%
1     82aa, >antibacterial06769... at 45.12%
2     85aa, >antibacterial06783... at 43.53%
3     86aa, >antibacterial07134... *
4     84aa, >antibacterial07232... at 44.05%
5     86aa, >antibacterial07451... at 40.70%
6     86aa, >antibacterial07452... at 41.86%
7     84aa, >antibacterial07503... at 50.00%
8     21aa, >antibacterial10372... at 42.86%
9     35aa, >antibacterial12928... at 35.00%
>Cluster 434
0     21aa, >Antimicrobial... at 42.86%
1     86aa, >antibacterial07153... *
>Cluster 435
0     84aa, >antibacterial07141... at 46.43%
1     86aa, >antibacterial07251... *
2     20aa, >antibacterial08254... at 35.00%
>Cluster 436
0     86aa, >antibacterial08551... *
>Cluster 437
0     24aa, >antimicrobial... at 41.67%
1     85aa, >Antimicrobial... *
>Cluster 438
0     85aa, >Antimicrobial... *
```

```
>Cluster 439
0	44aa, >antimicrobial... at 40.91%
1	22aa, >antimicrobial... at 40.91%
2	85aa, >Antimicrobial... *
3	27aa, >antibacterial07309... at 40.74%
4	20aa, >antibacterial07331... at 35.00%
5	11aa, >antibacterial10659... at 45.45%
6	84aa, >antibacterial10824... at 67.86%
7	19aa, >antibacterial11462... *
8	23aa, >antibacterial13042... at 43.48%
>Cluster 440
0	85aa, >antibacterial06449... *
1	70aa, >antibacterial06450... at 70.00%
>Cluster 441
0	78aa, >antibacterial06451... at 43.59%
1	74aa, >antibacterial06477... at 40.54%
2	76aa, >antibacterial06755... at 44.74%
3	85aa, >antibacterial06781... *
4	74aa, >antibacterial06938... at 40.54%
5	77aa, >antibacterial07132... at 40.26%
6	25aa, >antibacterial08067... at 35.00%
7	24aa, >antibacterial08145... at 41.67%
8	35aa, >antibacterial08883... at 42.86%
9	27aa, >antibacterial08926... at 40.74%
10	18aa, >antibacterial09202... at 44.44%
>Cluster 442
0	83aa, >antibacterial06765... at 46.99%
1	85aa, >antibacterial06864... *
2	85aa, >antibacterial06865... at 80.00%
3	83aa, >antibacterial07142... at 40.96%
4	84aa, >antibacterial07146... at 41.67%
5	85aa, >antibacterial07154... at 50.59%
>Cluster 443
0	30aa, >Antimicrobial... at 35.00%
1	85aa, >antibacterial06909... *
2	35aa, >antibacterial12096... at 35.00%
>Cluster 444
0	17aa, >antimicrobial... at 41.18%
1	85aa, >antibacterial07174... *
>Cluster 445
0	85aa, >antibacterial07242... *
>Cluster 446
0	85aa, >antibacterial07525... *
1	24aa, >antibacterial12399... at 41.67%
>Cluster 447
0	85aa, >antibacterial10814... *
>Cluster 448
0	33aa, >Antifungal... at 42.42%
1	85aa, >antibacterial11095... *
2	17aa, >antibacterial12345... at 41.18%
>Cluster 449
0	83aa, >Antimicrobial... at 59.04%
1	84aa, >Antibacterial;Antif... *
2	76aa, >Antimicrobial... at 42.11%
```

```
3     76aa, >Antibacterial... at 47.37%
4     52aa, >Antibacterial... at 65.38%
5     24aa, >antimicrobial... at 41.67%
6     32aa, >antibacterial11656... at 40.62%
>Cluster 450
0     84aa, >Antimicrobial... *
1     13aa, >anti-Gram-;antibact... at 53.85%
>Cluster 451
0     41aa, >Antimicrobial... *
1     37aa, >Antimicrobial... at 43.24%
2     38aa, >antimicrobial... at 42.11%
3     37aa, >Antimicrobial... at 40.54%
4     84aa, >Antimicrobial... *
5     84aa, >Antimicrobial... at 46.43%
6     39aa, >antimicrobial... at 41.03%
7     39aa, >antimicrobial... at 41.03%
8     34aa, >antimicrobial... at 44.12%
9     17aa, >antibacterial08240... at 41.18%
10    20aa, >antibacterial09122... at 35.00%
>Cluster 452
0     80aa, >Antimicrobial... at 76.25%
1     84aa, >Antimicrobial... *
2     25aa, >antibacterial;antif... at 44.00%
3     26aa, >antibacterial09849... at 50.00%
4     30aa, >antibacterial09851... at 83.33%
5     74aa, >antibacterial10985... at 68.92%
6     74aa, >antibacterial10986... at 64.86%
>Cluster 453
0     53aa, >Antimicrobial... at 50.38%
1     44aa, >Antimicrobial... at 43.18%
2     45aa, >antibacterial;Antif... at 42.22%
3     51aa, >Antimicrobial... at 56.67%
4     51aa, >Antimicrobial... at 58.63%
5     51aa, >Antimicrobial... *
6     51aa, >Antimicrobial... at 70.20%
7     69aa, >Antimicrobial... at 40.58%
8     65aa, >Antimicrobial... at 35.00%
9     84aa, >Antimicrobial... *
10    51aa, >Antimicrobial... at 78.43%
11    51aa, >Antimicrobial... at 68.63%
12    54aa, >Antimicrobial... at 75.19%
13    51aa, >Antimicrobial... at 58.82%
14    58aa, >Antimicrobial... at 50.34%
>Cluster 454
0     37aa, >Gram-... at 40.54%
1     69aa, >anti-Gram+;Antibact... at 46.38%
2     30aa, >Antibacterial;Antif... at 43.33%
3     28aa, >Antimicrobial... at 42.86%
4     34aa, >Antimicrobial;Gram-... at 52.94%
5     34aa, >anti-Gram+;Antimicr... at 47.06%
6     84aa, >antibacterial06630... *
7     63aa, >antibacterial06634... *
8     18aa, >antibacterial08511... at 44.44%
9     33aa, >antibacterial11806... at 42.42%
```

10   43aa, >antibacterial12191... at 41.86%
11   27aa, >antibacterial12391... at 44.44%
>Cluster 455
0   84aa, >antibacterial06734... *
1   71aa, >antibacterial07136... at 50.70%
>Cluster 456
0   61aa, >Antimicrobial... at 50.82%
1   35aa, >Antimicrobial... at 65.71%
2   24aa, >anti-Gram+;Antibact... at 41.67%
3   43aa, >Antimicrobial... at 55.81%
4   41aa, >Antimicrobial... at 56.10%
5   41aa, >Antimicrobial... at 60.73%
6   41aa, >Antimicrobial... at 58.54%
7   84aa, >antibacterial06849... *
8   15aa, >antibacterial12501... at 35.00%
9   12aa, >antibacterial12592... at 41.67%
>Cluster 457
0   84aa, >antibacterial07486... *
1   19aa, >antibacterial08055... at 42.11%
>Cluster 458
0   24aa, >Antimicrobial... at 41.67%
1   20aa, >Antibacterial... at 35.00%
2   24aa, >antimicrobial... at 41.67%
3   24aa, >antibacterial;Antif... at 41.67%
4   24aa, >Antibacterial;Antif... at 41.67%
5   24aa, >Antibacterial;Antif... at 50.00%
6   24aa, >antimicrobial... at 41.67%
7   84aa, >antibacterial07494... *
>Cluster 459
0   23aa, >anti-Gram+;Antibact... at 43.48%
1   36aa, >Antimicrobial... at 44.44%
2   84aa, >antibacterial10812... *
>Cluster 460
0   84aa, >antibacterial11010... *
1   74aa, >antibacterial11011... at 75.68%
>Cluster 461
0   28aa, >antimicrobial;antiv... at 42.86%
1   84aa, >antibacterial11060... *
>Cluster 462
0   83aa, >Antimicrobial... *
1   68aa, >Antimicrobial... at 41.18%
2   49aa, >Antimicrobial... at 44.90%
3   29aa, >antibacterial12918... at 41.38%
>Cluster 463
0   83aa, >Antibacterial;Antif... *
1   26aa, >antibacterial13080... at 78.31%
>Cluster 464
0   41aa, >anti-Gram+;antibact... at 41.46%
1   81aa, >Antimicrobial... at 74.07%
2   83aa, >Antimicrobial... *
3   29aa, >antibacterial06891... at 38.38%
>Cluster 465
0   43aa, >Antimicrobial... at 60.47%
1   60aa, >Antibacterial... at 43.33%

2   44aa, >Antimicrobial... at 70.45%
3   44aa, >Antibacterial;Antif... at 77.27%
4   44aa, >Antibacterial;antif... at 65.91%
5   28aa, >antimicrobial... at 42.86%
6   43aa, >anti-Gram+;antifung... at 74.42%
7   83aa, >Antimicrobial... *
8   33aa, >anti-Gram+;Antibact... at 42.42%
9   24aa, >antibacterial08280... at 41.67%
10   82aa, >antibacterial11059... at 74.39%
>Cluster 466
0   27aa, >antibacterial;Antif... at 44.44%
1   83aa, >Antimicrobial... *
>Cluster 467
0   47aa, >antibacterial;antim... at 48.94%
1   46aa, >Antibacterial;Antif... at 43.48%
2   45aa, >antifungal;antimicr... at 35.00%
3   44aa, >antimicrobial... at 43.18%
4   42aa, >antifungal;antimicr... at 42.86%
5   42aa, >antimicrobial... at 40.48%
6   48aa, >antifungal;antimicr... at 41.67%
7   39aa, >antimicrobial... at 46.15%
8   50aa, >antifungal;antimicr... at 35.00%
9   49aa, >antimicrobial... at 42.86%
10   50aa, >Antifungal... at 35.00%
11   45aa, >antifungal;antimicr... at 35.00%
12   47aa, >antibacterial;antim... at 48.94%
13   47aa, >antimicrobial;Antim... *
14   47aa, >antifungal;antimicr... at 55.32%
15   47aa, >Antimicrobial;Antim... at 40.43%
16   47aa, >antimicrobial... at 51.06%
17   47aa, >antimicrobial... at 57.45%
18   47aa, >antimicrobial... at 48.94%
19   47aa, >Antimicrobial... at 40.43%
20   49aa, >antimicrobial... at 46.94%
21   49aa, >Antifungal;antimicr... at 57.14%
22   83aa, >antibacterial06709... *
23   64aa, >antibacterial07175... at 40.62%
24   47aa, >antibacterial07339... at 53.19%
25   45aa, >antibacterial07340... at 57.78%
26   46aa, >antibacterial07341... at 50.00%
27   45aa, >antibacterial07342... at 44.44%
28   46aa, >antibacterial07343... at 52.17%
29   49aa, >antibacterial07419... at 55.10%
30   51aa, >antibacterial08530... at 41.18%
31   47aa, >antibacterial08830... at 44.68%
32   83aa, >antibacterial10976... at 78.31%
>Cluster 468
0   31aa, >Antimicrobial... at 41.94%
1   24aa, >antibacteria;Antiba... at 45.83%
2   66aa, >anti-Gram-;antifung... at 57.58%
3   83aa, >antibacterial06758... *
>Cluster 469
0   40aa, >antimicrobial... at 35.00%
1   38aa, >antibacterial;antim... at 52.63%

```
2     38aa, >antimicrobial... at 47.37%
3     38aa, >antifungal;antimicr... at 60.53%
4     38aa, >Antimicrobial... at 63.16%
5     43aa, >anti-Gram+;antibact... at 53.49%
6     43aa, >anti-Gram+;antibact... at 55.81%
7     38aa, >Antifungal;antimicr... at 55.26%
8     44aa, >anti-Gram+;antimicr... at 54.55%
9     43aa, >anti-Gram+;antibact... at 53.49%
10    40aa, >antimicrobial... at 42.50%
11    38aa, >antifungal;antimicr... at 47.37%
12    26aa, >Antimicrobial... at 53.85%
13    82aa, >Antimicrobial... *
14    52aa, >antibacterial07307... at 46.15%
15    38aa, >antibacterial08796... at 57.89%
16    60aa, >antibacterial10771... at 80.00%
>Cluster 470
0     82aa, >Antimicrobial... *
1     19aa, >antibacterial09982... at 42.11%
2     30aa, >antibacterial10887... at 35.00%
3     18aa, >antibacterial12924... at 44.44%
>Cluster 471
0     71aa, >Antimicrobial... at 40.85%
1     71aa, >Antimicrobial... at 45.07%
2     82aa, >Antimicrobial... *
3     75aa, >antibacterial06716... at 44.00%
4     19aa, >antibacterial08580... at 47.37%
5     80aa, >antibacterial10969... at 35.00%
>Cluster 472
0     31aa, >antimicrobial... at 41.94%
1     82aa, >antiviral... *
2     82aa, >antibacterial07491... at 53.66%
>Cluster 473
0     33aa, >Antibacterial;antim... at 42.42%
1     82aa, >antibacterial10818... *
>Cluster 474
0     81aa, >anti-Gram+;Antibact... *
>Cluster 475
0     81aa, >Antimicrobial... *
1     76aa, >Antimicrobial... at 52.63%
2     76aa, >Antimicrobial... at 47.37%
3     67aa, >Antimicrobial... at 41.79%
4     67aa, >Antimicrobial... at 44.78%
5     67aa, >Antimicrobial... at 43.28%
6     77aa, >Antimicrobial... at 46.75%
7     68aa, >Antimicrobial... at 44.12%
8     20aa, >antibacterial08364... at 35.00%
>Cluster 476
0     40aa, >antibacterial;antif... at 42.50%
1     40aa, >anti-Gram-;antibact... *
2     45aa, >Antibacterial... at 73.78%
3     40aa, >anti-Gram+;antibact... at 72.50%
4     34aa, >anti-Gram+;Antibact... *
5     40aa, >anti-Gram-;antibact... at 80.00%
6     81aa, >Antimicrobial... *
```

7   63aa, >Antimicrobial... at 42.86%
8   63aa, >Antimicrobial... at 55.56%
9   64aa, >Antimicrobial... at 54.69%
10   64aa, >Antimicrobial... at 56.25%
11   63aa, >Antimicrobial... at 63.49%
12   63aa, >Antimicrobial... at 58.73%
13   63aa, >Antimicrobial... at 57.14%
14   37aa, >antibacterial06627... at 45.95%
15   30aa, >antibacterial06657... at 35.00%
16   63aa, >antibacterial06922... at 57.14%
17   63aa, >antibacterial06923... at 55.56%
18   63aa, >antibacterial06924... at 63.49%
19   63aa, >antibacterial07116... at 58.73%
20   37aa, >antibacterial07562... at 40.54%
21   39aa, >antibacterial08828... at 51.28%
22   41aa, >antibacterial09770... at 63.41%
23   27aa, >antibacterial10613... at 44.44%
24   64aa, >antibacterial11046... at 54.69%
25   63aa, >antibacterial11054... at 55.56%
26   63aa, >antibacterial11055... at 53.97%
27   63aa, >antibacterial11056... at 61.90%
28   63aa, >antibacterial11057... at 61.90%
>Cluster 477
0   27aa, >Antibacterial... at 40.74%
1   54aa, >anti-Gram-... at 70.37%
2   81aa, >Antimicrobial... *
3   46aa, >Antimicrobial... at 60.87%
4   22aa, >antimicrobial... at 40.91%
5   41aa, >Antibacterial;Antim... at 41.46%
>Cluster 478
0   52aa, >Antimicrobial... at 67.31%
1   41aa, >antifungal;antimicr... at 41.46%
2   56aa, >Antimicrobial... at 50.00%
3   53aa, >Antimicrobial... at 45.28%
4   74aa, >Antibacterial... at 43.24%
5   45aa, >Antimicrobial... at 83.33%
6   81aa, >Antimicrobial... *
>Cluster 479
0   46aa, >Antimicrobial... at 54.35%
1   46aa, >Antimicrobial... at 56.52%
2   46aa, >Antimicrobial... at 58.70%
3   59aa, >Antimicrobial... at 52.54%
4   59aa, >Antimicrobial... at 57.63%
5   44aa, >Antimicrobial... at 65.91%
6   48aa, >Antimicrobial... at 52.08%
7   47aa, >Antimicrobial... at 55.32%
8   53aa, >Antimicrobial... at 66.04%
9   59aa, >Antibacterial;Antim... at 79.66%
10   42aa, >Antimicrobial... at 57.14%
11   41aa, >Antimicrobial... at 41.46%
12   40aa, >Antimicrobial... at 50.00%
13   46aa, >Antimicrobial... at 52.17%
14   66aa, >antibacterial06863... at 40.91%
15   79aa, >antibacterial06875... at 84.81%

```
16    81aa, >antibacterial06876... *
17    65aa, >antibacterial07198... at 60.00%
18    30aa, >antibacterial09010... at 35.00%
19    40aa, >antibacterial11100... at 50.00%
>Cluster 480
0     27aa, >antibacterial;antif... at 40.74%
1     81aa, >antibacterial07300... *
2     34aa, >antibacterial07877... at 41.18%
3     20aa, >antibacterial09110... at 45.00%
>Cluster 481
0     23aa, >anti-Gram-;antifung... at 43.48%
1     58aa, >antibacterial... at 63.79%
2     26aa, >antifungal... at 42.31%
3     32aa, >antibacterial09621... at 62.50%
4     81aa, >antibacterial11067... *
5     24aa, >antibacterial12919... at 45.83%
>Cluster 482
0     29aa, >anti-Gram+;Antibact... at 41.38%
1     74aa, >Antimicrobial... at 41.89%
2     76aa, >Antimicrobial... at 60.53%
3     80aa, >Antimicrobial... *
4     58aa, >Antimicrobial... at 51.72%
5     74aa, >Antimicrobial... at 40.54%
6     22aa, >anti-Gram+;antibact... at 40.91%
>Cluster 483
0     48aa, >Antimicrobial... at 43.75%
1     44aa, >Antimicrobial... at 45.45%
2     41aa, >Antimicrobial... at 51.22%
3     42aa, >Antimicrobial... at 47.62%
4     24aa, >anti-Gram+;Antibact... at 45.83%
5     37aa, >antimicrobial... at 54.05%
6     33aa, >anti-Gram+;antibact... at 42.42%
7     30aa, >antimicrobial... at 35.00%
8     29aa, >antimicrobial... at 44.83%
9     28aa, >antimicrobial... at 46.43%
10    29aa, >Antimicrobial... at 48.28%
11    29aa, >anti-Gram+;Antibact... at 44.83%
12    29aa, >Antimicrobial... at 41.38%
13    34aa, >antibacterial;antim... at 47.06%
14    28aa, >antimicrobial... at 60.71%
15    28aa, >antifungal;antimicr... at 57.14%
16    37aa, >antimicrobial... at 70.27%
17    37aa, >antibacterial;Antif... at 45.95%
18    37aa, >Antimicrobial... at 56.76%
19    37aa, >anti-Gram+;antibact... at 56.76%
20    37aa, >antimicrobial... at 67.57%
21    37aa, >anti-Gram+;antibact... at 70.27%
22    37aa, >Antimicrobial... at 62.16%
23    37aa, >anti-Gram+;antibact... at 70.27%
24    37aa, >antimicrobial... at 70.27%
25    28aa, >anti-Gram+;antibact... at 57.14%
26    33aa, >antimicrobial... at 45.45%
27    37aa, >anti-Gram+;Antibact... at 51.35%
28    37aa, >Antimicrobial... at 59.46%
```

29 37aa, >Antimicrobial... at 70.27%
30 36aa, >Antimicrobial... at 63.89%
31 32aa, >anti-Gram+;Antibact... at 53.12%
32 33aa, >antibacterial;antim... at 45.45%
33 28aa, >antifungal;antimicr... at 53.57%
34 28aa, >anti-Gram-;antimicr... at 46.43%
35 28aa, >Antibacterial;Antif... at 57.14%
36 28aa, >antibacterial;antif... at 53.57%
37 28aa, >antibacterial;antif... at 53.57%
38 28aa, >anti-Gram+;Antimicr... at 50.00%
39 33aa, >anti-Gram+;antibact... at 42.42%
40 37aa, >anti-Gram+;Antibact... at 51.35%
41 37aa, >anti-Gram+;Antibact... at 56.76%
42 49aa, >Gram-... at 40.82%
43 37aa, >antimicrobial... at 64.86%
44 37aa, >anti-Gram+;Antibact... at 62.16%
45 28aa, >antibacterial;antim... at 50.00%
46 28aa, >anti-Gram-;antibact... at 42.86%
47 28aa, >anti-Gram-;antibact... at 42.86%
48 57aa, >Antimicrobial... at 52.63%
49 66aa, >Antimicrobial... at 63.64%
50 66aa, >Antimicrobial... *
51 58aa, >Antimicrobial... at 55.17%
52 60aa, >Antimicrobial... at 58.33%
53 37aa, >antimicrobial... at 51.35%
54 66aa, >Antimicrobial... at 53.03%
55 73aa, >Antimicrobial... at 42.47%
56 72aa, >Antimicrobial... at 43.06%
57 75aa, >Antimicrobial... at 35.00%
58 76aa, >Antimicrobial... at 46.05%
59 67aa, >Antimicrobial... at 61.19%
60 71aa, >Antimicrobial... at 53.52%
61 76aa, >Antimicrobial... at 55.26%
62 75aa, >Antimicrobial... at 60.00%
63 76aa, >Antimicrobial... at 56.58%
64 71aa, >Antimicrobial... at 49.30%
65 78aa, >Antimicrobial... at 69.23%
66 72aa, >Antimicrobial... at 51.39%
67 70aa, >Antimicrobial... at 47.14%
68 70aa, >Antimicrobial... at 48.57%
69 78aa, >Antimicrobial... at 43.59%
70 76aa, >Antimicrobial... at 59.21%
71 71aa, >Antimicrobial... at 59.15%
72 76aa, >Antimicrobial... at 64.47%
73 76aa, >Antimicrobial... at 59.21%
74 76aa, >Antimicrobial... at 57.89%
75 75aa, >Antimicrobial... at 60.00%
76 71aa, >Antimicrobial... at 61.97%
77 75aa, >Antimicrobial... at 58.67%
78 78aa, >Antimicrobial... at 73.08%
79 76aa, >Antimicrobial... at 67.11%
80 72aa, >Antibacterial... at 59.72%
81 63aa, >Antimicrobial... at 49.21%
82 73aa, >Antimicrobial... at 49.32%

83	67aa,	>Antimicrobial... at 47.76%
84	67aa,	>Antimicrobial... at 53.73%
85	70aa,	>Antimicrobial... at 52.86%
86	77aa,	>Antimicrobial... at 49.35%
87	71aa,	>Antimicrobial... at 50.70%
88	71aa,	>Antimicrobial... at 49.30%
89	71aa,	>Antimicrobial... at 46.48%
90	70aa,	>Antimicrobial... at 47.14%
91	68aa,	>Antimicrobial... at 48.53%
92	72aa,	>Antimicrobial... at 52.78%
93	75aa,	>Antimicrobial... at 60.00%
94	69aa,	>Antimicrobial... at 62.32%
95	75aa,	>Antimicrobial... at 58.67%
96	66aa,	>Antimicrobial... at 48.48%
97	71aa,	>Antimicrobial... at 64.79%
98	72aa,	>Antimicrobial... at 56.94%
99	72aa,	>Antimicrobial... at 54.17%
100	78aa,	>Antimicrobial... at 76.92%
101	75aa,	>Antimicrobial... at 60.00%
102	76aa,	>Antimicrobial... at 61.84%
103	78aa,	>Antimicrobial... at 75.64%
104	74aa,	>Antimicrobial... at 66.22%
105	70aa,	>Antimicrobial... at 47.14%
106	75aa,	>Antimicrobial... at 57.33%
107	75aa,	>Antimicrobial... at 57.33%
108	74aa,	>Antimicrobial... at 54.05%
109	63aa,	>Antimicrobial... at 55.56%
110	78aa,	>Antimicrobial... at 71.79%
111	72aa,	>Antimicrobial... at 58.33%
112	71aa,	>Antimicrobial... at 49.30%
113	76aa,	>Antimicrobial... at 73.68%
114	70aa,	>Antimicrobial... at 62.86%
115	71aa,	>Antimicrobial... at 61.97%
116	72aa,	>Antimicrobial... at 52.78%
117	72aa,	>Antimicrobial... at 51.39%
118	75aa,	>Antimicrobial... at 48.00%
119	72aa,	>Antimicrobial... at 58.33%
120	70aa,	>Antimicrobial... at 45.71%
121	70aa,	>Antimicrobial... at 48.57%
122	76aa,	>Antimicrobial... at 64.47%
123	74aa,	>Antimicrobial... at 63.51%
124	76aa,	>Antimicrobial... at 64.47%
125	65aa,	>Antimicrobial... at 49.23%
126	70aa,	>Antimicrobial... at 65.71%
127	71aa,	>Antimicrobial... at 53.52%
128	69aa,	>Antimicrobial... at 49.28%
129	63aa,	>Antimicrobial... at 60.32%
130	66aa,	>Antimicrobial... at 50.00%
131	76aa,	>Antimicrobial... at 69.74%
132	76aa,	>Antimicrobial... at 56.58%
133	73aa,	>Antimicrobial... at 61.64%
134	78aa,	>Antimicrobial... at 71.79%
135	76aa,	>Antimicrobial... at 59.21%
136	76aa,	>Antimicrobial... at 68.42%

```
137   73aa, >Antimicrobial... at 57.53%
138   70aa, >Antimicrobial... at 47.14%
139   74aa, >Antimicrobial... at 58.11%
140   70aa, >Antimicrobial... at 48.57%
141   74aa, >Antimicrobial... at 60.81%
142   74aa, >Antimicrobial... at 64.86%
143   80aa, >Antimicrobial... *
144   40aa, >Antimicrobial... at 52.50%
145   71aa, >Antimicrobial... at 53.52%
146   30aa, >Antimicrobial... at 35.00%
147   37aa, >Antibacterial;Antif... at 54.05%
148   45aa, >Antimicrobial... at 60.00%
149   46aa, >Antimicrobial... at 52.17%
150   30aa, >antimicrobial... at 43.33%
151   34aa, >antibacterial... at 47.06%
152   80aa, >antibacterial06714... at 35.00%
153   71aa, >antibacterial06788... at 56.34%
154   80aa, >antibacterial06789... at 57.50%
155   80aa, >antibacterial07088... at 43.75%
156   33aa, >antibacterial07471... at 48.48%
157   25aa, >antibacterial07483... at 52.00%
158   25aa, >antibacterial09100... at 35.00%
159   25aa, >antibacterial09101... at 35.00%
160   28aa, >antibacterial09501... at 46.43%
161   33aa, >antibacterial10106... at 42.42%
162   76aa, >antibacterial10972... at 40.79%
163   74aa, >antibacterial10974... at 41.89%
164   72aa, >antibacterial10996... at 61.11%
165   37aa, >antibacterial11845... at 48.65%
>Cluster 484
0     66aa, >Antimicrobial... at 46.97%
1     80aa, >Antimicrobial... *
2     13aa, >antibacterial08024... at 53.85%
3     11aa, >antibacterial08113... at 45.45%
4     23aa, >antibacterial12533... at 43.48%
5     19aa, >antibacterial13135... at 42.11%
>Cluster 485
0     80aa, >Antimicrobial... *
>Cluster 486
0     80aa, >Antimicrobial... *
>Cluster 487
0     80aa, >Antimicrobial... *
>Cluster 488
0     47aa, >Antifungal;antimicr... at 57.45%
1     49aa, >Antifungal... at 37.35%
2     47aa, >antifungal;Antimicr... *
3     48aa, >antifungal;Antimicr... at 79.17%
4     47aa, >antimicrobial... at 46.81%
5     80aa, >Antimicrobial... *
6     70aa, >antibacterial06756... at 41.43%
7     47aa, >antibacterial07831... at 69.36%
8     47aa, >antibacterial07832... at 69.36%
9     47aa, >antibacterial07833... at 82.49%
10    47aa, >antibacterial07834... at 82.49%
```

11   48aa, >antibacterial07835... at 82.49%
12   46aa, >antibacterial08526... at 76.98%
>Cluster 489
0   49aa, >Antimicrobial... at 40.82%
1   55aa, >antibacterial... at 45.45%
2   50aa, >antibacterial;Antif... at 52.00%
3   50aa, >Antifungal... at 48.00%
4   19aa, >antimicrobial... at 32.11%
5   24aa, >antibacterial;Antif... at 35.00%
6   50aa, >anti-Gram+;Antibact... *
7   50aa, >antifungal;antimicr... at 48.00%
8   50aa, >antifungal;antimicr... at 38.00%
9   49aa, >antifungal;antimicr... at 46.94%
10   50aa, >antifungal;antimicr... at 42.00%
11   50aa, >antibacterial;antif... at 54.00%
12   52aa, >antibacterial... at 57.69%
13   50aa, >antifungal;antimicr... at 35.00%
14   51aa, >antifungal;antimicr... at 47.06%
15   52aa, >Antifungal... at 76.92%
16   51aa, >Antifungal;antimicr... *
17   50aa, >Antifungal;antimicr... at 76.00%
18   80aa, >antibacterial06723... *
19   80aa, >antibacterial06724... at 48.75%
20   78aa, >antibacterial06768... at 43.59%
21   23aa, >antibacterial06969... at 43.48%
22   80aa, >antibacterial07089... at 82.75%
23   80aa, >antibacterial07090... at 86.25%
24   79aa, >antibacterial07091... at 82.14%
25   80aa, >antibacterial07092... *
26   79aa, >antibacterial07093... at 80.61%
27   70aa, >antibacterial07094... at 80.57%
28   80aa, >antibacterial07096... *
29   80aa, >antibacterial07097... at 86.25%
30   75aa, >antibacterial07102... at 35.00%
31   24aa, >antibacterial09037... at 41.67%
32   51aa, >antibacterial09052... *
33   75aa, >antibacterial09150... at 35.00%
>Cluster 490
0   50aa, >anti-Gram+;Antibact... at 66.00%
1   62aa, >Antimicrobial... at 51.61%
2   74aa, >Antimicrobial... at 40.54%
3   65aa, >Antibacterial;Antif... at 43.08%
4   65aa, >Antimicrobial... at 35.00%
5   80aa, >antibacterial06832... *
6   67aa, >antibacterial07163... at 40.30%
7   58aa, >antibacterial07171... at 79.31%
8   65aa, >antibacterial07172... at 41.54%
>Cluster 491
0   80aa, >antibacterial06883... *
1   80aa, >antibacterial06889... at 70.00%
2   23aa, >antibacterial08095... at 42.61%
3   23aa, >antibacterial08098... *
4   23aa, >antibacterial08099... at 71.30%
5   32aa, >antibacterial10522... at 40.62%

```
>Cluster 492
0	48aa, >Antibacterial;Antif... at 72.92%
1	80aa, >antibacterial06910... *
2	26aa, >antibacterial09199... at 42.31%
3	26aa, >antibacterial09200... at 42.31%
>Cluster 493
0	60aa, >Antibacterial... at 58.33%
1	80aa, >antibacterial11008... *
>Cluster 494
0	79aa, >Antibacterial... *
>Cluster 495
0	69aa, >Antimicrobial... at 47.83%
1	66aa, >Antimicrobial... at 62.12%
2	79aa, >Antimicrobial... *
3	74aa, >Antimicrobial... at 64.86%
4	77aa, >Antimicrobial... at 53.25%
5	76aa, >Antimicrobial... at 42.11%
6	66aa, >antibacterial11104... at 40.91%
>Cluster 496
0	29aa, >antimicrobial... at 51.72%
1	79aa, >Antimicrobial... *
2	79aa, >Antimicrobial... at 70.89%
3	31aa, >antibacterial07445... at 51.61%
>Cluster 497
0	40aa, >antibacterial;antim... at 42.50%
1	79aa, >Antimicrobial... *
2	79aa, >antibacterial07192... at 65.82%
>Cluster 498
0	69aa, >Antimicrobial... *
1	70aa, >Antimicrobial... at 42.86%
2	72aa, >Antimicrobial... at 51.39%
3	72aa, >Antimicrobial... at 45.83%
4	79aa, >Antimicrobial... *
5	72aa, >Antimicrobial... at 52.78%
6	35aa, >Antibacterial;Antim... at 32.86%
7	30aa, >antibacterial12453... at 35.00%
>Cluster 499
0	79aa, >Antimicrobial... *
1	79aa, >Antimicrobial... at 54.43%
2	78aa, >Antimicrobial... at 52.56%
>Cluster 500
0	79aa, >antifungal;Antimicr... *
1	79aa, >Antimicrobial... at 80.41%
2	38aa, >antimicrobial... at 41.74%
3	76aa, >antibacterial07441... at 65.79%
>Cluster 501
0	23aa, >Gram-... *
1	23aa, >Antibacterial;Antif... at 81.30%
2	22aa, >Antibacterial... at 82.91%
3	67aa, >Antimicrobial... at 68.66%
4	69aa, >Antimicrobial... at 49.28%
5	68aa, >Antimicrobial... at 48.53%
6	69aa, >Antimicrobial... at 36.38%
7	41aa, >Antimicrobial... at 60.98%
```

```
8     22aa, >antibacterial09616... at 71.82%
9     22aa, >antibacterial10109... at 78.91%
10    22aa, >antibacterial10110... at 78.91%
11    11aa, >antibacterial10601... at 45.45%
12    79aa, >antibacterial10815... *
13    67aa, >antibacterial11071... at 40.75%
>Cluster 502
0     79aa, >antibacterial10836... *
>Cluster 503
0     26aa, >Antibacterial... at 42.31%
1     71aa, >antibacterial11015... at 64.79%
2     79aa, >antibacterial11016... *
3     65aa, >antibacterial11040... at 49.23%
4     40aa, >antibacterial11089... at 50.00%
5     68aa, >antibacterial11091... at 60.29%
>Cluster 504
0     28aa, >antimicrobial... at 46.43%
1     79aa, >antibacterial11090... *
>Cluster 505
0     28aa, >antimicrobial... at 50.00%
1     77aa, >Antimicrobial... at 53.25%
2     75aa, >Antimicrobial... at 50.67%
3     78aa, >Antimicrobial... *
4     31aa, >antimicrobial... at 45.16%
>Cluster 506
0     78aa, >Antimicrobial... *
>Cluster 507
0     43aa, >Antimicrobial... at 41.86%
1     45aa, >Antibacterial... at 35.00%
2     78aa, >Antimicrobial... *
3     42aa, >antibacterial10395... at 40.48%
>Cluster 508
0     78aa, >antibacterial06519... *
1     52aa, >antibacterial06520... at 84.62%
2     45aa, >antibacterial06580... at 46.67%
3     45aa, >antibacterial06959... at 35.00%
4     24aa, >antibacterial08126... at 58.33%
5     19aa, >antibacterial08204... at 42.11%
6     24aa, >antibacterial08237... at 50.00%
7     24aa, >antibacterial12687... at 45.83%
>Cluster 509
0     76aa, >antibacterial06726... at 47.37%
1     78aa, >antibacterial06860... *
>Cluster 510
0     68aa, >antibacterial06773... at 48.53%
1     65aa, >antibacterial06774... at 50.77%
2     69aa, >antibacterial06775... at 47.83%
3     78aa, >antibacterial06915... *
4     67aa, >antibacterial07100... at 55.22%
5     64aa, >antibacterial07101... at 51.56%
6     25aa, >antibacterial10256... at 35.00%
>Cluster 511
0     78aa, >antibacterial07193... *
1     17aa, >antibacterial09934... at 41.18%
```

```
2     35aa, >antibacterial10800... at 35.00%
3     18aa, >antibacterial11901... at 44.44%
>Cluster 512
0     78aa, >antibacterial07606... *
1     19aa, >antibacterial12413... at 42.11%
>Cluster 513
0     78aa, >antibacterial09484... *
>Cluster 514
0     17aa, >antibacterial... at 52.94%
1     78aa, >antibacterial10835... *
2     21aa, >antibacterial12189... at 47.62%
>Cluster 515
0     35aa, >antibacterial07082... at 35.00%
1     78aa, >antibacterial10869... *
>Cluster 516
0     48aa, >antibacterial;antif... at 75.00%
1     26aa, >antimicrobial... at 42.31%
2     20aa, >antibacterial09108... at 35.00%
3     20aa, >antibacterial09109... at 45.00%
4     78aa, >antibacterial10967... *
>Cluster 517
0     67aa, >Antibacterial... at 40.30%
1     77aa, >Antibacterial... *
>Cluster 518
0     77aa, >anti-Gram-;Antibact... *
>Cluster 519
0     76aa, >Antimicrobial... at 51.32%
1     47aa, >Antimicrobial... at 40.43%
2     53aa, >Antimicrobial... at 63.02%
3     53aa, >Antimicrobial... at 58.49%
4     52aa, >Antimicrobial... at 53.85%
5     54aa, >Antibacterial... at 45.93%
6     53aa, >Antimicrobial... at 56.60%
7     76aa, >Antimicrobial... at 75.53%
8     77aa, >Antimicrobial... *
9     77aa, >Antimicrobial... at 82.21%
10    79aa, >Antimicrobial... at 82.21%
11    54aa, >Antimicrobial... at 57.41%
12    55aa, >Antimicrobial... at 56.36%
13    53aa, >Antimicrobial... at 47.17%
>Cluster 520
0     29aa, >anti-Gram+;antibact... at 41.38%
1     69aa, >Antimicrobial... at 57.97%
2     77aa, >Antimicrobial... *
3     62aa, >Antimicrobial... at 40.32%
4     67aa, >Antimicrobial... at 43.28%
5     53aa, >Antimicrobial... at 45.28%
6     30aa, >antibacterial07361... at 35.00%
>Cluster 521
0     77aa, >Antimicrobial... *
>Cluster 522
0     29aa, >antibacterial;antim... at 41.38%
1     77aa, >Antimicrobial... *
2     23aa, >antibacterial08218... at 52.17%
```

3   30aa, >antibacterial13273... at 43.33%
>Cluster 523
0   50aa, >antibacterial06504... at 42.00%
1   46aa, >antibacterial06539... at 41.30%
2   77aa, >antibacterial06693... *
3   49aa, >antibacterial06694... at 77.55%
4   49aa, >antibacterial06936... at 40.82%
5   45aa, >antibacterial06957... at 44.44%
6   46aa, >antibacterial06958... at 43.48%
7   46aa, >antibacterial07077... at 43.48%
8   47aa, >antibacterial07283... at 46.81%
9   22aa, >antibacterial13382... at 40.91%
>Cluster 524
0   37aa, >Antimicrobial... at 64.86%
1   77aa, >antibacterial06784... *
2   12aa, >antibacterial09947... at 41.67%
>Cluster 525
0   77aa, >antibacterial06862... *
>Cluster 526
0   48aa, >Antibacterial;Antim... at 41.67%
1   69aa, >antibacterial06882... at 42.03%
2   69aa, >antibacterial07111... at 40.58%
3   67aa, >antibacterial07203... at 58.21%
4   77aa, >antibacterial07204... *
5   73aa, >antibacterial07205... at 75.34%
6   71aa, >antibacterial07206... at 57.75%
7   71aa, >antibacterial07207... at 50.70%
>Cluster 527
0   77aa, >antibacterial07462... *
1   17aa, >antibacterial08394... at 64.71%
>Cluster 528
0   77aa, >antibacterial10352... *
>Cluster 529
0   77aa, >antibacterial10862... *
>Cluster 530
0   76aa, >Antimicrobial... *
1   75aa, >Antimicrobial... at 58.00%
2   50aa, >Antimicrobial... at 86.00%
>Cluster 531
0   76aa, >Antimicrobial... *
1   64aa, >Antimicrobial... at 42.19%
>Cluster 532
0   64aa, >Antimicrobial... at 40.62%
1   76aa, >Antimicrobial... *
2   76aa, >Antimicrobial... at 84.21%
>Cluster 533
0   49aa, >antimicrobial... at 42.86%
1   76aa, >Antifungal... *
>Cluster 534
0   70aa, >Antimicrobial... at 45.71%
1   55aa, >Antimicrobial... at 47.27%
2   71aa, >Antimicrobial... at 52.11%
3   71aa, >Antimicrobial... at 76.06%
4   76aa, >Antimicrobial... *

```
5     71aa, >Antimicrobial... at 52.11%
6     72aa, >Antimicrobial... at 50.00%
7     71aa, >Antibacterial... at 50.70%
8     76aa, >Antimicrobial... at 73.68%
9     71aa, >Antimicrobial... at 47.89%
10    72aa, >Antimicrobial... at 50.00%
11    74aa, >Antimicrobial... at 52.70%
12    69aa, >Antimicrobial... at 43.48%
13    71aa, >Antimicrobial... at 49.30%
14    48aa, >antibacterial;antim... at 41.67%
>Cluster 535
0     64aa, >Antimicrobial... at 40.62%
1     61aa, >Antimicrobial... at 40.98%
2     73aa, >Antimicrobial... at 53.42%
3     59aa, >Antimicrobial... at 42.37%
4     70aa, >Antimicrobial... at 67.14%
5     76aa, >Antimicrobial... *
6     72aa, >Antimicrobial... at 61.11%
>Cluster 536
0     53aa, >Antimicrobial... at 47.17%
1     53aa, >anti-Gram-;Antibact... at 79.25%
2     40aa, >antimicrobial... at 35.00%
3     76aa, >Antimicrobial... *
>Cluster 537
0     72aa, >Antimicrobial... at 44.44%
1     76aa, >Antimicrobial... *
>Cluster 538
0     76aa, >antibacterial;Antif... *
>Cluster 539
0     76aa, >antibacterial06555... *
>Cluster 540
0     38aa, >antibacterial;antim... at 52.63%
1     38aa, >anti-Gram+;Antibact... at 52.63%
2     39aa, >Antimicrobial... at 82.18%
3     39aa, >antimicrobial... *
4     38aa, >antimicrobial... at 52.63%
5     76aa, >antibacterial06892... *
6     73aa, >antibacterial07127... at 42.47%
7     74aa, >antibacterial07157... at 47.30%
8     18aa, >antibacterial08103... at 44.44%
9     20aa, >antibacterial09148... at 35.00%
>Cluster 541
0     76aa, >antibacterial07223... *
1     67aa, >antibacterial07261... at 68.66%
2     59aa, >antibacterial10935... at 66.10%
>Cluster 542
0     74aa, >anti-Gram+;Antibact... at 62.16%
1     76aa, >antibacterial10764... *
2     76aa, >antibacterial10822... at 52.63%
>Cluster 543
0     75aa, >Antimicrobial... *
1     73aa, >Antimicrobial... at 84.93%
2     24aa, >antimicrobial... at 41.67%
3     25aa, >antibacterial... at 35.00%
```

4   31aa, >antibacterial07040... at 45.16%
>Cluster 544
0   66aa, >Antimicrobial... at 81.82%
1   75aa, >Antimicrobial... *
2   61aa, >Antimicrobial... at 68.85%
3   66aa, >Antimicrobial... at 53.03%
4   47aa, >Antimicrobial... at 42.55%
5   44aa, >Antimicrobial... at 40.91%
6   45aa, >Antimicrobial... at 44.44%
7   68aa, >Antimicrobial... at 57.35%
8   68aa, >Antimicrobial;Antim... at 58.82%
9   68aa, >Antimicrobial... at 57.35%
>Cluster 545
0   37aa, >anti-Gram+;antifung... at 40.54%
1   75aa, >anti-Gram-;Antibact... *
2   32aa, >antimicrobial... at 43.75%
3   18aa, >antibacterial09174... at 50.00%
>Cluster 546
0   22aa, >anti-Gram+;Gram-... at 40.91%
1   69aa, >Antimicrobial... at 69.57%
2   69aa, >Antibacterial... at 85.51%
3   71aa, >Antimicrobial... at 84.51%
4   75aa, >Antimicrobial... *
5   23aa, >anti-Gram+;Antimicr... at 43.48%
>Cluster 547
0   75aa, >Antimicrobial... *
1   73aa, >Antibacterial;Antif... at 81.78%
2   54aa, >antibacterial07329... at 41.85%
3   65aa, >antibacterial07330... at 50.77%
>Cluster 548
0   75aa, >Antimicrobial... *
>Cluster 549
0   69aa, >Antimicrobial... at 40.58%
1   68aa, >Antimicrobial... at 45.59%
2   75aa, >Antimicrobial... *
>Cluster 550
0   75aa, >Antimicrobial... *
1   20aa, >antibacterial09398... at 35.00%
>Cluster 551
0   33aa, >Antimicrobial;Gram-... at 42.42%
1   33aa, >anti-Gram+;Antimicr... at 42.42%
2   75aa, >Antimicrobial... *
3   28aa, >antibacterial07038... at 42.86%
>Cluster 552
0   75aa, >Antimicrobial... *
>Cluster 553
0   75aa, >antibacterial06469... *
1   51aa, >antibacterial06517... at 43.14%
2   71aa, >antibacterial06668... at 40.85%
3   28aa, >antibacterial06690... at 42.86%
4   46aa, >antibacterial07076... at 54.35%
5   47aa, >antibacterial07286... at 46.81%
6   47aa, >antibacterial07289... at 51.06%
7   21aa, >antibacterial07393... at 42.86%

```
8     51aa, >antibacterial07406... at 41.18%
9     46aa, >antibacterial07564... at 45.65%
10    46aa, >antibacterial07617... at 50.00%
11    24aa, >antibacterial08123... at 45.83%
>Cluster 554
0     75aa, >antibacterial06471... *
1     49aa, >antibacterial06472... at 81.63%
2     66aa, >antibacterial07028... at 40.91%
3     46aa, >antibacterial07279... at 43.48%
4     18aa, >antibacterial10936... at 44.44%
>Cluster 555
0     57aa, >Antibacterial... at 43.86%
1     75aa, >antibacterial06772... *
2     73aa, >antibacterial07130... at 50.68%
3     16aa, >antibacterial13039... at 43.75%
>Cluster 556
0     22aa, >Antimicrobial... at 45.45%
1     47aa, >antifungal;antimicr... *
2     72aa, >Antimicrobial... at 77.78%
3     17aa, >antimicrobial... at 41.18%
4     45aa, >antifungal;antimicr... at 64.44%
5     46aa, >antifungal;antimicr... at 69.57%
6     75aa, >antibacterial06777... *
7     74aa, >antibacterial06778... at 61.62%
8     43aa, >antibacterial07417... at 65.12%
9     72aa, >antibacterial09151... at 74.72%
10    75aa, >antibacterial09748... at 86.67%
11    74aa, >antibacterial10806... at 72.97%
>Cluster 557
0     75aa, >antibacterial10396... *
1     75aa, >antibacterial10397... at 73.33%
>Cluster 558
0     71aa, >antibacterial07477... at 47.89%
1     75aa, >antibacterial10849... *
2     75aa, >antibacterial10850... at 68.00%
>Cluster 559
0     74aa, >Antibacterial;Antif... *
1     74aa, >Antimicrobial... at 48.65%
2     24aa, >antibacterial10113... at 45.83%
3     26aa, >antibacterial10575... at 46.15%
4     74aa, >antibacterial10984... at 47.30%
5     74aa, >antibacterial11001... at 41.89%
>Cluster 560
0     37aa, >Antimicrobial... at 40.54%
1     74aa, >Antimicrobial... *
2     45aa, >Antimicrobial... at 35.00%
>Cluster 561
0     42aa, >Antimicrobial... at 42.86%
1     13aa, >antimicrobial... at 46.15%
2     74aa, >anti-Gram+;Antibact... *
3     28aa, >antibacterial07066... at 46.43%
4     21aa, >antibacterial10144... at 42.86%
>Cluster 562
0     25aa, >antibacterial;antim... at 35.00%
```

```
1     74aa, >Antimicrobial... *
>Cluster 563
0     74aa, >anti-Gram+;Antibact... *
>Cluster 564
0     28aa, >anti-Gram+... at 46.43%
1     57aa, >Antimicrobial... at 43.86%
2     74aa, >Antimicrobial... *
3     69aa, >Antimicrobial... at 44.93%
>Cluster 565
0     50aa, >antibacterial06496... at 84.00%
1     74aa, >antibacterial06497... *
>Cluster 566
0     25aa, >antimicrobial... at 35.00%
1     24aa, >Antimicrobial... at 41.67%
2     74aa, >antibacterial06505... *
3     48aa, >antibacterial06984... at 43.75%
4     46aa, >antibacterial06985... at 50.00%
5     46aa, >antibacterial07427... at 41.30%
6     46aa, >antibacterial07514... at 45.65%
7     44aa, >antibacterial07533... at 47.73%
8     21aa, >antibacterial08545... at 42.86%
>Cluster 567
0     23aa, >antibacterial... at 43.48%
1     34aa, >antibacterial;antim... at 47.06%
2     74aa, >antibacterial06518... *
3     46aa, >antibacterial07466... at 43.48%
4     74aa, >antibacterial07490... at 41.89%
5     18aa, >antibacterial07575... at 44.44%
6     28aa, >antibacterial08440... at 42.86%
7     26aa, >antibacterial09762... at 46.15%
>Cluster 568
0     50aa, >Antibacterial;Antim... at 78.00%
1     36aa, >Antimicrobial... at 41.67%
2     42aa, >Antibacterial;Antim... at 40.48%
3     62aa, >antibacterial06821... at 50.00%
4     63aa, >antibacterial06835... at 55.56%
5     73aa, >antibacterial06836... at 49.32%
6     73aa, >antibacterial06837... at 43.84%
7     74aa, >antibacterial06838... *
8     63aa, >antibacterial11006... at 55.56%
>Cluster 569
0     70aa, >antibacterial06501... at 35.00%
1     50aa, >antibacterial06577... at 52.00%
2     70aa, >antibacterial06637... at 35.00%
3     74aa, >antibacterial07405... *
4     46aa, >antibacterial07616... at 43.48%
>Cluster 570
0     74aa, >antibacterial09106... *
1     20aa, >antibacterial10624... at 35.00%
>Cluster 571
0     26aa, >antibacterial07933... at 46.15%
1     21aa, >antibacterial08047... at 42.86%
2     74aa, >antibacterial10847... *
>Cluster 572
```

```
0     74aa, >antibacterial11037... *
>Cluster 573
0     73aa, >anti-Gram+;Antibact... *
>Cluster 574
0     26aa, >antifungal;antimicr... at 46.15%
1     23aa, >anti-Gram+;antibact... at 43.48%
2     73aa, >Antimicrobial... *
3     27aa, >antibacterial;Antif... at 40.74%
>Cluster 575
0     73aa, >Antimicrobial... *
1     72aa, >antibacterial11107... at 58.33%
>Cluster 576
0     68aa, >Antimicrobial... at 42.65%
1     73aa, >Antimicrobial... *
2     59aa, >Antimicrobial... at 54.24%
>Cluster 577
0     49aa, >antibacterial06473... at 81.63%
1     73aa, >antibacterial06474... *
2     73aa, >antibacterial06475... at 83.56%
3     71aa, >antibacterial06696... at 40.85%
4     71aa, >antibacterial06700... at 40.85%
5     46aa, >antibacterial06701... at 45.65%
6     46aa, >antibacterial07078... at 47.83%
7     71aa, >antibacterial07080... at 42.25%
8     46aa, >antibacterial07081... at 47.83%
9     49aa, >antibacterial07489... at 65.31%
10    23aa, >antibacterial11673... at 47.83%
11    28aa, >antibacterial12899... at 46.43%
>Cluster 578
0     73aa, >antibacterial06516... *
1     47aa, >antibacterial06575... at 40.43%
2     69aa, >antibacterial06578... at 40.58%
3     70aa, >antibacterial07284... at 41.43%
4     70aa, >antibacterial07285... at 42.86%
>Cluster 579
0     46aa, >antibacterial;antif... at 78.26%
1     46aa, >antibacterial;Antif... at 73.91%
2     73aa, >antibacterial06782... *
3     44aa, >antibacterial09753... at 83.18%
>Cluster 580
0     50aa, >Antibacterial;Antim... at 72.00%
1     73aa, >antibacterial06884... *
>Cluster 581
0     56aa, >Antimicrobial... at 82.14%
1     73aa, >antibacterial09612... *
2     24aa, >antibacterial10731... at 41.67%
>Cluster 582
0     72aa, >Antimicrobial... *
>Cluster 583
0     50aa, >Antimicrobial... at 52.00%
1     72aa, >Antimicrobial... *
2     49aa, >Antimicrobial... at 46.94%
3     61aa, >Antimicrobial... at 73.77%
4     58aa, >Antimicrobial... at 83.10%
```

5   61aa, >Antimicrobial... at 86.89%
>Cluster 584
0   70aa, >Antibacterial... at 72.86%
1   72aa, >Antibacterial... *
>Cluster 585
0   38aa, >anti-Gram+... at 44.74%
1   33aa, >Antimicrobial... at 42.42%
2   71aa, >Antimicrobial... at 43.66%
3   72aa, >Antimicrobial... *
4   65aa, >Antimicrobial... at 44.62%
>Cluster 586
0   64aa, >Antimicrobial... at 56.25%
1   67aa, >Antimicrobial... at 58.21%
2   65aa, >Antimicrobial... at 35.00%
3   72aa, >Antimicrobial... *
4   72aa, >Antimicrobial... at 73.61%
>Cluster 587
0   71aa, >Antimicrobial... at 61.97%
1   72aa, >Antimicrobial... *
>Cluster 588
0   72aa, >Antimicrobial... *
1   24aa, >antibacterial08674... at 41.67%
>Cluster 589
0   44aa, >Antifungal;antimicr... at 40.91%
1   44aa, >antimicrobial... at 43.18%
2   44aa, >anti-Gram+;antibact... at 83.18%
3   44aa, >Antifungal... at 86.36%
4   44aa, >Antifungal;antimicr... *
5   44aa, >Antifungal... at 78.64%
6   46aa, >antifungal;antimicr... at 80.43%
7   42aa, >antifungal;antimicr... at 42.86%
8   41aa, >antimicrobial... at 41.46%
9   54aa, >antibacterial06870... at 72.22%
10   44aa, >antibacterial09553... at 81.82%
11   44aa, >antibacterial09554... at 86.36%
12   44aa, >antibacterial09555... at 81.82%
13   44aa, >antibacterial09556... at 84.09%
14   44aa, >antibacterial09557... at 79.55%
15   44aa, >antibacterial09558... at 75.00%
16   44aa, >antibacterial09559... at 77.27%
17   72aa, >antibacterial10805... *
18   43aa, >antibacterial11497... at 83.72%
19   44aa, >antibacterial11498... at 77.27%
>Cluster 590
0   72aa, >antibacterial10826... *
>Cluster 591
0   71aa, >anti-Gram+;Antibact... *
>Cluster 592
0   71aa, >anti-Gram+;Antibact... *
1   23aa, >antibacterial07962... at 43.48%
>Cluster 593
0   46aa, >Antibacterial;Antif... at 45.65%
1   71aa, >Antifungal... *
2   43aa, >Antibacterial;Antim... at 44.19%

```
3     47aa, >Antifungal... at 48.94%
4     47aa, >antibacterial07421... at 44.68%
5     15aa, >antibacterial08699... at 35.00%
>Cluster 594
0     71aa, >Antimicrobial... *
>Cluster 595
0     46aa, >antimicrobial... at 65.22%
1     42aa, >antifungal;antimicr... at 50.00%
2     42aa, >antimicrobial... at 52.38%
3     42aa, >antimicrobial... at 52.38%
4     42aa, >antimicrobial... at 50.00%
5     71aa, >Antifungal... *
6     70aa, >antibacterial06914... at 61.43%
7     27aa, >antibacterial08928... at 44.44%
8     13aa, >antibacterial09588... at 53.85%
9     14aa, >antibacterial10791... at 42.86%
>Cluster 596
0     71aa, >Antimicrobial... *
1     38aa, >antibacterial10650... at 63.16%
>Cluster 597
0     71aa, >Antimicrobial... *
1     38aa, >antibacterial08783... at 42.11%
>Cluster 598
0     71aa, >Antimicrobial... *
>Cluster 599
0     71aa, >Antimicrobial... *
1     29aa, >antibacterial;antim... at 44.83%
2     37aa, >Antimicrobial... at 48.65%
>Cluster 600
0     45aa, >Antimicrobial... at 68.10%
1     40aa, >antibacterial;antim... *
2     41aa, >anti-Gram+;antibact... at 70.48%
3     42aa, >Antimicrobial... at 70.48%
4     46aa, >Antimicrobial... at 83.48%
5     71aa, >Antimicrobial... *
6     60aa, >Antibacterial... at 76.67%
7     43aa, >Antimicrobial... at 69.77%
>Cluster 601
0     71aa, >Antimicrobial... *
>Cluster 602
0     37aa, >antibacterial;antim... at 45.95%
1     71aa, >antibacterial06515... *
>Cluster 603
0     30aa, >Antibacterial;Antif... at 35.00%
1     20aa, >antimicrobial... at 45.00%
2     71aa, >antibacterial06576... *
3     47aa, >antibacterial06581... at 61.70%
4     71aa, >antibacterial06582... at 53.52%
>Cluster 604
0     21aa, >Antibacterial;Antif... at 42.86%
1     71aa, >antibacterial06661... *
2     71aa, >antibacterial06697... at 56.34%
3     71aa, >antibacterial06698... at 50.70%
4     71aa, >antibacterial06699... at 52.11%
```

```
5     47aa, >antibacterial07291... at 44.68%
6     71aa, >antibacterial07467... at 40.85%
>Cluster 605
0     71aa, >antibacterial06695... *
1     71aa, >antibacterial07281... at 42.25%
2     13aa, >antibacterial11467... at 46.15%
>Cluster 606
0     34aa, >antimicrobial... at 44.12%
1     16aa, >Antimicrobial... at 43.75%
2     65aa, >Antimicrobial... at 35.00%
3     60aa, >Antimicrobial... at 70.00%
4     64aa, >Antimicrobial... at 40.62%
5     41aa, >Antibacterial;Antim... at 48.78%
6     36aa, >antimicrobial... at 41.67%
7     63aa, >antibacterial06829... at 50.79%
8     64aa, >antibacterial07161... at 48.44%
9     63aa, >antibacterial07162... at 61.90%
10    67aa, >antibacterial07164... at 43.28%
11    63aa, >antibacterial07166... at 58.73%
12    63aa, >antibacterial07167... at 52.38%
13    71aa, >antibacterial07168... *
14    64aa, >antibacterial07169... at 40.62%
15    38aa, >L01A000349... at 44.74%
16    25aa, >antibacterial11409... at 44.00%
>Cluster 607
0     71aa, >antibacterial07287... *
1     71aa, >antibacterial07288... at 68.87%
2     71aa, >antibacterial07290... at 56.20%
3     30aa, >antibacterial09853... at 43.33%
4     29aa, >antibacterial11384... at 41.38%
>Cluster 608
0     36aa, >Antimicrobial... at 41.67%
1     21aa, >antibacterial07368... at 52.38%
2     71aa, >antibacterial07563... *
3     16aa, >antibacterial13333... at 50.00%
>Cluster 609
0     69aa, >anti-Gram+;Antibact... at 43.48%
1     29aa, >Anti-Gram-;antibact... at 41.38%
2     69aa, >antibacterial10742... at 42.03%
3     71aa, >antibacterial10803... *
>Cluster 610
0     71aa, >antibacterial10853... *
>Cluster 611
0     23aa, >antimicrobial... at 43.48%
1     67aa, >antibacterial06747... at 73.13%
2     22aa, >antibacterial07928... at 40.91%
3     25aa, >antibacterial08071... at 44.00%
4     71aa, >antibacterial10977... *
5     28aa, >antibacterial13258... at 50.00%
>Cluster 612
0     68aa, >antibacterial10817... at 77.94%
1     70aa, >antibacterial11017... at 35.00%
2     68aa, >antibacterial11033... at 85.29%
3     71aa, >antibacterial11034... *
```

```
4     70aa, >antibacterial11035... at 75.71%
>Cluster 613
0     70aa, >Antimicrobial... *
1     32aa, >Antimicrobial... at 40.62%
>Cluster 614
0     70aa, >Antimicrobial... *
>Cluster 615
0     51aa, >Antimicrobial... at 86.27%
1     70aa, >Antimicrobial... *
2     67aa, >antibacterial07165... at 40.30%
3     43aa, >antibacterial11122... at 80.70%
>Cluster 616
0     70aa, >anti-Gram-... *
1     18aa, >antibacterial08665... at 50.00%
>Cluster 617
0     70aa, >antibacterial06538... *
1     70aa, >antibacterial06579... at 48.57%
2     46aa, >antibacterial06636... at 47.83%
3     47aa, >antibacterial07074... at 40.43%
4     70aa, >antibacterial07280... at 57.14%
5     68aa, >antibacterial07535... at 54.41%
6     70aa, >antibacterial07614... at 47.14%
7     70aa, >antibacterial07615... at 61.43%
8     70aa, >antibacterial07619... at 57.14%
9     46aa, >antibacterial07620... at 60.87%
>Cluster 618
0     70aa, >antibacterial06624... *
1     46aa, >antibacterial07030... at 69.13%
>Cluster 619
0     70aa, >antibacterial07139... *
1     70aa, >antibacterial07303... at 42.86%
2     24aa, >antibacterial08137... at 45.83%
>Cluster 620
0     64aa, >antibacterial07140... at 45.31%
1     62aa, >antibacterial07236... at 40.32%
2     70aa, >antibacterial07238... *
3     70aa, >antibacterial07304... at 47.14%
4     55aa, >antibacterial07469... at 43.64%
5     56aa, >antibacterial07470... at 42.86%
6     32aa, >antibacterial08889... at 46.88%
>Cluster 621
0     70aa, >antibacterial07426... *
1     70aa, >antibacterial07515... at 44.29%
>Cluster 622
0     18aa, >antibacterial08662... at 44.44%
1     70aa, >antibacterial10891... *
>Cluster 623
0     69aa, >anti-Gram+;Antibact... *
>Cluster 624
0     69aa, >Antimicrobial... *
>Cluster 625
0     69aa, >Antimicrobial... *
>Cluster 626
0     45aa, >Antimicrobial... at 73.33%
```

```
1     48aa, >Antimicrobial... at 72.92%
2     48aa, >Antimicrobial... at 58.33%
3     43aa, >Antimicrobial... at 55.81%
4     69aa, >Antimicrobial... *
5     69aa, >antibacterial07159... at 62.32%
6     52aa, >antibacterial11117... at 59.62%
>Cluster 627
0     42aa, >Antimicrobial... at 42.86%
1     69aa, >Antimicrobial... *
2     67aa, >antibacterial07123... at 40.30%
3     35aa, >antibacterial07438... at 42.86%
>Cluster 628
0     40aa, >antimicrobial... at 35.00%
1     69aa, >Antimicrobial... *
2     69aa, >antibacterial11070... at 69.57%
3     20aa, >antibacterial13288... at 35.00%
>Cluster 629
0     60aa, >Antimicrobial... at 46.67%
1     69aa, >Antimicrobial... *
2     26aa, >antibacterial10253... at 42.31%
>Cluster 630
0     56aa, >antibacterial06806... at 42.86%
1     69aa, >antibacterial06913... *
2     20aa, >antibacterial12019... at 35.00%
>Cluster 631
0     24aa, >antimicrobial... at 41.67%
1     69aa, >antibacterial06920... *
2     30aa, >antibacterial07990... at 35.00%
>Cluster 632
0     36aa, >anti-Gram+;antibact... at 55.56%
1     41aa, >antibacterial;antim... at 48.78%
2     44aa, >Antimicrobial... at 43.18%
3     49aa, >Antimicrobial... at 42.86%
4     64aa, >Antimicrobial... at 42.19%
5     64aa, >Antimicrobial... at 45.31%
6     64aa, >Antimicrobial... at 42.19%
7     64aa, >Antimicrobial... at 42.19%
8     64aa, >Antimicrobial... at 45.31%
9     64aa, >Antimicrobial... at 42.19%
10    44aa, >antimicrobial... at 40.91%
11    43aa, >Antimicrobial... at 58.14%
12    34aa, >antibacterial06980... at 44.12%
13    64aa, >antibacterial07176... at 45.31%
14    64aa, >antibacterial07177... at 43.75%
15    64aa, >antibacterial07178... at 42.19%
16    64aa, >antibacterial07179... at 40.62%
17    69aa, >antibacterial07189... *
18    68aa, >antibacterial07215... at 54.41%
19    68aa, >antibacterial07216... at 60.29%
20    68aa, >antibacterial07217... at 61.76%
21    44aa, >antibacterial09765... at 40.91%
22    60aa, >antibacterial10823... at 45.00%
23    62aa, >antibacterial11074... at 41.94%
>Cluster 633
```

```
0     69aa, >antibacterial07536... *
1     24aa, >antibacterial10412... at 41.67%
>Cluster 634
0     19aa, >antibacterial08663... at 42.11%
1     69aa, >antibacterial11096... *
>Cluster 635
0     68aa, >anti-Gram+;anti-HIV... *
1     18aa, >antibacterial08891... at 44.44%
>Cluster 636
0     21aa, >antimicrobial... at 42.86%
1     68aa, >Antifungal... *
2     36aa, >antimicrobial... at 41.67%
3     31aa, >antibacterial07574... at 51.61%
4     25aa, >antibacterial08070... at 35.00%
>Cluster 637
0     68aa, >Antimicrobial... *
1     61aa, >Antimicrobial... at 44.26%
>Cluster 638
0     23aa, >anti-Gram+;Antibact... at 43.48%
1     68aa, >Antimicrobial... *
2     26aa, >antibacterial13234... at 42.31%
>Cluster 639
0     68aa, >Antimicrobial... *
1     21aa, >antimicrobial;antiv... at 42.86%
>Cluster 640
0     37aa, >Antimicrobial... at 40.54%
1     40aa, >Antimicrobial... at 42.50%
2     67aa, >Antibacterial;Antif... at 50.75%
3     68aa, >Antimicrobial... *
4     47aa, >Antibacterial;Gram-... at 53.19%
5     40aa, >antibacterial07591... at 35.00%
6     42aa, >antibacterial07602... at 40.48%
>Cluster 641
0     68aa, >Antimicrobial... *
1     68aa, >Antimicrobial... at 52.94%
>Cluster 642
0     64aa, >Antimicrobial... at 42.19%
1     68aa, >Antimicrobial... *
2     19aa, >antibacterial08540... at 42.11%
>Cluster 643
0     35aa, >Antibacterial;Antif... at 48.57%
1     45aa, >antibacterial;Antif... at 73.33%
2     68aa, >Antifungal... *
>Cluster 644
0     50aa, >anti-Gram+;antibact... at 35.00%
1     68aa, >antibacterial10834... *
2     66aa, >antibacterial10863... at 78.79%
>Cluster 645
0     67aa, >Antimicrobial... *
>Cluster 646
0     67aa, >Antibacterial... *
1     34aa, >antibacterial07461... at 41.18%
>Cluster 647
0     21aa, >antimicrobial... at 47.62%
```

1 67aa, >Antimicrobial... *
>Cluster 648
0 67aa, >Antimicrobial... *
1 26aa, >antibacterial11315... at 42.31%
>Cluster 649
0 67aa, >Antimicrobial... *
>Cluster 650
0 30aa, >Antibacterial;Antif... at 35.00%
1 39aa, >antibacterial;Antif... at 69.23%
2 39aa, >Anti-Gram-... at 43.59%
3 64aa, >Antimicrobial... at 50.00%
4 62aa, >Antimicrobial... at 46.77%
5 63aa, >Antimicrobial... at 58.73%
6 63aa, >Antimicrobial... at 52.38%
7 62aa, >Antimicrobial... at 48.39%
8 63aa, >Antimicrobial... at 52.38%
9 62aa, >Antimicrobial... at 48.39%
10 67aa, >Antimicrobial... *
11 64aa, >Antimicrobial... at 51.56%
12 65aa, >Antimicrobial... at 35.00%
13 62aa, >Antimicrobial... at 46.77%
14 64aa, >Antimicrobial... at 53.12%
15 62aa, >Antimicrobial... at 45.16%
16 62aa, >Antimicrobial... at 59.68%
17 63aa, >Antimicrobial... at 53.97%
18 63aa, >Antimicrobial... at 52.38%
19 62aa, >Antimicrobial... at 67.74%
20 63aa, >Antimicrobial... at 46.03%
21 65aa, >Antimicrobial... at 41.54%
22 66aa, >Antimicrobial... at 42.42%
23 41aa, >antibacterial;antim... at 53.66%
24 40aa, >Antibacterial... at 50.00%
25 66aa, >antibacterial06855... at 46.97%
26 63aa, >antibacterial06916... at 42.86%
27 62aa, >antibacterial06918... at 58.06%
28 65aa, >antibacterial06925... at 47.69%
29 62aa, >antibacterial06926... at 43.55%
30 63aa, >antibacterial06927... at 47.62%
31 63aa, >antibacterial06928... at 49.21%
32 61aa, >antibacterial06929... at 63.93%
33 62aa, >antibacterial06930... at 58.06%
34 63aa, >antibacterial07117... at 50.79%
35 63aa, >antibacterial07118... at 47.62%
36 59aa, >antibacterial07121... at 49.15%
37 63aa, >antibacterial07122... at 42.86%
38 62aa, >antibacterial07131... at 51.61%
39 32aa, >antibacterial07558... at 43.75%
40 38aa, >antibacterial07744... at 55.26%
41 39aa, >antibacterial07757... at 61.54%
42 37aa, >antibacterial08410... at 43.24%
43 38aa, >antibacterial08829... at 47.37%
44 39aa, >antibacterial10679... at 61.54%
45 58aa, >antibacterial10942... at 44.83%
46 65aa, >antibacterial11012... at 50.77%

47   62aa, >antibacterial11050... at 46.77%
48   63aa, >antibacterial11051... at 47.62%
49   67aa, >antibacterial11052... at 49.25%
50   59aa, >antibacterial11053... at 52.54%
51   65aa, >antibacterial11061... at 41.54%
52   63aa, >antibacterial11062... at 49.21%
53   23aa, >antibacterial13345... at 73.91%
>Cluster 651
0   65aa, >Antimicrobial... at 44.62%
1   67aa, >Antimicrobial... *
>Cluster 652
0   21aa, >antimicrobial... at 42.86%
1   44aa, >antibacterial;antim... at 43.18%
2   62aa, >Antimicrobial... at 43.55%
3   65aa, >Antimicrobial... at 50.77%
4   67aa, >Antimicrobial... *
5   30aa, >antibacterial10619... at 43.33%
>Cluster 653
0   64aa, >Antimicrobial... at 46.88%
1   67aa, >Antimicrobial... *
2   66aa, >Antimicrobial... at 43.94%
>Cluster 654
0   38aa, >Antibacterial... at 78.95%
1   42aa, >anti-Gram+;antibact... at 83.33%
2   28aa, >Antimicrobial... at 42.86%
3   28aa, >anti-Gram-;Antibact... at 42.86%
4   36aa, >antibacterial;antim... at 44.44%
5   63aa, >Antimicrobial... at 84.13%
6   67aa, >Antimicrobial... *
7   67aa, >Antibacterial... at 85.07%
8   64aa, >Antimicrobial... at 43.75%
9   64aa, >Antimicrobial... at 43.75%
10   64aa, >Antimicrobial... at 40.62%
11   35aa, >Antimicrobial... at 35.00%
12   64aa, >antibacterial07170... at 43.75%
13   42aa, >antibacterial09658... at 80.95%
14   35aa, >antibacterial10598... at 48.57%
>Cluster 655
0   24aa, >antimicrobial... at 41.67%
1   24aa, >antimicrobial... at 41.67%
2   24aa, >Antimicrobial... at 41.67%
3   24aa, >antimicrobial... at 45.83%
4   67aa, >Antimicrobial... *
5   24aa, >anti-Gram+;antifung... at 41.67%
6   24aa, >antimicrobial... at 41.67%
>Cluster 656
0   46aa, >Antimicrobial... at 69.57%
1   48aa, >Antimicrobial... at 81.25%
2   46aa, >Antibacterial... at 69.57%
3   50aa, >Antimicrobial... at 70.00%
4   46aa, >Antimicrobial... at 52.17%
5   46aa, >Antibacterial;Antim... at 76.09%
6   52aa, >Antimicrobial... at 76.92%
7   54aa, >Antimicrobial... at 61.11%

```
8     48aa, >Antimicrobial... at 70.83%
9     48aa, >Antimicrobial... at 77.08%
10    48aa, >Antimicrobial... at 66.67%
11    48aa, >Antimicrobial... at 70.83%
12    47aa, >Antimicrobial... at 82.98%
13    48aa, >Antimicrobial... at 72.92%
14    62aa, >antibacterial06815... at 40.32%
15    67aa, >antibacterial06833... *
16    62aa, >antibacterial06834... at 51.61%
17    16aa, >antibacterial07011... at 43.75%
>Cluster 657
0     27aa, >antimicrobial... at 40.74%
1     33aa, >antibacterial06446... at 45.45%
2     67aa, >antibacterial06851... *
3     42aa, >antibacterial07624... at 42.86%
>Cluster 658
0     23aa, >Anti-Gram-;antibact... at 43.48%
1     67aa, >antibacterial06896... *
2     60aa, >antibacterial06897... at 66.67%
3     62aa, >antibacterial06898... at 51.61%
4     60aa, >antibacterial06899... at 68.33%
5     57aa, >antibacterial06900... at 68.42%
>Cluster 659
0     32aa, >Antimicrobial... at 40.62%
1     21aa, >antibacterial08454... at 42.86%
2     67aa, >antibacterial10999... *
>Cluster 660
0     29aa, >antimicrobial... at 41.38%
1     29aa, >antibacterial;antim... at 41.38%
2     24aa, >antibacterial;Antif... at 45.83%
3     60aa, >antibacterial06921... at 51.67%
4     67aa, >antibacterial11042... *
5     60aa, >antibacterial11043... at 45.00%
6     62aa, >antibacterial11047... at 45.16%
7     60aa, >antibacterial11049... at 50.00%
>Cluster 661
0     66aa, >anti-Gram+;Antimicr... *
1     64aa, >antibacterial08089... at 78.12%
2     66aa, >antibacterial08090... at 77.88%
3     66aa, >antibacterial10916... at 86.36%
>Cluster 662
0     66aa, >Antibacterial... *
>Cluster 663
0     65aa, >Antimicrobial... at 56.92%
1     66aa, >Antimicrobial... *
2     60aa, >Antimicrobial... at 48.33%
>Cluster 664
0     60aa, >Antimicrobial... at 41.67%
1     61aa, >Antimicrobial... at 50.82%
2     61aa, >Antimicrobial... at 44.26%
3     66aa, >Antimicrobial... *
4     61aa, >Antimicrobial... at 44.26%
>Cluster 665
0     37aa, >antibacterial... at 40.54%
```

1 24aa, >antimicrobial... at 45.83%
2 35aa, >anti-Gram+;antifung... at 60.00%
3 51aa, >Antimicrobial... at 52.94%
4 36aa, >antibacterial... at 66.67%
5 26aa, >Antimicrobial... at 46.15%
6 35aa, >antibacterial... at 48.57%
7 63aa, >Antimicrobial... at 47.62%
8 66aa, >Antimicrobial... *
9 24aa, >antibacterial07036... at 41.67%
10 59aa, >antibacterial07119... at 42.37%
11 36aa, >antibacterial08273... at 41.67%
12 31aa, >antibacterial10309... at 51.61%
13 34aa, >antibacterial10948... at 44.12%
14 40aa, >antibacterial11103... at 35.00%
>Cluster 666
0 66aa, >Antimicrobial... *
>Cluster 667
0 66aa, >Antimicrobial... *
>Cluster 668
0 34aa, >anti-Gram+;Antibact... at 47.06%
1 32aa, >Antimicrobial... at 40.62%
2 65aa, >antibacterial06736... at 58.46%
3 66aa, >antibacterial06737... *
4 61aa, >antibacterial07073... at 62.30%
>Cluster 669
0 29aa, >antimicrobial... at 48.28%
1 29aa, >antimicrobial... at 41.38%
2 37aa, >anti-Gram+;Antimicr... at 40.54%
3 66aa, >antibacterial06822... *
4 26aa, >antibacterial10388... at 46.15%
5 61aa, >antibacterial11003... at 45.90%
6 35aa, >antibacterial11565... at 35.00%
7 26aa, >antibacterial12440... at 42.31%
>Cluster 670
0 42aa, >antibacterial;antim... at 45.24%
1 66aa, >antibacterial06831... *
2 33aa, >antibacterial12503... at 42.42%
>Cluster 671
0 20aa, >Antimicrobial... at 35.00%
1 66aa, >antibacterial06886... *
>Cluster 672
0 43aa, >anti-Gram+;Antibact... at 58.14%
1 39aa, >Antimicrobial... at 64.10%
2 38aa, >anti-Gram+;Antibact... at 47.37%
3 38aa, >anti-Gram+;Antibact... at 42.11%
4 64aa, >Antifungal... at 40.62%
5 66aa, >antibacterial07124... *
6 30aa, >antibacterial08781... at 35.00%
>Cluster 673
0 64aa, >Antibacterial... at 65.62%
1 36aa, >Antimicrobial... at 44.44%
2 64aa, >antibacterial08087... at 56.25%
3 66aa, >antibacterial08088... *
>Cluster 674

0	45aa, >antibacterial09858... at 71.11%
1	45aa, >antibacterial09859... at 73.33%
2	52aa, >antibacterial09860... at 100.00%
3	66aa, >antibacterial09861... *
4	31aa, >antibacterial09863... at 61.29%
5	27aa, >antibacterial09894... at 62.96%
6	41aa, >antibacterial10912... at 70.73%
7	53aa, >antibacterial10940... at 56.60%
8	62aa, >antibacterial10941... at 74.19%
9	51aa, >antibacterial11099... at 72.55%
10	37aa, >antibacterial11102... at 40.54%
11	45aa, >antibacterial11397... at 57.78%
12	28aa, >antibacterial12663... at 60.71%
13	34aa, >antibacterial12930... *
14	37aa, >antibacterial13021... at 72.97%
15	37aa, >antibacterial13022... at 72.97%
16	48aa, >antibacterial13023... at 66.67%
17	46aa, >antibacterial13166... at 80.43%
18	28aa, >antibacterial13313... at 42.86%
>Cluster 675
0	65aa, >Antibacterial... *
1	37aa, >Antifungal;antimicr... at 43.24%
>Cluster 676
0	29aa, >antibacterial;antim... at 41.38%
1	65aa, >Antimicrobial... *
2	35aa, >Antibacterial... at 35.00%
3	26aa, >antibacterial09796... at 50.00%
4	26aa, >antibacterial09800... at 50.00%
5	14aa, >antibacterial11832... at 50.00%
>Cluster 677
0	44aa, >Antimicrobial... at 75.00%
1	42aa, >Antimicrobial... at 64.29%
2	44aa, >Antibacterial... at 80.91%
3	65aa, >Antimicrobial... *
4	36aa, >Antimicrobial... at 44.44%
5	36aa, >antibacterial07292... at 41.67%
>Cluster 678
0	65aa, >Antimicrobial... *
>Cluster 679
0	65aa, >Antimicrobial... *
>Cluster 680
0	54aa, >Antimicrobial... at 61.11%
1	65aa, >Antimicrobial... *
2	64aa, >Antimicrobial... at 69.06%
3	51aa, >Antimicrobial... at 50.98%
>Cluster 681
0	65aa, >anti-Gram-... *
1	52aa, >Antimicrobial... at 50.00%
>Cluster 682
0	38aa, >anti-Gram+;Antibact... at 57.89%
1	38aa, >anti-Gram+;Antibact... at 73.68%
2	64aa, >Antimicrobial... at 53.12%
3	65aa, >Antimicrobial... *
4	20aa, >antibacterial08048... at 35.00%

```
5     23aa, >antibacterial10267... at 47.83%
6     26aa, >antibacterial12169... at 46.15%
>Cluster 683
0     47aa, >Antimicrobial... at 40.43%
1     63aa, >Antimicrobial... at 87.30%
2     65aa, >Antimicrobial... *
>Cluster 684
0     50aa, >Antimicrobial... at 64.00%
1     48aa, >Antimicrobial... at 64.58%
2     50aa, >Antimicrobial... at 66.00%
3     50aa, >Antimicrobial... at 72.00%
4     50aa, >Antimicrobial... at 66.00%
5     46aa, >Antimicrobial... *
6     50aa, >Antimicrobial... at 62.00%
7     50aa, >Antimicrobial... at 62.00%
8     53aa, >Antimicrobial... at 75.47%
9     46aa, >Antimicrobial... at 71.74%
10    65aa, >antibacterial07188... *
11    45aa, >antibacterial08012... at 77.78%
12    47aa, >antibacterial08013... at 80.00%
13    43aa, >antibacterial08014... at 77.78%
14    41aa, >antibacterial08015... at 73.33%
15    46aa, >antibacterial08016... at 86.67%
16    45aa, >antibacterial08017... at 66.00%
>Cluster 685
0     14aa, >antimicrobial... at 42.86%
1     45aa, >antibacterial;Antif... at 55.56%
2     38aa, >Antimicrobial... at 68.42%
3     40aa, >antibacterial07528... at 60.00%
4     48aa, >antibacterial10572... at 50.00%
5     65aa, >antibacterial10811... *
>Cluster 686
0     61aa, >anti-Gram+;Antibact... at 47.54%
1     64aa, >anti-Gram+;Antibact... *
>Cluster 687
0     24aa, >antimicrobial... at 45.83%
1     64aa, >Antimicrobial... *
2     20aa, >antibacterial11952... at 35.00%
>Cluster 688
0     64aa, >antibacterial... *
1     20aa, >antibacterial13048... at 35.00%
2     35aa, >antibacterial13162... at 35.00%
>Cluster 689
0     45aa, >Antimicrobial... at 83.33%
1     64aa, >Antimicrobial... *
2     63aa, >Antimicrobial... at 76.19%
>Cluster 690
0     64aa, >Antimicrobial... *
1     31aa, >antibacterial10609... at 41.94%
>Cluster 691
0     39aa, >Antimicrobial... at 41.03%
1     64aa, >Antimicrobial... *
>Cluster 692
0     64aa, >Antimicrobial... *
```

1   64aa, >Antimicrobial... at 67.19%
>Cluster 693
0   44aa, >Antimicrobial... at 45.45%
1   40aa, >Antibacterial;Antif... at 45.00%
2   30aa, >anti-Gram+;antifung... at 50.00%
3   63aa, >Antimicrobial... at 49.21%
4   64aa, >Antimicrobial... *
5   62aa, >Antimicrobial... at 46.77%
6   35aa, >antibacterial;antim... at 51.43%
7   36aa, >Antimicrobial... at 58.33%
8   36aa, >Antimicrobial... at 58.33%
9   36aa, >Antimicrobial... at 52.78%
10   36aa, >Antimicrobial... at 55.56%
11   64aa, >antibacterial07218... at 45.31%
12   38aa, >antibacterial09805... at 52.63%
>Cluster 694
0   64aa, >Antimicrobial... *
>Cluster 695
0   64aa, >Antimicrobial... *
1   64aa, >Antimicrobial... at 75.94%
>Cluster 696
0   64aa, >Gram-... *
1   20aa, >antibacterial12708... at 45.00%
2   25aa, >antibacterial13157... at 35.00%
>Cluster 697
0   63aa, >antibacterial06735... at 41.27%
1   64aa, >L01A003805... *
2   16aa, >antibacterial12967... at 43.75%
>Cluster 698
0   64aa, >antibacterial09844... *
>Cluster 699
0   60aa, >anti-Gram-... at 46.67%
1   47aa, >anti-Gram-;antimicr... at 46.81%
2   39aa, >anti-Gram-;antimicr... at 76.92%
3   63aa, >anti-Gram+;Gram-... *
4   18aa, >antibacterial10230... at 44.44%
5   37aa, >antibacterial13336... at 40.54%
>Cluster 700
0   63aa, >Anti-Gram-... *
>Cluster 701
0   35aa, >antifungal;antimicr... at 35.00%
1   63aa, >Antifungal;Antimicr... *
>Cluster 702
0   63aa, >Antimicrobial... *
1   58aa, >Antimicrobial... at 67.93%
2   58aa, >Antimicrobial... at 65.52%
3   58aa, >Antimicrobial... at 62.07%
4   39aa, >antibacterial11281... at 41.03%
>Cluster 703
0   63aa, >anti-Gram+;antifung... *
>Cluster 704
0   63aa, >Antimicrobial... *
1   34aa, >antibacterial08262... at 44.12%
>Cluster 705

```
0     21aa, >antimicrobial... at 42.86%
1     63aa, >Antimicrobial... *
2     63aa, >Antimicrobial... at 71.43%
>Cluster 706
0     63aa, >Antimicrobial... *
1     31aa, >antibacterial07853... at 48.39%
2     25aa, >antibacterial13005... at 44.00%
>Cluster 707
0     63aa, >Antimicrobial... *
>Cluster 708
0     63aa, >Antimicrobial... *
>Cluster 709
0     63aa, >Antimicrobial... *
>Cluster 710
0     63aa, >Antimicrobial... *
1     33aa, >antibacterial11253... at 42.42%
>Cluster 711
0     24aa, >Antibacterial... at 41.67%
1     40aa, >antimicrobial... at 67.50%
2     39aa, >antimicrobial... at 46.15%
3     39aa, >antimicrobial... at 46.15%
4     43aa, >antimicrobial... at 48.84%
5     40aa, >antimicrobial... at 62.50%
6     63aa, >Antibacterial... *
7     40aa, >antimicrobial... at 47.50%
8     23aa, >antibacterial11509... at 43.48%
>Cluster 712
0     63aa, >Antimicrobial... *
>Cluster 713
0     63aa, >Antibacterial;Antif... *
>Cluster 714
0     63aa, >antibacterial06528... *
1     28aa, >antibacterial06629... at 75.00%
>Cluster 715
0     44aa, >Antimicrobial... at 84.09%
1     44aa, >Antibacterial;Antim... at 84.09%
2     45aa, >Antimicrobial... at 41.36%
3     45aa, >Antimicrobial... at 63.64%
4     45aa, >Antimicrobial... at 41.36%
5     45aa, >Antimicrobial... at 68.89%
6     47aa, >Antimicrobial... at 57.45%
7     49aa, >Antimicrobial... at 53.06%
8     44aa, >Antibacterial;Antim... at 47.73%
9     63aa, >antibacterial06828... *
10    62aa, >antibacterial07160... at 43.55%
11    43aa, >antibacterial11111... *
>Cluster 716
0     34aa, >Antifungal;antimicr... at 44.12%
1     63aa, >antibacterial07604... *
2     63aa, >antibacterial07605... at 82.54%
>Cluster 717
0     63aa, >antibacterial11005... *
>Cluster 718
0     62aa, >Antimicrobial... *
```

```
>Cluster 719
0	62aa, >Antimicrobial... *
>Cluster 720
0	62aa, >Antimicrobial... *
>Cluster 721
0	62aa, >Antimicrobial... *
1	56aa, >Antimicrobial... at 41.07%
2	59aa, >Antimicrobial... at 72.88%
3	25aa, >antibacterial06531... at 35.00%
4	19aa, >antibacterial08412... at 42.11%
>Cluster 722
0	62aa, >Antimicrobial... *
1	32aa, >antibacterial10651... at 78.12%
>Cluster 723
0	62aa, >anti-Gram+;Antibact... *
>Cluster 724
0	62aa, >Antibacterial... *
>Cluster 725
0	59aa, >Antibacterial;Gram-... at 62.71%
1	62aa, >antibacterial06458... *
2	60aa, >antibacterial06672... at 56.67%
>Cluster 726
0	62aa, >antibacterial06911... *
>Cluster 727
0	42aa, >anti-Gram+;Antibact... at 40.48%
1	42aa, >Antibacterial... at 59.52%
2	42aa, >Antibacterial;Antim... at 59.52%
3	62aa, >antibacterial07180... *
4	62aa, >antibacterial07181... at 79.03%
>Cluster 728
0	55aa, >anti-Gram-... at 50.91%
1	39aa, >anti-Gram+;antibact... at 46.15%
2	39aa, >anti-Gram+;antibact... at 46.15%
3	14aa, >antimicrobial... at 42.86%
4	31aa, >Antifungal;Gram-... at 45.16%
5	30aa, >Antibacterial;Antif... at 35.00%
6	62aa, >antibacterial07916... *
7	35aa, >antibacterial08493... at 35.00%
>Cluster 729
0	61aa, >Antimicrobial... *
>Cluster 730
0	59aa, >Antimicrobial... at 50.85%
1	60aa, >Antimicrobial... at 83.33%
2	61aa, >Antimicrobial... *
3	31aa, >antibacterial06569... at 41.94%
4	13aa, >antibacterial08081... at 53.85%
5	35aa, >antibacterial09293... at 80.00%
6	35aa, >antibacterial09295... at 65.71%
7	26aa, >antibacterial12418... at 46.15%
8	18aa, >antibacterial13036... at 44.44%
>Cluster 731
0	61aa, >Antimicrobial... *
1	18aa, >antibacterial10188... at 44.44%
>Cluster 732
```

```
0     61aa, >Antimicrobial... *
1     27aa, >anti-Gram+... at 40.74%
2     29aa, >antibacterial08981... at 62.07%
>Cluster 733
0     19aa, >Antibacterial... at 42.11%
1     19aa, >Antibacterial... at 42.11%
2     60aa, >antibacterial06468... at 70.00%
3     61aa, >antibacterial06727... *
4     53aa, >antibacterial06728... at 43.40%
5     60aa, >antibacterial06729... at 58.33%
6     61aa, >antibacterial06730... at 60.66%
7     61aa, >antibacterial06731... at 62.30%
8     61aa, >antibacterial06732... at 62.30%
9     50aa, >antibacterial06803... at 35.00%
10    60aa, >antibacterial06932... at 60.00%
11    61aa, >antibacterial07095... at 62.30%
>Cluster 734
0     61aa, >antibacterial08743... *
1     20aa, >antibacterial12162... at 35.00%
>Cluster 735
0     33aa, >antimicrobial... at 45.45%
1     26aa, >antibacterial08292... at 42.31%
2     61aa, >antibacterial10890... *
>Cluster 736
0     41aa, >Antibacterial;Antim... at 41.46%
1     48aa, >Antimicrobial... at 52.08%
2     32aa, >Antimicrobial... at 43.75%
3     22aa, >antibacterial10249... at 45.45%
4     61aa, >antibacterial11101... *
>Cluster 737
0     60aa, >anti-Gram+;Antimicr... *
>Cluster 738
0     60aa, >anti-Gram+... *
>Cluster 739
0     60aa, >anti-Gram+;Antibact... *
1     27aa, >antibacterial11976... at 40.74%
>Cluster 740
0     60aa, >Antimicrobial... *
1     58aa, >Antimicrobial... at 60.34%
2     60aa, >Antimicrobial... at 60.00%
3     60aa, >Antimicrobial... at 81.67%
>Cluster 741
0     60aa, >Antimicrobial... *
1     60aa, >Antimicrobial... at 80.00%
2     60aa, >Antimicrobial... at 81.67%
>Cluster 742
0     60aa, >Antimicrobial... *
1     24aa, >antimicrobial... at 41.67%
>Cluster 743
0     58aa, >Antimicrobial... at 44.83%
1     60aa, >Antimicrobial... *
2     59aa, >Antimicrobial... at 45.76%
3     59aa, >Antimicrobial... at 44.07%
4     60aa, >Antimicrobial... at 41.67%
```

5   59aa, >antibacterial06917... at 44.07%
6   59aa, >antibacterial07115... at 42.37%
7   59aa, >antibacterial07120... at 44.07%
>Cluster 744
0   59aa, >Antimicrobial... at 50.85%
1   60aa, >Antimicrobial... *
2   30aa, >antibacterial08980... at 80.00%
>Cluster 745
0   56aa, >Antimicrobial... at 41.07%
1   56aa, >Antimicrobial... at 58.93%
2   60aa, >Antimicrobial... *
>Cluster 746
0   60aa, >anti-Gram+;Gram-... *
>Cluster 747
0   60aa, >Antifungal... *
1   47aa, >antibacterial07517... at 51.06%
>Cluster 748
0   60aa, >antibacterial07237... *
1   24aa, >antibacterial08513... at 45.83%
2   18aa, >antibacterial13099... at 50.00%
>Cluster 749
0   58aa, >antifungal... at 83.10%
1   41aa, >antibacterial07264... at 41.46%
2   60aa, >antibacterial07386... *
>Cluster 750
0   60aa, >antibacterial08263... *
>Cluster 751
0   37aa, >Antimicrobial... at 43.24%
1   46aa, >antifungal;antimicr... at 43.48%
2   60aa, >antibacterial08562... *
3   57aa, >antibacterial10346... at 73.68%
>Cluster 752
0   60aa, >antibacterial09875... *
>Cluster 753
0   60aa, >antibacterial10914... *
>Cluster 754
0   59aa, >Antimicrobial... *
>Cluster 755
0   21aa, >antibacterial;antim... at 42.86%
1   37aa, >antimicrobial... at 51.35%
2   51aa, >Antimicrobial... at 58.82%
3   49aa, >antimicrobial... at 65.31%
4   30aa, >anti-Gram+;Antibact... at 35.00%
5   34aa, >Antibacterial;Antif... at 41.18%
6   59aa, >Antimicrobial... *
7   21aa, >antimicrobial... at 52.38%
8   34aa, >antibacterial08001... at 44.12%
9   19aa, >antibacterial08582... at 42.11%
10   20aa, >antibacterial12516... at 45.00%
>Cluster 756
0   18aa, >anti-Gram+;antibact... at 44.44%
1   25aa, >antimicrobial... at 35.00%
2   24aa, >Antibacterial;Antif... at 41.67%
3   59aa, >Antimicrobial... *

```
4     30aa, >antibacterial06982... at 53.33%
5     23aa, >antibacterial09309... at 43.48%
6     59aa, >antibacterial11045... at 57.63%
7     58aa, >antibacterial11048... at 50.00%
8     23aa, >antibacterial13148... at 43.48%
>Cluster 757
0     59aa, >Antimicrobial... *
>Cluster 758
0     43aa, >Antimicrobial... at 83.72%
1     47aa, >Antimicrobial... at 74.47%
2     54aa, >Antimicrobial... at 70.37%
3     46aa, >Antimicrobial... at 80.43%
4     50aa, >Antimicrobial... at 80.00%
5     49aa, >Antimicrobial... at 77.55%
6     49aa, >Antimicrobial... at 81.63%
7     46aa, >Antimicrobial... *
8     47aa, >Antimicrobial... at 85.11%
9     49aa, >Antimicrobial... at 81.63%
10    59aa, >Antimicrobial... *
11    47aa, >Antimicrobial... at 78.72%
12    48aa, >Antimicrobial... at 62.50%
13    50aa, >Antimicrobial... at 60.00%
14    40aa, >Antimicrobial... at 50.00%
15    45aa, >Antimicrobial... at 35.00%
16    13aa, >antibacterial12155... at 61.54%
>Cluster 759
0     51aa, >Antimicrobial... at 60.78%
1     46aa, >Antimicrobial... at 65.22%
2     35aa, >antimicrobial... at 68.57%
3     48aa, >Antimicrobial... at 56.25%
4     57aa, >Antimicrobial... at 68.42%
5     51aa, >Antimicrobial... at 54.90%
6     48aa, >Antibacterial;Antim... at 62.50%
7     47aa, >Antibacterial... at 59.57%
8     58aa, >Antimicrobial... at 60.34%
9     56aa, >Antimicrobial... at 75.00%
10    59aa, >Antimicrobial... *
11    57aa, >Antimicrobial... at 70.18%
>Cluster 760
0     32aa, >antibacterial07138... at 40.62%
1     33aa, >antibacterial07549... at 42.42%
2     59aa, >antibacterial07879... *
>Cluster 761
0     28aa, >Gram-... at 42.86%
1     59aa, >antibacterial08366... *
2     58aa, >antibacterial10801... at 41.38%
3     33aa, >antibacterial11936... at 42.42%
>Cluster 762
0     30aa, >anti-Gram+;Antibact... at 35.00%
1     18aa, >antimicrobial... at 44.44%
2     18aa, >antibacterial06662... at 55.56%
3     59aa, >antibacterial09880... *
4     29aa, >antibacterial09883... at 83.10%
5     50aa, >antibacterial09886... at 64.00%
```

```
>Cluster 763
0     58aa, >Antibacterial... *
>Cluster 764
0     33aa, >antimicrobial... at 42.42%
1     58aa, >Antimicrobial... *
2     58aa, >Antimicrobial... at 83.10%
3     46aa, >Antimicrobial... at 50.00%
4     55aa, >Antimicrobial... at 35.00%
>Cluster 765
0     58aa, >Antimicrobial... *
1     23aa, >Antibacterial... at 52.17%
>Cluster 766
0     58aa, >Antimicrobial... *
1     52aa, >Antimicrobial... at 40.38%
2     20aa, >antibacterial13093... at 35.00%
>Cluster 767
0     48aa, >Antimicrobial... at 43.75%
1     58aa, >Antimicrobial... *
>Cluster 768
0     58aa, >Antimicrobial... *
>Cluster 769
0     58aa, >Antibacterial... *
>Cluster 770
0     57aa, >Antibacterial... *
>Cluster 771
0     57aa, >Antimicrobial... *
1     22aa, >anti-Gram+;antibact... at 40.91%
>Cluster 772
0     57aa, >Antibacterial;Antif... *
1     36aa, >antibacterial;antim... at 41.67%
>Cluster 773
0     18aa, >Antifungal;antimicr... at 44.44%
1     57aa, >Antimicrobial... *
>Cluster 774
0     56aa, >Antimicrobial... at 51.79%
1     53aa, >Antimicrobial... at 47.17%
2     57aa, >Antimicrobial... *
3     55aa, >Antimicrobial... at 50.91%
4     51aa, >Antimicrobial... at 64.71%
5     51aa, >Antimicrobial... at 58.82%
6     48aa, >Antimicrobial... at 50.00%
7     47aa, >Antimicrobial... at 46.81%
8     51aa, >Antimicrobial... at 45.10%
9     27aa, >antibacterial07882... at 74.07%
10    28aa, >antibacterial11181... at 42.86%
>Cluster 775
0     49aa, >Antimicrobial... at 65.31%
1     57aa, >Antimicrobial... *
2     57aa, >Antimicrobial... at 49.12%
>Cluster 776
0     51aa, >Antimicrobial... at 84.31%
1     50aa, >Antimicrobial... at 78.00%
2     51aa, >Antimicrobial... at 84.31%
3     57aa, >Antimicrobial... *
```

4   51aa, >Antimicrobial... at 84.31%
5   40aa, >Antimicrobial... at 85.00%
6   57aa, >Antimicrobial... at 71.93%
7   57aa, >Antimicrobial... at 66.67%
8   57aa, >Antimicrobial... at 70.18%
9   51aa, >Antimicrobial... at 54.90%
10   57aa, >Antimicrobial... at 68.42%
11   37aa, >antibacterial06813... *
12   36aa, >antibacterial07661... at 41.67%
13   38aa, >antibacterial09656... at 65.79%
14   24aa, >antibacterial11518... at 70.83%
>Cluster 777
0   57aa, >Antimicrobial... *
>Cluster 778
0   54aa, >Antimicrobial... at 61.11%
1   57aa, >Antimicrobial... *
2   48aa, >antimicrobial... at 41.67%
3   40aa, >antibacterial09288... at 57.50%
4   39aa, >antibacterial09299... at 74.36%
5   39aa, >antibacterial09300... at 82.05%
>Cluster 779
0   47aa, >Antimicrobial... at 42.55%
1   47aa, >Antimicrobial... at 44.68%
2   56aa, >Antimicrobial... at 57.14%
3   57aa, >Antimicrobial... *
>Cluster 780
0   57aa, >Antimicrobial... *
>Cluster 781
0   57aa, >Antimicrobial... *
1   57aa, >Antimicrobial... at 61.40%
>Cluster 782
0   55aa, >antibacterial... at 79.09%
1   57aa, >antibacterial10351... *
>Cluster 783
0   56aa, >anti-Gram+... *
1   18aa, >antibacterial09659... at 44.44%
>Cluster 784
0   39aa, >antimicrobial... at 41.03%
1   24aa, >antimicrobial... at 41.67%
2   56aa, >anti-Gram+;Antibact... *
3   23aa, >Antimicrobial... at 52.17%
>Cluster 785
0   37aa, >antibacterial;antim... at 45.95%
1   56aa, >Antifungal;Antimicr... *
2   27aa, >anti-Gram+;antibact... at 44.44%
3   34aa, >antibacterial08260... at 44.12%
>Cluster 786
0   43aa, >Antimicrobial... at 81.40%
1   38aa, >Antimicrobial... at 55.26%
2   56aa, >Antimicrobial... *
3   42aa, >Antimicrobial... at 61.90%
>Cluster 787
0   56aa, >Antimicrobial... *
>Cluster 788

0   56aa, >Antimicrobial... *
1   56aa, >Antimicrobial... at 62.50%
>Cluster 789
0   56aa, >Antimicrobial... *
>Cluster 790
0   56aa, >Antimicrobial... *
>Cluster 791
0   56aa, >Antimicrobial... *
>Cluster 792
0   56aa, >Antimicrobial... *
>Cluster 793
0   56aa, >Antimicrobial... *
1   20aa, >antibacterial08253... at 45.00%
>Cluster 794
0   56aa, >Antimicrobial... *
1   21aa, >antibacterial11916... at 47.62%
>Cluster 795
0   56aa, >antibacterial07137... *
>Cluster 796
0   56aa, >antibacterial07345... *
>Cluster 797
0   35aa, >antibacterial06494... at 42.86%
1   34aa, >antibacterial09869... at 61.76%
2   55aa, >antibacterial11097... at 67.27%
3   56aa, >antibacterial11119... *
4   27aa, >antibacterial11334... at 70.37%
5   36aa, >antibacterial11550... at 47.22%
6   36aa, >antibacterial11575... at 77.78%
7   36aa, >antibacterial12398... at 69.44%
8   38aa, >antibacterial12665... at 50.00%
9   38aa, >antibacterial12666... at 68.42%
10   36aa, >antibacterial12703... *
11   36aa, >antibacterial13087... at 55.56%
12   38aa, >antibacterial13197... at 44.74%
13   38aa, >antibacterial13198... at 47.37%
14   38aa, >antibacterial13199... at 55.26%
15   32aa, >antibacterial13236... at 53.12%
16   37aa, >antibacterial13338... at 56.76%
17   37aa, >antibacterial13340... at 62.16%
18   34aa, >antibacterial13341... at 65.29%
19   36aa, >antibacterial13344... at 66.11%
20   39aa, >antibacterial13356... at 56.41%
21   36aa, >antibacterial13417... *
22   36aa, >antibacterial13418... at 83.33%
>Cluster 798
0   55aa, >Antimicrobial... *
1   39aa, >antimicrobial... at 41.03%
2   48aa, >antifungal;antimicr... at 60.42%
>Cluster 799
0   55aa, >anti-Gram+;Antibact... *
>Cluster 800
0   55aa, >Antimicrobial... *
1   44aa, >Antimicrobial... at 68.64%
>Cluster 801

```
0     55aa, >anti-Gram+... *
1     23aa, >antimicrobial... at 33.48%
2     55aa, >anti-Gram+... at 74.55%
>Cluster 802
0     55aa, >Antimicrobial... *
1     55aa, >Antimicrobial... at 79.09%
>Cluster 803
0     55aa, >Antimicrobial... *
1     37aa, >antibacterial10713... at 54.05%
>Cluster 804
0     55aa, >Antimicrobial... *
>Cluster 805
0     55aa, >Antimicrobial... *
>Cluster 806
0     55aa, >Antimicrobial... *
1     26aa, >antibacterial10879... at 84.62%
>Cluster 807
0     55aa, >Antimicrobial... *
>Cluster 808
0     53aa, >Antimicrobial... at 47.17%
1     55aa, >Antimicrobial... *
2     30aa, >antibacterial10744... at 80.00%
3     31aa, >antibacterial10873... at 61.29%
>Cluster 809
0     55aa, >Antimicrobial... *
>Cluster 810
0     55aa, >anti-Gram+;Antibact... *
>Cluster 811
0     55aa, >anti-Gram+;antifung... *
>Cluster 812
0     55aa, >antibacterial06544... *
1     53aa, >antibacterial07233... at 47.17%
>Cluster 813
0     55aa, >antibacterial07263... *
>Cluster 814
0     55aa, >antibacterial10968... *
>Cluster 815
0     33aa, >Antimicrobial... at 66.67%
1     28aa, >Antimicrobial... at 50.00%
2     54aa, >Antimicrobial... *
3     29aa, >Antimicrobial... at 76.21%
4     26aa, >antimicrobial... at 42.31%
5     30aa, >Antimicrobial... at 73.33%
6     22aa, >Antimicrobial... at 68.18%
7     34aa, >antibacterial06962... at 75.29%
8     17aa, >antibacterial07695... *
9     14aa, >antibacterial08297... at 42.86%
10    18aa, >antibacterial09543... at 44.44%
11    13aa, >antibacterial09580... at 53.85%
12    35aa, >antibacterial09766... at 45.71%
13    23aa, >antibacterial09774... at 60.87%
14    33aa, >antibacterial11655... at 63.64%
15    13aa, >antibacterial11737... at 46.15%
16    16aa, >antibacterial12992... at 50.00%
```

```
>Cluster 816
0	54aa, >Antimicrobial... *
1	51aa, >Antimicrobial... at 47.06%
>Cluster 817
0	54aa, >Antimicrobial... *
>Cluster 818
0	48aa, >Antimicrobial... at 45.83%
1	54aa, >Antimicrobial... *
>Cluster 819
0	27aa, >antimicrobial... at 44.44%
1	54aa, >anti-Gram+;Antibact... *
2	25aa, >antimicrobial... at 35.00%
>Cluster 820
0	54aa, >Antimicrobial... *
>Cluster 821
0	22aa, >Antibacterial;Gram-... at 40.91%
1	54aa, >antibacterial07703... *
>Cluster 822
0	37aa, >antimicrobial... at 43.24%
1	37aa, >antibacterial;Gram-... at 43.24%
2	37aa, >Antibacterial... at 83.78%
3	46aa, >antibacterial08114... at 71.74%
4	52aa, >antibacterial08213... at 80.38%
5	54aa, >antibacterial08214... *
6	17aa, >antibacterial08271... at 38.82%
7	39aa, >antibacterial09061... at 43.59%
8	39aa, >antibacterial09628... *
9	37aa, >antibacterial10682... at 48.65%
>Cluster 823
0	53aa, >Antibacterial... *
1	53aa, >Antimicrobial... at 69.81%
2	34aa, >Antimicrobial... at 76.47%
>Cluster 824
0	43aa, >Antibacterial... at 83.02%
1	53aa, >Antibacterial... *
>Cluster 825
0	51aa, >Antimicrobial... at 49.02%
1	53aa, >Antimicrobial... *
2	51aa, >Antibacterial;Antim... at 45.10%
3	44aa, >anti-Gram+;antibact... at 43.18%
4	13aa, >antibacterial06593... at 53.85%
5	13aa, >antibacterial09560... at 46.15%
6	13aa, >antibacterial10392... at 53.85%
>Cluster 826
0	51aa, >Antimicrobial... at 41.18%
1	51aa, >Antimicrobial... at 41.18%
2	51aa, >Antimicrobial... at 45.10%
3	43aa, >Antimicrobial... at 41.86%
4	49aa, >Antimicrobial... at 44.90%
5	51aa, >Antimicrobial... at 43.14%
6	53aa, >Antimicrobial... *
7	51aa, >Antimicrobial... at 43.14%
8	48aa, >Antimicrobial... at 43.75%
9	53aa, >Antimicrobial... at 45.28%
```

```
10    44aa, >Antimicrobial... at 47.73%
11    47aa, >Antimicrobial... at 40.43%
>Cluster 827
0     53aa, >Antimicrobial... *
>Cluster 828
0     31aa, >Antibacterial;Antim... at 61.29%
1     53aa, >Antimicrobial... *
2     45aa, >Antimicrobial... at 35.00%
>Cluster 829
0     50aa, >Antimicrobial... at 58.00%
1     53aa, >antibacterial10732... *
>Cluster 830
0     53aa, >antibacterial10885... *
1     20aa, >antibacterial11972... at 35.00%
2     50aa, >antibacterial13414... at 84.00%
>Cluster 831
0     52aa, >anti-Gram+;Gram-... *
>Cluster 832
0     52aa, >antifungal... *
>Cluster 833
0     52aa, >antifungal... *
>Cluster 834
0     52aa, >Antimicrobial... *
1     44aa, >Antimicrobial... at 40.91%
>Cluster 835
0     52aa, >Antimicrobial... *
>Cluster 836
0     52aa, >Antimicrobial... *
>Cluster 837
0     23aa, >antimicrobial... at 43.48%
1     35aa, >antibacterial09693... at 45.71%
2     52aa, >antibacterial10296... *
3     33aa, >antibacterial10297... at 66.67%
4     48aa, >antibacterial10302... at 45.83%
5     48aa, >antibacterial10305... *
6     28aa, >antibacterial10308... at 57.14%
7     30aa, >antibacterial11548... at 35.00%
8     23aa, >antibacterial12369... at 47.83%
>Cluster 838
0     51aa, >Antimicrobial... *
1     47aa, >Antimicrobial... at 65.96%
2     51aa, >Antimicrobial... at 68.16%
3     23aa, >anti-Gram+;Antimicr... at 43.48%
4     26aa, >antifungal... at 42.31%
>Cluster 839
0     51aa, >anti-Gram+;Antibact... *
1     26aa, >antibacterial11645... at 42.31%
>Cluster 840
0     51aa, >Antifungal;Gram-... *
>Cluster 841
0     51aa, >Antimicrobial... *
1     51aa, >Antimicrobial... at 78.43%
>Cluster 842
0     44aa, >Antimicrobial... at 40.91%
```

1   51aa, >Antimicrobial... *
2   44aa, >Antimicrobial... at 43.18%
3   23aa, >antibacterial09966... at 43.48%
>Cluster 843
0   51aa, >Antimicrobial... *
>Cluster 844
0   51aa, >antibacterial;antim... *
>Cluster 845
0   50aa, >Antimicrobial... at 46.00%
1   51aa, >Antimicrobial... *
>Cluster 846
0   51aa, >anti-Gram+;Gram-... *
1   38aa, >anti-Gram+... at 50.00%
>Cluster 847
0   51aa, >anti-Gram+;Antibact... *
>Cluster 848
0   48aa, >antimicrobial... at 41.67%
1   51aa, >anti-Gram+;Gram-... *
>Cluster 849
0   29aa, >anti-Gram+;Antibact... at 41.38%
1   51aa, >antibacterial06511... *
>Cluster 850
0   39aa, >antifungal;antimicr... at 43.59%
1   51aa, >antibacterial07538... *
>Cluster 851
0   51aa, >antibacterial08464... *
>Cluster 852
0   50aa, >antimicrobial... *
1   49aa, >antimicrobial... at 53.06%
2   50aa, >antimicrobial... at 48.00%
3   50aa, >antimicrobial... at 46.00%
>Cluster 853
0   50aa, >antimicrobial... *
1   45aa, >antimicrobial... at 35.00%
>Cluster 854
0   50aa, >antimicrobial... *
>Cluster 855
0   47aa, >antimicrobial... at 57.45%
1   47aa, >antimicrobial... at 63.83%
2   50aa, >antimicrobial... *
>Cluster 856
0   28aa, >anti-Gram+;antibact... at 42.86%
1   50aa, >antibacterial;antif... *
>Cluster 857
0   50aa, >antibacterial;antim... *
>Cluster 858
0   50aa, >Antimicrobial... *
>Cluster 859
0   49aa, >antifungal;antimicr... at 46.94%
1   49aa, >antifungal;antimicr... at 46.94%
2   49aa, >antifungal;antimicr... at 48.98%
3   49aa, >antifungal;antimicr... at 44.90%
4   50aa, >antifungal;antimicr... *
>Cluster 860

0   50aa, >anti-Gram+;Gram-... *
>Cluster 861
0   50aa, >antimicrobial... *
1   37aa, >antibacterial08502... at 40.54%
>Cluster 862
0   50aa, >antibacterial07607... *
1   50aa, >antibacterial07608... at 72.00%
>Cluster 863
0   49aa, >antimicrobial... *
>Cluster 864
0   49aa, >antibacterial;Antif... *
>Cluster 865
0   49aa, >antimicrobial... *
>Cluster 866
0   21aa, >antimicrobial... at 47.62%
1   49aa, >antimicrobial... *
2   30aa, >antibacterial13385... at 35.00%
>Cluster 867
0   49aa, >antimicrobial... *
>Cluster 868
0   49aa, >antimicrobial... *
1   49aa, >antimicrobial... at 53.06%
>Cluster 869
0   49aa, >antimicrobial... *
>Cluster 870
0   49aa, >antimicrobial... *
>Cluster 871
0   49aa, >antimicrobial... *
>Cluster 872
0   49aa, >antimicrobial... *
>Cluster 873
0   16aa, >antimicrobial... at 43.75%
1   49aa, >Antimicrobial... *
>Cluster 874
0   49aa, >Antimicrobial... *
>Cluster 875
0   38aa, >Antimicrobial... at 42.11%
1   49aa, >Antimicrobial... *
2   47aa, >Antimicrobial... at 40.43%
>Cluster 876
0   49aa, >Antimicrobial... *
>Cluster 877
0   11aa, >antibacterial08300... at 45.45%
1   49aa, >antibacterial12381... *
>Cluster 878
0   48aa, >antimicrobial... *
1   45aa, >antimicrobial... at 75.56%
>Cluster 879
0   48aa, >antifungal;antimicr... *
1   48aa, >antibacterial... at 41.67%
>Cluster 880
0   48aa, >Antimicrobial... *
>Cluster 881
0   48aa, >Antibacterial;Antim... *

```
1     24aa, >antibacterial08124... at 41.67%
>Cluster 882
0     48aa, >antibacterial;antim... *
1     31aa, >anti-Gram-;antibact... at 41.94%
2     32aa, >anti-Gram+;Antibact... at 40.62%
>Cluster 883
0     48aa, >antimicrobial... *
>Cluster 884
0     48aa, >antimicrobial;antiv... *
1     31aa, >antimicrobial;antiv... at 80.32%
2     36aa, >antimicrobial;antiv... at 52.78%
3     18aa, >antibacterial09674... at 44.44%
>Cluster 885
0     48aa, >anti-Gram+;antifung... *
>Cluster 886
0     48aa, >Antimicrobial... *
>Cluster 887
0     48aa, >Antimicrobial... *
1     21aa, >antibacterial07414... at 42.86%
>Cluster 888
0     48aa, >Antimicrobial... *
>Cluster 889
0     48aa, >Antimicrobial... *
>Cluster 890
0     46aa, >antimicrobial... at 43.48%
1     48aa, >antimicrobial... *
2     39aa, >antimicrobial... at 51.28%
3     42aa, >antimicrobial... at 42.86%
4     43aa, >antimicrobial... at 60.47%
>Cluster 891
0     48aa, >antimicrobial... *
>Cluster 892
0     48aa, >antibacterial06688... *
>Cluster 893
0     48aa, >antibacterial07432... *
1     48aa, >antibacterial07433... at 67.50%
2     31aa, >antibacterial07437... at 48.39%
3     23aa, >antibacterial07924... at 43.48%
4     48aa, >antibacterial08571... at 79.58%
>Cluster 894
0     48aa, >antibacterial07492... *
1     36aa, >antibacterial10515... at 44.44%
>Cluster 895
0     48aa, >antibacterial10220... *
>Cluster 896
0     33aa, >antibacterial06459... at 48.48%
1     33aa, >antibacterial06483... at 51.52%
2     33aa, >antibacterial06522... at 54.55%
3     33aa, >antibacterial07056... at 54.55%
4     33aa, >antibacterial07064... at 54.55%
5     48aa, >antibacterial11299... *
>Cluster 897
0     26aa, >antibacterial;antim... at 42.31%
1     48aa, >antibacterial11524... *
```

```
2     41aa, >antibacterial11662... at 82.93%
3     35aa, >antibacterial12102... at 85.71%
4     46aa, >antibacterial12103... at 81.30%
5     37aa, >antibacterial12130... at 81.08%
6     40aa, >antibacterial12477... at 52.50%
7     39aa, >antibacterial12842... at 36.15%
8     42aa, >antibacterial13015... at 41.90%
>Cluster 898
0     21aa, >antimicrobial... at 47.62%
1     48aa, >antibacterial11563... *
>Cluster 899
0     47aa, >Antifungal... *
>Cluster 900
0     47aa, >Antimicrobial... *
>Cluster 901
0     47aa, >antimicrobial... *
1     18aa, >antibacterial12810... at 44.44%
>Cluster 902
0     43aa, >Antimicrobial... at 60.47%
1     47aa, >antibacterial;antim... *
2     46aa, >antimicrobial... at 54.35%
3     46aa, >antimicrobial... at 56.52%
4     38aa, >antimicrobial... at 44.74%
5     38aa, >antimicrobial... at 47.37%
6     32aa, >antibacterial11732... at 40.62%
>Cluster 903
0     47aa, >Antimicrobial... *
1     47aa, >Antimicrobial... at 80.85%
>Cluster 904
0     47aa, >antibacterial;antim... *
>Cluster 905
0     26aa, >antimicrobial... at 57.69%
1     26aa, >antimicrobial... at 80.77%
2     27aa, >antimicrobial... at 48.15%
3     47aa, >antimicrobial... *
4     29aa, >antimicrobial... at 62.07%
5     29aa, >antimicrobial... at 83.10%
>Cluster 906
0     47aa, >antimicrobial... *
>Cluster 907
0     47aa, >Antimicrobial... *
>Cluster 908
0     47aa, >Antimicrobial... *
1     44aa, >Antibacterial... at 40.91%
>Cluster 909
0     40aa, >Antimicrobial... at 35.00%
1     47aa, >Antimicrobial... *
2     47aa, >Antimicrobial... at 67.36%
>Cluster 910
0     21aa, >antimicrobial... at 42.86%
1     47aa, >antimicrobial... *
2     30aa, >antibacterial13196... at 35.00%
>Cluster 911
0     46aa, >Antimicrobial... at 47.83%
```

1   47aa, >Antimicrobial... *
>Cluster 912
0   38aa, >antimicrobial... at 42.11%
1   47aa, >Antimicrobial... *
>Cluster 913
0   47aa, >anti-Gram+... *
>Cluster 914
0   38aa, >antimicrobial... at 44.74%
1   38aa, >antimicrobial... at 44.74%
2   36aa, >antimicrobial... at 41.67%
3   33aa, >antimicrobial... at 51.52%
4   33aa, >antimicrobial... at 51.52%
5   47aa, >antimicrobial... *
6   18aa, >antibacterial09899... at 44.44%
>Cluster 915
0   46aa, >antimicrobial... *
>Cluster 916
0   46aa, >antiviral... *
>Cluster 917
0   46aa, >anti-Gram+;Antibact... *
>Cluster 918
0   46aa, >Antimicrobial... *
>Cluster 919
0   46aa, >Antibacterial;Antim... *
1   33aa, >Antimicrobial... at 42.42%
>Cluster 920
0   46aa, >Antimicrobial... *
1   44aa, >Antimicrobial... at 43.18%
>Cluster 921
0   38aa, >Antimicrobial... at 47.37%
1   46aa, >Antimicrobial... *
2   36aa, >Antimicrobial... at 41.67%
3   22aa, >antibacterial11452... at 40.91%
>Cluster 922
0   46aa, >Antimicrobial... *
1   44aa, >Antimicrobial... at 81.82%
>Cluster 923
0   46aa, >anti-Gram+;Antibact... *
>Cluster 924
0   46aa, >Antimicrobial... *
1   46aa, >Antimicrobial... at 52.17%
>Cluster 925
0   46aa, >Antimicrobial... *
>Cluster 926
0   46aa, >Antimicrobial... *
>Cluster 927
0   46aa, >Antimicrobial... *
>Cluster 928
0   46aa, >Antimicrobial... *
>Cluster 929
0   28aa, >Antimicrobial;Antim... at 42.86%
1   46aa, >antimicrobial... *
>Cluster 930
0   46aa, >antibacterial06507... *

>Cluster 931
0 46aa, >antibacterial09665... *
>Cluster 932
0 34aa, >antibacterial12603... at 55.88%
1 46aa, >antibacterial12702... *
>Cluster 933
0 38aa, >antibacterial12682... at 65.79%
1 46aa, >antibacterial12830... *
2 46aa, >antibacterial12839... at 71.74%
>Cluster 934
0 46aa, >antibacterial12986... *
>Cluster 935
0 45aa, >antibacterial;antim... *
>Cluster 936
0 45aa, >antifungal;antimicr... *
>Cluster 937
0 45aa, >anti-Gram-;anti-HIV... *
1 37aa, >Antimicrobial... at 40.54%
2 28aa, >Antimicrobial... at 42.86%
3 32aa, >antibacterial;antim... at 40.62%
>Cluster 938
0 45aa, >Antibacterial... *
1 45aa, >antibacterial09667... at 55.56%
2 45aa, >antibacterial09771... at 62.22%
3 45aa, >antibacterial09772... at 64.44%
4 45aa, >antibacterial10680... at 62.22%
5 44aa, >antibacterial10773... at 47.73%
6 45aa, >antibacterial11812... at 62.22%
7 45aa, >antibacterial11813... at 62.22%
8 45aa, >antibacterial11814... at 62.22%
9 45aa, >antibacterial11815... at 62.22%
>Cluster 939
0 45aa, >Antimicrobial... *
1 33aa, >antibacterial10947... at 60.61%
>Cluster 940
0 13aa, >Gram-... at 46.15%
1 45aa, >Antimicrobial... *
>Cluster 941
0 45aa, >antimicrobial... *
>Cluster 942
0 45aa, >Antimicrobial... *
1 25aa, >antibacterial11318... at 35.00%
>Cluster 943
0 45aa, >antimicrobial;Antim... *
>Cluster 944
0 45aa, >Antimicrobial... *
1 44aa, >Antimicrobial... at 72.73%
>Cluster 945
0 45aa, >anti-Gram+;antibact... *
>Cluster 946
0 45aa, >antibacterial06868... *
>Cluster 947
0 36aa, >antibacterial06513... at 80.00%
1 35aa, >antibacterial06954... at 81.43%

```
2     45aa, >antibacterial10915... *
3     47aa, >antibacterial11496... at 83.33%
4     35aa, >antibacterial13177... at 82.86%
>Cluster 948
0     45aa, >antibacterial11182... *
>Cluster 949
0     34aa, >antibacterial10934... at 78.24%
1     35aa, >antibacterial12023... at 81.43%
2     45aa, >antibacterial12680... *
>Cluster 950
0     22aa, >antibacterial11426... at 40.91%
1     24aa, >antibacterial12609... at 79.17%
2     37aa, >antibacterial13232... at 56.76%
3     45aa, >antibacterial13368... *
>Cluster 951
0     44aa, >anti-Gram+... *
1     44aa, >anti-Gram+... at 84.09%
>Cluster 952
0     44aa, >anti-Gram+;antibact... *
1     43aa, >antimicrobial.... at 58.14%
2     44aa, >antimicrobial.... at 75.00%
3     28aa, >Antimicrobial... at 36.43%
4     22aa, >antibacterial08760... at 40.91%
5     41aa, >antibacterial10998... at 65.85%
6     23aa, >antibacterial11131... at 52.17%
>Cluster 953
0     44aa, >anti-Gram+;antibact... *
>Cluster 954
0     24aa, >antimicrobial... at 41.67%
1     44aa, >anti-Gram+;antibact... *
>Cluster 955
0     44aa, >anti-Gram+;Antibact... *
1     38aa, >anti-Gram+;Antibact... at 55.26%
>Cluster 956
0     39aa, >antibacterial;antim... at 64.10%
1     44aa, >antibacterial... *
2     15aa, >antibacterial10170... at 66.67%
>Cluster 957
0     44aa, >Antimicrobial... *
>Cluster 958
0     44aa, >Antimicrobial... *
1     44aa, >antibacterial;antim... at 54.55%
2     44aa, >Antibacterial... at 54.55%
3     44aa, >Antimicrobial... at 70.45%
>Cluster 959
0     44aa, >Antimicrobial... *
1     44aa, >Antimicrobial... at 45.45%
>Cluster 960
0     44aa, >Antimicrobial... *
>Cluster 961
0     44aa, >antimicrobial... *
>Cluster 962
0     44aa, >Antimicrobial... *
>Cluster 963
```

0   37aa, >anti-Gram+;antibact... at 48.65%
1   44aa, >anti-Gram+;Antibact... *
>Cluster 964
0   44aa, >anti-Gram+;antibact... *
1   35aa, >antibacterial12643... at 45.71%
2   35aa, >antibacterial12668... at 48.57%
>Cluster 965
0   40aa, >antibacterial;antim... at 62.50%
1   24aa, >antibacterial;Antif... at 66.67%
2   24aa, >anti-Gram+;Antibact... at 66.67%
3   34aa, >antimicrobial... at 41.18%
4   24aa, >anti-Gram+;Antibact... *
5   27aa, >antimicrobial... at 44.44%
6   44aa, >Antimicrobial... *
7   44aa, >Antimicrobial... at 77.27%
8   44aa, >Antimicrobial... at 75.00%
9   44aa, >Antimicrobial... at 86.36%
10   44aa, >Antimicrobial... at 72.73%
11   44aa, >Antimicrobial... at 72.73%
12   44aa, >Antimicrobial... at 79.55%
13   44aa, >Antimicrobial... at 86.36%
14   44aa, >Antimicrobial... at 86.36%
15   24aa, >antibacterial... at 62.50%
16   21aa, >antibacterial... *
17   13aa, >antibacterial... at 46.15%
>Cluster 966
0   44aa, >Antimicrobial... *
>Cluster 967
0   41aa, >antibacterial;Antif... at 70.73%
1   44aa, >antimicrobial... *
2   44aa, >antimicrobial... at 56.82%
3   44aa, >antimicrobial... at 56.82%
4   44aa, >antimicrobial... at 75.00%
>Cluster 968
0   44aa, >antibacterial10882... *
>Cluster 969
0   23aa, >Antimicrobial... at 43.48%
1   41aa, >antibacterial11553... at 78.05%
2   42aa, >antibacterial11554... at 66.67%
3   43aa, >antibacterial11589... at 60.47%
4   44aa, >antibacterial11590... *
>Cluster 970
0   41aa, >antibacterial11421... at 53.66%
1   35aa, >antibacterial12716... at 35.00%
2   28aa, >antibacterial12765... at 46.43%
3   44aa, >antibacterial12783... *
>Cluster 971
0   43aa, >antimicrobial;antiv... *
1   30aa, >antibacterial13122... at 35.00%
>Cluster 972
0   43aa, >anti-Gram+;antimicr... *
>Cluster 973
0   43aa, >antibacterial;Antif... *
1   18aa, >antibacterial12921... at 44.44%

```
>Cluster 974
0	43aa, >Antimicrobial... *
>Cluster 975
0	43aa, >antibacterial;antim... *
>Cluster 976
0	43aa, >Antimicrobial... *
1	20aa, >antibacterial09117... at 35.00%
>Cluster 977
0	43aa, >antimicrobial... *
>Cluster 978
0	43aa, >anti-Gram+;antibact... *
1	27aa, >antibacterial08429... at 55.56%
>Cluster 979
0	28aa, >anti-Gram-;Antibact... at 42.86%
1	43aa, >Antimicrobial... *
>Cluster 980
0	43aa, >Antibacterial;Antim... *
>Cluster 981
0	43aa, >Antimicrobial... *
>Cluster 982
0	40aa, >antibacterial;antif... at 70.00%
1	43aa, >antibacterial;antim... *
>Cluster 983
0	43aa, >anti-Gram+;antibact... *
1	11aa, >antibacterial08110... at 45.45%
>Cluster 984
0	43aa, >Antimicrobial... *
1	43aa, >Antibacterial... at 76.74%
2	43aa, >antibacterial10829... at 67.44%
>Cluster 985
0	42aa, >Antibacterial;antim... at 42.86%
1	39aa, >antimicrobial... at 41.03%
2	39aa, >antimicrobial... at 46.15%
3	39aa, >antimicrobial... at 41.03%
4	43aa, >anti-Gram-... *
5	37aa, >antibacterial07527... at 40.54%
>Cluster 986
0	43aa, >antibacterial;antim... *
>Cluster 987
0	43aa, >antimicrobial... *
>Cluster 988
0	43aa, >antimicrobial... *
1	41aa, >antimicrobial... at 83.02%
>Cluster 989
0	40aa, >antibacterial06621... at 65.00%
1	43aa, >antibacterial06622... *
2	32aa, >antibacterial06643... at 68.75%
3	38aa, >antibacterial06656... at 57.89%
4	28aa, >antibacterial06686... at 46.43%
5	41aa, >antibacterial07315... at 68.29%
6	40aa, >antibacterial07316... at 62.50%
7	40aa, >antibacterial07317... at 70.00%
8	31aa, >antibacterial07429... at 41.94%
9	20aa, >antibacterial10124... at 45.00%
```

>Cluster 990
0 43aa, >antibacterial09662... *
1 43aa, >antibacterial09663... at 72.09%
>Cluster 991
0 43aa, >antibacterial10677... *
>Cluster 992
0 43aa, >antibacterial13063... *
>Cluster 993
0 43aa, >antibacterial13208... *
>Cluster 994
0 42aa, >anti-Gram+;Antibact... *
>Cluster 995
0 42aa, >anti-Gram-;antibact... *
>Cluster 996
0 42aa, >antimicrobial... *
>Cluster 997
0 42aa, >antimicrobial... *
>Cluster 998
0 42aa, >Antimicrobial... *
>Cluster 999
0 42aa, >anti-Gram+... *
>Cluster 1000
0 38aa, >antimicrobial;Antim... at 86.47%
1 42aa, >antimicrobial... *
>Cluster 1001
0 42aa, >anti-Gram+;antibact... *
>Cluster 1002
0 42aa, >antimicrobial... *
>Cluster 1003
0 42aa, >Antimicrobial... *
>Cluster 1004
0 42aa, >Antimicrobial... *
1 37aa, >Antimicrobial... at 54.05%
>Cluster 1005
0 42aa, >antimicrobial... *
>Cluster 1006
0 24aa, >antibacterial08139... at 41.67%
1 42aa, >antibacterial08239... *
2 26aa, >antibacterial09223... at 57.69%
3 32aa, >antibacterial09224... at 56.25%
4 31aa, >antibacterial09834... at 58.06%
>Cluster 1007
0 42aa, >antibacterial09657... *
>Cluster 1008
0 35aa, >antibacterial09871... at 62.86%
1 29aa, >antibacterial11547... at 48.28%
2 42aa, >antibacterial11763... *
3 35aa, >antibacterial12026... at 45.71%
4 36aa, >antibacterial13339... at 52.78%
>Cluster 1009
0 39aa, >antibacterial11447... at 48.72%
1 42aa, >antibacterial12747... *
2 42aa, >antibacterial12748... at 57.14%
3 42aa, >antibacterial12753... at 52.38%

4   42aa, >antibacterial12758... at 47.62%
5   42aa, >antibacterial12759... at 57.14%
6   42aa, >antibacterial12768... at 52.38%
7   28aa, >antibacterial12778... at 42.86%
8   42aa, >antibacterial12781... at 52.38%
9   42aa, >antibacterial12785... at 57.14%
>Cluster 1010
0   36aa, >antibacterial12406... at 78.18%
1   42aa, >antibacterial12786... *
>Cluster 1011
0   41aa, >Antibacterial;Antif... *
>Cluster 1012
0   41aa, >antibacterial;antim... *
1   29aa, >antibacterial11821... at 71.31%
>Cluster 1013
0   41aa, >Antimicrobial... *
>Cluster 1014
0   41aa, >antimicrobial... *
>Cluster 1015
0   41aa, >Antimicrobial... *
>Cluster 1016
0   41aa, >Antimicrobial... *
1   41aa, >Antimicrobial... at 87.80%
>Cluster 1017
0   41aa, >antibacterial07313... *
1   30aa, >antibacterial13257... at 35.00%
>Cluster 1018
0   28aa, >antimicrobial... at 46.43%
1   29aa, >antimicrobial... at 41.38%
2   23aa, >anti-Gram+;antibact... at 56.52%
3   30aa, >antibacterial;antim... at 35.00%
4   41aa, >antibacterial11949... *
>Cluster 1019
0   41aa, >antibacterial11960... *
>Cluster 1020
0   26aa, >antibacterial08597... at 42.31%
1   41aa, >antibacterial12877... *
>Cluster 1021
0   18aa, >antibacterial07970... at 44.44%
1   41aa, >antibacterial13012... *
>Cluster 1022
0   20aa, >anti-Gram+;antibact... at 50.00%
1   24aa, >antibacterial08142... at 45.83%
2   33aa, >antibacterial11401... at 57.58%
3   33aa, >antibacterial11402... at 72.73%
4   30aa, >antibacterial11403... at 66.67%
5   41aa, >antibacterial13279... *
>Cluster 1023
0   41aa, >antibacterial13314... *
>Cluster 1024
0   40aa, >anti-Gram+;antibact... *
1   18aa, >antimicrobial... at 44.44%
>Cluster 1025
0   39aa, >antifungal;antimicr... at 71.79%

1   40aa, >antifungal;antimicr... *
>Cluster 1026
0   40aa, >anti-Gram+;antibact... *
1   42aa, >antibacterial08978... at 86.50%
>Cluster 1027
0   40aa, >Antimicrobial... *
>Cluster 1028
0   40aa, >Antimicrobial... *
1   18aa, >antibacterial09721... at 44.44%
>Cluster 1029
0   40aa, >antibacterial06670... *
>Cluster 1030
0   40aa, >antibacterial06888... *
>Cluster 1031
0   40aa, >antibacterial10952... *
>Cluster 1032
0   26aa, >antibacterial09054... at 42.31%
1   26aa, >antibacterial12455... at 42.31%
2   40aa, >antibacterial12927... *
>Cluster 1033
0   34aa, >antibacterial11907... at 76.47%
1   36aa, >antibacterial12002... at 61.11%
2   40aa, >antibacterial13421... *
>Cluster 1034
0   39aa, >Antibacterial;Antif... *
1   27aa, >antibacterial08929... at 40.74%
>Cluster 1035
0   39aa, >antimicrobial... *
1   33aa, >antimicrobial... at 81.82%
2   34aa, >antimicrobial... at 73.53%
3   35aa, >antimicrobial... at 77.14%
>Cluster 1036
0   39aa, >Antibacterial;Antim... *
>Cluster 1037
0   39aa, >anti-Gram+;Antibact... *
1   23aa, >antibacterial11803... at 43.48%
>Cluster 1038
0   39aa, >antibacterial;antim... *
>Cluster 1039
0   39aa, >anti-Gram+;antibact... *
>Cluster 1040
0   39aa, >antimicrobial... *
1   39aa, >antimicrobial... at 46.15%
>Cluster 1041
0   39aa, >Antimicrobial... *
>Cluster 1042
0   39aa, >Antimicrobial... *
>Cluster 1043
0   39aa, >antibacterial08491... *
1   21aa, >antibacterial10077... at 80.48%
2   23aa, >antibacterial11917... at 61.30%
>Cluster 1044
0   23aa, >antimicrobial... at 43.48%
1   37aa, >antibacterial07310... at 43.24%

```
2     37aa, >antibacterial07374... at 43.24%
3     37aa, >antibacterial07375... at 45.95%
4     25aa, >antibacterial08628... at 35.00%
5     39aa, >antibacterial09062... *
>Cluster 1045
0     39aa, >antibacterial11503... *
>Cluster 1046
0     31aa, >Antibacterial;antim... at 41.94%
1     28aa, >Antibacterial;Antif... at 50.00%
2     33aa, >anti-Gram-;antibact... at 42.42%
3     37aa, >antibacterial;antim... at 40.54%
4     33aa, >antimicrobial... at 69.70%
5     12aa, >antibacterial10140... at 41.67%
6     28aa, >antibacterial11864... at 42.86%
7     39aa, >antibacterial11885... *
>Cluster 1047
0     32aa, >antibacterial07344... at 50.00%
1     39aa, >antibacterial12929... *
>Cluster 1048
0     38aa, >Antimicrobial... *
>Cluster 1049
0     38aa, >antibacterial... *
>Cluster 1050
0     28aa, >Antibacterial;antim... at 42.86%
1     38aa, >antimicrobial... *
>Cluster 1051
0     38aa, >Antifungal;antimicr... *
>Cluster 1052
0     31aa, >Anti-Gram-;Antibact... at 54.84%
1     33aa, >anti-Gram+;antibact... at 42.42%
2     33aa, >anti-Gram+;antibact... at 84.85%
3     28aa, >antibacterial;antim... at 46.43%
4     37aa, >Antimicrobial... at 67.57%
5     38aa, >anti-Gram+;antifung... *
6     28aa, >antibacterial;antim... at 42.86%
7     38aa, >antibacterial11866... at 63.16%
>Cluster 1053
0     38aa, >Antimicrobial... *
1     36aa, >antibacterial06542... at 86.11%
2     13aa, >antibacterial07567... at 53.85%
3     35aa, >antibacterial09294... at 35.00%
>Cluster 1054
0     38aa, >antimicrobial... *
1     38aa, >antimicrobial... at 81.58%
2     38aa, >antimicrobial... at 86.84%
3     38aa, >antimicrobial... at 73.68%
4     38aa, >antimicrobial... at 65.79%
5     38aa, >antimicrobial... at 74.21%
6     38aa, >antimicrobial... at 72.47%
7     38aa, >antimicrobial... at 73.68%
8     38aa, >antimicrobial... at 86.84%
9     38aa, >antimicrobial... at 71.58%
10    38aa, >antimicrobial... at 82.47%
11    39aa, >antimicrobial... at 82.47%
```

12   39aa, >antimicrobial... at 84.21%
13   39aa, >antimicrobial... at 58.95%
14   27aa, >antibacterial08930... at 40.74%
>Cluster 1055
0   36aa, >antimicrobial... at 47.22%
1   38aa, >antimicrobial... *
>Cluster 1056
0   38aa, >Antimicrobial... *
>Cluster 1057
0   38aa, >antimicrobial... *
>Cluster 1058
0   33aa, >antibacterial;antim... at 42.42%
1   38aa, >Antimicrobial... *
2   37aa, >Antimicrobial... at 43.24%
>Cluster 1059
0   38aa, >Antifungal;antimicr... *
>Cluster 1060
0   38aa, >antifungal;antimicr... *
1   38aa, >antifungal;antimicr... at 65.79%
>Cluster 1061
0   38aa, >antibacterial08236... *
>Cluster 1062
0   28aa, >anti-Gram+;antibact... at 67.86%
1   28aa, >antimicrobial... at 75.00%
2   38aa, >antibacterial08421... *
>Cluster 1063
0   38aa, >antibacterial10725... *
>Cluster 1064
0   14aa, >antibacterial08766... at 42.86%
1   14aa, >antibacterial08767... at 42.86%
2   14aa, >antibacterial08768... at 50.00%
3   14aa, >antibacterial08769... at 42.86%
4   15aa, >antibacterial08770... at 42.86%
5   15aa, >antibacterial08771... at 42.86%
6   15aa, >antibacterial08772... at 42.86%
7   15aa, >antibacterial08773... at 50.00%
8   15aa, >antibacterial08774... at 50.00%
9   38aa, >antibacterial10797... *
>Cluster 1065
0   37aa, >anti-Gram-;antibact... at 51.35%
1   37aa, >antibacterial07899... at 64.86%
2   38aa, >antibacterial10798... *
>Cluster 1066
0   23aa, >antibacterial07927... at 43.48%
1   27aa, >antibacterial11098... at 40.74%
2   25aa, >antibacterial11961... at 84.00%
3   38aa, >antibacterial13379... *
>Cluster 1067
0   35aa, >antibacterial;antif... at 54.29%
1   34aa, >Antimicrobial... at 70.59%
2   34aa, >Antimicrobial... at 71.18%
3   37aa, >Antimicrobial... *
4   37aa, >Antimicrobial... at 84.19%
5   37aa, >Antimicrobial... at 81.89%

6 37aa, >Antimicrobial... at 72.97%
7 37aa, >Antimicrobial... at 84.19%
8 37aa, >Antimicrobial... at 44.05%
9 37aa, >Antimicrobial... at 41.35%
10 23aa, >Antibacterial;antim... at 33.48%
>Cluster 1068
0 37aa, >anti-Gram-;antifung... *
>Cluster 1069
0 30aa, >anti-Gram+;antibact... at 46.67%
1 30aa, >antibacterial;antim... at 60.00%
2 34aa, >Antifungal;Antimicr... at 58.82%
3 37aa, >antibacterial;antim... *
4 33aa, >antimicrobial... at 63.64%
5 33aa, >anti-Gram+;Antibact... at 42.42%
6 33aa, >anti-Gram-;antibact... at 45.45%
7 33aa, >anti-Gram+;antibact... at 66.67%
8 33aa, >anti-Gram+;antibact... at 60.61%
9 33aa, >anti-Gram+;Antibact... at 48.48%
10 33aa, >anti-Gram+;Antibact... at 51.52%
11 33aa, >antimicrobial... at 48.48%
12 33aa, >Antimicrobial... at 54.55%
13 33aa, >antimicrobial... at 48.48%
14 33aa, >Antimicrobial... at 66.67%
15 31aa, >antimicrobial... at 45.16%
16 31aa, >antimicrobial... at 45.16%
17 33aa, >anti-Gram+;Antibact... at 51.52%
18 33aa, >antimicrobial... at 42.42%
19 33aa, >Antimicrobial... at 42.42%
20 33aa, >anti-Gram+;antifung... at 42.42%
21 33aa, >antimicrobial... at 48.48%
22 35aa, >antimicrobial... at 42.86%
23 33aa, >antimicrobial... at 42.42%
24 33aa, >anti-Gram+;Antibact... at 45.45%
25 30aa, >antibacterial11753... at 46.67%
26 33aa, >antibacterial11778... at 63.64%
27 33aa, >antibacterial11819... at 66.67%
28 33aa, >antibacterial11822... at 69.70%
29 33aa, >antibacterial11862... at 63.64%
30 34aa, >antibacterial13017... at 50.00%
>Cluster 1070
0 27aa, >anti-Gram+;antibact... at 44.44%
1 37aa, >antibacterial;antim... *
>Cluster 1071
0 37aa, >Antibacterial... *
1 37aa, >Antibacterial;Antif... at 86.19%
2 37aa, >antibacterial10586... at 81.08%
3 37aa, >antibacterial10587... at 86.19%
>Cluster 1072
0 37aa, >antibacterial06503... *
>Cluster 1073
0 37aa, >antibacterial08020... *
>Cluster 1074
0 37aa, >antibacterial11749... *
1 36aa, >antibacterial12025... at 80.56%

>Cluster 1075
0   23aa, >antifungal;antimicr... at 65.22%
1   36aa, >antifungal;antimicr... *
2   23aa, >antifungal;antimicr... at 47.83%
>Cluster 1076
0   36aa, >antibacterial;antim... *
>Cluster 1077
0   24aa, >Antibacterial;Antif... at 45.83%
1   36aa, >antifungal... *
>Cluster 1078
0   36aa, >antimicrobial... *
>Cluster 1079
0   23aa, >antimicrobial... at 43.48%
1   36aa, >antibacterial07267... *
>Cluster 1080
0   36aa, >antibacterial07610... *
1   36aa, >antibacterial07611... at 69.44%
2   36aa, >antibacterial09476... at 75.00%
3   26aa, >antibacterial10295... at 65.38%
>Cluster 1081
0   33aa, >antibacterial08264... at 66.67%
1   36aa, >antibacterial08611... *
>Cluster 1082
0   36aa, >antibacterial09011... *
>Cluster 1083
0   35aa, >antibacterial11603... at 62.86%
1   36aa, >antibacterial11750... *
2   35aa, >antibacterial12108... at 51.43%
3   35aa, >antibacterial12506... at 54.29%
4   35aa, >antibacterial12704... at 71.43%
>Cluster 1084
0   36aa, >antibacterial12114... *
1   35aa, >antibacterial12497... at 45.71%
>Cluster 1085
0   35aa, >antibacterial... at 57.14%
1   36aa, >antibacterial12173... *
>Cluster 1086
0   20aa, >antibacterial09123... at 35.00%
1   32aa, >antibacterial09904... at 43.75%
2   36aa, >antibacterial12608... *
>Cluster 1087
0   33aa, >antibacterial11764... at 57.58%
1   33aa, >antibacterial11766... at 51.52%
2   16aa, >antibacterial12373... at 68.75%
3   36aa, >antibacterial12941... *
>Cluster 1088
0   36aa, >antibacterial13109... *
>Cluster 1089
0   36aa, >antibacterial13331... *
>Cluster 1090
0   36aa, >antibacterial13400... *
>Cluster 1091
0   36aa, >antibacterial13420... *
>Cluster 1092

```
0     35aa, >antibacterial;antif... *
1     19aa, >antibacterial10189... at 42.11%
>Cluster 1093
0     35aa, >Antimicrobial... *
>Cluster 1094
0     35aa, >anti-Gram+;Gram-... *
>Cluster 1095
0     32aa, >anti-Gram+;antibact... at 50.25%
1     32aa, >antibacterial;antim... at 70.62%
2     35aa, >antibacterial;antim... *
3     29aa, >antibacterial;antif... at 86.55%
4     35aa, >anti-Gram+;antibact... at 81.43%
>Cluster 1096
0     35aa, >antibacterial06455... *
1     35aa, >antibacterial06457... at 62.86%
2     35aa, >antibacterial06481... at 45.71%
3     35aa, >antibacterial06937... at 71.43%
4     18aa, >antibacterial08619... at 44.44%
>Cluster 1097
0     35aa, >antibacterial07305... *
1     33aa, >antibacterial07306... at 72.73%
>Cluster 1098
0     23aa, >antibacterial08728... at 43.48%
1     35aa, >antibacterial08882... *
2     23aa, >antibacterial09059... at 43.48%
>Cluster 1099
0     35aa, >antibacterial10961... *
>Cluster 1100
0     35aa, >antibacterial11569... *
1     35aa, >antibacterial12107... at 74.29%
2     35aa, >antibacterial13187... at 65.71%
>Cluster 1101
0     34aa, >antibacterial;Antif... *
1     26aa, >antibacterial07761... at 50.00%
2     26aa, >antibacterial11154... at 50.00%
>Cluster 1102
0     34aa, >anti-Gram-... *
1     34aa, >antibacterial08261... at 41.18%
>Cluster 1103
0     34aa, >antibacterial;antim... *
>Cluster 1104
0     34aa, >anti-Gram+;antibact... *
>Cluster 1105
0     11aa, >antibacterial07864... at 54.55%
1     34aa, >antibacterial07878... *
2     19aa, >antibacterial07890... at 52.63%
3     33aa, >antibacterial08002... at 57.58%
4     11aa, >antibacterial09190... at 45.45%
5     14aa, >antibacterial09262... at 42.86%
6     15aa, >antibacterial09648... at 46.67%
7     15aa, >antibacterial09649... at 53.33%
8     15aa, >antibacterial09650... at 35.00%
9     15aa, >antibacterial09651... at 46.67%
10    15aa, >antibacterial09652... at 46.67%
```

11   15aa, >antibacterial09653... at 60.00%
12   19aa, >antibacterial09699... at 42.11%
13   15aa, >antibacterial09921... at 35.00%
14   20aa, >antibacterial09935... at 35.00%
15   19aa, >antibacterial09938... at 57.89%
16   20aa, >antibacterial10325... at 45.00%
17   20aa, >antibacterial10623... at 45.00%
>Cluster 1106
0   18aa, >antibacterial07971... at 44.44%
1   32aa, >antibacterial08552... at 56.25%
2   34aa, >antibacterial08555... *
3   28aa, >antibacterial08556... at 67.86%
4   28aa, >antibacterial08557... at 46.43%
>Cluster 1107
0   34aa, >antibacterial11504... *
>Cluster 1108
0   34aa, >antibacterial12646... *
>Cluster 1109
0   34aa, >antibacterial12671... *
>Cluster 1110
0   34aa, >antibacterial13373... *
>Cluster 1111
0   33aa, >antibacterial;antim... *
1   30aa, >anti-Gram+;antibact... at 43.33%
>Cluster 1112
0   33aa, >antibacterial... *
1   33aa, >antibacterial... at 63.64%
2   33aa, >antibacterial... at 66.67%
>Cluster 1113
0   33aa, >Antifungal... *
>Cluster 1114
0   33aa, >antibacterial;antim... *
>Cluster 1115
0   33aa, >antibacterial;antim... *
>Cluster 1116
0   33aa, >antifungal;antimicr... *
1   18aa, >antimicrobial... at 44.44%
>Cluster 1117
0   33aa, >antimicrobial... *
1   33aa, >anti-Gram+;Antibact... at 72.73%
>Cluster 1118
0   32aa, >Gram-... at 50.00%
1   33aa, >Antifungal;Gram-... *
2   32aa, >antibacterial08463... at 43.75%
>Cluster 1119
0   33aa, >antibacterial07851... *
1   30aa, >antibacterial... at 83.55%
>Cluster 1120
0   33aa, >antibacterial08019... *
>Cluster 1121
0   33aa, >antibacterial08233... *
>Cluster 1122
0   33aa, >antibacterial08548... *
>Cluster 1123

0 33aa, >antibacterial09903... *
>Cluster 1124
0 33aa, >antibacterial10870... *
>Cluster 1125
0 33aa, >antibacterial10876... *
1 29aa, >antibacterial1... at 79.31%
>Cluster 1126
0 33aa, >antibacterial11312... *
>Cluster 1127
0 33aa, >antibacterial11765... *
>Cluster 1128
0 33aa, >antibacterial11768... *
>Cluster 1129
0 33aa, >antibacterial12139... *
>Cluster 1130
0 33aa, >antibacterial12594... *
>Cluster 1131
0 33aa, >antibacterial12833... *
>Cluster 1132
0 23aa, >Antimicrobial... at 86.96%
1 23aa, >Anti-Gram-... at 69.57%
2 18aa, >Antimicrobial... at 83.33%
3 23aa, >antibacterial... *
4 23aa, >antibacterial... at 43.48%
5 22aa, >antibacterial... at 82.61%
6 28aa, >antibacterial... at 65.22%
7 23aa, >antibacterial... at 60.87%
8 23aa, >antibacterial... at 56.52%
9 23aa, >antibacterial... at 56.52%
10 24aa, >antibacterial... at 82.61%
11 23aa, >antibacterial... at 65.22%
12 33aa, >antibacterial... *
>Cluster 1133
0 32aa, >antimicrobial... *
1 32aa, >antibacterial;antim... at 40.62%
>Cluster 1134
0 32aa, >antibacterial;Antif... *
>Cluster 1135
0 32aa, >antimicrobial... *
1 13aa, >antibacterial07603... at 46.15%
>Cluster 1136
0 32aa, >antimicrobial... *
>Cluster 1137
0 32aa, >antimicrobial... *
>Cluster 1138
0 32aa, >antimicrobial... *
>Cluster 1139
0 32aa, >antimicrobial... *
>Cluster 1140
0 32aa, >antibacterial... *
>Cluster 1141
0 32aa, >antibacterial... *
>Cluster 1142
0 32aa, >antibacterial... *

```
>Cluster 1143
0     31aa, >antibacterial;antim... *
1     18aa, >antibacterial... at 44.44%
>Cluster 1144
0     28aa, >antifungal;antimicr... at 42.86%
1     31aa, >anti-Gram+;Antifung... *
2     31aa, >antifungal;antimicr... at 51.61%
>Cluster 1145
0     31aa, >antibacterial;antith... *
>Cluster 1146
0     28aa, >anti-Gram-;Antibact... at 46.43%
1     31aa, >anti-Gram+;antimicr... *
2     31aa, >antimicrobial... at 67.74%
3     31aa, >antibacterial09003... at 61.29%
4     31aa, >antibacterial09004... at 54.84%
>Cluster 1147
0     31aa, >antimicrobial... *
1     31aa, >antibacterial;antim... at 83.55%
2     19aa, >antibacterial09498... at 42.11%
>Cluster 1148
0     31aa, >antimicrobial... *
1     30aa, >antimicrobial... at 83.55%
>Cluster 1149
0     31aa, >antimicrobial... *
>Cluster 1150
0     31aa, >antibacterial06665... *
1     25aa, >antibacterial06960... at 73.10%
2     29aa, >antibacterial09876... at 80.10%
>Cluster 1151
0     31aa, >antibacterial08018... *
>Cluster 1152
0     31aa, >antibacterial08066... *
>Cluster 1153
0     31aa, >antibacterial10933... *
>Cluster 1154
0     31aa, >antibacterial13150... *
>Cluster 1155
0     13aa, >antibacterial06600... at 46.15%
1     23aa, >antibacterial09226... at 47.83%
2     28aa, >antibacterial09481... at 60.71%
3     21aa, >antibacterial09525... at 47.62%
4     31aa, >antibacterial13182... *
>Cluster 1156
0     31aa, >antibacterial13324... *
>Cluster 1157
0     30aa, >antibacterial09773... *
>Cluster 1158
0     30aa, >antibacterial09852... *
>Cluster 1159
0     24aa, >antibacterial10362... at 41.67%
1     30aa, >antibacterial11377... *
>Cluster 1160
0     30aa, >antibacterial12793... *
>Cluster 1161
```

0   30aa, >antibacterial13318... *
>Cluster 1162
0   29aa, >Antimicrobial... *
1   29aa, >Antimicrobial... at 41.38%
2   28aa, >anti-Gram-;antibact... at 46.43%
3   29aa, >Antimicrobial... at 44.83%
>Cluster 1163
0   29aa, >anti-Gram+;Antibact... *
1   18aa, >Antimicrobial... at 44.44%
>Cluster 1164
0   29aa, >antimicrobial... *
>Cluster 1165
0   29aa, >antimicrobial... *
>Cluster 1166
0   29aa, >anti-Gram+;antifung... *
1   18aa, >antibacterial09675... at 44.44%
>Cluster 1167
0   29aa, >anti-Gram+;antifung... *
>Cluster 1168
0   29aa, >anti-Gram+;Antibact... *
1   26aa, >antibacterial10275... at 53.85%
>Cluster 1169
0   29aa, >antibacterial;Antif... *
1   29aa, >antibacterial13028... at 82.76%
2   26aa, >antibacterial13029... at 73.10%
>Cluster 1170
0   29aa, >antibacterial07366... *
>Cluster 1171
0   14aa, >antibacterial07583... at 42.86%
1   23aa, >antibacterial07584... at 47.83%
2   29aa, >antibacterial07880... *
3   19aa, >antibacterial07891... at 52.63%
4   29aa, >antibacterial07892... at 79.31%
5   29aa, >antibacterial07893... at 58.62%
6   29aa, >antibacterial07894... at 72.41%
7   22aa, >antibacterial08433... at 45.45%
8   15aa, >antibacterial09647... at 35.00%
9   19aa, >antibacterial09691... at 42.11%
10   29aa, >antibacterial09915... at 68.97%
11   21aa, >antibacterial09916... at 71.43%
12   19aa, >antibacterial09936... at 68.42%
13   19aa, >antibacterial09937... at 63.16%
14   21aa, >antibacterial09968... at 57.14%
15   21aa, >antibacterial09969... at 52.38%
16   19aa, >antibacterial09978... at 57.89%
17   25aa, >antibacterial09983... at 76.00%
18   28aa, >antibacterial09984... at 67.86%
19   25aa, >antibacterial09985... at 56.00%
20   28aa, >antibacterial09986... at 60.71%
21   28aa, >antibacterial09987... at 67.86%
22   14aa, >antibacterial10190... at 42.86%
>Cluster 1172
0   29aa, >antibacterial11265... *
>Cluster 1173

0 29aa, >antibacterial11455... *
>Cluster 1174
0 29aa, >antibacterial12754... *
>Cluster 1175
0 18aa, >antibacterial12669... at 44.44%
1 29aa, >antibacterial13107... *
>Cluster 1176
0 28aa, >anti-Gram+;antifung... *
1 27aa, >antimicrobial... at 82.86%
>Cluster 1177
0 28aa, >antimicrobial... *
>Cluster 1178
0 28aa, >antimicrobial... *
>Cluster 1179
0 28aa, >antibacterial;antim... *
1 28aa, >antibacterial;antim... at 82.86%
2 29aa, >Antibacterial;antim... at 85.71%
>Cluster 1180
0 28aa, >antibacterial06499... *
1 27aa, >antibacterial06704... at 77.78%
2 27aa, >antibacterial06706... at 81.48%
3 28aa, >antibacterial06955... at 85.71%
4 28aa, >antibacterial06956... at 85.71%
5 27aa, >antibacterial07085... at 85.19%
>Cluster 1181
0 21aa, >antibacterial06602... at 52.38%
1 18aa, >antibacterial06613... at 77.78%
2 21aa, >antibacterial06616... at 61.90%
3 28aa, >antibacterial06617... *
4 16aa, >antibacterial06998... at 56.25%
5 13aa, >antibacterial07573... at 46.15%
6 13aa, >antibacterial08026... at 61.54%
7 13aa, >antibacterial09599... at 46.15%
>Cluster 1182
0 28aa, >antibacterial06673... *
1 28aa, >antibacterial07424... at 75.00%
2 28aa, >antibacterial07425... at 75.00%
3 28aa, >antibacterial07428... at 67.86%
4 28aa, >antibacterial07430... at 67.86%
>Cluster 1183
0 28aa, >antibacterial06771... *
>Cluster 1184
0 28aa, >antibacterial07067... *
1 29aa, >antibacterial07068... at 82.86%
2 29aa, >antibacterial07070... at 79.86%
>Cluster 1185
0 28aa, >antibacterial07383... *
>Cluster 1186
0 23aa, >antibacterial07777... at 61.54%
1 28aa, >antibacterial07804... *
>Cluster 1187
0 28aa, >antibacterial08879... *
>Cluster 1188
0 26aa, >antibacterial09204... at 53.85%

```
1     19aa, >antibacterial09214... at 47.37%
2     28aa, >antibacterial09477... *
3     28aa, >antibacterial09482... at 50.00%
4     19aa, >antibacterial09503... at 42.11%
5     21aa, >antibacterial09510... at 52.38%
6     21aa, >antibacterial09514... at 46.67%
7     22aa, >antibacterial09524... at 35.45%
8     26aa, >antibacterial10250... at 38.31%
9     19aa, >antibacterial11647... at 32.11%
>Cluster 1189
0     25aa, >antibacterial;antim... at 35.00%
1     28aa, >antibacterial10254... *
>Cluster 1190
0     28aa, >antibacterial10261... *
>Cluster 1191
0     13aa, >antibacterial10427... at 46.15%
1     28aa, >antibacterial10743... *
>Cluster 1192
0     28aa, >antibacterial10888... *
>Cluster 1193
0     28aa, >antibacterial11386... *
1     25aa, >antibacterial13149... at 48.00%
>Cluster 1194
0     28aa, >antibacterial11507... *
1     28aa, >antibacterial11508... at 78.57%
>Cluster 1195
0     28aa, >antibacterial11921... *
>Cluster 1196
0     28aa, >antibacterial11963... *
>Cluster 1197
0     28aa, >antibacterial11999... *
>Cluster 1198
0     28aa, >antibacterial12751... *
1     28aa, >antibacterial12782... at 42.86%
>Cluster 1199
0     28aa, >antibacterial12762... *
1     28aa, >antibacterial12773... at 46.43%
>Cluster 1200
0     28aa, >antibacterial12770... *
>Cluster 1201
0     28aa, >antibacterial12784... *
>Cluster 1202
0     28aa, >antibacterial12906... *
>Cluster 1203
0     28aa, >antibacterial12975... *
>Cluster 1204
0     28aa, >antibacterial12995... *
>Cluster 1205
0     27aa, >Antifungal... *
>Cluster 1206
0     27aa, >antifungal;antimicr... *
>Cluster 1207
0     27aa, >antimicrobial... *
>Cluster 1208
```

0   27aa, >antibacterial07072... *
1   25aa, >antibacterial07380... at 48.00%
>Cluster 1209
0   27aa, >antibacterial07450... *
>Cluster 1210
0   27aa, >antibacterial09755... *
1   27aa, >antibacterial09756... at 74.07%
2   27aa, >antibacterial09757... at 74.07%
3   27aa, >antibacterial09758... at 66.67%
>Cluster 1211
0   27aa, >antibacterial13321... *
>Cluster 1212
0   26aa, >antimicrobial... *
>Cluster 1213
0   26aa, >anti-Gram-;antibact... *
>Cluster 1214
0   26aa, >anti-Gram-;antibact... *
>Cluster 1215
0   26aa, >antimicrobial... *
>Cluster 1216
0   26aa, >antibacterial;antim... *
>Cluster 1217
0   26aa, >antibacterial;antim... *
1   26aa, >antibacterial;antim... at 65.38%
2   26aa, >antibacterial09733... at 61.54%
>Cluster 1218
0   26aa, >antimicrobial... *
>Cluster 1219
0   26aa, >anti-Gram+;antibact... *
1   14aa, >antibacterial08745... at 42.86%
>Cluster 1220
0   26aa, >antimicrobial... *
>Cluster 1221
0   26aa, >antimicrobial... *
>Cluster 1222
0   26aa, >Antimicrobial... *
>Cluster 1223
0   26aa, >antibacterial08073... *
>Cluster 1224
0   26aa, >antibacterial08403... *
>Cluster 1225
0   14aa, >antibacterial07582... at 42.86%
1   26aa, >antibacterial08598... *
2   26aa, >antibacterial09057... at 42.31%
3   26aa, >antibacterial09948... at 42.31%
>Cluster 1226
0   25aa, >Antimicrobial... at 38.00%
1   12aa, >antibacterial09017... at 41.67%
2   26aa, >antibacterial09053... *
3   26aa, >antibacterial09055... at 84.62%
4   12aa, >antibacterial09264... at 58.33%
5   13aa, >antibacterial09283... at 46.15%
6   15aa, >antibacterial09654... at 35.00%
7   26aa, >antibacterial10341... at 49.23%

```
>Cluster 1227
0	26aa, >antibacterial09801... *
>Cluster 1228
0	20aa, >antibacterial11321... at 50.00%
1	26aa, >antibacterial11374... *
>Cluster 1229
0	26aa, >antibacterial11399... *
>Cluster 1230
0	26aa, >antibacterial11446... *
>Cluster 1231
0	26aa, >antibacterial12439... *
>Cluster 1232
0	26aa, >antibacterial12641... *
>Cluster 1233
0	26aa, >antibacterial12908... *
>Cluster 1234
0	26aa, >antibacterial13317... *
>Cluster 1235
0	25aa, >Antifungal... *
>Cluster 1236
0	25aa, >antibacterial11450... *
>Cluster 1237
0	25aa, >antibacterial11464... *
>Cluster 1238
0	23aa, >Antimicrobial... at 78.26%
1	24aa, >antimicrobial... *
>Cluster 1239
0	24aa, >antimicrobial... *
>Cluster 1240
0	24aa, >antimicrobial... *
>Cluster 1241
0	23aa, >antibacterial... at 82.61%
1	24aa, >antibacterial06549... *
>Cluster 1242
0	23aa, >antifungal;antimicr... at 86.96%
1	24aa, >antibacterial10678... *
>Cluster 1243
0	23aa, >antibacterial06454... at 43.48%
1	24aa, >antibacterial12379... *
>Cluster 1244
0	23aa, >antibacterial07365... *
1	23aa, >antibacterial09212... at 81.30%
2	23aa, >antibacterial09213... at 81.30%
>Cluster 1245
0	23aa, >antibacterial07666... *
>Cluster 1246
0	23aa, >antibacterial08402... *
>Cluster 1247
0	23aa, >antibacterial12934... *
>Cluster 1248
0	23aa, >antibacterial13209... *
>Cluster 1249
0	22aa, >Antimicrobial... *
>Cluster 1250
```

0   21aa, >antimicrobial... *
>Cluster 1251
0   21aa, >anti-Gram+;Gram-... *
>Cluster 1252
0   21aa, >antibacterial08807... *
>Cluster 1253
0   21aa, >antibacterial08923... *
1   23aa, >antibacterial10290... at 85.71%
2   22aa, >antibacterial10292... at 85.71%
3   21aa, >antibacterial12991... at 85.45%
>Cluster 1254
0   21aa, >antibacterial09902... *
1   14aa, >antibacterial10322... at 50.00%
2   13aa, >antibacterial10429... at 46.15%
>Cluster 1255
0   21aa, >antibacterial10582... *
>Cluster 1256
0   21aa, >antibacterial10889... *
>Cluster 1257
0   21aa, >antibacterial12740... *
>Cluster 1258
0   14aa, >antibacterial08746... at 57.14%
1   12aa, >antibacterial09016... at 41.67%
2   15aa, >antibacterial09655... at 46.67%
3   19aa, >antibacterial09666... at 42.11%
4   19aa, >antibacterial09668... at 42.11%
5   19aa, >antibacterial09689... at 42.11%
6   19aa, >antibacterial09690... at 47.37%
7   19aa, >antibacterial09700... at 47.37%
8   11aa, >antibacterial09822... at 45.45%
9   16aa, >antibacterial10319... at 56.25%
10   15aa, >antibacterial10320... at 35.00%
11   19aa, >antibacterial10323... at 68.42%
12   18aa, >antibacterial10324... at 72.22%
13   20aa, >antibacterial10329... *
14   13aa, >antibacterial10692... at 46.15%
>Cluster 1259
0   19aa, >antibacterial07642... *
1   19aa, >antibacterial07643... at 68.42%
2   19aa, >antibacterial07644... at 63.16%
3   19aa, >antibacterial07645... at 78.95%
4   19aa, >antibacterial09671... at 57.89%
5   19aa, >antibacterial09697... at 42.11%
6   15aa, >antibacterial09701... at 35.00%
7   14aa, >antibacterial09702... at 42.86%
8   13aa, >antibacterial09703... at 46.15%
>Cluster 1260
0   19aa, >antibacterial08387... *
>Cluster 1261
0   18aa, >anti-Gram-;antibact... *
>Cluster 1262
0   18aa, >anti-Gram+;antibact... *
>Cluster 1263
0   18aa, >antibacterial09328... *

```
>Cluster 1264
0     13aa, >antibacterial07572... *
1     11aa, >antibacterial09191... at 63.64%
>Cluster 1265
0     13aa, >antibacterial10281... *
>Cluster 1266
0     13aa, >antibacterial10423... *
1     14aa, >antibacterial10424... at 82.11%
2     12aa, >antibacterial10434... at 87.31%
>Cluster 1267
0     12aa, >antibacterial08033... *
1     12aa, >antibacterial10112... at 41.67%
```

The representative sequence denoted with the * symbol at the end of the line; The non-representative sequences displayed with the percentage of sequence similarity to the representative sequence of that cluster.
