# Supplementary material for: Ensemble-AMPPred: Robust AMP Prediction and Recognition Using the Ensemble Learning Method with a New Hybrid Feature for Differentiating AMPs
Source: Genes (Basel). 2021 Jan 21;12(2):137. doi: 10.3390/genes12020137 (PMC7911732; doi:10.3390/genes12020137)
Supplement: Supplementary file 1 [file genes-12-00137-s001.zip › Ensemble-AMPPred_supplement/Supplementary File S4.pdf]

Performance comparison of machine learning models when trained with imbalanced datasets with the ratio of AMPs to nonAMPs equal to 1:2 (900 AMPs and 1800 non-AMPs).

Model	MLP	SVM	KNN	RBF	LDA	NB	DT	DL
Training Accuracy	80.26%	85.48%	86.48%	83.03%	84.07%	78.67%	77.96%	83.59%
Training AUC	0.798	0.874	0.902	0.868	0.906	0.863	0.682	0.906
Training True Positive (TP)	0.607	0.729	0.725	0.788	0.712	0.808	0.591	0.655
Training True Negative (TN)	0.901	0.918	0.935	0.852	0.905	0.776	0.874	0.926
Training False Positive (FP)	0.099	0.082	0.065	0.148	0.095	0.224	0.126	0.074
Training False Negative (FN)	0.393	0.271	0.275	0.212	0.288	0.192	0.409	0.345
Testing Dataset 1								
AMP (11,634)	5,869 *50.44%*	6,604 *56.76%*	8,417 *72.35%*	7,626 *65.54%*	6,713 *57.70%*	6,171 *53.04%*	6,975 *59.95%*	7,427 *63.84%*
Non-AMP (35,795)	33,557 *93.75%*	34,130 *95.34%*	33,983 *94.94%*	35,006 *97.79%*	34,467 *96.29%*	29,976 *83.74%*	31,586 *88.24%*	34,857 *97.38%*
Testing Dataset 2								
AMP_S1 (1,461)	880 *60.26%*	859 *58.79%*	994 *68.03%*	727 *49.76%*	761 *52.09%*	643 *44.01%*	576 *39.42%*	827 *56.60%*
AMP_S2 (917)	516 *56.27%*	586 *63.90%*	662 *72.19.%*	492 *53.65%*	560 *61.07%*	498 *54.31%*	559 *60.96%*	527 *57.47%*
Non-AMP S1 (2,404)	1,950 *81.11%*	2,067 *85.98%*	2,108 *87.69%*	1,963 *81.65%*	2,000 *83.19%*	1,755 *73.01%*	1,946 *80.95%*	2,177 *90.56%*
Non-AMP S2 (828)	694 *83.82%*	731 *88.28%*	784 *94.69%*	791 *95.53%*	795 *96.01%*	662 *79.95%*	732 88.40%	753 *90.95%*

Performance comparison of machine learning models when trained with imbalanced datasets with the ratio of AMPs to nonAMPs equal to 1:3 (600 AMPs and 1800 non-AMPs).

Model	MLP	SVM	KNN	RBF	LDA	NB	DT	DL
Training Accuracy	79.08%	81.92%	81.12%	77.71%	85.75%	73.12%	84.87%	87.21%
Training AUC	0.708	0.838	0.843	0.768	0.807	0.728	0.785	0.903
Training True Positive (TP)	0.331	0.445	0.371	0.411	0.666	0.592	0.721	0.735
Training True Negative (TN)	0.944	0.944	0.958	0.899	0.921	0.778	0.891	0.918
Training False Positive (FP)	0.056	0.056	0.042	0.101	0.079	0.222	0.109	0.082
Training False Negative (FN)	0.669	0.555	0.629	0.589	0.334	0.408	0.279	0.265
Testing Dataset 1								
AMP (11,634)	4,558<br>*39.18%*	6,324<br>*54.36%*	7,053<br>*60.62%*	5,337<br>*45.87%*	6,423<br>*55.21%*	5,794<br>*49.80%*	4,796<br>*41.22%*	5,857<br>*50.34%*
Non-AMP (35,795)	34,226<br>*95.62%*	34,978<br>*97.71%*	35,346<br>*98.75%*	33,142<br>*92.59%*	33,878<br>*94.64%*	30,901<br>*86.33%*	25,962<br>*72.53%*	33,740<br>*94.26%*
Testing Dataset 2								
AMP_S1 (1,461)	758<br>*51.88%*	685<br>*46.89%*	774<br>*52.97%*	663<br>*45.37%*	696<br>*47.64%*	599<br>*40.99%*	386<br>*26.42%*	713<br>*48.83%*
AMP_S2 (917)	411<br>*44.82%*	572<br>*62.38%*	506<br>*55.17%*	411<br>*44.82%*	571<br>*62.27%*	464<br>*50.59%*	369<br>*40.23%*	440<br>*47.98%*
Non-AMP S1 (2,404)	2,317<br>*96.38%*	2,093<br>*87.06%*	2,260<br>*94.01%*	2,116<br>*88.02%*	2,117<br>*88.06%*	1,945<br>*80.91%*	2,007<br>*83.48%*	2,189<br>*91.06%*
Non-AMP S2 (828)	827<br>*99.87%*	801<br>*96.73%*	826<br>*99.76%*	780<br>*94.20%*	784<br>*94.68%*	652<br>*78.74%*	735<br>*88.76%*	811<br>*97.95%*
